# Novel uncharged triazole salicylaldoxime derivatives as potential acetylcholinesterase reactivators: comprehensive computational study, synthesis and *in vitro* evaluation†

**DOI:** 10.1039/d3ra05658a

**Published:** 2023-09-28

**Authors:** Mohammad Hadi Baghersad, Azizollah Habibi, Arash Dehdashti nejad

**Affiliations:** a Applied Biotechnology Research Center, Baqiyatallah University of Medical Sciences Tehran Iran Hadibaghersad@bmsu.ac.ir; b Faculty of Chemistry, Kharazmi University No. 43, P. Code 15719-14911, Mofatteh Street, Enghelab Ave. Tehran Iran

## Abstract

The present study aims to design and synthesise novel uncharged aldoximes and explore their reactivation abilities, structures, descriptors, and mechanisms of action, as well as assessing the interactions and stabilities in the active site of paraoxon-inhibited acetylcholinesterase enzyme using computational studies and *in vitro* assay. The comprehensive computational studies including quantum chemical, molecular dynamics simulations and molecular docking were conducted on paraoxon-inhibited human acetylcholinesterase to investigate the reactivation ability of the novel aldoximes and compare them with pralidoxime as a reactivator model molecule.

## Abstract 2

We design and synthesis new aldoximes without charge and test their reactivation, structure, and action of paraoxon-blocked acetylcholinesterase using calculations and experiments.

## Graphical abstract

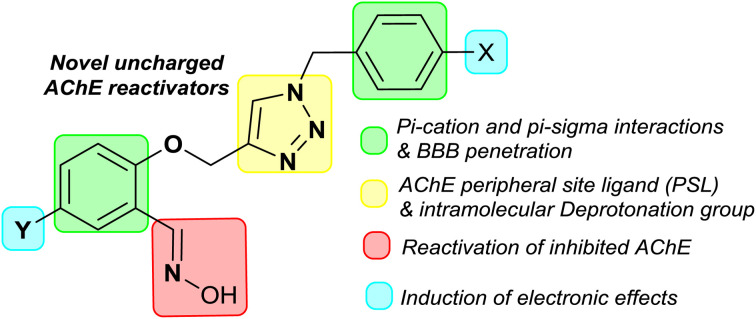

## 1. Introduction

**Fig. 1:**
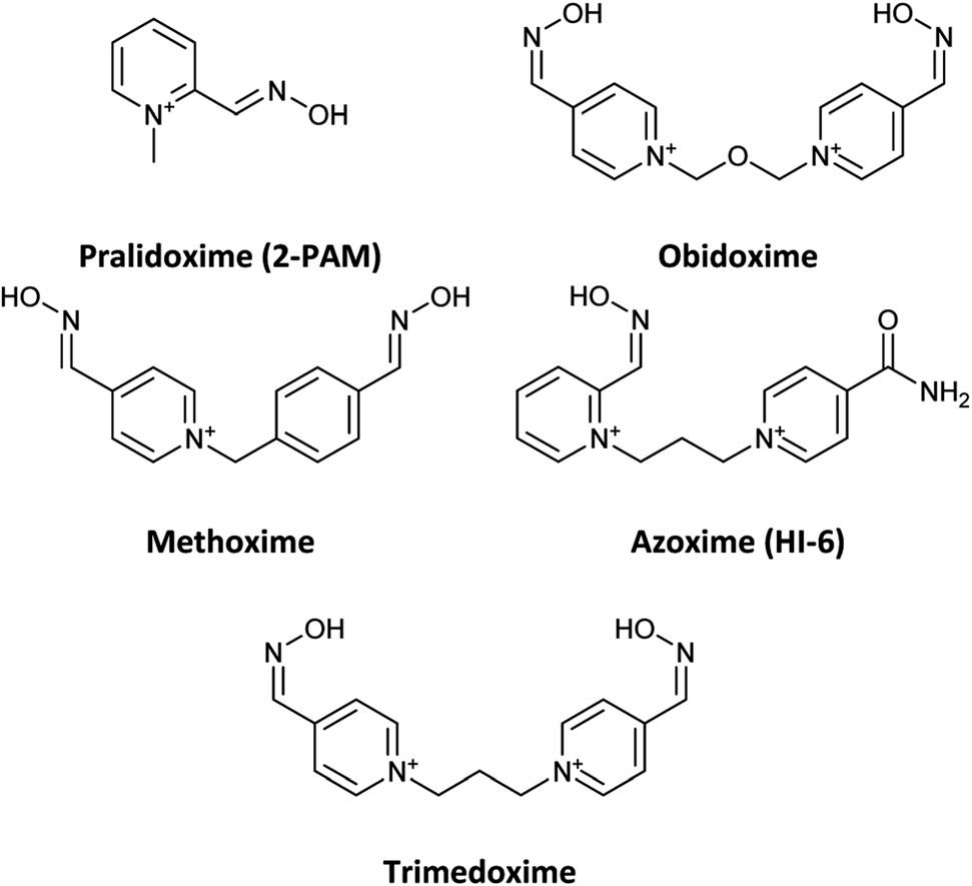
Name and chemical structure of oximes are used to treat OPs poisoning.

Organophosphates (OPs) are the most effective neurotoxic chemical warfare agents capable of inhibiting the acetylcholinesterase enzyme (AChE).^1^ Inhibition of AChE causes the neurotransmitter acetylcholine (ACh) to overstimulate cholinergic receptors, resulting in acute symptoms including convulsions and death.^2^ Under normal circumstances, the hydroxyl group of the amino acid serine attacks ACh in the active site of the enzyme. The acetylated serine is subsequently hydrolysed, and the enzyme's active site prepares to accept the next acetylcholine molecule.^3^ Similarly, OPs may easily reach the enzyme's active region and react with the serine, resulting in a phosphorylated enzyme complex. The phosphorylation of serine causes the inhibition of AChE. In terms of chemistry, and according to the recognized mechanism for reactivating inhibited AChE, a nucleophilic substitution reaction is required to break the covalent bond formed between serine oxygen and organophosphate phosphorus atoms.^4^ In reality, the nucleophile assault on the phosphoryl–enzyme complex in the first step causes an addition reaction, and the enzyme is subsequently liberated as a leaving group in the second step.^5^ Under physiological conditions, many substances with at least one nucleophilic moiety, such as oximes, hydroxamic acids, hydroxylamines, hydrazones, guanylhydrazones, and so on, can reactivate the inhibited AChE to a certain extent.^6^ Among these, oximes, particularly cationic oximes like pyridinium aldoximes, are easily deprotonated in the AChE active site, resulting in effective AChE reactivation. Since the discovery of pralidoxime (2-PAM) in 1955, a plethora of oximes have been synthesized, firstly to identify an efficient antidote against the nerve agent soman, and subsequently to discover central nervous system (CNS) active reactivators and oximes with a wider range against OPs. Today, oxime-based medicines such as pralidoxime, obidoxime, trimedoxime, methoxime, and asoxime (HI-6) are used to treat OPs poisoning (Fig. 1).^7^

**Fig. 2:**
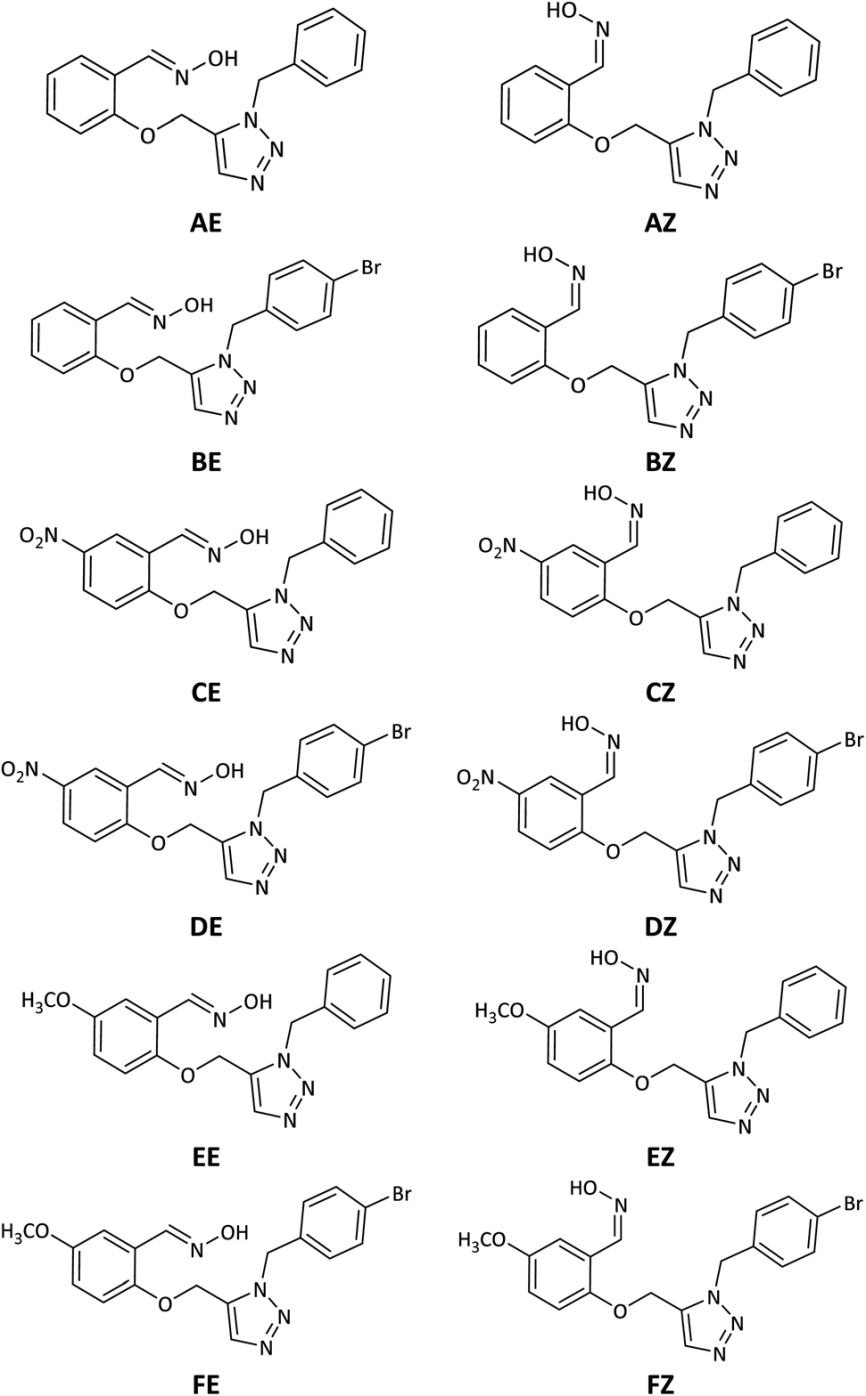
Schematic representation of the 2D structure of the designed triazole salicylaldoxime derivatives.

However, despite the fact that several cationic oximes have been proven to efficiently reactivate AChE, none of them has been proved effective against all known OPs. Moreover, cationic oximes have difficulty penetrating the blood–brain barrier (BBB), which includes reducing their effectiveness. Molecules that can pass across the BBB should have a molecular polar surface area (PSA) of less than 60 Å, a favourable interaction with human serum albumin (HSA), adequate fat solubility, and a mass of less than 400 Daltons.^8^ According to the literature, BBB penetration takes place in two stages: drug partitioning into the hydrophilic moieties of the phospholipids present on the BBB membrane surfaces, which is often aided by drug ionization, and continuing BBB penetration. It occurs by passing through the lipophilic inner moiety of the phospholipid bilayer, which requires the molecule to be in the uncharged form. These circumstances need the presence of protonation and deprotonating groups in molecules able to cross the BBB.^9^ The discovery of novel uncharged reactivators has received greater attention in recent years due to the difficulty that permanently charged oximes have in crossing the blood–brain barrier. As a result, several research on developing uncharged oximes with the objective of crossing the BBB have been reported in the literature, utilizing diverse techniques and structural variations.^10,11^ Furthermore, numerous uncharged oximes which have less potent in compared with the pralidoxime, obidoxime, and HI-6 were presented recent years.^12–16^ In this work, considering the importance of the issue, several uncharged aldoximes were designed based on the parameters affecting the reactivating property and the ability to cross the BBB (Fig. 2). Quantum chemical studies, molecular docking and molecular dynamic (MD) simulations were used to investigate the interaction and behaviour of the designed molecular structures complexed with paraoxon-inhibited human AChE. In the next step, the structures with the highest reactivation potential and crossing the BBB were synthesized, and finally, their ability to activate the paraoxon-inhibited human red blood cell (RBC) AChE was tested *in vitro*.

## 2. Experimental

### 2.1. Computational methods

The organophosphorus paraoxon-inhibited Ser203 residue with a protonated His447 imidazole ring was taken from the PDB structure of the human AChE complexed with paraoxon and 2-PAM (PDB:5HFA) for theoretical computations.^17^ Hereafter, the paraoxon-inhibited Ser203 residue is called SON203 and the inhibition process of the active Ser203 by the organophosphorus compound paraoxon is termed as SON. Optimizations were performed using this paraoxon-modelled inhibited enzyme. All calculations were performed in Gaussian 09 suite of programs.^18^ All stereo-optimizations were performed at the M062X/6-31G* level in an aqueous phase by the polarizable continuum model (PCM) using the integral equation formulation (IEF-PCM).^19–23^ The default UFF radii, including explicit hydrogen atoms, were considered. Frequency calculations have been performed for validating the optimized geometrical structure of the species. In this approach, reactants, intermediates or products correspond to minimum points on the potential energy surface (PES) and should lack an imaginary (negative) frequency. Nonetheless, transition states are first-order saddle points and have only one imaginary frequency. The empirical nucleophilicity index (*ω*^−^) was calculated as follows.^24,25^1
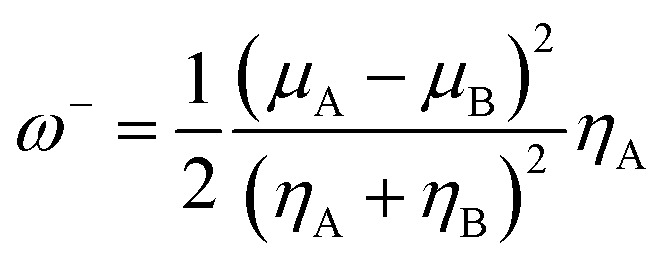
where *μ*_A_ and *μ*_B_ are corresponding chemical potentials, and *η*_A_ and *η*_B_ are corresponding hardnesses, A is the nucleophile (oxime), and B is the electrophilic OP-inhibited serine in the reactivation process. Chemical potentials and hardnesses were calculated using the energies of the frontier molecular orbitals, HOMO and LUMO, *ε*_H_ and *ε*_L_, as *μ* ≈ (*ε*_H_ + *ε*_L_)/2 and *η* ≈ *ε*_L_ − *ε*_H_, respectively.

The sequence of amino acid residues at the active site of human and mouse AChE enzymes is identical. Consequently, they can be used instead of each other in empirical and theoretical studies as an approximate.^26,27^ For docking and MD simulations, the PDB structure of the human AChE enzyme complexed with paraoxon and 2-PAM (PDB:5HFA) was obtained from the Protein Data Bank. After identifying missing residues (Pro259, Gly260, Gly261, Thr262, Gly263, and Gly264) in 5HFA, “fill in” option in Modeller was used to treat missing residues. After that, the drug molecule of 2-PAM was removed to remain only the paraoxon-inhibited protein. MacroModel was used to add hydrogen atoms to the protein structure and perform energy minimization using the MMFF force field and the PRCG method.^28^ This structure was applied in docking studies by Autodock4.2.^29^ The oxime molecules were optimized before docking studies in Gaussian 09 at the M062X/6-31G* level of theory. Docking simulation was performed in Autodock, where the drug molecules (ligand) were developed at six spatial degrees of freedom (DOFs) such as rotation, translation, and torsion. The interaction energy was calculated at each step to achieve the global minimum energy. A stochastic search algorithm, the Lamarckian genetic algorithm (LGA), was used in Autodock for searching the grid space and evaluating energy on the ligand position considering the target energy grids. A local-based search genetic algorithm (GALS) was used for docking probability calculations. The docking parameter file was prepared for each reactivator considering 100 genetic algorithm (GA) and 150 local search (LS) runs along with 3000 interactions.^30^ The Kollman charges were added to the protein molecule, and charge deficiency was disturbed on all protein atoms. Only polar hydrogen atoms in drugs and protein were considered. The grid map was prepared by Autogrid using a 70 × 70 × 70 Å grid box, including the ligand.

After docking, the protein–drug complexes were examined based on the distance between the phosphorus (P) atom of the SON203 residue and the oxygen (O) atom of oxime for all reactivators, and the complexes with the least distance were attended in MD simulation. MD simulations were carries out by the GROMACS 5.1.4 package using the Gromos96 force field.^31^ As this force field lacks the parameters required for simulating the SON residue and underexamined drugs, therefore the ATB server was used to generate required files for GROMACS computations, and the obtained parameters were included in the Gromos96 force field.^27,32,33^ The complexes were immersed in the cuboid box filled with water molecules at a distance of 10 Å away from the box wall. Suitable ions were used to neutralize the boxes. The flexible SPC water model was applied along with the steepest descent algorithm for energy minimization of each system and relaxation of solvent molecules.^34^ Simulations were performed for 1 ns in NVT and NPT ensembles to equilibrate the designed systems. Finally, MD simulation was implemented for all systems for 20 ns with a time step of 2 fs with the LINCS algorithm to constrain the bond lengths.^35^ For each system component, the PME algorithm was applied to evaluate electrostatic interactions.^36^ The V-rescale coupling and the Berendsen algorithm were used to maintain a constant temperature and pressure for all components during simulations, respectively.^37,38^

Discovery Studio Visualizer was used to examine interactions, and generate 2D diagrams of interactions, and also 3D docking images.^39^ In addition, GROMACS tools were applied to process different analyses on the MD simulation trajectory.

### 2.2. Chemistry

#### 2.2.1. General

All materials used were purchased from Merck. Analytical thin layer chromatography (TLC) was performed on precoated silica gel 60 F254 plates. Visualization on TLC was achieved by UV light (254 nm). IR Spectra were recorded on a PerkinElmer Spectrum RXI FT-IR spectrometer; in cm^−1^. ^1^H and ^13^C-NMR Spectra were recorded on Bruker AMX-300 (^1^H NMR 300 MHz and ^13^C NMR 75.4 MHz) spectrometer; in CDCl_3_ or DMSO; *δ* in ppm, *J* in Hz. Melting points were determined with a Electrothermal 9200 apparatus. Elemental analyses were performed using a PerkinElmer 2004 series [II] CHN elemental analyser.

#### 2.2.2. Synthetic procedures

##### 2.2.2.1. *O*-Propargylated salicylaldehyde

Propargyl bromide (7.40 mmol) and anhydrous K_2_CO_3_ (1.00 g) were added to a solution of salicylaldehyde (6.17 mmol) in DMF (15 mL). The mixture was stirred at room temperature for 16 h, and then it was poured on crushed ice. The precipitated product was filtered and dissolved in EtOAc (100 mL). The solution was washed with water (50 mL), brine (50 mL), and water (50 mL), respectively. The organic layer was dried over anhydrous Na_2_SO_4_ and concentrated under vacuum to give *O*-propargylated salicylaldehyde (80–85%). The product was isolated as colourless crystals, mp 68–69 °C. ^1^H NMR (300 MHz, CDCl_3_) *δ* 10.48 (s, 1H), 7.86 (dd, *J* = 7.7, 1.6 Hz, 1H), 7.60–7.54 (m, 1H), 7.14–7.06 (m, 2H), 4.83 (d, *J* = 2.3 Hz, 2H), 2.58 (t, *J* = 2.3 Hz, 1H); ^13^C NMR (75 MHz, CDCl_3_) *δ* 189.4, 159.7, 135.7, 128.3, 125.4, 121.7, 113.2, 77.6, 76.4, 56.3; IR (KBr, cm^−1^): 3270, 2973, 2871, 2116, 1683, 1598, 1458, 1287, 1224, 1193, 1009, 757, 676, 654, 611, 462, 441.

##### 2.2.2.2. *O*-Propargylated salicylaldoxime

A mixture of *O*-propargylated salicylaldehyde (2.40 mmol), pyridine (3.6 mmol), hydroxylamine hydrochloride (2.88 mmol), and 5 mL ethanol was refluxed for 3 hours. The solvent was removed, and the residue was acidified with 6 N HCl. It was extracted with CHCl_3_ (3 × 20 mL), washed with brine, and dried over anhydrous sodium sulfate and solvent was evaporated under vacuum to give *O*-propargylated salicylaldoxime. The product was isolated as colourless crystals, mp 68–69 °C. ^1^H NMR (300 MHz, CDCl_3_): *δ* 9.41 (s, 1H), 8.54 (s, 1H), 7.73 (dd, *J* = 7.7, 1.8 Hz, 1H), 7.47–7.29 (m, 1H), 7.16–6.94 (m, 2H), 4.77 (d, *J* = 2.4 Hz, 2H), 2.56 (t, *J* = 2.4 Hz, 1H). ^13^C NMR (75 MHz, CDCl_3_): *δ* 155.50, 146.38, 131.02, 127.08, 121.64, 121.17, 112.72, 78.10, 75.99, 56.19; IR (KBr, cm^−1^): 3259, 3147, 3017, 2935, 2113, 1600, 1493, 1460, 1450, 1225, 1027, 751, 658, 638, 554, 470.

##### 2.2.2.3. Triazole salicylaldoximes A and B

*O*-Propargylated salicylaldoxime (1–1.8 mmol), benzyl azide or 4-bromobenzyl azide (0.99–1.1 mmol) and [Cp*Ru–Cl(PPh3)2] (1 mol%) were dissolved in anhydrous benzene (or anhydrous THF) (8–10 mL). The resulting mixture was heated to 80 °C and stirred vigorously for 3–4 h. After cooling to r.t., the solvent was removed under vacuum. Purification flash column chromatography (hexanes–EtOAc) provided triazole salicylaldoximes A and B.

###### 2.2.2.3.1 2-((1-Benzyl-1*H*-1,2,3-triazol-5-yl)methoxy)benzaldehyde oxime (A)

Compound A was isolated as pale yellow solid, mp: 92.3–95.4 °C. ^1^H NMR (300 MHz, CDCl_3_) *δ* 9.0 (s, 1H), 8.31 (s, 1H), 7.81–7.62 (m, 2H), 7.37–7.27 (m, 4H), 7.13–7.16 (m, 2H), 7.07–6.95 (m, 1H), 6.78 (d, *J* = 8.3 Hz, 1H), 5.66 (s, 2H), 4.94 (s, 2H). ^13^C NMR (75 MHz, CDCl_3_) *δ* 155.18, 145.45, 134.01, 131.95, 130.92, 128.95, 128.92, 128.52, 127.45, 127.44, 127.37, 127.30, 127.25, 121.29, 111.85, 77.43, 77.00, 76.58, 58.60, 52.65. IR (KBr, cm^−1^) 3238, 3063, 2922, 1600, 1494, 1461, 1295, 1231, 1123, 1016, 958, 752, 709. Anal. calcd for C_17_H_16_N_4_O_2_ (308.34): C, 66.22%; H, 5.23%; N, 18.17% found: 66.16%; H, 5.25%; N, 18.21%.

###### 2.2.2.3.2 2-((1-(4-Bromobenzyl)-1*H*-1,2,3-triazol-5-yl)methoxy)benzaldehyde oxime (B)

Compound B was isolated as pale yellow solid, mp: 126.2–129.0 °C. IR (KBr) 3413, 2921, 2851, 1599, 1490, 1455, 1293, 1246, 1126, 1012, 963, 754. ^1^H NMR (300 MHz, DMSO-d_6_) *δ* 11.18 (d, *J* = 1.6 Hz, 1H), 8.01–7.89 (m, 2H), 7.63 (dd, *J* = 7.8, 1.7 Hz, 1H), 7.61–7.44 (m, 2H), 7.41–7.21 (m, 1H), 7.15 (t, *J* = 8.2 Hz, 3H), 6.98 (t, *J* = 7.5 Hz, 1H), 5.67 (s, 2H), 5.29 (s, 2H). ^13^C NMR (75 MHz, DMSO-d_6_) *δ* 154.88, 142.96, 135.02, 132.86, 131.6, 131.48, 130.50, 129.64, 129.56, 129.50, 125.54, 121.29, 112.76, 58.93, 50.28.

#### 2.2.3. *In vitro* assay

##### 2.2.3.1. Preparation of RBC ghosts

Human blood samples were prepared from Iranian Blood Transfusion Organization, homogenized with 10% EDTA (100 mL) and centrifuged at 3000 rpm for 10 min to separate the plasma and buffy coat. The packed RBCs were washed three times with phosphate-buffered saline (PBS, pH 7.4, 0.1 M) and resuspended in the same buffer to a hematocrit of 40%. The RBCs were then hemolyzed by adding distilled water to a final volume ratio of 1 : 10 and incubated at 4 °C for 30 min with gentle shaking. The hemolysate was centrifuged at 10 000 rpm for 15 min to obtain the RBC ghosts as the pellet. The RBC ghosts were washed three times with PBS and resuspended in the same buffer. The RBC ghosts were stored at −20 °C until further use.^40^

##### 2.2.3.2. Inhibition of RBC ghost acetylcholinesterase by paraoxon

In order to maximum inhibition, first the prepared suspension of RBC ghosts (200 μL) in the previous step was centrifuged. Then 10 μL of the supernatant was discarded and replaced with 10 μL of paraoxon (5 mM, ethanolic solution) and after vortexing, it was incubated for 30 minutes at 37 °C. In order to remove the excess of paraoxon, the suspension was centrifuged again and after separating the supernatant, it was washed twice (each time with 200 μL of phosphate buffer) and centrifuged. Then the RBC ghosts were suspended in 200 μL of phosphate buffer and homogenized. The obtained suspension was used for performing the experiments of measuring the reactivation efficacy and kinetic parameters. RBC AChE activities were measured spectrophotometrically with a modified Ellmann assay.^41,42^

The inhibition effect was calculated according to eqn (2):2
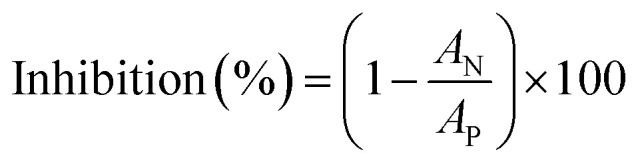
where the *A*_N_ is AChE activity in presence of paraoxon, and the *A*_P_ is AChE activity in control sample.

##### 2.2.3.3. Reactivation of inhibited RBC AChE

The new synthesized reactivators were prepared as a stock solution of 0.2 M in DMSO. The inhibited RBC ghosts (S) were incubated with oximes at 37 °C for 30 min (final concentration of oximes was 0.1 mM). The suspension of uninhibited RBC ghost (P) was used as a positive control. To eliminate the acetylthiocholine iodide oximolysis effect, oxime solution was added to the positive control suspension. The inhibited RBC ghost suspension (N) was used as a negative control and instead of oxime, DMSO was added to it. RBC ghosts AChE activities were measured by the Ellman method as mentioned above. The reactivation effect was calculated according to eqn (3):3
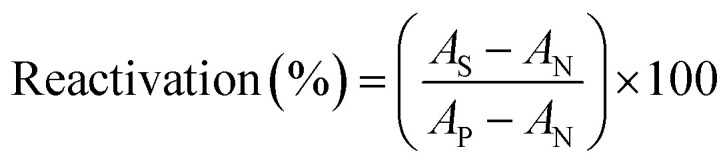


##### 2.2.3.4. Determination of kinetic parameters

In order to determine the reactivation kinetic parameters of the reactivators, the reactivation rate at different time intervals and at different concentrations were measured. The inhibited RBC ghosts were incubated with oximes in various concentrations. The enzyme activity was calculated every 5 minutes, up to 2 hours. At each oxime concentration, observed first-order rate constant of reactivation (*k*_obs_) was calculated from the slope of the initial portion of log(100 − % reactivation) *vs.* time of reactivation plot as *k*_obs_ = −2.303 × slope, assuming an approach to 100% reactivation. Then, *k*_obs_*vs.* [OX] plot was drawn and used to calculate rate constants according to method described by Kovarik *et al.*^43^

## 3. Results and discussion

### 3.1. Molecular structure design

Neutrality, intramolecular protonation–deprotonation possibility and suitable p*K*_a_ of oxime moiety were considered in the design of the novel compounds as the key parameters affecting the reactivating property and the ability to cross the BBB (Fig. 2, structure A–F). Because of their neutrality, as well as the presence of protonation groups in the designed molecules, it is predicted that they will be more capable of reactivating inhibited AChE and crossing the BBB than the cationic oximes. The aldoxime moieties in designed compounds are responsible for removing the phosphorus moiety from the hydroxyl serine residue at the active estearic site of inhibited AChE. The triazole moieties have the ability to deprotonate the oxime hydroxyl groups intramolecularly and increase their nucleophilicity. Additionally, a recent research by Kovarik *et al.* (2013) reveals that the reactivation of inhibited AChE may be enhanced by the use of a peripheral site ligand (PSL), such as a triazole ring. Covalently affixing a PSL to oxime group can improve the binding affinity between AChE and it. This might improve the overall reactivation effectiveness of the structure.^44^ The benzyl moieties were chosen for expected stronger binding *via* pi–cation and pi–sigma interactions with the cholinesterase binding sites. Furthermore, triazole salicylaldoxime derivatives with methoxy, nitro and bromo groups were considered to survey the electronic effects on reactivation activity and BBB penetrating ability of the designed compounds.

### 3.2. Quantum chemical studies

**Fig. 3:**
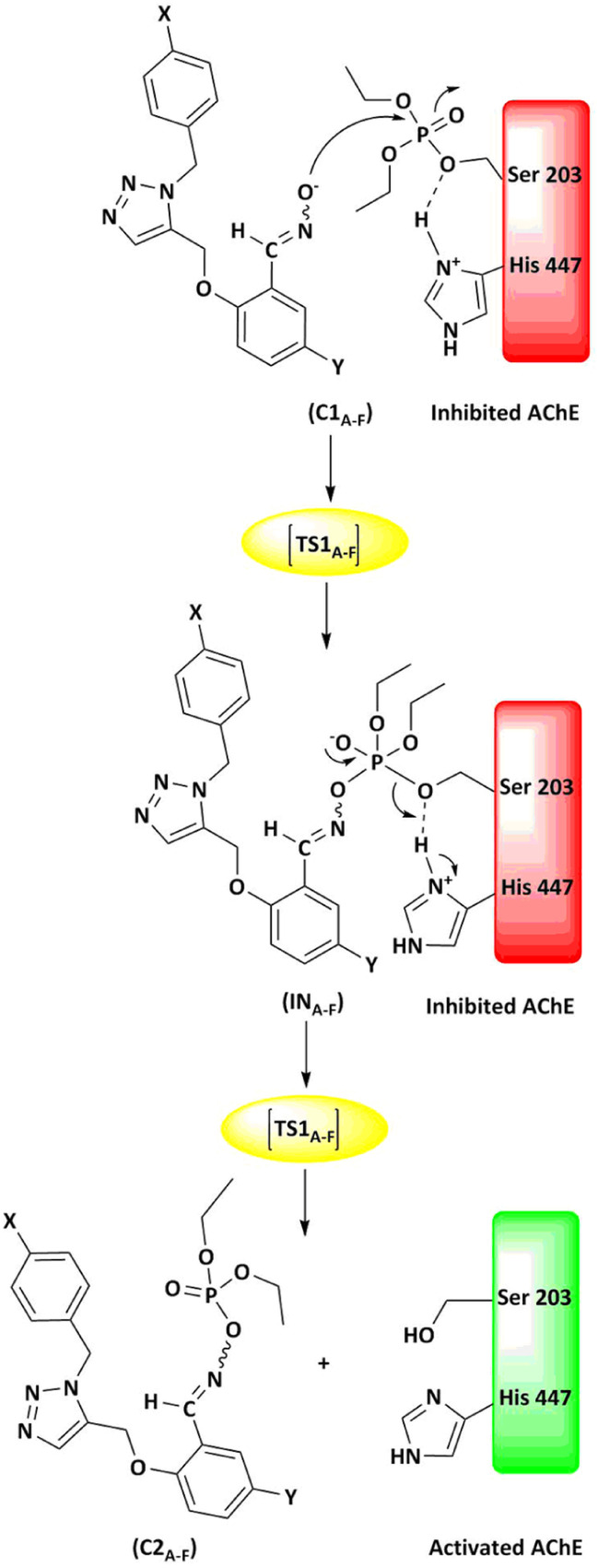
Schematic of the paraoxon-conjugated serine molecule (SON) reactivation process by A–F species.

**Fig. 4:**
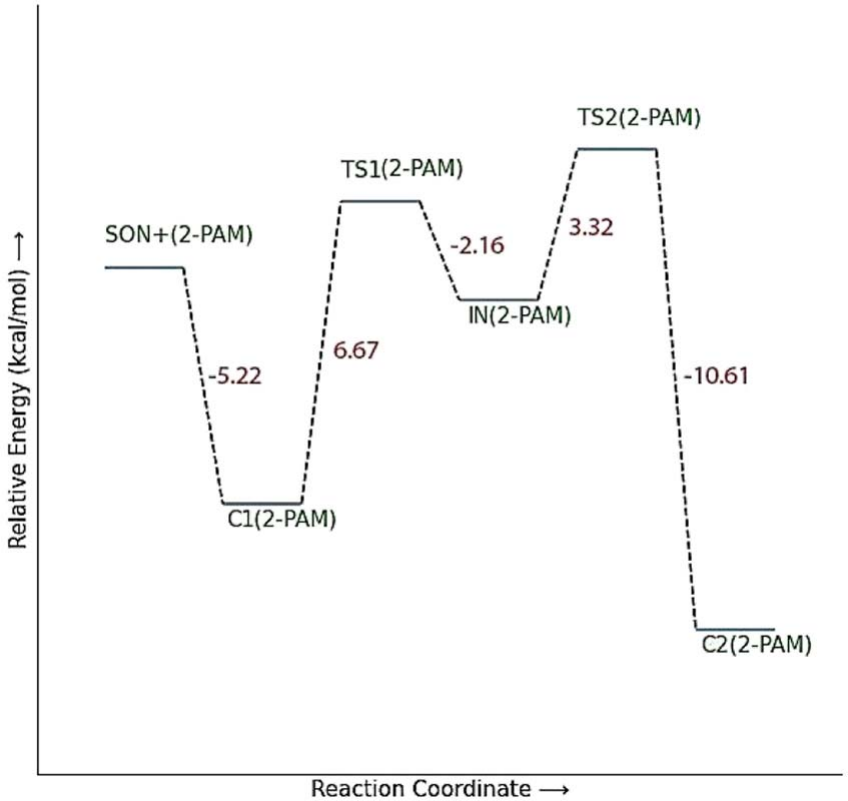
The energy diagram of the optimized structures at the theoretical M062X/6-31G* level for reactivating AChE-paraoxon by 2-PAM in the aqueous phase.

**Fig. 5:**
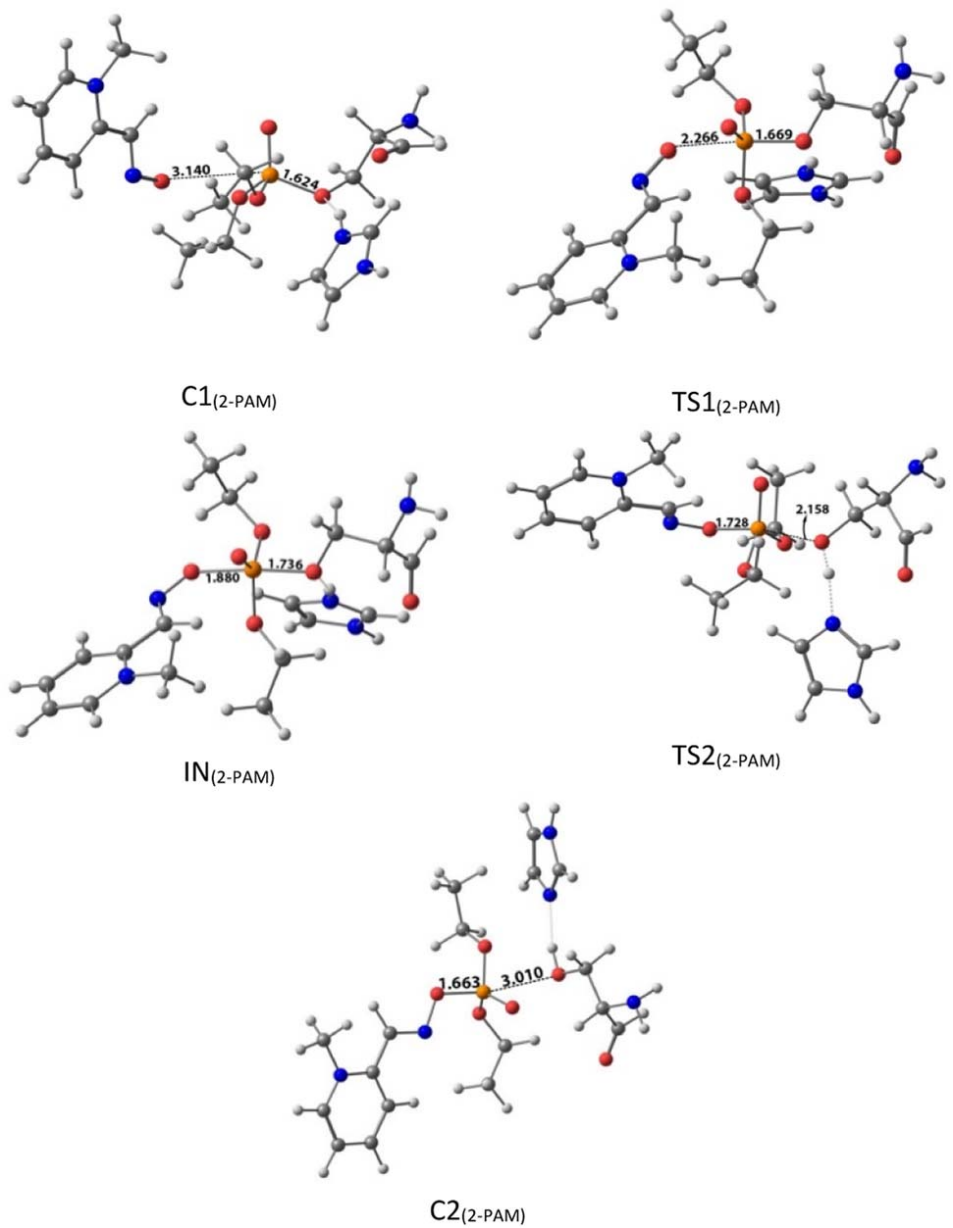
The optimized structures at the theoretical M062X/6-31G* level and the selected bond lengths (Å) of species involved in reactivating SON by 2-PAM in the aqueous phase.

The energy comparison of the optimized structures of the designed compounds (Fig. 2, structure A–F) shows that *E* isomers are more stable than *Z* isomers in all species (ESI File and Table S1†). The compound F showed the highest energy difference of 6.3 kcal mol^−1^, whereas compound C showed the lowest difference of 2.76 kcal mol^−1^ among oxime isomers. The theoretical level of M062X/6-31G* was used to study the mechanism of the reactivation of paraoxon-inhibited human AChE by designed oximes and 2-PAM. The optimal structure of the paraoxon-conjugated Ser203 residue derived from the AChE-paraoxon enzyme was applied in the reactivation process.^45,46^ The reactivation mechanism of the inhibited enzyme by the compounds A–F was studied, and the results were compared with 2-PAM. According to the literature, serine-inhibited AChE is reactivated by nucleophiles involved in an addition–elimination process. AChE-paraoxon reactivation by the desired oximes and pralidoxime follows the same addition–elimination pathway, involving a trigonal bipyramidal intermediate (IN). Thus, two complexes and one intermediate are located on the PES as local minima, and two corresponding transition states link these local minima (Fig. 3). According to the obtained results for 2-PAM, as shown in Fig. 4 and 5, the paraoxon-conjugated serine moiety forms a complex with 2-PAM (C1_(2-PAM)_), which is more energetically stable than the total energy of individual reactants by 5.22 kcal mol^−1^, due to the charge dipole interaction between the anionic nucleophile and SON.

The activation energy calculated for the 2-PAM attack to the P atom in paraoxon is 6.67 kcal mol^−1^ as compared with the C1_(2-PAM)_ complex. The attacking of 2-PAM happened at the opposite site of the serine moiety through and almost linear procedure, forming the first transition state (TS1_(2-PAM)_). The bond distance between the O atom of oxime and the P atom in 2-PAM decreased from 3.14 Å in C1_(2-PAM)_ to 2.266 Å in TS1_(2-PAM)_. A trigonal bipyramidal intermediate is formed after the first transition state (IN_(2-PAM)_), which is 2.16 kcal mol^−1^ more stable than the first transition state. In this structure, the O_(oxime)_ − P_(2-PAM)_ distance decreased to 1.88 Å. On the other hand, the P–O bond distance of serine increased from 1.67 Å in TS1_(2-PAM)_ to 1.736 Å and continues until the end of the process. The protonated histidine residue stabilizes the oxygen atom in the serine group with strong hydrogen bonding, and the length of hydrogen bond in IN(2-PAM) decreases compared to TS1_(2-PAM)_ (from 2 Å to 1.81 Å). The second transition state (TS2_(2-PAM)_) occurs with an activation energy of 3.32 kcal mol^−1^ to release the leaving group of serine. The hydrogen bond turns stronger, and its length becomes shorter in TS2_(2-PAM)_ (1.03 Å) to stabilize the serine group. The second transition state leads to a complex C2_(2-PAM)_ between the leaving group of serine and the paraoxon species bonded to 2-PAM. The distance between the O_(serine)_ and P_(paraoxon)_ atoms decreases from 1.728 Å in TS2_(2-PAM)_ to 1.663 Å in C2_(2-PAM)_. The negatively charged serine species receive the proton from histidine group and stabilize this complex. As indicated in the PES diagram (Fig. 4), the second step of the mechanism, *i.e.*, leaving the enzyme and separation from the P atom in paraoxon, controls the rate of AChE reactivation by 2-PAM, reactivating the AChE-paraoxon drug with an activation energy of 7.83 kcal mol^−1^. AChE-paraoxon is reactivated by all studied oximes through the same addition–elimination pathway with a trigonal bipyramidal intermediate (Fig. 3).

**Fig. 6:**
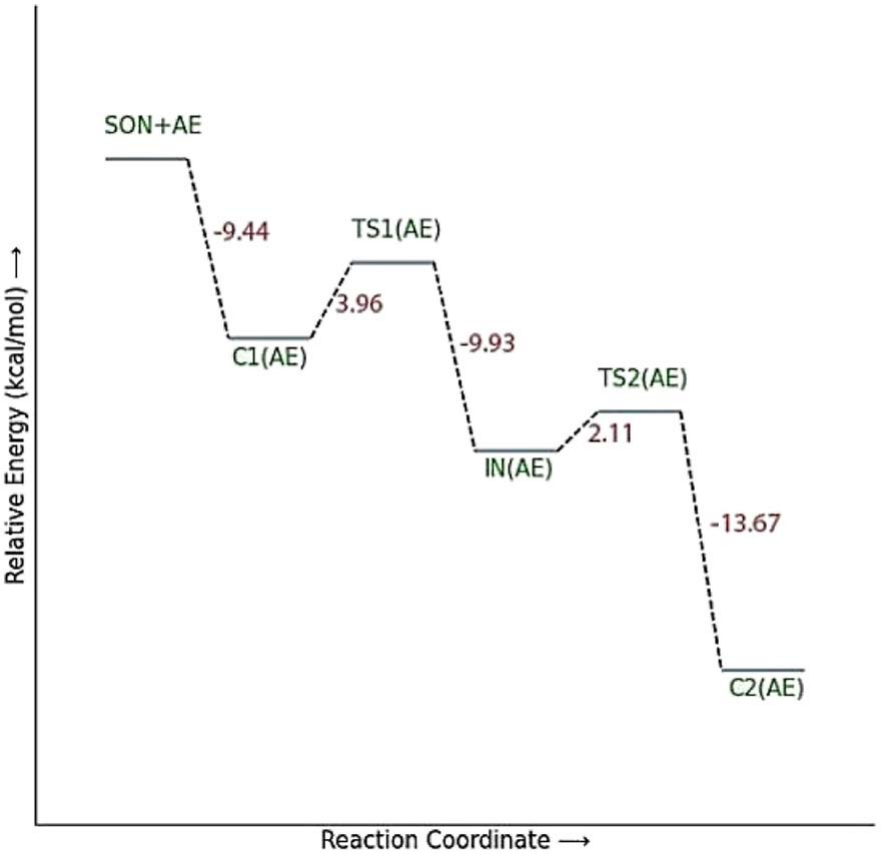
The energy diagram of the optimized structures at the theoretical M062X/6-31G* level for reactivating AChE-paraoxon by AE in the aqueous phase.

**Fig. 7:**
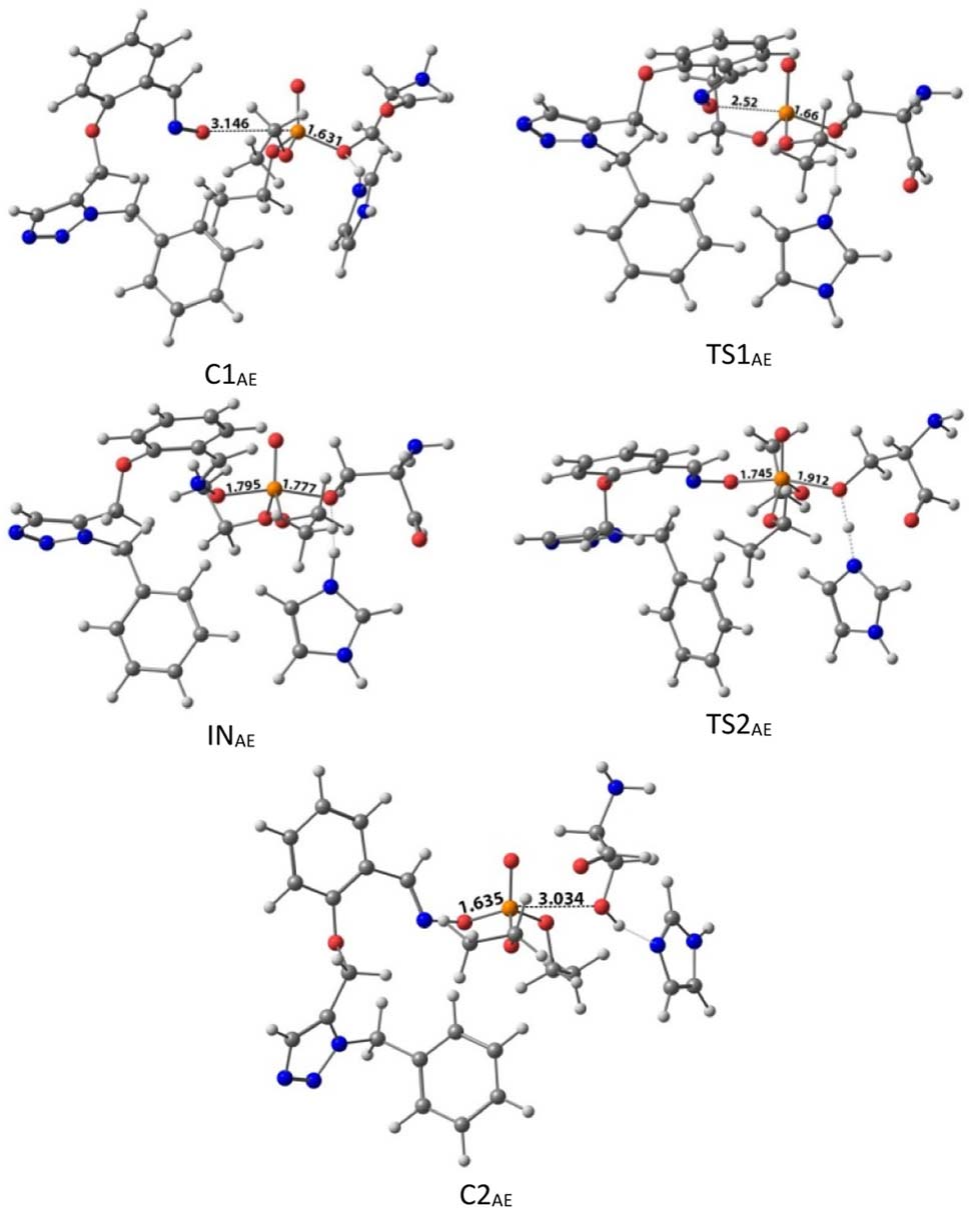
The optimized structures at the theoretical M062X/6-31G* level and the selected bond lengths (Å) of species involved in reactivating SON by AE in the aqueous phase.

The calculated PES and the optimized structures for the AE species have been shown in Fig. 6 and 7. The nucleophilic AE became energetically more stable by 9.44 kcal mol^−1^ than separated reactants after forming a complex with the paraoxon-inhibited enzyme (C1_AE_). As shown in the PES diagram (Fig. 6), the oxime group of AE in TS1_AE_ attacks the P atom in the inhibited enzyme with an energy barrier of 3.96 kcal mol^−1^. According to Fig. 7, the distance between the O atom in AE and the P atom decreases from 3.146 Å in C1_AE_ to 2.52 Å in TS1_AE_. A trigonal bipyramidal intermediate (IN_AE_) is formed in the next step, which is more stable than the first transition state (TS1_AE_) by 9.93 kcal mol^−1^, and the distance between the O atom in AE and the P atom in paraoxon decreases to 1.795 Å. On the other hand, the distance between the O atom of the serine group and the P atom increases from 1.66 Å in TS1_AE_ to 1.78 Å in IN_AE_, and simultaneously, the O atom in the serine group establishes hydrogen interactions with the protonated histidine residue to be stabilized. With increasing the distance between the O atom in serine and the P atom from 1.78 Å in IN_AE_ to 1.912 Å, the second transition state (TS2_AE_) occurs with an energy barrier of 2.11 kcal mol^−1^ for the release of the serine leaving group. As the hydrogen bond between the oxygen atom in serine and the charged histidine residue becomes stronger and shorter, the second transition state causes the formation of the complex C2_AE_ between the leaving group of serine and the paraoxon species bonded to AE. The PES diagrams and the optimized structures involved in AChE reactivation by other triazole salicylaldoxime derivatives are presented in ESI File (Fig. S3 to S14).† According to these figures, it is observed that in general the reactivation process is similar to AE for all species. Also, for all studied neutral oximes, the first step of the enzyme reactivation process, *i.e.*, anionic species attack to the P atom in AChE-paraoxon, is rate-determining step; unlike 2-PAM, for which the second step of the reactivation mechanism, *i.e.*, enzyme leaving and its separation from paraoxon, controls the reaction rate.

In general, *E* isomers have lower activation barrier energy than *Z* isomers in reactivating the inhibited enzyme, except for species F for which the activation energy of *Z* isomers is less than *E* isomers by 0.19 kcal mol^−1^. Among the studied *Z* isomers, the highest and lowest activation energies of 5.26 and 4.29 kcal mol^−1^ were respectively observed for CZ and BZ. Among the studied *E* isomers, the highest and lowest activation energies of 4.95 and 3.21 kcal mol^−1^ were respectively belonged to DE and BE. In other words, both B isomers showed the lowest activation energy among the studied oximes. After species B, oxime A exhibited lower activation energy among the studied species. Accordingly, from mechanistic standpoint, oximes B and, A outperformed other oximes. In general, all considered neutral oximes have a lower activation energy than the cationic oxime, like pralidoxime, indicating the better performance of these neutral oximes than 2-PAM in reactivating AChE-paraoxon. Similarly, Lo *et al.* reported that in the reactivation mechanism of the tabun-inhibited AChE enzyme, in contrast to the studied charged oximes, where the second step of the reactivation process was the rate-determining step; for neutral oxime *N*-(pyridin-2-yl) hydroxylamine, the first step was the determining rate, and the activation energy of this process for this neutral oxime was much less than that of the charged oximes.^47^

The results of CHelpG charge analyses have been indicated in Table 1. According to the results, there are more negative charges on the oximic oxygen of triazole salicylaldoxime derivatives than 2-PAM. Furthermore, oximes A, B, and F have the highest negative charge on their oximic oxygen, indicating higher nucleophilicity of these oximes. The nucleophilicity index (*ω*^−^) of drugs was also studied. This index is a robust, comprehensive descriptor for the nucleophilicity of a nucleophilic species relative to an electrophilic species. The efficiency of the reactivation process is dependent on the nucleophilicity of the reactivating molecule. The calculated nucleophilicity index in Table 1 shows that 2-PAM with *ω*^−^ = 0.0058 eV has the lowest nucleophilicity index among the studied species, indicating higher nucleophilicity of neutral oximes than 2-PAM; as a result, these nucleophilic compounds act more effectively. As shown in Table 1, oximes A, E, and B have the highest *ω*^−^ among the studied molecules. It is worth nothing that the drugs reactivating OP-inhibited AChE can penetrate the BBB and affect the performance of the central nervous system. However, charged oximes are unable to penetrate the BBB and thus cannot reactivate AChE in the central nervous system.^48^ According to the literature, neutral oximes can more effectively pass through the BBB for reactivating AChE in the central nervous system.^44,49–52^ The penetration of reactivators to the BBB is dependent on their lipophilicity and is inversely related to their ionization degree.^52^ It is, therefore, useful and necessary to examine the ability of compounds to penetrate the BBB. Such a property is studied by calculating log *P*, indicating the lipophilicity/hydrophilicity of oximes.^53,54^ Low log *P* values indicate a more hydrophilic nature and less penetration to the BBB. The partition coefficient of octanol/water (log *P*) was calculated by the online server ALOGPS 2.1.^55^

**Table 1:** Calculated properties of nucleophiles and electrophiles at the theoretical M062X/6-31G* level in the aqueous phase

	Hardness (*η*) (a.u.)	Chemical potentials (*μ*) (a.u.)	*ω* ^−^ (eV)	CHelpG
2-PAM	0.21668	−0.13523	0.0058	−0.422
AE	0.23178	−0.09306	0.0453	−0.598
BE	0.22575	−0.09723	0.0395	−0.599
CE	0.17414	−0.13108	0.0076	−0.563
DE	0.17511	−0.13176	0.0072	−0.572
EE	0.23322	−0.09346	0.0447	−0.591
FE	0.22720	−0.09792	0.0387	−0.600
AZ	0.21905	−0.09199	0.0463	−0.510
BZ	0.20655	−0.09834	0.0375	−0.507
CZ	0.17499	−0.12462	0.0115	−0.481
DZ	0.17514	−0.12476	0.0114	−0.480
EZ	0.22010	−0.09289	0.0451	−0.499
FZ	0.20837	−0.09886	0.0369	−0.502
Paraoxon-inhibited AChE	0.31782	−0.1589		

According to the results in Table 2, the drug 2-PAM as a charged oxime shows negative log *P*, indicating its inability to penetrate the BBB. The uncharged oximes showed positive log *P*, indicating higher lipophilicity of these neutral pharmaceutical candidates. Also, positive log *P* values indicate higher penetrability of the designed reactivators in the BBB.

**Table 2:** The octanol–water partition coefficient (log *P*) of different oximes

Compound	2-PAM	A	B	C	D	E	F
Log *P*	−3.04	2.31	3.39	3.16	3.20	2.48	3.2

### 3.3. Molecular docking studies

**Fig. 8:**
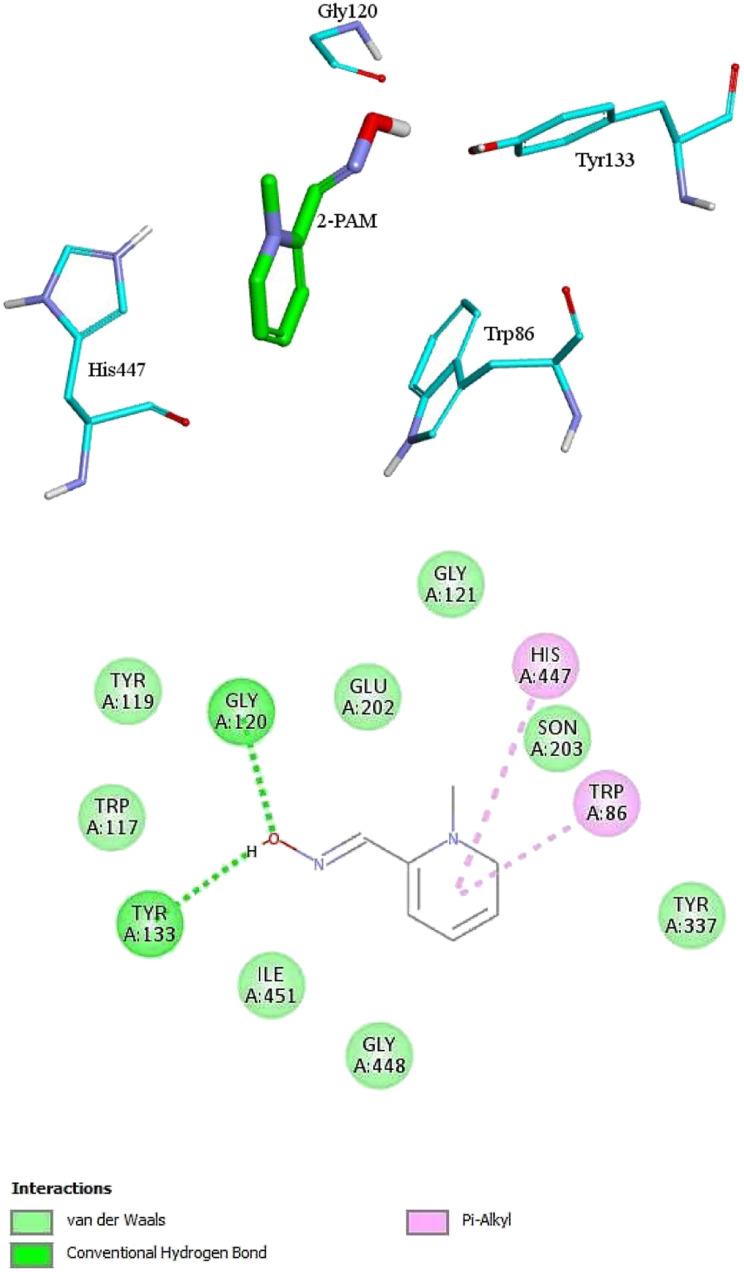
The 2D and 3D structures of interactions between the amino acids of the active site of enzyme AChE-paraoxon and 2-PAM oxime.

**Fig. 9:**
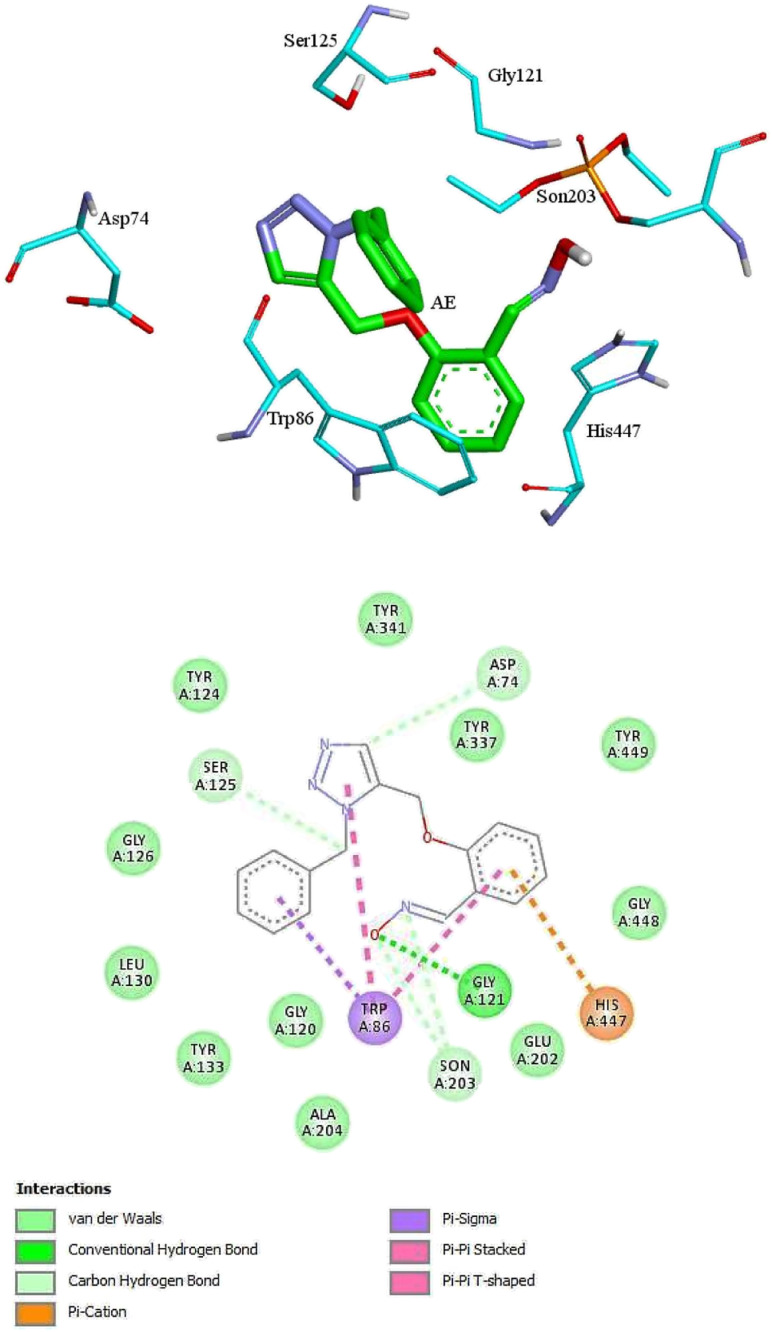
The 2D and 3D structures of interactions between the amino acids of the active site of enzyme AChE-paraoxon and AE oxime.

For further analysis, docking studies of the desired drugs and the AChE-paraoxon enzyme were carried out. Molecular docking can predict the position and orientation of drug binding in the enzyme active site, which plays a key role in the design of more effective reactivators. Considering energy ranking, these studies predict the ligand–acceptor bond conformation. Our fundamental criteria to select docking positions were the shortest distance between the oxime oxygen atom and the P atom in the organophosphorus paraoxon, which plays a critical role in the reactivation process, and higher stability. As presented in ESI File and Table S2,† the designed oximes have a more negative binding energy than 2-PAM. Also, the number of interactions between each of the uncharged oximes and the enzyme is greater than that between 2-PAM and the enzyme. Whereas, hydrogen bonds and hydrophobic interactions are more effective than noncovalent interactions in stabilizing drug molecules in the enzyme active site, this demonstrates that uncharged oximes are more stable in the enzyme active site, as confirmed by binding energies obtained from docking calculations. The docking results for 2-PAM in Fig. 8 display that this oxime establishes hydrogen bonds with TYR133 and GLY120 residues in the active site. Moreover, TRP86 and HIS447 that are catalytic anionic site (CAS) enzyme residues, have pi–alkyl interaction with 2-PAM. Other interactions between oximes and the enzyme are listed in ESI File and Table S2.† The calculated binding energy for 2-PAM is −4.96 kcal mol^−1^, which is the highest binding energy for the studied oximes, and the O_(Oxime)_ − P_(paraoxon)_ distance in the complex AChE-paraoxon/2-PAM equals 0.73 nm, which is the largest distance compared to other oximes. Docking studies were also conducted for uncharged oximes. Due to the structural similarity of the candidate drugs, they have almost the same interactions with the residues of the enzyme active site. As is clear from Fig. 9, there is hydrogen interaction between AE and the GLY121 residue, and the HIS447 residue makes a strong pi–cation interaction with the AE benzene ring.

In addition, the aromatic TRP86 residue has pi–sigma interactions with the AE benzyl group, and pi–pi interactions with the benzene and triazole rings. Oxime AE has hydrophobic interactions with the aromatic residues TYR341 and TYR124 from the peripheral anionic site (PAS) and the residues GLU202 and TYR337 from the catalytic anionic site. Docking structure indicates that AE has a binding energy of −6.72 kcal mol^−1^ and a O_(Oxime)_ − P_(paraoxon)_ distance of 0.37 nm in the complex with AChE-paraoxon. The docking results for AZ (ESI File and Fig. S15a†) demonstrate that, like AE, the GLY121 residue has hydrogen interaction, and the TRP86 residue has pi interaction with triazole, benzene, and benzyl rings of AZ. Moreover, AZ has other hydrophobic interactions with PAS residues such as ASP74, TYR124, and TYR341 and CAS residues such as TRP86, GLY122, GLU202, and HIS447.

The O_(Oxime)_ − P_(paraoxon)_ distance in the AZ structure obtained from docking calculations is 0.33 nm with a binding energy of −6.94 kcal mol^−1^. The docking results for other oximes have been shown in ESI File and Fig. S15.† The presence of NO_2_, OCH_3_, and bromine (Br) substitutes in the studied oximes has caused interactions between the oximes and the enzyme. For instance, in the structures obtained from docking calculations, the residues of TYR133 for CE, ALA127 for CZ, and TYR133 and ALA127 for DE and DZ contribute to creating a hydrogen bond with the NO_2_ substitute of these oximes.

In addition, the residues of TYR119 and TYR133 for EE, TYR341 for EZ, TYR119 and TYR133 for FE, and TYR72 and TRP124 for FZ establish noncovalent pi–alkyl interactions with the OCH_3_ substitute of these oximes.

In the case of Br substitute, for example, in the enzyme–FE complex, the CAS residues TYR337, TYR449, HIS447, and TRP86 have noncovalent interactions with Br. It is noteworthy that, as shown in ESI File and Table S2,† for all uncharged oximes, the number of interactions with the CAS site of the enzyme is larger than the PAS site, which may play an effective role in the greater reactivation effect of these oximes.

### 3.4. Molecular dynamic simulation

**Fig. 10:**
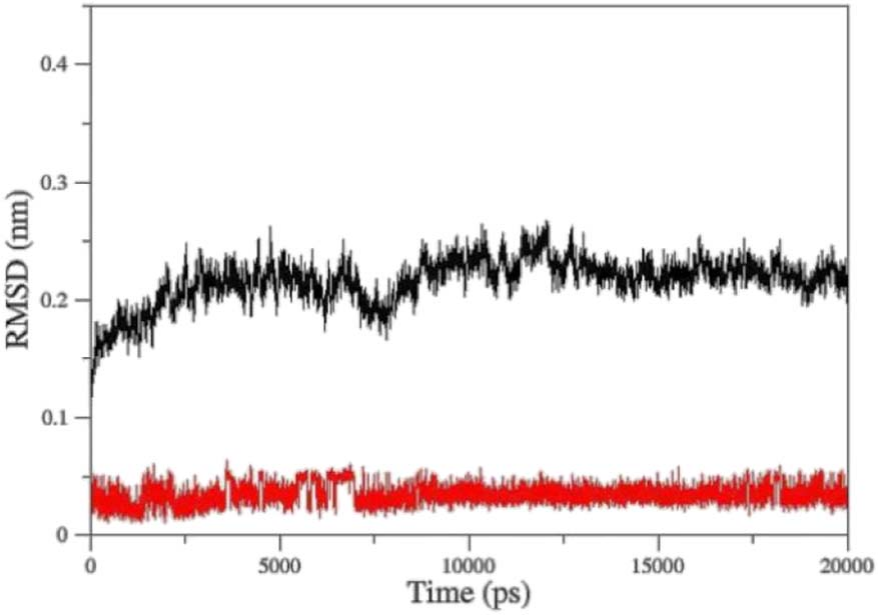
Cα-RMSD *versus* time for AChE-paraoxon (black)/2-PAM (red) in the AChE-paraoxon/2-PAM system.

Molecular Dynamic (MD) simulation was conducted to gain a better understanding of the drug–protein complex. The efficiency of oximes is dependent on their reactivity and affinity to the AChE–OP complex, which reactivity is originated from the nucleophilic activity of the oxime, and affinity is due to physicochemical properties (steric compatibility, electrostatic effects, hydrophobic interactions, *etc.*).^56,57^ RMSD diagrams *versus* time were plotted for the AChE-paraoxon/oxime complex in the presence of different oximes to analyze the stability and affinity of the system. Fig. 10 shows the RMSD of the 2-PAM drug and the AChE-paraoxon enzyme obtained from simulating the AChE-paraoxon/2-PAM system. The results for 2-PAM considerably showed slight position changes, and its RMSD diagram fluctuated around the constant value of 0.03, which can be related to its affinity and interactions with the enzyme.

**Fig. 11:**
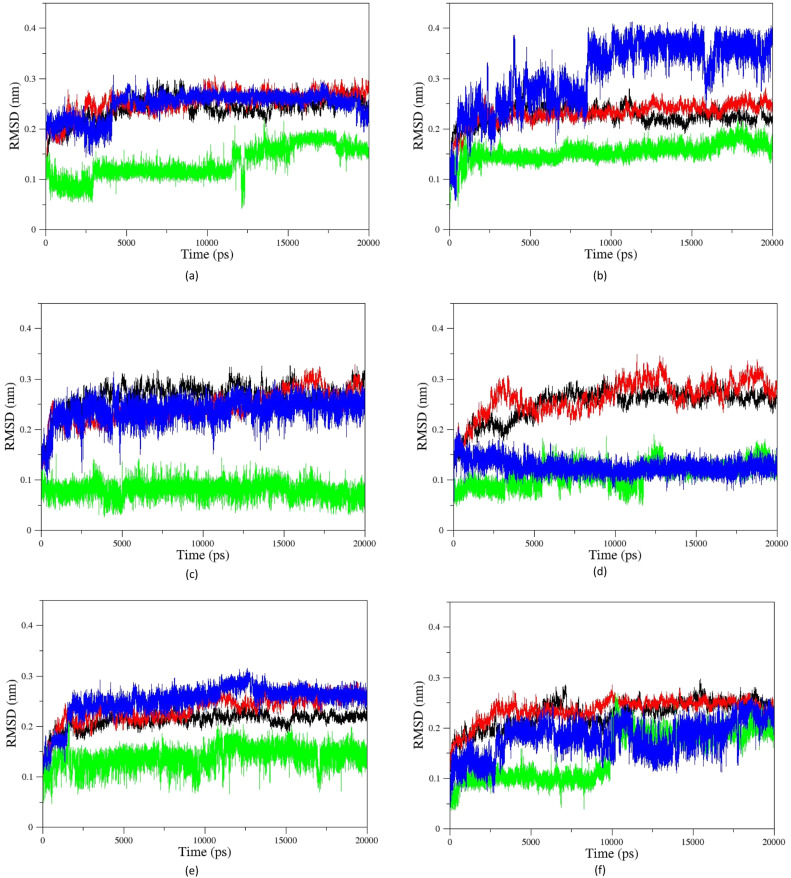
Cα-RMSD *versus* time for AChE-paraoxon complexed with (a) A, (b) B, (c) C, (d) D, (e) E, and (f) F uncharged oximes. The enzyme complexed with *E* isomers (black), *E* isomers of oximes (green), the enzyme complexed with *Z* isomers (red), *Z* isomers of oximes (blue).

Fig. 11 shows the RMSD diagrams for oximes and enzymes in other systems. As illustrated, in all studied systems for uncharged oximes, *E* isomers are more stable than *Z* ones. Oximes and proteins also have an RMSD less than 0.35, except for the oxime BZ that its RMSD fluctuates up to 0.4 and shows considerable position changes during simulation. Due to the larger structure than 2-PAM and more DOFs, uncharged oximes highlighted more significant RMSD variations than 2-PAM during simulation.

Furthermore, the large structure of uncharged oximes increases the volume of the interaction site with the enzyme leading to an increase in the number of water molecules in this site and thereby stabilizing hydrogen bonds between the solvent and oximes. Table 3 shows the average number of hydrogen bonds between the water molecules and oximes, confirming this conclusion. As is clear in this table, the average number of hydrogen bonds between the solvent molecules present in the binding pocket of enzyme and uncharged oximes is larger than its corresponding value with 2-PAM. Table 3 shows the average number of hydrogen bonds between the oximes and the enzyme, and as seen, all uncharged oximes except for AE, AZ, BE, and EZ form more hydrogen bonds with the enzyme than 2-PAM. Consequently, establishing hydrogen bonds with the solvent and the enzyme active site could help to stabilize uncharged oximes. Souza *et al.* have shown oximes with larger structure increase the volume of the enzyme–drug interaction site and thus enhance the presence of water molecules in this region. They also showed that in addition to creating stabilizing interactions between the drug and the appropriate amino acids, the interactions between the drug and solvent molecules can also stabilize the drug at the site of interaction with the protein.^58^Fig. 11f, for example, shows the RMSD of the isomers of oxime F and the enzyme in the respective systems.

**Table 3:** The average number of hydrogen bonds between the enzyme and oximes (first column) and the number of hydrogen bonds between oximes and solvent (second column)

	Protein–oxime ± SD	Oxime–solvent ± SD
2-PAM	1.7357 ± 0.0048	0.0657 ± 0.0019
AE	1.2189 ± 0.0050	0.9585 ± 0.0063
BE	1.3552 ± 0.0041	2.0384 ± 0.0078
CE	1.9328 ± 0.0059	3.4368 ± 0.0085
DE	2.7109 ± 0.0090	1.5440 ± 0.0083
EE	3.6163 ± 0.0080	1.8868 ± 0.0090
FE	2.6001 ± 0.0093	1.8593 ± 0.0091
AZ	1.1991 ± 0.0035	1.8942 ± 0.0083
BZ	1.7717 ± 0.0075	1.4689 ± 0.0082
CZ	1.7454 ± 0.0051	2.6494 ± 0.0092
DZ	1.9965 ± 0.0078	2.0889 ± 0.0086
EZ	1.5020 ± 0.0077	1.5479 ± 0.0089
FZ	3.3306 ± 0.0085	1.1991 ± 0.0063

**Fig. 12:**
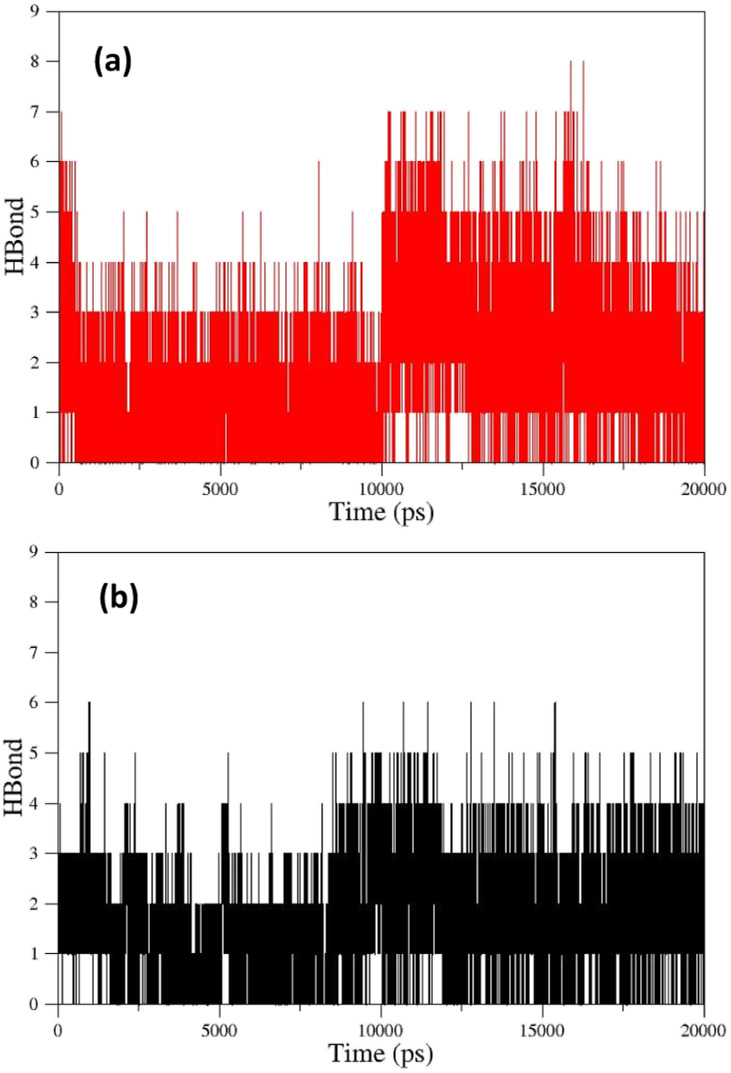
(a) The number of hydrogen bonds formed between oxime FE and solvent molecules in the enzyme active site, (b) the number of hydrogen bonds formed between oxime BZ and AChE-paraoxon enzyme over simulation.

As can be seen, the position changes of the oxime FE within AChE-paraoxon stabilize after 10 ns. This stability may be due to hydrogen bonds between the oxime and solvent molecules so that the average number of hydrogen bonds increases from 4 to 6 after 10 ns (Fig. 12a). Fig. 11b illustrates the RMSD diagrams for the isomers of oxime B and the enzyme. As seen, the RMSD of oxime BZ is stabilized after 9 ns and fluctuates around 0.36, which can be related to an increase in the average number of stable hydrogen bonds between the oxime and enzyme from 3 to 5 after 9 ns (Fig. 12b). The number of hydrogen bonds between the oxime–enzyme and oxime–solvent for all systems has been indicated in ESI File and Fig. S16.† The other systems revealed remarkable stability in the RMSD.

The percentage of hydrogen bond (H-bond) occupation for different oximes during simulation has been reported in ESI File and Table S17.† According to this material, the residues GLU202 and ALA127 have the highest percentage of H-bond occupation during the formation of hydrogen bonds with 2-PAM over simulation. For oxime AE, the aromatic residue TYR133 revealed the highest H-bond occupation percentage.

Moreover, the residues TRP86, ALA127, and SON203 demonstrated a considerable occupation percentage for forming hydrogen bonds with AE. Both TPR86 and SON203 residues belong to the CAS site, and SON203 is considered a key residue in this site. The residue GLU202 has the largest share in occupying H-bond in forming hydrogen bonds between oxime AZ and the enzyme active site. As indicated ESI File and Table S17,† the number of residues in the enzyme active site with the largest contribution in occupying H-bond is larger for uncharged oximes than 2-PAM, and some key residues such as SON203 and HIS447 revealed an H-bond occupation percentage larger than 10%. In addition to these residues in the CAS site, the residues GLU202, GLY121, and TYR337 in the same site and the aromatic residues TYR124 and TYR341 in the PAS site have significant hydrogen interactions with uncharged oximes during simulation, indicating more effective stabilization of uncharged oximes in the enzyme active site and probably higher efficiency in inhibited enzyme reactivation. The residue ALA127 for oximes EE and FE and the residue ASN87 for oxime FZ occupy a large percentage of H-bond in hydrogen interaction with the oxygen atom in the substitute OCH_3_. In addition to the oximic OH and its bonded nitrogen, as well as the triazole ring in the structure of studied uncharged oximes, the presence of NO_2_ and OCH_3_ substitutes in the structure of some oximes plays a key role in hydrogen interactions with residues in the enzyme active site. For instance, the residue ALA127 for oximes CE, CZ, and DE, and for oxime DE, in addition to ALA127, ASN87 have a significant contribution to H-bond occupation in the interaction with oxygen atoms of the substitute NO_2_ of the oxime during simulation. As shown in ESI File and Table S17,† the residues ASN87 for oxime BZ, TYR124 for oximes DE and DZ, TYR337 for oximes EE and FE, and TYR133 for oxime FZ play a significant role in hydrogen bonding with triazole ring nitrogen atoms. In general, the distribution of hydrogen bonds reveals the formation of more stable hydrogen bonds between AChE-paraoxon and uncharged oximes, which may indicate the better performance of these oximes as reactivators of the paraoxon-inhibited AChE enzyme.

**Fig. 13:**
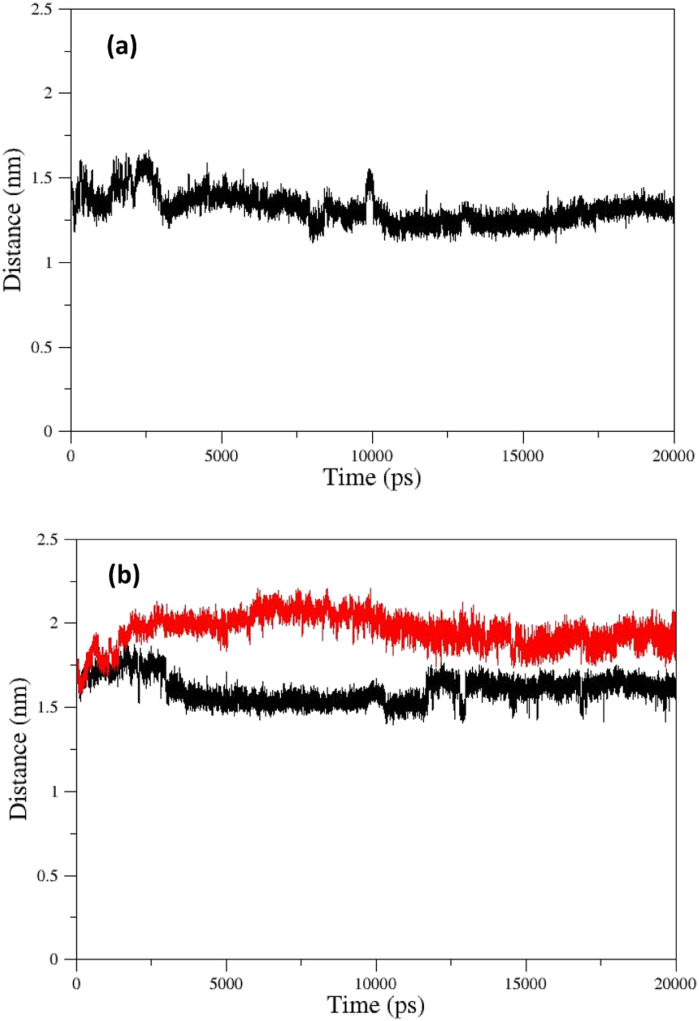
COM distances between paraoxon and oximes during simulation for oximes (a) 2-PAM (b) EE (black) and EZ (red).

The changes in the distance between the center of mass (COM) of paraoxon and oximes were calculated as a function of time for different systems during simulation. As shown in Fig. 13a, oxime 2-PAM is placed closer to the enzyme active site than uncharged oximes over simulation due to the larger structure of uncharged oximes and consequently, steric hindrance to moves toward paraoxon. It can also be related to the stronger interaction of uncharged oximes with the solvent (water) molecules in the AChE binding pocket (Table 3, ESI File and Fig. S16†). Among the distance diagrams, isomers of oxime E show severe fluctuations (Fig. 13b). For EE, the distance downwardly trend from the beginning of the simulation, and the oxime approaches paraoxon so that the distance remains constant after 13 ns and fluctuates around a constant value. Oxime EZ moves away from paraoxon (unlike EE) up to 13 ns and then fluctuates around a constant value.

The change in the COM distance for *E* isomers can be attributed to the formation of a more stable complex with the enzyme by oxime E, which the constant RMSD of *E* isomers (Fig. 11e) after 13 ns confirms this issue. It is hypothesized that placing the oxime at a safe distance from the catalytic site is important for its stability. The distance diagrams for other systems have been displayed in ESI File and Fig. S18.†

### 3.5. Selection and synthesis of the novel reactivator(s)

**Scheme 1:**
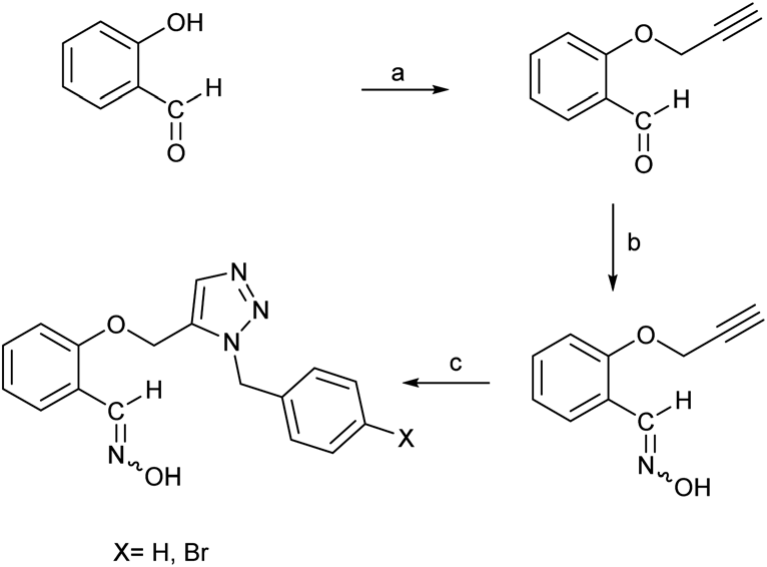
Synthesis of triazole salicylaldoxime derivatives. (a) propargyl bromide, K_2_CO_3_, DMF, RT; (b) NH_2_OH(aq), K_2_CO_3_, ethanol; (c) benzyl or 4-bromobenzyl azide, pentamethylcyclopentadienyl bis(triphenylphosphine)ruthenium(ii) chloride, toluene, 80 °C, 4 h.

According to the quantum chemical studies, compound B showed the lowest activation energy among the studied oximes. In the second state, oxime A exhibited lower activation energy among the studied species. Accordingly, from mechanistic standpoint, oximes B and, A outperformed other oximes. The results of CHelpG charge analyses have been indicate that oximes A, E, and B have the higher nucleophilicity index and probably reactivation effects among the studied molecules. High positive log *P* value of compound B indicates its higher penetrability in the BBB. Finally, according to molecular docking studies and molecular dynamic simulation it can be concluded that oximes A and B outperform other triazole salicylaldoxime derivatives in reactivating the paraoxon-inhibited human AChE enzyme. Therefore, the compounds A and B were selected for synthesizing. Regarding the synthesis, initially *O*-propargyl salicylaldehyde was synthesized from propargyl bromide and salicylaldehyde as starting materials using nucleophilic substitution reaction in DMF (Scheme 1). In the next step, *O*-propargyl salicylaldoxime was prepared by nucleophilic addition of a hydroxylamine (NH_2_OH) to the *O*-propargyl salicylaldehyde in EtOH. Finally, 1,5-triazole salicylaldoxime derivatives were synthesized *via* cycloaddition reaction between *O*-propargyl salicylaldoximes and benzyl azides.^59^ All the synthesized compounds were identified and characterized by elemental analysis (CHN), FT-IR and NMR spectroscopy (ESI File and Fig. S20–S27†).

### 3.6. Determination of reactivation efficacy parameters

Because reactivation is afforded by dissociated oximate anion, the dissociation constant (p*K*_a_) is a crucial element for the removal of the phosphyl moiety. Additionally, the element limiting penetration across biological membranes is appropriate p*K*_a_. For standard reactivators, these values vary from 7.04 for HLö-7 to 8.2 for trimedoxime.^60^ Therefore, p*K*_a_ values for the compounds A and B were determined by spectrophotometry^61^ and compared with 2-PAM (Table 4). The results showed that the p*K*_a_ values for compounds A and B were within the mentioned reactivator p*K*_a_ standard range. The presence of protonation–deprotonation groups in the designed molecules may cause the fact that, despite the neutrality of these compounds, the obtained acidity values are very close to the acidity of 2-PAM (p*K*_a_ = 7.61). The results showed that the two new oximes, A and B, had similar polarity and nucleophilicity to 2-PAM (p*K*_a_ values of 7.52 ± 0.05 and 7.82 ± 0.06, respectively), indicating similar ability to donate an electron pair to AChE.

**Table 4:** p*K*_a_ values for pralidoxime and the compounds A and B

Compound	2-PAM	A	B
p*K*_a_ values	7.61 (ref. 60)	7.52 ± 0.05	7.82 ± 0.06

Following the guidance of the computational studies, two candidate compounds (A and B) were also evaluated by *in vitro* experiments. The ability of recently synthesized substances to reactivate paraoxon-inhibited RBC AChE was assessed using a modified Ellman's assay.^41,42^ The reactivation results of paraoxon-inhibited RBC AChE are listed in Table 5. The results showed that the two new oximes, A and B, had low reactivation percentages (15.4 ± 0.94 and 14.4 ± 0.71, respectively) after 30 min of incubation at 37 °C and pH 7.4, indicating poor reactivation ability of AChE.

**Table 5:** Reactivation of paraoxon-inhibited RBC hAChE by new synthesized oximes (0.1 mM)

Synthesized oximes	Reactivation % ± SD (30 min, 37 °C, pH = 7.4)
A	15.4 ± 0.94
B	14.3 ± 0.71
2-PAM	27.2 ± 0.17

In order to get a deeper comprehension of the reactivation mechanism of the oximes A and B, the reactivation kinetic parameters were calculated. Experimental details to determine the concentration dependence of the apparent reactivation rate *k*_obs_ for the reactivation of paraoxon-inhibited RBC AChE are described in the ESI File, page 205.†

Paraoxon-inhibited RBC AChE was incubated at 37 °C with at least 4 concentrations of compound A and B in phosphate buffer (0.1 M, pH 7.4). At time intervals ranging from 5 to 180 min, the observed first-order rate constant of reactivation (*k*_obs_) at different concentrations of compound A and B ([OX]) were calculated. At each oxime concentration, *k*_obs_ was calculated from the slope of the initial portion of log(100 − % reactivation) *vs.* time of reactivation plot as *k*_obs_ = −2.303 × slope, assuming an approach to 100% reactivation (ESI File and Fig. S29†). Then, *k*_obs_*vs.* [OX] plot was drawn to calculate rate constants (ESI File and Fig. S30†). Since *k*_obs_*vs.* [OX] was linear, the slope corresponded to overall second-order rate constant of reactivation (*k*_r_); in this case, maximum first-order rate constant (*k*_+2_) and the dissociation constant of the phosphorylated enzyme–oxime complex (*K*_ox_) could not be determined. The results were presented in Table 6. For comparison, *k*_r_ values of enzyme reactivation by 2-PAM, obidoxime, HI-6 and HLö-7 are also collected in the Table 6 from Worek *et al.* previous work.^40^ The *k*_r_ values showed that, contrary to the results of the calculations, the designed compounds (A and B) had far less reactivation efficacy than pralidoxime. However, they had low reaction rates with AChE (*k*_r_ values of 0.067 and 0.054 mM^−1^ min^−1^, respectively), indicating slow reactivation kinetics.

**Table 6:** Second-order rate constants *k*_r_ (mM^−1^ min^−1^) for the reactivation of paraoxon-inhibited AChE by oximes

Compound	2-PAM	Obidoxime	HI-6	HLö-7	A	B
*k*_r_ (mM^−1^ min^−1^)	0.908	5.27	0.365	7.11	0.067 ± 0.0093	0.054 ± 0.0024

In summary, we designed and evaluated novel uncharged 1,2,3-triazole salicylaldoxime derivatives for the reactivation of paraoxon-inhibited human AChE. We performed computational studies to predict the reactivity and BBB permeability of designed compounds and compared them with 2-PAM, a standard pyridinium aldoxime. We then selected and synthesized two compounds A and B based on the computational results. We modelled the reaction between the oximes and the AChE-paraoxon complex, which is formed when paraoxon binds covalently to the serine residue at the active site of AChE. Several parameters were calculated to compare the reactivity of A, B and 2-PAM, such as hardness (*η*), chemical potentials (*μ*), *ω*^−^ values, CHelpG charges and p*K*_a_ values. The compounds A and B have lower hardness (*η*) and higher chemical potentials (*μ*) than 2-PAM, indicating that they are more reactive and less stable than 2-PAM. They also have higher *ω*^−^ values, which measure the nucleophilicity of the oximes, and higher CHelpG charges on the oxygen atom of the oxime group, which reflect the electron-donating ability of the oximes. These properties suggest that A and B are more nucleophilic and more electron-rich than 2-PAM, which could facilitate their attack on the phosphorus atom of the inhibited enzyme. However, A and B also have similar p*K*_a_ values as 2-PAM, which means that they have similar acid–base properties. However, previous studies have shown that uncharged oximes with lower p*K*_a_ values are more effective than those with higher p*K*_a_ values in reactivating OP-inhibited AChE.^8^ This is because lower p*K*_a_ values imply higher concentrations of the anionic form of the oxime, which is more nucleophilic and more reactive than the neutral form.

One of the main goals of developing uncharged oximes is to improve their ability to cross the blood–brain barrier (BBB) and reach the CNS structures where OP-inhibited AChE is located. The presence of a permanent cationic charge in pyridinium oximes makes them unable to cross the BBB efficiently. Therefore, uncharged oximes appear as an interesting alternative to overcome this problem. We performed theoretical calculations to estimate the BBB permeability of A and B, and compared them with 2-PAM. We calculated some parameters that are related to BBB permeability, such as log *P* (octanol–water partition coefficient), MW (molecular weight) and HBD (number of hydrogen bond donors). They have slightly higher lipophilicity, but also higher molecular weight and similar hydrogen bonding potential and polarity. We determined the reactivation rates (*k*_r_) of A and B by measuring the recovery of AChE activity after incubation with paraoxon and the oximes at different concentrations. As can be seen from Table 5, A and B have lower reactivation percentages than 2-PAM, indicating that they are less effective in reversing the inhibition of human AChE by paraoxon. As can be seen from Table 6, A and B have lower *k*_r_ values than 2-PAM and other pyridinium oximes, indicating that they are less efficient in restoring the activity of paraoxon-inhibited human AChE. This is consistent with previous studies that reported low reactivation rates for uncharged oximes. Our results suggest that A and B have some advantages and disadvantages in terms of reactivity and BBB permeability compared to 2-PAM. They have higher nucleophilicity and electron-donating ability, but also similar p*K*_a_ values. They have slightly higher lipophilicity, but also higher molecular weight and similar hydrogen bonding potential and polarity. They have lower reactivation rates and percentages than 2-PAM *in vitro*. Therefore, A and B could be optimized by modifying their structure to enhance their reactivity and improve their BBB permeability.

## 4. Conclusions

We have designed, synthesized and evaluated two new uncharged oximes, A and B, for the reactivation of human AChE inhibited by paraoxon, a toxic OP compound. We have used computational methods to estimate the nucleophilicity, electron-donating ability, lipophilicity, hydrogen bonding potential and polarity of these oximes, and compared them with 2-PAM, a widely used charged oxime. We have also measured the *in vitro* reactivation rate and percentage of these oximes for paraoxon-inhibited AChE. Our results show that A and B have higher reactivity and BBB permeability than 2-PAM, but they also have similar acidity and lower reactivation efficiency. Therefore, we suggest that A and B can be further optimized by modifying their structure to increase their reactivity and BBB permeability, which could lead to the development of more effective uncharged oximes for the treatment of OP poisoning.

## Author contributions

Dr M. H. Baghersad: conceptualization, methodology, provision of study materials, reagents, materials, laboratory samples, instrumentation, writing original draft, visualization, supervision and project administration. Dr A. Habibi: validation, writing – review & editing, visualization. A. Dehdashtinejad: formal analysis, investigation, provision of study materials, reagents, materials, laboratory samples, instrumentation, computing resources, or other analysis tools. All authors reviewed the manuscript.

## Conflicts of interest

The authors declare no conflict of interest.

## Supplementary Material

RA-013-D3RA05658A-s001
