# Supplementary material for: Novel uncharged triazole salicylaldoxime derivatives as potential acetylcholinesterase reactivators: comprehensive computational study, synthesis and in vitro evaluation
Source: RSC Adv. 2023 Sep 28;13(41):28527–41. doi: 10.1039/d3ra05658a (PMC10534079; doi:10.1039/d3ra05658a)

Electronic Supplementary Material (ESI) for RSC Advances.
This journal is © The Royal Society of Chemistry 2023

# Supplementary Information

## Novel uncharged triazole salicylaldoxime derivatives as potential acetylcholinesterase reactivators: comprehensive computational study, synthesis and in vitro evaluation

Mohammad Hadi Baghersad*, [1], Azizollah Habibi [2] and Arash dehdashti nejad [1]

[1] Applied Biotechnology Research Center, Baqiyatallah University of Medical Sciences, Tehran, Iran.
[2] Faculty of Chemistry, Kharazmi University, No. 43, P. Code 15719-14911, Mofatteh Street, Enghelab Ave., Tehran, Iran.

**Corresponding author: Hadibaghersad@bmsu.ac.ir**

# Table of content

**S1.** The energy of optimized structures ....... 3
**S2.** The binding energy, O(Oxime)-P(POX) distance ....... 4
**S3.** The energy diagram of the optimized structures ....... 5
**S4.** The optimized structures of species involved in reactivating SON by AZ ....... 7
**S5.** The optimized structures of species involved in reactivating SON by BE ....... 8
**S6.** The optimized structures of species involved in reactivating SON by BZ ....... 9
**S7.** The optimized structures of species involved in reactivating SON by CE ....... 10
**S8.** The optimized structures of species involved in reactivating SON by CZ ....... 11
**S9.** The optimized structures species involved in reactivating SON by DE ....... 12
**S10.** The optimized structures of species involved in reactivating SON by DZ. ....... 13
**S11.** The optimized structures of species involved in reactivating SON by EE ....... 14
**S12.** The optimized structures of species involved in reactivating SON by EZ ....... 15
**S13.** The optimized structures of species involved in reactivating SON by FE ....... 16
**S14.** The optimized structures of species involved in reactivating SON by FZ ....... 17
**S15.** The 2D and 3D structures of interactions ....... 18
**S16.** The number of hydrogen bonds ....... 22
**S17.** The percentage of hydrogen bond occupation ....... 26
**S18.** COM distances between POX and oximes ....... 27
**S19.** M062X/6-31G* calculated cartesian coordinates of all optimized structures. ....... 28
**Spectra** ....... 199
FT-IR spectra ....... 199
**S20.** O-propargylated salicylaldehyde ....... 199
**S21.** O-propargylated salicylaldoxime ....... 199
**S22.** 2-((1-benzyl-1H-1,2,3-triazol-5-yl)methoxy)benzaldehyde oxime (A) ....... 200
**S23.** 2-((1-(4-bromobenzyl)-1H-1,2,3-triazol-5-yl)methoxy)benzaldehyde oxime (B) ....... 200
NMR spectra ....... 201
**S24.** O-propargylated salicylaldehyde ....... 201
**S25.** O-propargylated salicylaldoxime ....... 202
**S26.** 2-((1-benzyl-1H-1,2,3-triazol-5-yl)methoxy)benzaldehyde oxime (A) ....... 203
**S27.** 2-((1-(4-bromobenzyl)-1H-1,2,3-triazol-5-yl)methoxy)benzaldehyde oxime (B) ....... 204
**Determination of reactivation parameters** ....... 205
**S28.** Plot of reactivation *vs* time for compounds A and B. ....... 205
**S29.** Plot of log(100-react.%) *vs* time for compounds A and B ....... 205
**S30.** Plot of $K_{obs}$ *vs* concentration for compounds A and B ....... 205

**Computational results**

## S1. The energy of optimized structures

The energy of optimized structures at the theoretical M062X/6-31G* level in the aqueous phase for species involved in reactivating the serine-POX (SON) molecule through studied oximes.

| | | | | | | | |
|---|---|---|---|---|---|---|---|
| | | | | | | | |
| **AE** | -643759.3811 | **AZ** | -643754.0200 | **BE** | -2257202.653 | **BZ** | -2257196.561 |
| **C1**$_{AE}$ | -1443810.037 | **C1**$_{AZ}$ | -1443805.742 | **C1**$_{BE}$ | -3057256.034 | **C1**$_{BZ}$ | -3057248.093 |
| **TS1**$_{AE}$ | -1443806.077 | **TS1**$_{AZ}$ | -1443801.349 | **TS1**$_{BE}$ | -3057252.829 | **TS1**$_{BZ}$ | -3057243.803 |
| **IN**$_{AE}$ | -1443816.003 | **IN**$_{AZ}$ | -1443814.083 | **IN**$_{BE}$ | -3057260.494 | **IN**$_{BZ}$ | -3057256.495 |
| **TS2**$_{AE}$ | -1443813.897 | **TS2**$_{AZ}$ | -1443813.223 | **TS2**$_{BE}$ | -3057259.996 | **TS2**$_{BZ}$ | -3057255.784 |
| **C2**$_{AE}$ | -1443827.563 | **C2**$_{AZ}$ | -1443827.052 | **C2**$_{BE}$ | -3057274.219 | **C2**$_{BZ}$ | -3057269.650 |
| | | | | | | | |
| **CE** | -772044.2415 | **CZ** | -772041.4792 | **DE** | -2385487.366 | **DZ** | -2385483.919 |
| **C1**$_{CE}$ | -1572094.643 | **C1**$_{CZ}$ | -1572093.530 | **C1**$_{DE}$ | -3185541.799 | **C1**$_{DZ}$ | -3185535.760 |
| **TS1**$_{CE}$ | -1572090.140 | **TS1**$_{CZ}$ | -1572088.274 | **TS1**$_{DE}$ | -3185536.851 | **TS1**$_{DZ}$ | -3185530.508 |
| **IN**$_{CE}$ | -1572098.230 | **IN**$_{CZ}$ | -1572098.705 | **IN**$_{DE}$ | -3185542.723 | **IN**$_{DZ}$ | -3185540.989 |
| **TS2**$_{CE}$ | -1572096.186 | **TS2**$_{CZ}$ | -1572097.513 | **TS2**$_{DE}$ | -3185541.611 | **TS2**$_{DZ}$ | -3185539.730 |
| **C2**$_{CE}$ | -1572108.009 | **C2**$_{CZ}$ | -1572109.482 | **C2**$_{DE}$ | -3185554.430 | **C2**$_{DZ}$ | -3185551.797 |
| | | | | | | | |
| **EE** | -715595.9676 | **EZ** | -715590.5874 | **FE** | -2329039.422 | **FZ** | -2329033.122 |
| **C1**$_{EE}$ | -1515646.708 | **C1**$_{EZ}$ | -1515642.618 | **C1**$_{FE}$ | -3129093.845 | **C1**$_{FZ}$ | -3129085.130 |
| **TS1**$_{EE}$ | -1515642.617 | **TS1**$_{EZ}$ | -1515638.143 | **TS1**$_{FE}$ | -3129089.042 | **TS1**$_{FZ}$ | -3129080.523 |
| **IN**$_{EE}$ | -1515652.479 | **IN**$_{EZ}$ | -1515650.293 | **IN**$_{FE}$ | -3129096.894 | **IN**$_{FZ}$ | -3129092.755 |
| **TS2**$_{EE}$ | -1515650.441 | **TS2**$_{EZ}$ | -1515649.408 | **TS2**$_{FE}$ | -3129096.452 | **TS2**$_{FZ}$ | -3129091.881 |
| **C2**$_{EE}$ | -1515663.604 | **C2**$_{EZ}$ | -1515662.609 | **C2**$_{FE}$ | -3129110.287 | **C2**$_{FZ}$ | -3129105.037 |
| | | | | | | | |
| **2-PAM** | -286146.9395 | | | | | | |
| **C1**$_{(2-PAM)}$ | -1086193.381 | | | | | | |
| **TS1**$_{(2-PAM)}$ | -1086186.711 | | | | | | |
| **IN**$_{(2-PAM)}$ | -1086188.872 | | | | | | |
| **TS2**$_{(2-PAM)}$ | -1086185.549 | | | | | | |
| **C2**$_{(2-PAM)}$ | -1086196.157 | | | | | | |
| **C1**$_{(2-PAM)}$ | -1086193.381 | | | | | | |
| | | | | | | | |
| **AChE-POX** | -800041.2208 | | | | | | |

## S2. The binding energy, O(Oxime)-P(POX) distance

The binding energy, O(Oxime)-P(POX) distance, and amino acids interacting with oximes in the AChE-POX complex based on docking calculations.

| | **Energy (kcal/mol)** | **Distance O-P (nm)** | **Interaction Residues** |
|---|---|---|---|
| **2-PAM** | -4.96 | 0.73 | TRP86, TRP117, TYR119, GLY120, GLY121, TYR133, GLU202, SON203, TYR337, HIS447, GLU448, ILE451 |
| **AE** | -6.72 | 0.37 | ASP74, TRP86, GLY120, GLY121, TYR124, SER125, GLY126, LEU130, TYR133, GLU202, SON203, ALA204, TYR337, TYR341, HIS447, GLY448, TYR449 |
| **BE** | -8.16 | 0.38 | ASP74, TRP86, GLY120, GLY121, TYR124, SER125, GLY126, LEU130, TYR133, GLU202, SON203, VAL294, PHE295, PHE297, TYR337, PHE338, TYR341 |
| **CE** | -7.35 | 0.37 | GLN71, TYR72, VAL73, ASP74, TRP86, ASN87, PRO88, TYR119, GLY120, GLY121, TYR124, SER125, GLY126, ALA127, LEU130, TYR133, GLU202, SON203, TYR337, TYR341, HIS447 |
| **DE** | -6.04 | 0.36 | GLN71, TYR72, VAL73, ASP74, TRP86, ASN87, PRO88, TYR119, GLY120, GLY121, SER125, GLY126, ALA127, LEU130, TYR133, GLU202, SON203, TYR337, PHE338, TYR341 |
| **EE** | -7.59 | 0.49 | TYR72, VAL73, ASP74, TRP86, ASN87, TYR119, GLY120, GLY121, TYR124, SER125, GLY126, ALA127, LEU130, TYR133, GLU202, SON203, TYR337, TYR341, HIS447, GLU202, SON203, TYR337, TYR341, HIS447, GLY448 |
| **FE** | -7.14 | 0.35 | GLN71, TYR72, VAL73, ASP74, THR83, TRP86, ASN87, PRO88, TYR119, GLY120, GLY121, TYR124, SER125, GLY126, ALA127, LEU130, TYR133, GLU202, SON203, TYR337, TYR341 |
| **AZ** | -6.94 | 0.33 | ASP74, TRP86, GLY120, GLY121, GLY122, TYR124, SER125, GLY126, LEU130, TYR133, GLU202, SON203, TYR341, HIS447, GLY448 |
| **BZ** | -7.16 | 0.45 | TYR72, VAL73, ASP74, THR75, LEU76, TRP86, ASN87, PRO88, GLY120, GLY121, TYR124, SER125, GLY126, ALA127, LEU130, TYR133, SON203, TYR341 |
| **CZ** | -6.78 | 0.36 | TYR72, VAL73, ASP74, TRP86, ASN87, PRO88, TYR119, GLY120, GLY121, GLY122, TYR124, SER125, GLY126, ALA127, LEU130, TYR133, SON203, TYR337, TYR341, HIS447 |
| **DZ** | -6.83 | 0.47 | ASP74, TRP86, TYR119, GLY120, GLY121, TYR124, SER125, GLY126, ALA127, LEU130, TYR133, SON203, PHE295, PHE297, TYR337, PHE338, TYR341 |
| **EZ** | -6.05 | 0.52 | ASP74, TRP86, TRP117, GLY120, GLY121, TYR124, SER125, TYR133, GLU202, SON203, TYR337, TYR341, HIS447, GLY448, TYR449, ILE451 |
| **FZ** | -6.87 | 0.55 | TYR72, VAL73, ASP74, TRP86, ASN87, TYR119, GLY120, GLY121, TYR124, SER125, GLY126, ALA127, LEU130, TYR133, GLU202, SON203, TYR337, HIS447, GLY448, TYR449 |

## S3. The energy diagram of the optimized structures

The energy diagram of the optimized structures at the theoretical M062X/6-31G* level for reactivating AChE-POX by (a) AE, (b) AZ, (c) BE, (d) BZ, (e) CE, (f) CZ, (g) DE, (h) DZ, (i) EE, (j) EZ, (k) FE, and (l) FZ in the aqueous phase.

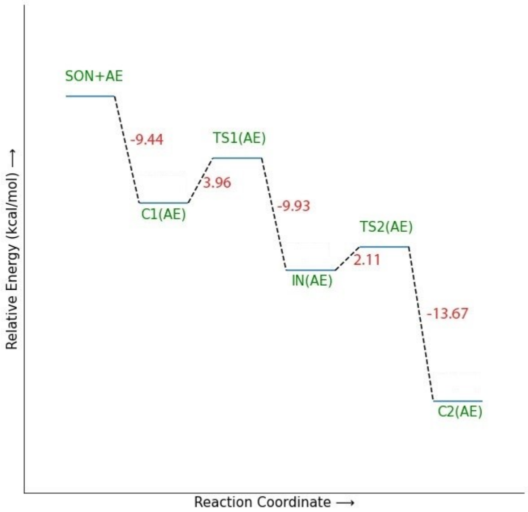


**(a)**

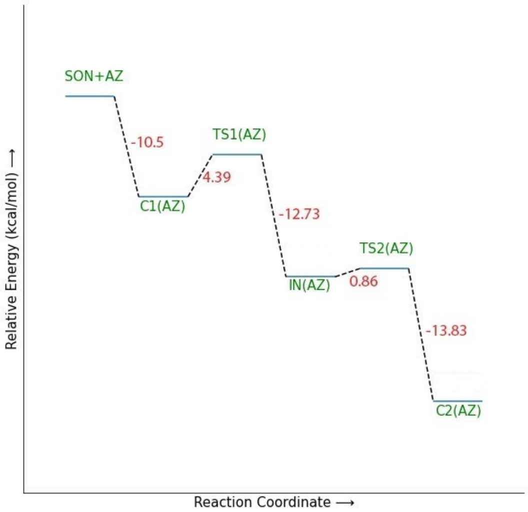


**(b)**

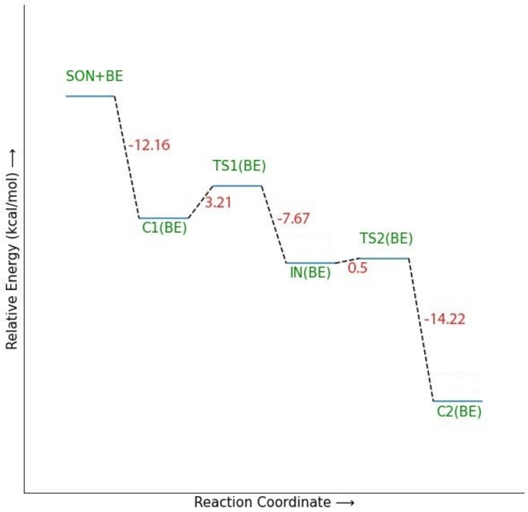


**(c)**

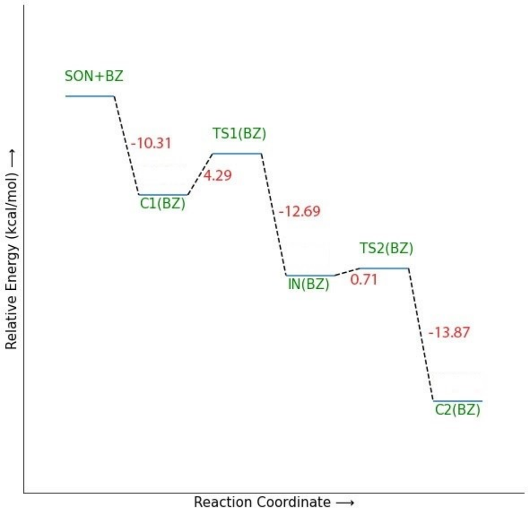


**(d)**

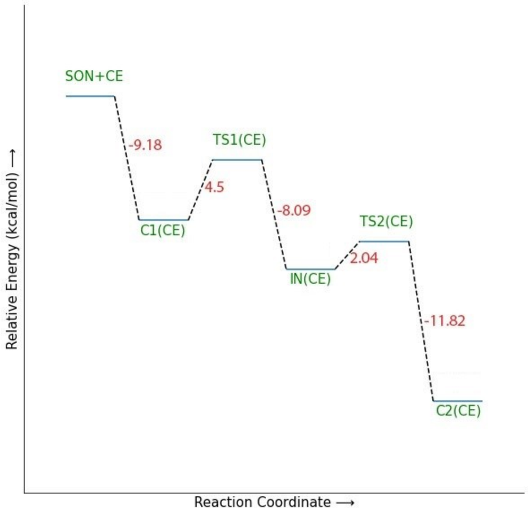


**(e)**

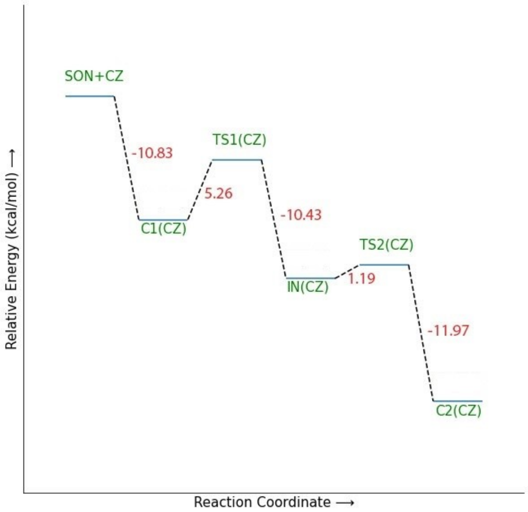


**(f)**

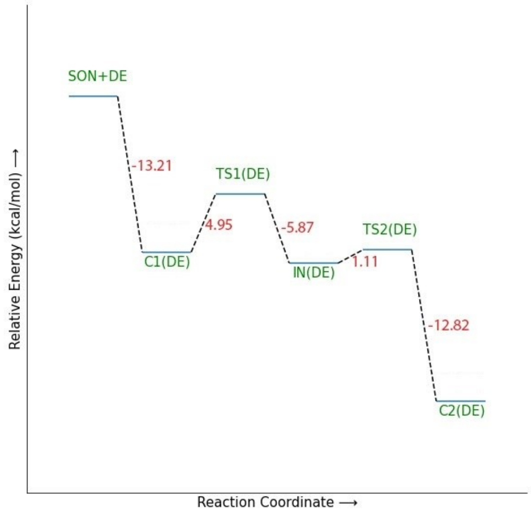
SON+DE
-13.21
TS1(DE)
C1(DE)
4.95
-5.87
TS2(DE)
IN(DE)
1.11
-12.82
C2(DE)
Relative Energy (kcal/mol) ⟶
Reaction Coordinate ⟶

**(g)**

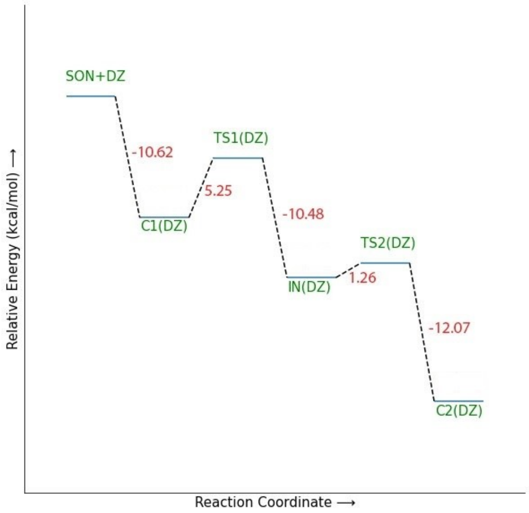
SON+DZ
-10.62
TS1(DZ)
5.25
C1(DZ)
-10.48
TS2(DZ)
IN(DZ)
1.26
-12.07
C2(DZ)
Relative Energy (kcal/mol) ⟶
Reaction Coordinate ⟶

**(h)**

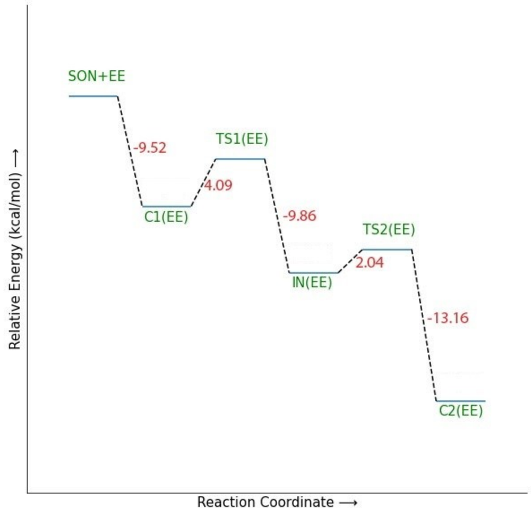
SON+EE
-9.52
TS1(EE)
4.09
C1(EE)
-9.86
TS2(EE)
2.04
IN(EE)
-13.16
C2(EE)
Relative Energy (kcal/mol) ⟶
Reaction Coordinate ⟶

**(i)**

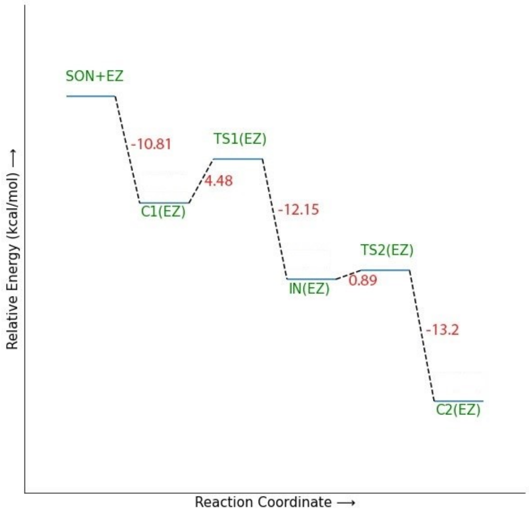
SON+EZ
-10.81
TS1(EZ)
4.48
C1(EZ)
-12.15
TS2(EZ)
IN(EZ)
0.89
-13.2
C2(EZ)
Relative Energy (kcal/mol) ⟶
Reaction Coordinate ⟶

**(j)**

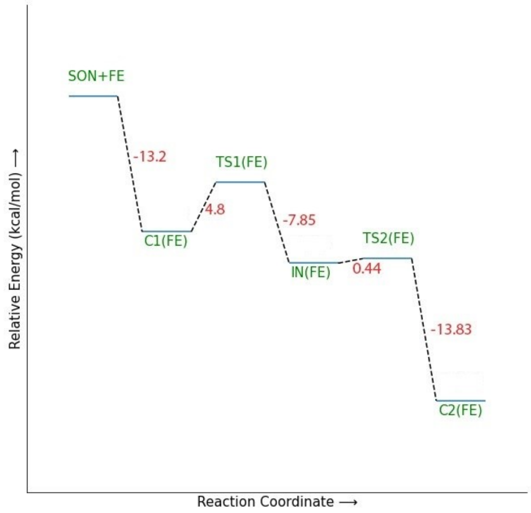
SON+FE
-13.2
TS1(FE)
4.8
C1(FE)
-7.85
TS2(FE)
IN(FE)
0.44
-13.83
C2(FE)
Relative Energy (kcal/mol) ⟶
Reaction Coordinate ⟶

**(k)**

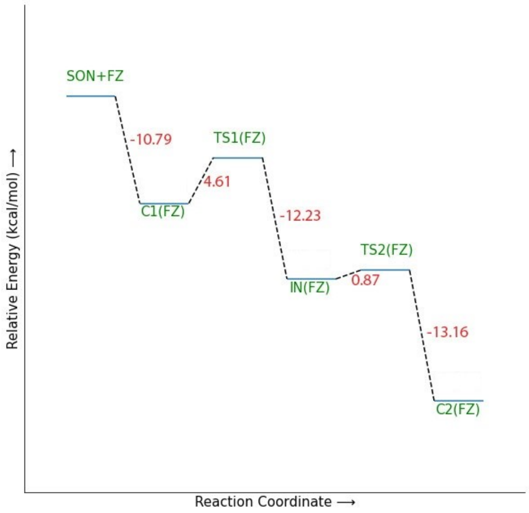
SON+FZ
-10.79
TS1(FZ)
4.61
C1(FZ)
-12.23
TS2(FZ)
IN(FZ)
0.87
-13.16
C2(FZ)
Relative Energy (kcal/mol) ⟶
Reaction Coordinate ⟶

**(l)**

## S4. The optimized structures of species involved in reactivating SON by AZ

The optimized structures at the theoretical M062X/6-31G* level and the selected bond lengths (Å) of species involved in reactivating SON by AZ in the aqueous phase.

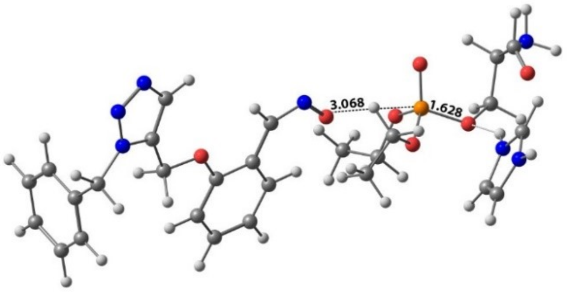


**C1(AZ)**

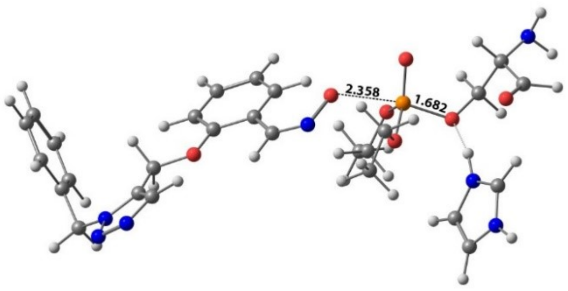


**TS1(AZ)**

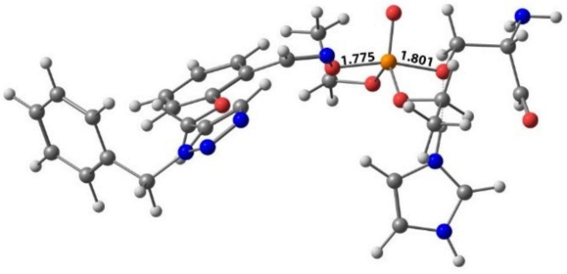


**IN(AZ)**

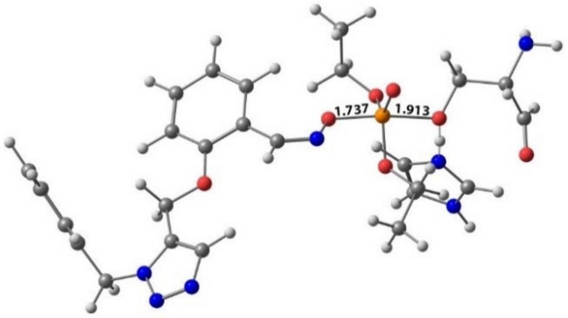


**TS2(AZ)**

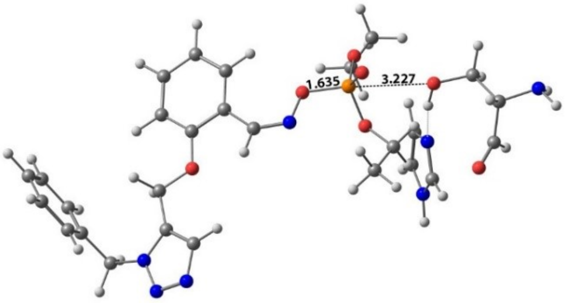


**C2(AZ)**

## S5. The optimized structures of species involved in reactivating SON by BE

The optimized structures at the theoretical M062X/6-31G* level and the selected bond lengths (Å) of species involved in reactivating SON by BE in the aqueous phase.

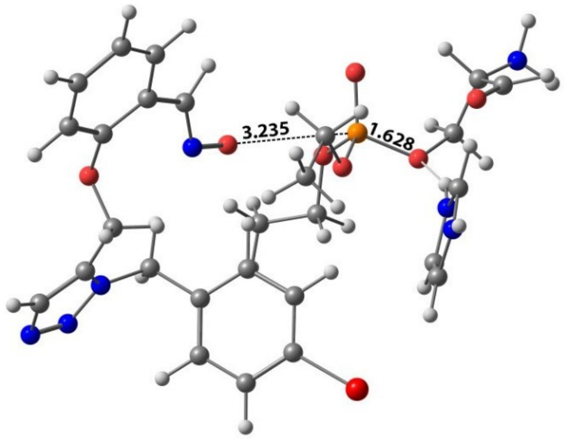


C1(BE)

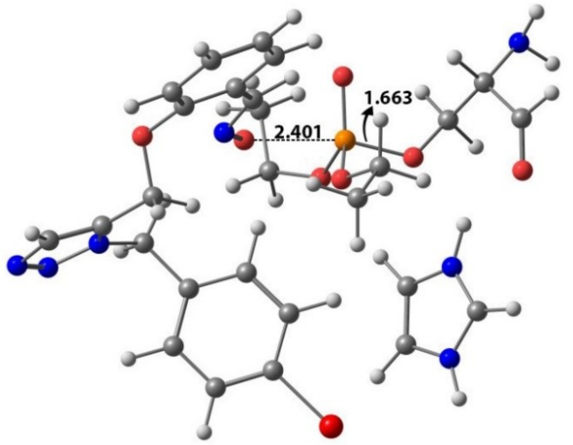


TS1(BE)

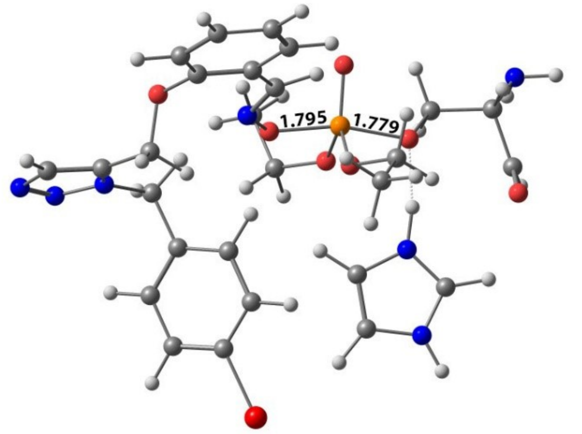


IN(BE)

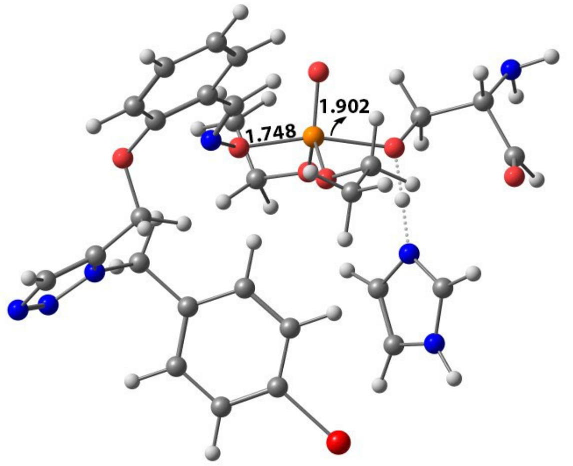


TS2(BE)

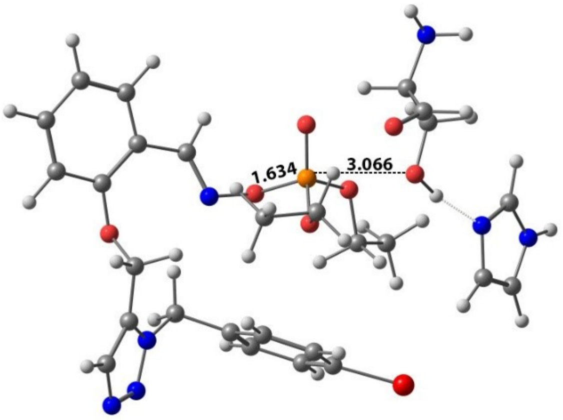


C2(BE)

## **S6.** The optimized structures of species involved in reactivating SON by BZ

The optimized structures at the theoretical M062X/6-31G* level and the selected bond lengths (Å) of species involved in reactivating SON by BZ in the aqueous phase.

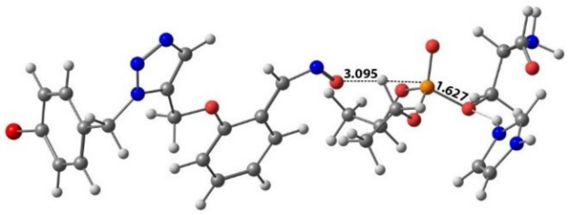


**C1(BZ)**

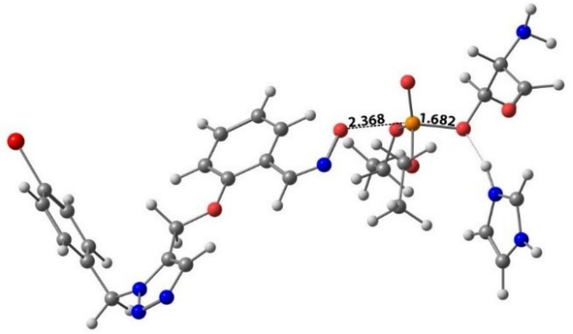


**TS1(BZ)**

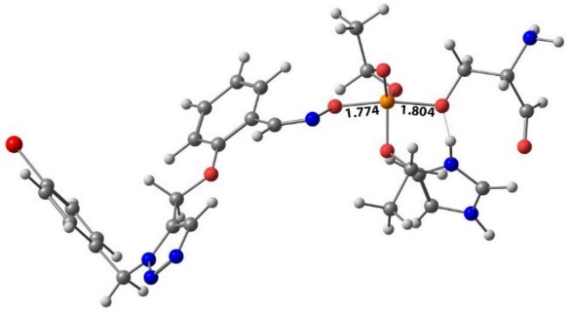


**IN(BZ)**

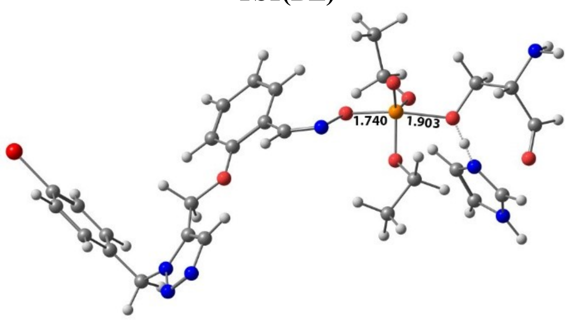


**TS2(BZ)**

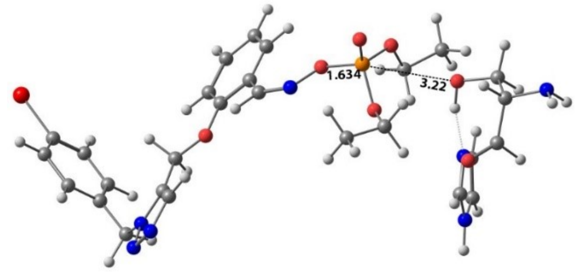


**C2(BZ)**

## **S7.** The optimized structures of species involved in reactivating SON by CE

The optimized structures at the theoretical M062X/6-31G* level and the selected bond lengths (Å) of species involved in reactivating SON by CE in the aqueous phase.

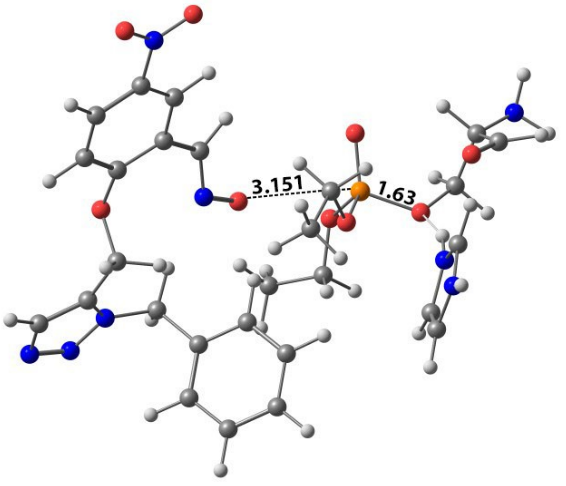


**C1(CE)**

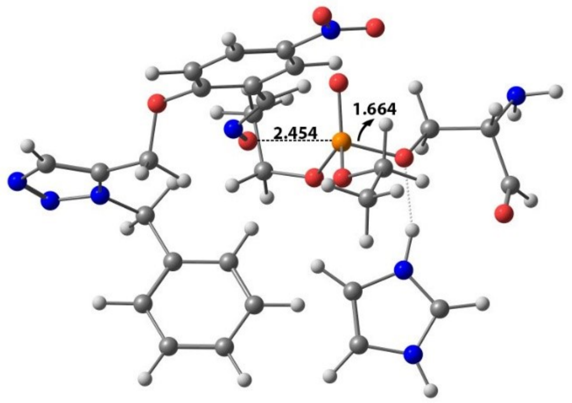


**TS1(CE)**

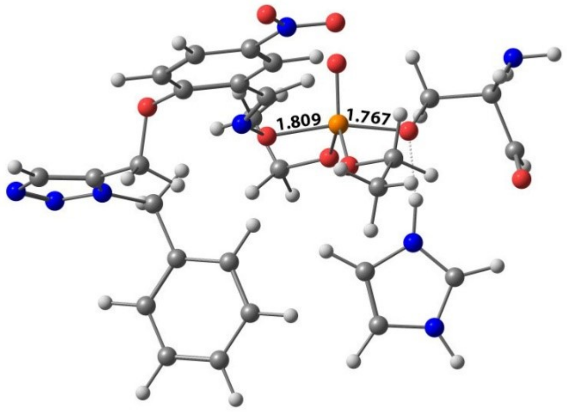


**IN(CE)**

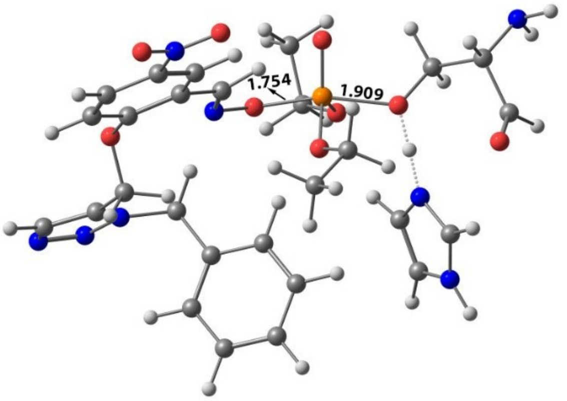


**TS2(CE)**

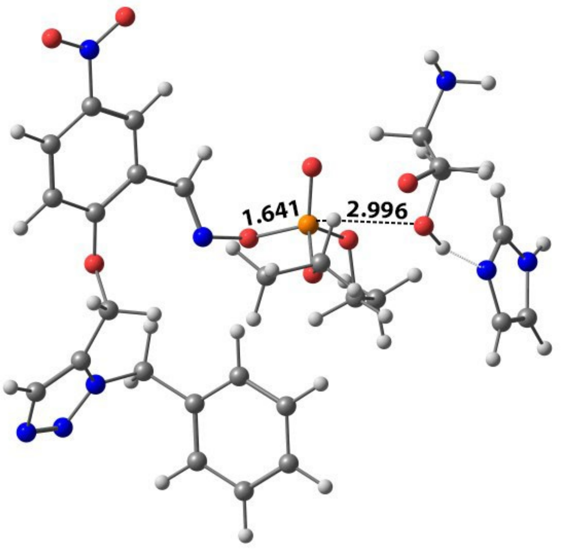


**C2(CE)**

## **S8.** The optimized structures of species involved in reactivating SON by CZ

The optimized structures at the theoretical M062X/6-31G* level and the selected bond lengths (Å) of species involved in reactivating SON by CZ in the aqueous phase.

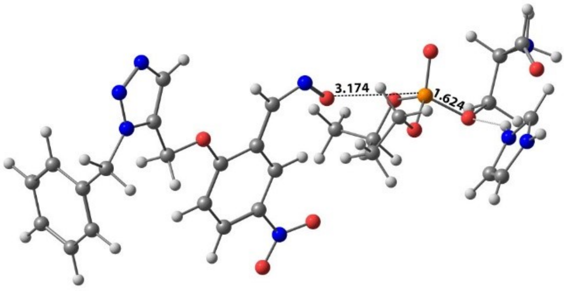


**C1(CZ)**

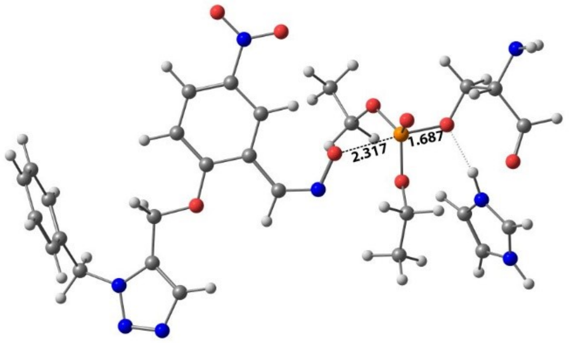


**TS1(CZ)**

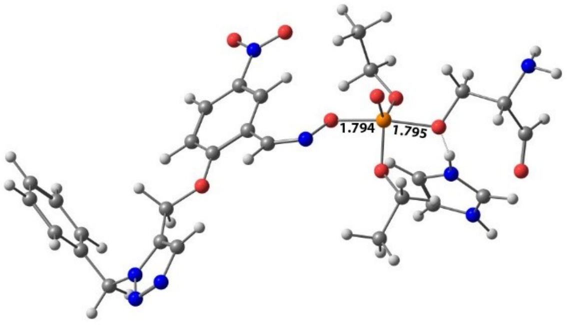


**IN(CZ)**

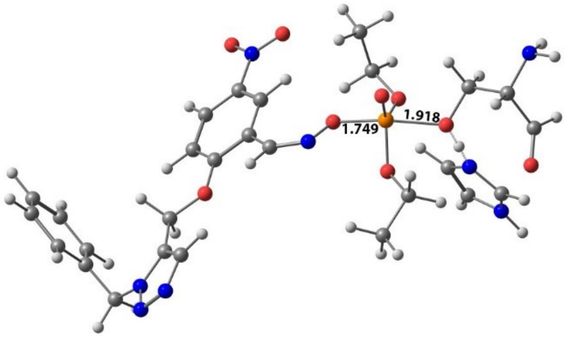


**TS2(CZ)**

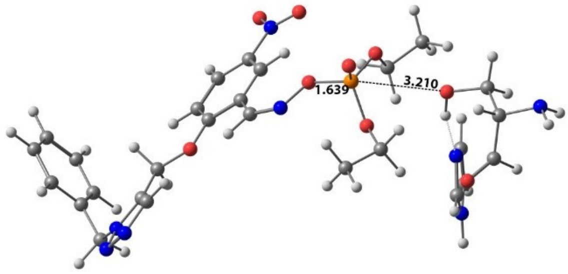


**C2(CZ)**

## **S9.** The optimized structures species involved in reactivating SON by DE

The optimized structures at the theoretical M062X/6-31G* level and the selected bond lengths (Å) of species involved in reactivating SON by DE in the aqueous phase.

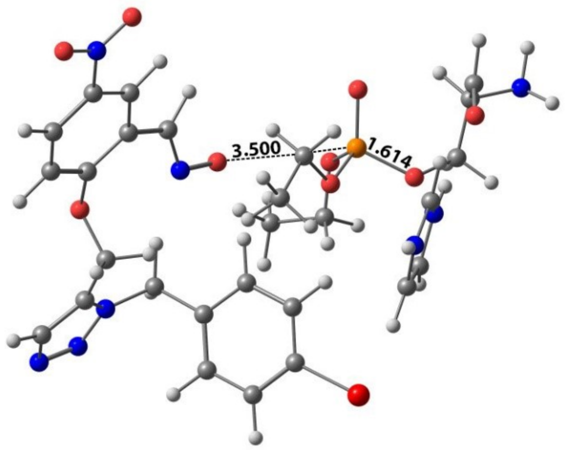


**C1(DE)**

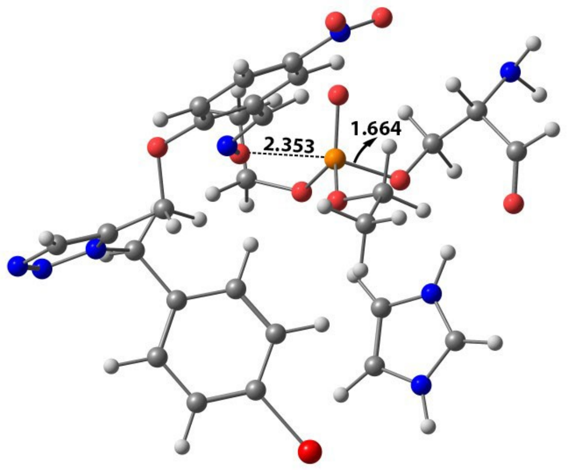


**TS1(DE)**

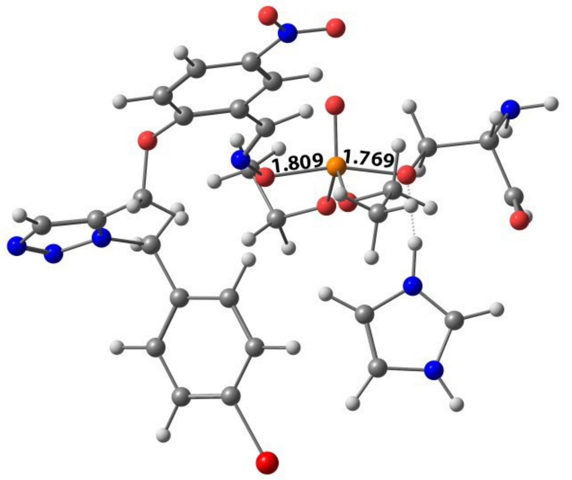


**IN(DE)**

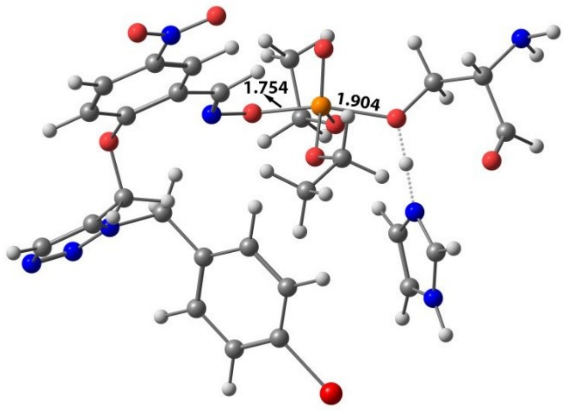


**TS2(DE)**

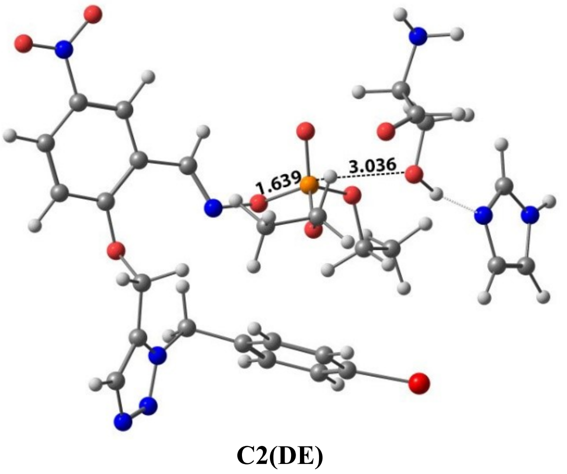


**C2(DE)**

## S10. The optimized structures of species involved in reactivating SON by DZ.

The optimized structures at the theoretical M062X/6-31G* level and the selected bond lengths (Å) of species involved in reactivating SON by DZ in the aqueous phase.

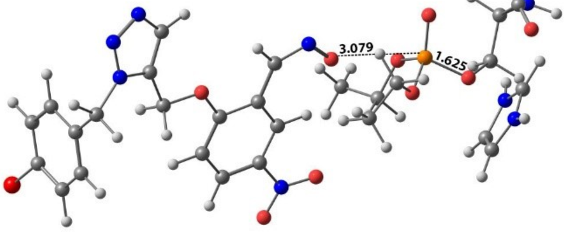


**C1(DZ)**

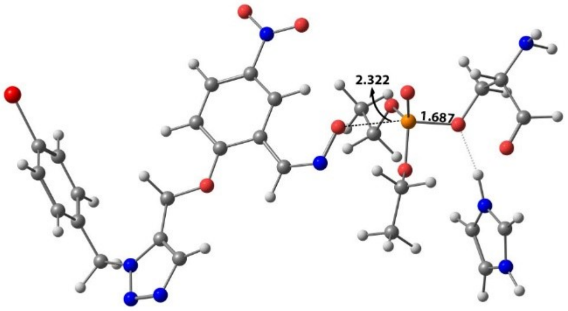


**TS1(DZ)**

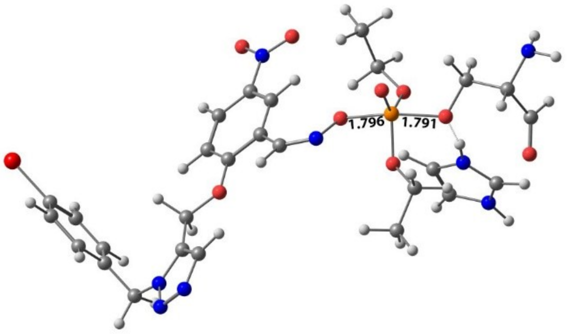


**IN(DZ)**

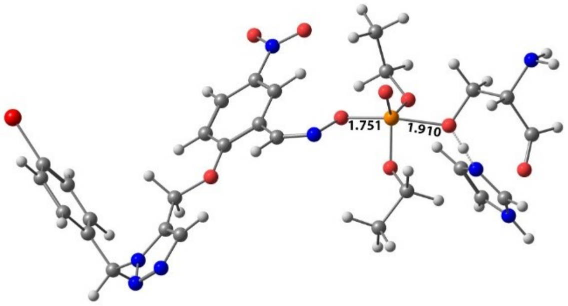


**TS2(DZ)**

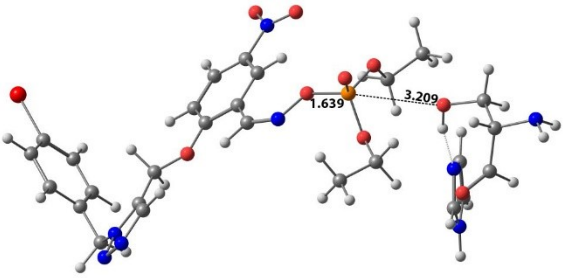


**C2(DZ)**

## **S11.** The optimized structures of species involved in reactivating SON by EE

The optimized structures at the theoretical M062X/6-31G* level and the selected bond lengths (Å) of species involved in reactivating SON by EE in the aqueous phase.

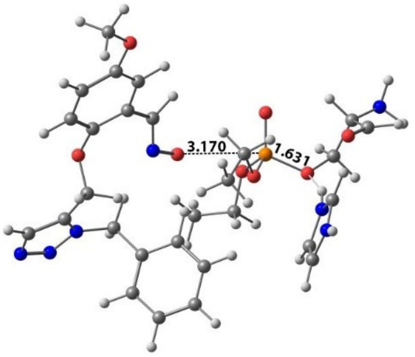


**C1(EE)**

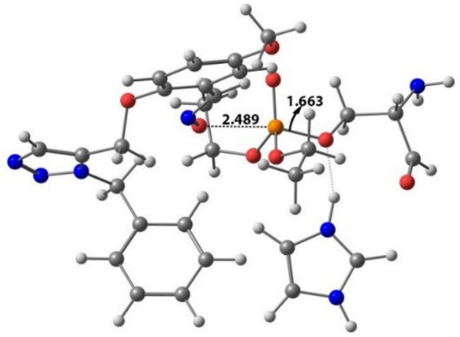


**TS1(EE)**

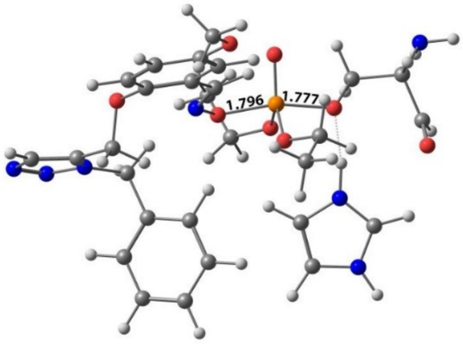


**IN(EE)**

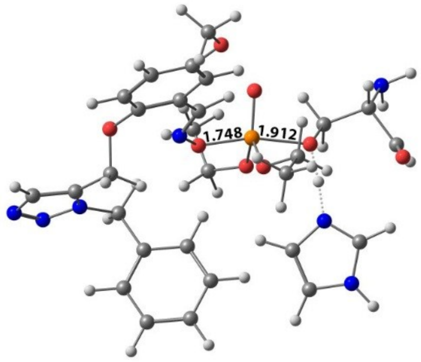


**TS2(EE)**

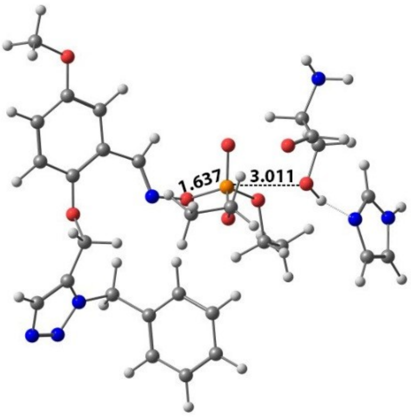


**C2(EE)**

## **S12.** The optimized structures of species involved in reactivating SON by EZ

The optimized structures at the theoretical M062X/6-31G* level and the selected bond lengths (Å) of species involved in reactivating SON by EZ in the aqueous phase.

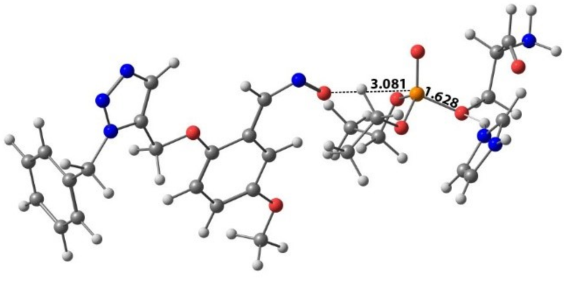


C1(EZ)

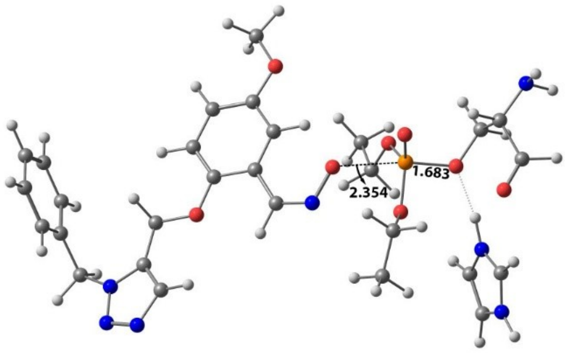


TS1(EZ)

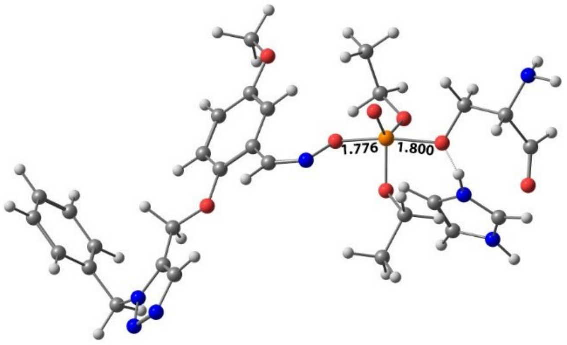


IN(EZ)

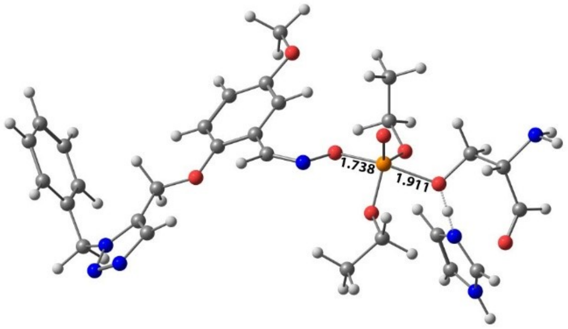


TS2(EZ)

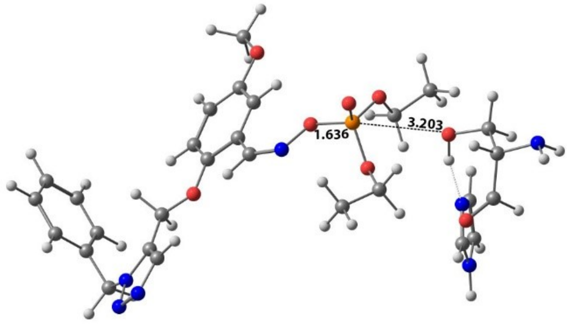


C2(EZ)

## **S13.** The optimized structures of species involved in reactivating SON by FE

The optimized structures at the theoretical M062X/6-31G* level and the selected bond lengths (Å) of species involved in reactivating SON by FE in the aqueous phase.

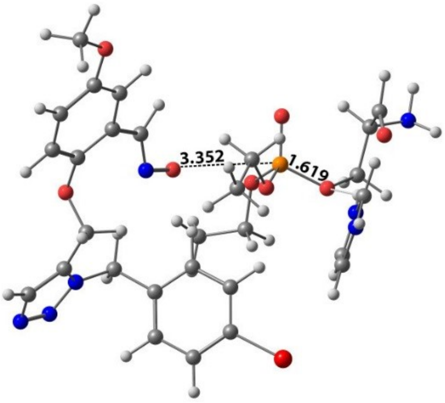


**C1(FE)**

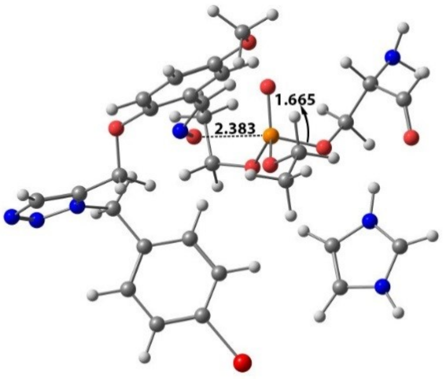


**TS1(FE)**

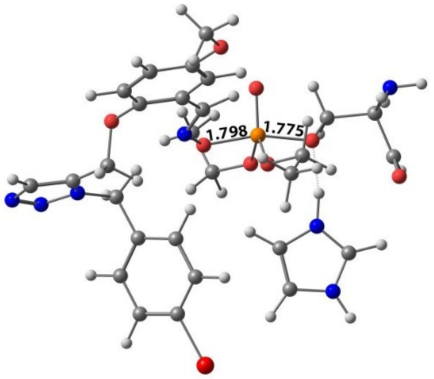


**IN(FE)**

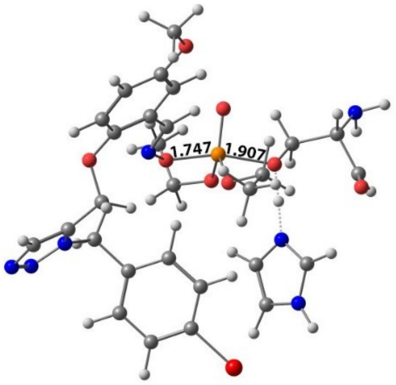


**TS2(FE)**

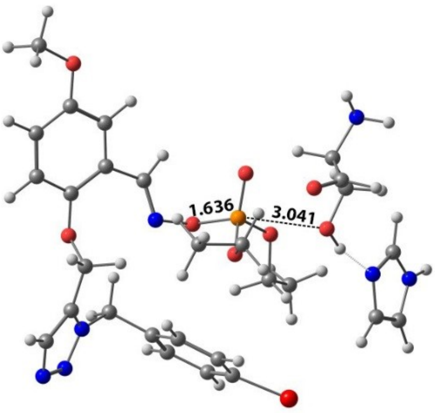


**C2(FE)**

## **S14.** The optimized structures of species involved in reactivating SON by FZ

The optimized structures at the theoretical M062X/6-31G* level and the selected bond lengths (Å) of species involved in reactivating SON by FZ in the aqueous phase.

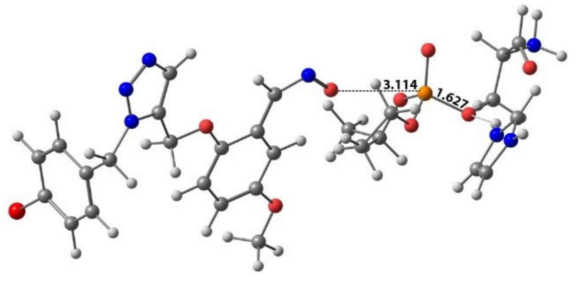


**C1(FZ)**

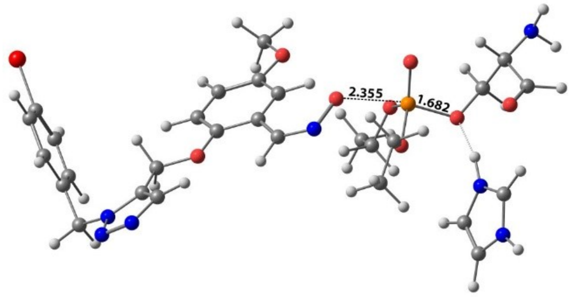


**TS1(FZ)**

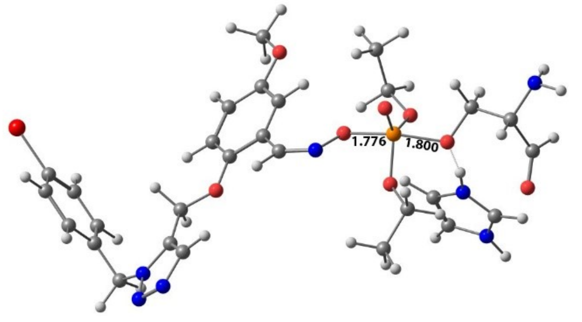


**IN(FZ)**

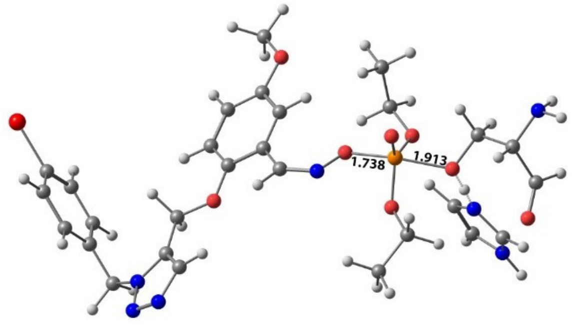


**TS2(FZ)**

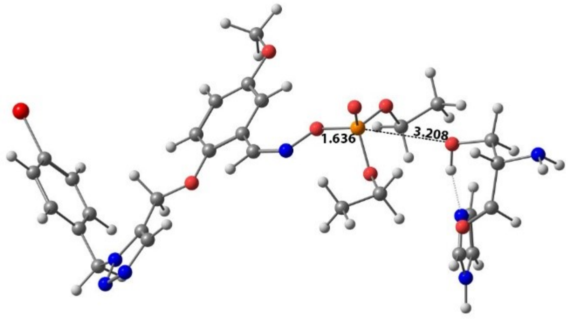


**C2(FZ)**

## **S15.** The 2D and 3D structures of interactions

The 2D and 3D structures of interactions between the amino acids of the active site of enzyme AChE-POX and (a) AZ, (b) BE, (c) BZ, (d) CE, (e) CZ, (f) DE, (g) DZ, (h) EE, (i) EZ, (j) FE, and (k) FZ oxime.

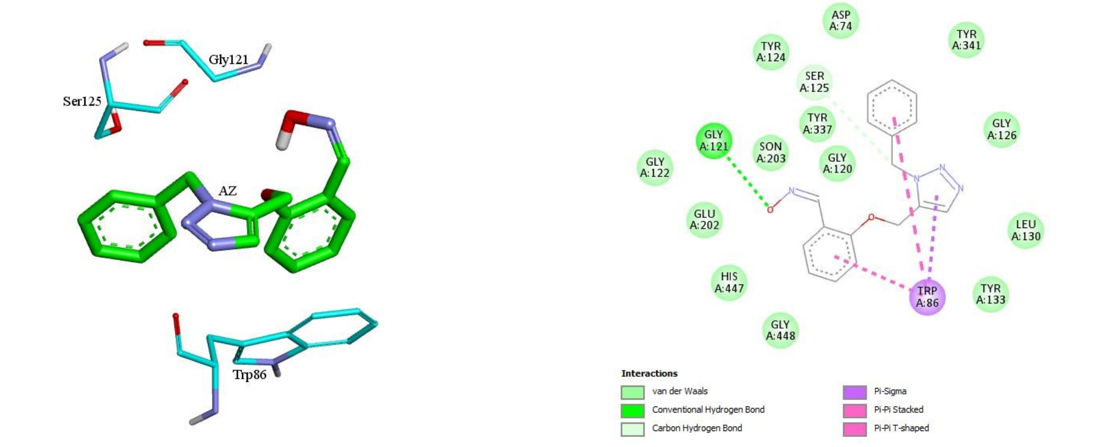


**(a)**

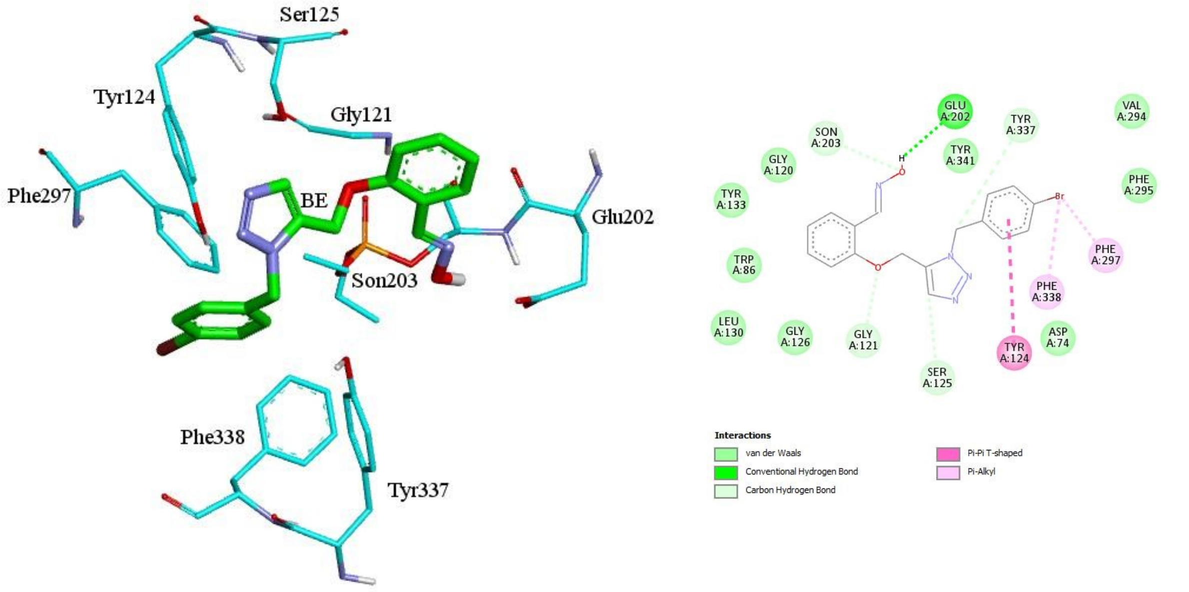


**(b)**

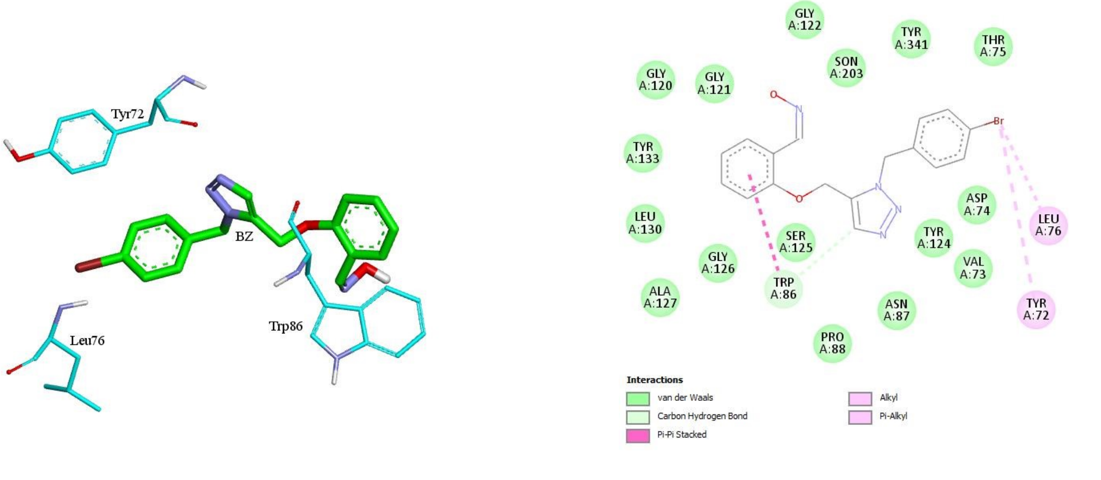

(c)

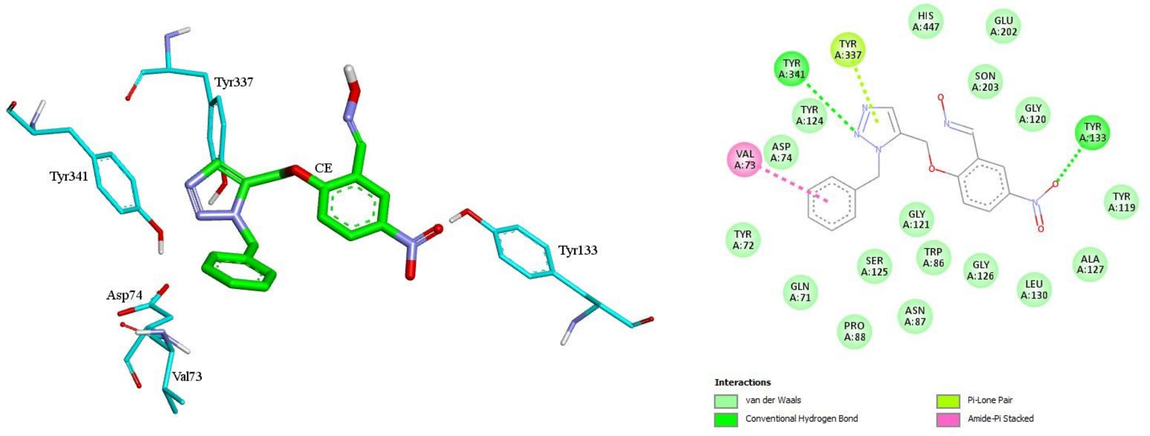

Tyr337
Tyr341
CE
Tyr133
Asp74
Val73
Interactions
van der Waals
Conventional Hydrogen Bond
Pi-Lone Pair
Amide-Pi Stacked


(d)

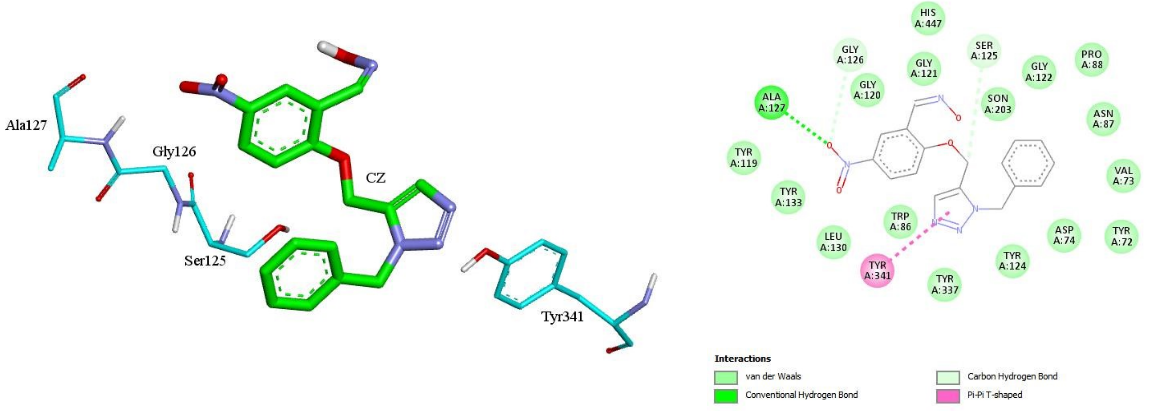

Ala127
Gly126
Ser125
CZ
Tyr341
Interactions
van der Waals
Conventional Hydrogen Bond
Carbon Hydrogen Bond
Pi-Pi T-shaped


(e)

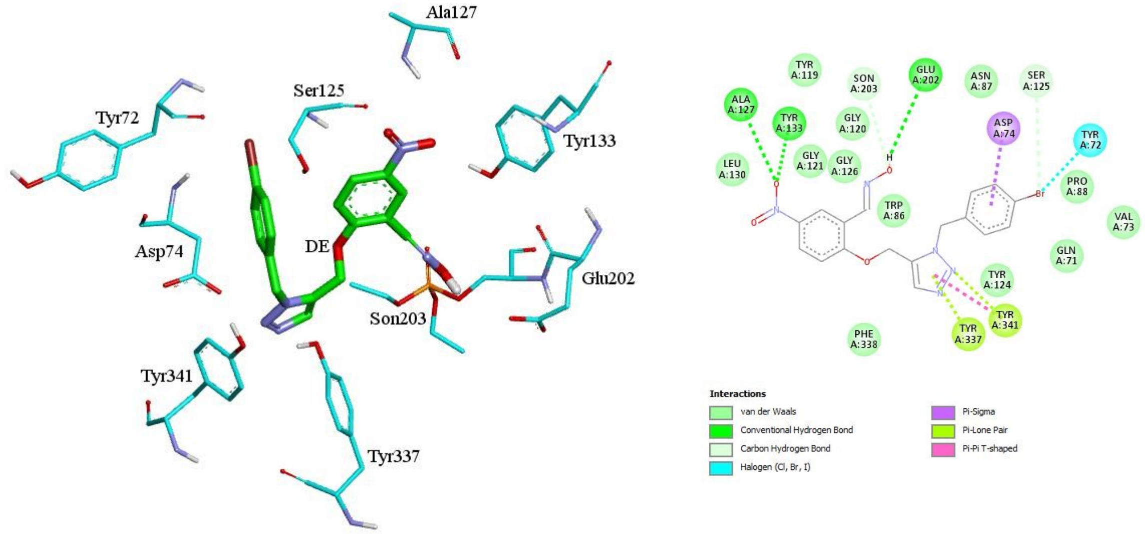

Ala127
Ser125
Tyr72
Tyr133
Asp74
DE
Glu202
Son203
Tyr341
Tyr337
Interactions
van der Waals
Conventional Hydrogen Bond
Carbon Hydrogen Bond
Halogen (Cl, Br, I)
Pi-Sigma
Pi-Lone Pair
Pi-Pi T-shaped


(f)

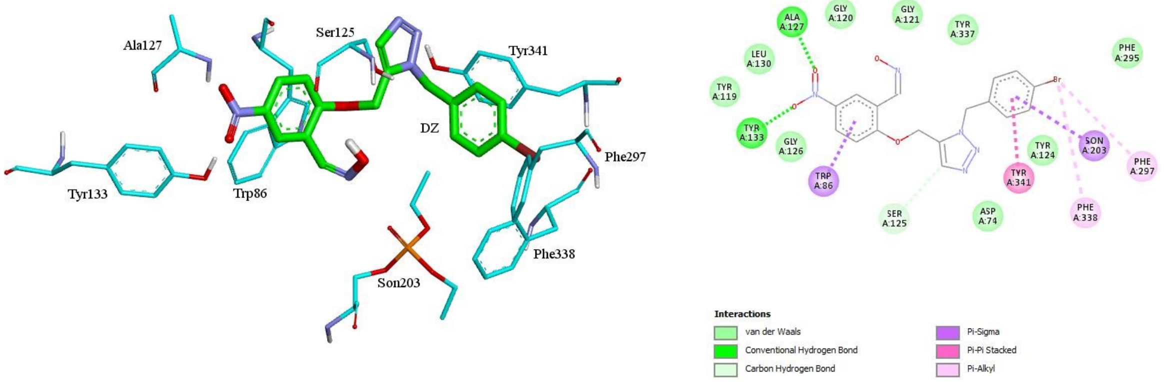
Ala127
Ser125
Tyr341
DZ
Tyr133
Trp86
Phe297
Phe338
Son203
Interactions
van der Waals
Conventional Hydrogen Bond
Carbon Hydrogen Bond
Pi-Sigma
Pi-Pi Stacked
Pi-Alkyl

**(g)**

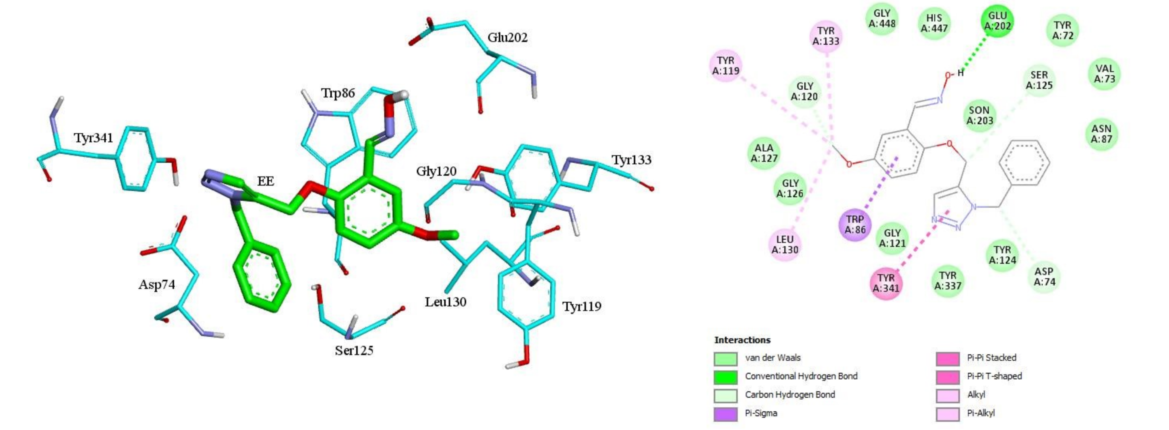
Glu202
Trp86
Tyr341
EE
Gly120
Tyr133
Asp74
Leu130
Tyr119
Ser125
Interactions
van der Waals
Conventional Hydrogen Bond
Carbon Hydrogen Bond
Pi-Sigma
Pi-Pi Stacked
Pi-Pi T-shaped
Alkyl
Pi-Alkyl

**(h)**

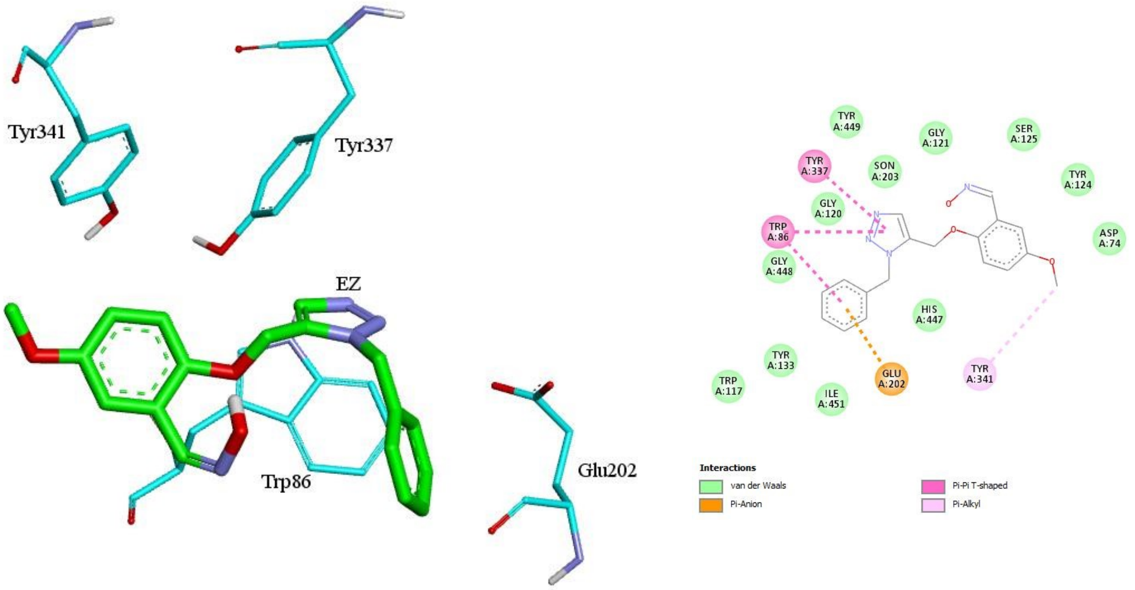
Tyr341
Tyr337
EZ
Trp86
Glu202
Interactions
van der Waals
Pi-Anion
Pi-Pi T-shaped
Pi-Alkyl

**(i)**

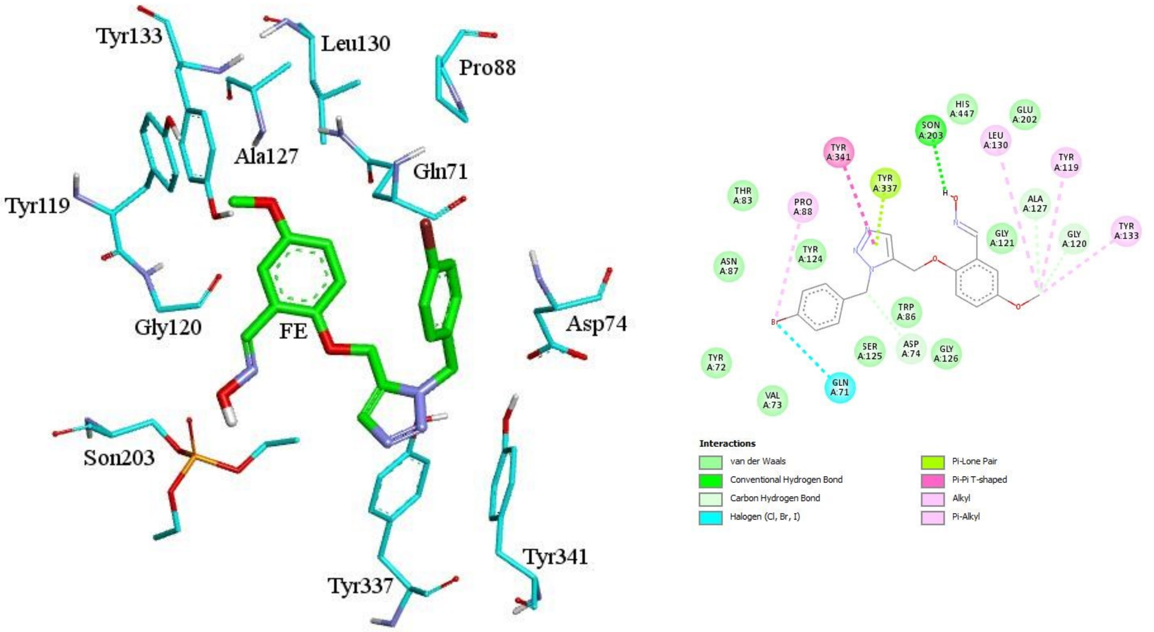
Tyr133
Leu130
Pro88
Ala127
Gln71
Tyr119
FE
Gly120
Asp74
Son203
Tyr337
Tyr341
Interactions
van der Waals
Conventional Hydrogen Bond
Carbon Hydrogen Bond
Halogen (Cl, Br, I)
Pi-Lone Pair
Pi-Pi T-shaped
Alkyl
Pi-Alkyl

**(j)**

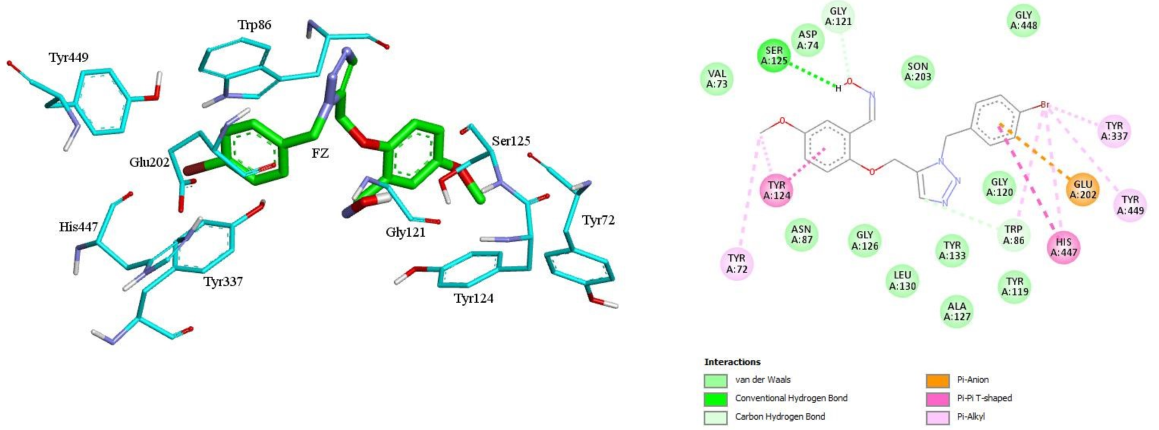
Trp86
Tyr449
FZ
Glu202
Ser125
His447
Gly121
Tyr72
Tyr337
Tyr124
Interactions
van der Waals
Conventional Hydrogen Bond
Carbon Hydrogen Bond
Pi-Anion
Pi-Pi T-shaped
Pi-Alkyl

**(k)**

## **S16.** The number of hydrogen bonds

The number of hydrogen bonds formed between AChE-POX and oxime (black) and the number of hydrogen bonds formed between oxime and solvent (red) during simulation for oximes (a) 2-PAM, (b) AE, (c) AZ, (d) BE, (e) BZ, (f) CE, (g) CZ, (h) DE, (i) DZ, (j) EE, (k) EZ, (l) FE, and (m) FZ.

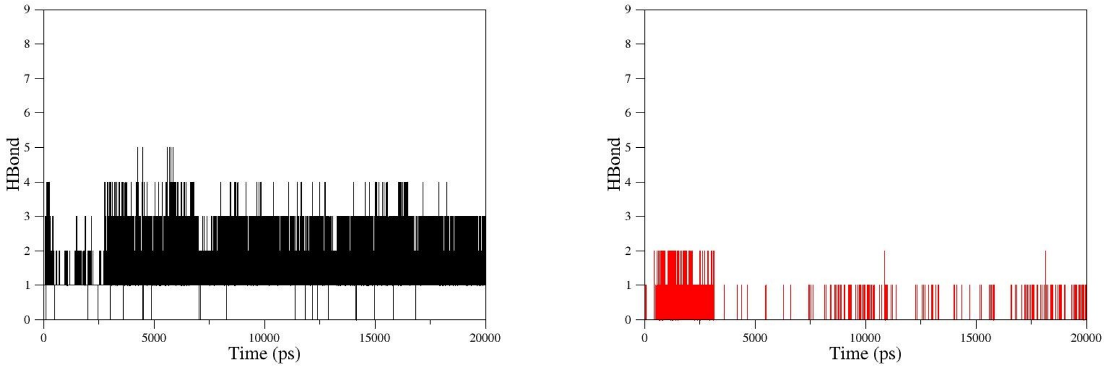


**(a)**

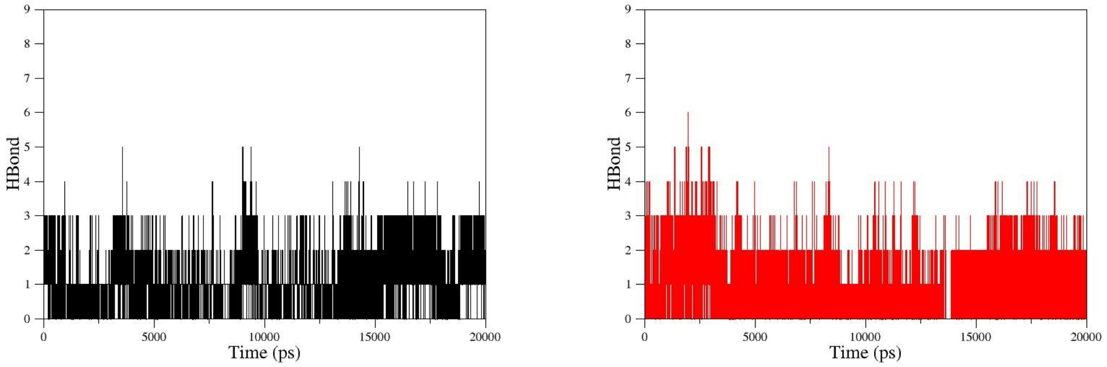


**(b)**

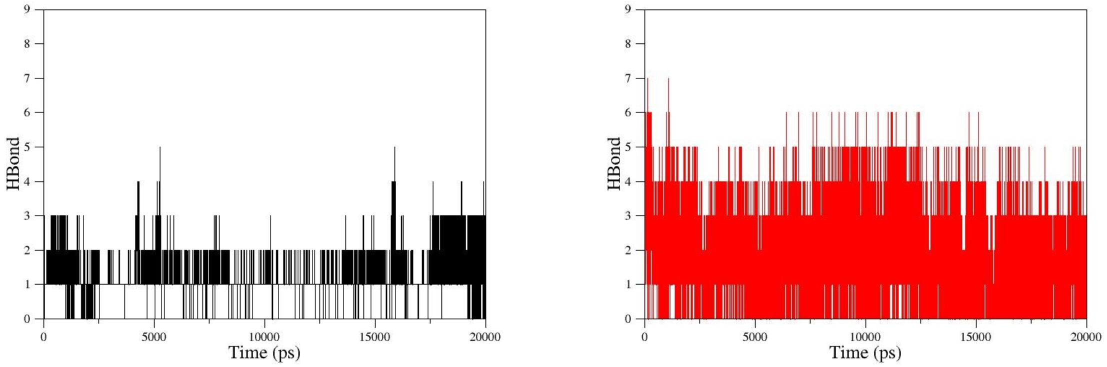


**(c)**

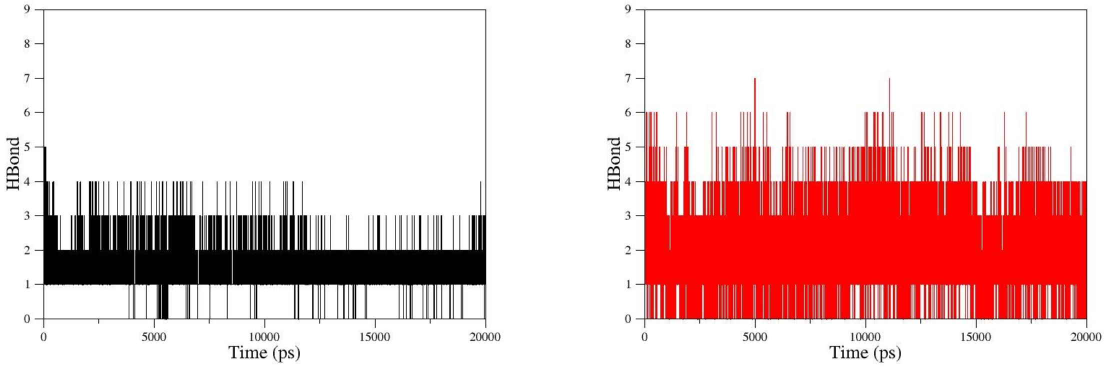
HBond
Time (ps)
HBond
Time (ps)

**(d)**

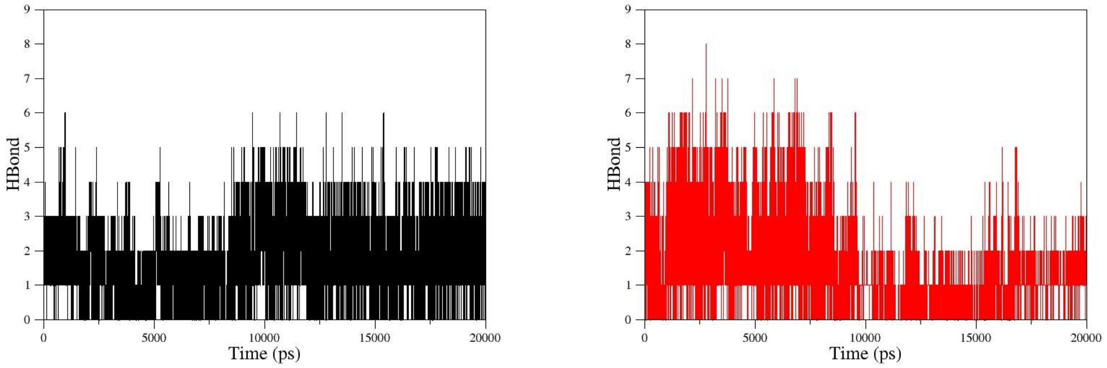
HBond
Time (ps)
HBond
Time (ps)

**(e)**

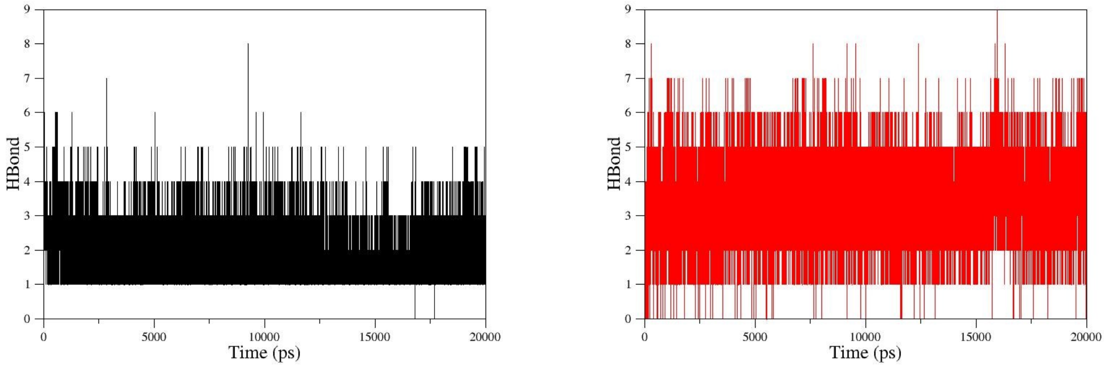
HBond
Time (ps)
HBond
Time (ps)

**(f)**

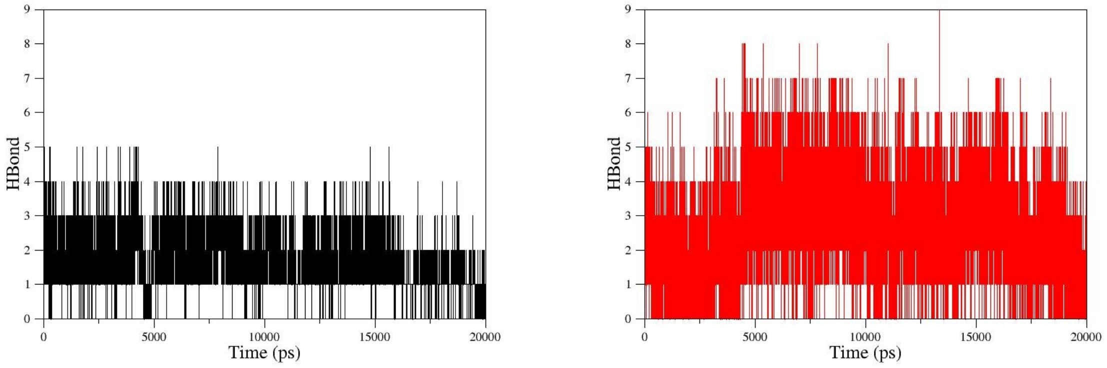
HBond
Time (ps)
HBond
Time (ps)

**(g)**

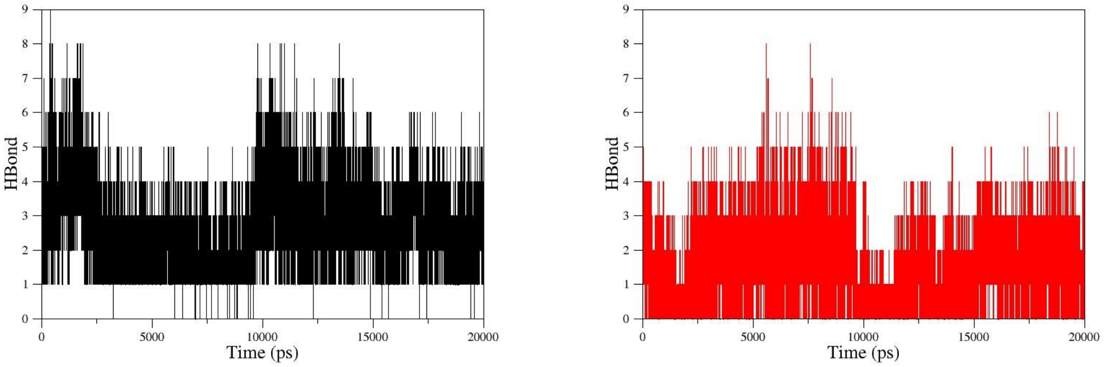
HBond
Time (ps)
HBond
Time (ps)

**(h)**

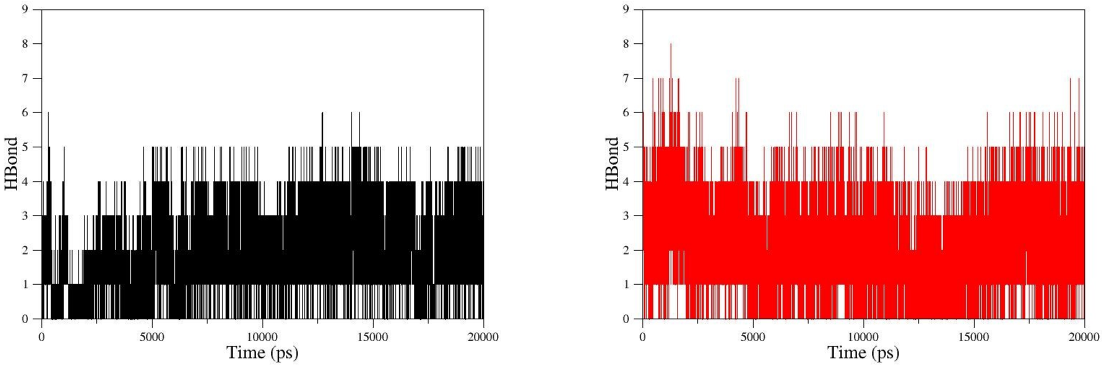
HBond
Time (ps)
HBond
Time (ps)

**(i)**

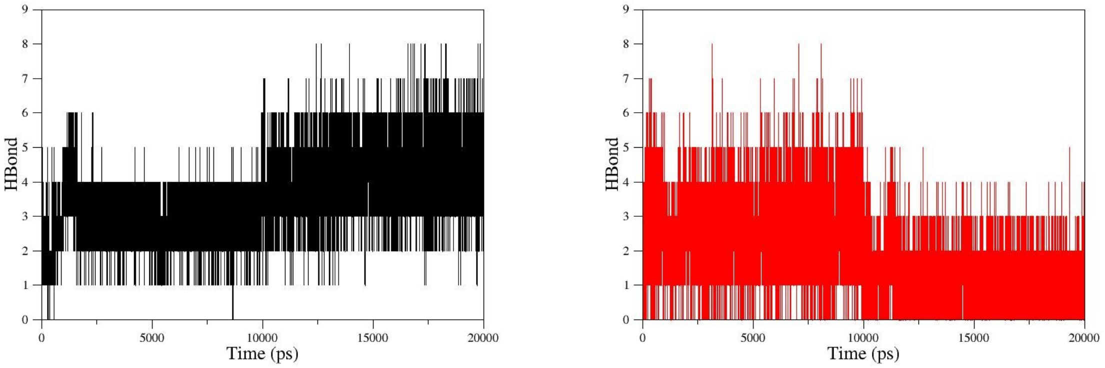
HBond
Time (ps)
HBond
Time (ps)

**(j)**

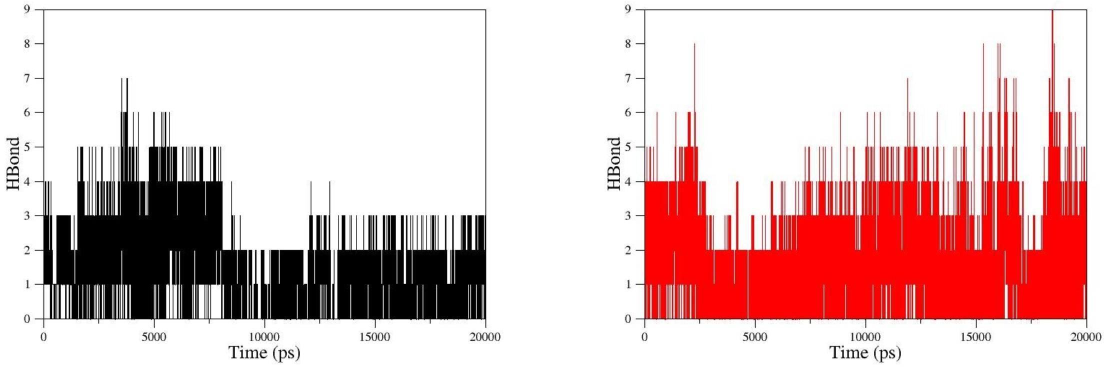
HBond
Time (ps)
HBond
Time (ps)

**(k)**

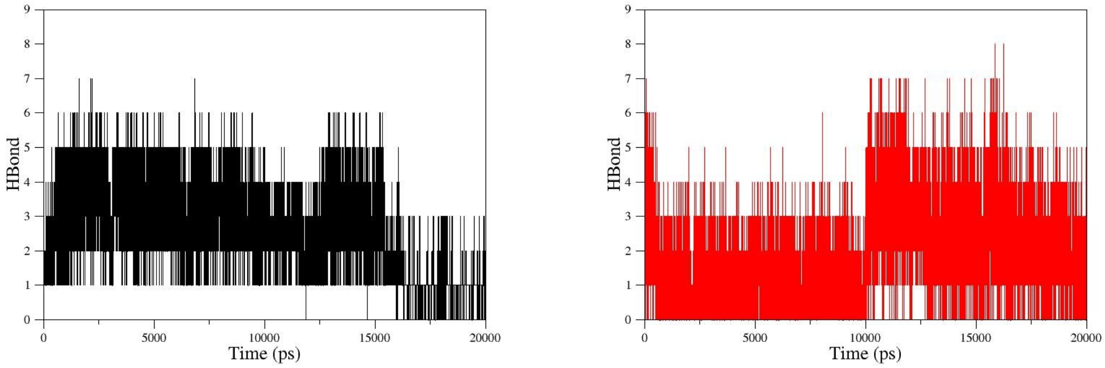
HBond
Time (ps)
HBond
Time (ps)

**(l)**

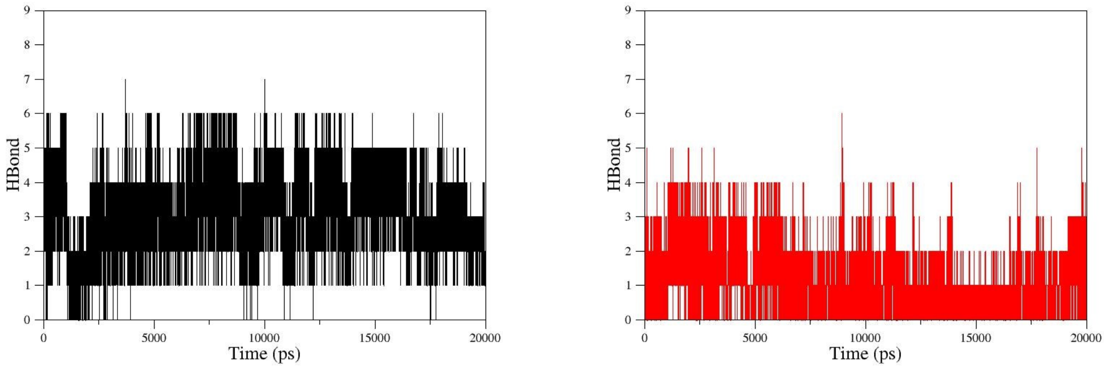
HBond
Time (ps)
HBond
Time (ps)

**(m)**

## **S17.** The percentage of hydrogen bond occupation

The percentage of hydrogen bond occupation between the residues of the enzyme AChE-POX and different oximes.

| **AChE-POX complexed by** | **Donor** | **Acceptor** | **Occupancy (%)** |
|---|---|---|---|
| **2-PAM** | 2-PAM (H1) | GLU202 (OE2) | 53.2 |
| | 2-PAM (H1) | GLU202 (OE1) | 53.4 |
| | ALA127 (H) | 2-PAM (O1) | 50.3 |
| **AE** | AE (H1) | SON203 (O3) | 12.1 |
| | AE (H1) | TRP86 (O) | 17.1 |
| | TYR133 (HH) | AE (O2) | 44.2 |
| | ALA127 (H) | AE (O2) | 12.0 |
| **BE** | BE (H1) | GLU202 (OE2) | 54.9 |
| | BE (H1) | GLU202 (OE1) | 46.0 |
| | GLY121 (H) | BE (N4) | 21.6 |
| **CE** | CE (H1) | GLU202 (OE2) | 53.8 |
| | CE (H1) | GLU202 (OE1) | 64.6 |
| | ALA127 (H) | CE (O1) | 34.5 |
| | ALA127 (H) | CE (O2) | 18.1 |
| | GLY121 (H) | CE (N5) | 13.5 |
| **DE** | DE (H1) | GLU202 (OE2) | 46.0 |
| | DE (H1) | GLU202 (OE1) | 59.4 |
| | TYR337 (HH) | DE (N3) | 14.0 |
| | TYR337 (HH) | DE (N2) | 22.9 |
| | ALA127 (H) | DE (O2) | 10.2 |
| | TYR124 (HH) | DE (N3) | 19.3 |
| | TYR124 (HH) | DE (N2) | 22.7 |
| | ASN87 (D21) | DE (O1) | 27.1 |
| **EE** | EE (H1) | GLU202 (OE2) | 58.3 |
| | EE (H1) | GLU202 (OE1) | 63.9 |
| | TYR337 (HH) | EE (N2) | 28.3 |
| | TYR337 (HH) | EE (N1) | 45.6 |
| | ALA127 (H) | EE (O1) | 85.2 |
| | GLY121 (H) | EE (N4) | 73.0 |
| **FE** | FE (H1) | HIS447 (NE2) | 16.0 |
| | FE (H1) | GLU202 (OE2) | 38.6 |
| | FE (H1) | GLU202 (OE1) | 53.0 |
| | TYR337 (HH) | FE (N2) | 22.7 |
| | TYR337 (HH) | FE (N1) | 35.1 |
| | ALA127 (H) | FE (O1) | 28.6 |
| | GLY121 (H) | FE (O3) | 19.7 |
| | GLY121 (H) | FE (N4) | 38.1 |
| **AZ** | AZ (H1) | GLU202 (OE2) | 35.1 |
| | AZ (H1) | GLU202 (OE1) | 59.8 |
| **BZ** | LYS348 (HZ1) | BZ (O2) | 18.4 |
| | LYS348 (HZ1) | BZ (N4) | 17.1 |
| | TYR341 (HH) | BZ (O2) | 12.0 |
| | ASN87 (D21) | BZ (N2) | 28.0 |
| | ASN87 (D21) | BZ (N1) | 67.4 |
| **CZ** | CZ (H1) | GLU202 (OE2) | 35.4 |
| | CZ (H1) | GLU202 (OE1) | 57.7 |
| | ALA127 (H) | CZ (O1) | 18.7 |
| | ALA127 (H) | CZ (O2) | 48.5 |
| **DZ** | TYR337 (HH) | DZ (O4) | 59.2 |
| | TYR337 (HH) | DZ (N5) | 39.6 |
| | ALA127 (H) | DZ (O1) | 12.9 |
| | TYR124 (HH) | DZ (N3) | 41.9 |
| | TYR124 (HH) | DZ (N2) | 29.8 |
| **EZ** | LYS348 (HZ1) | EZ (O3) | 31.3 |
| | TYR341 (HH) | EZ (O3) | 29.7 |
| | TYR341 (HH) | EZ (O2) | 21.3 |
| | TYR77 (H) | EZ (O3) | 17.0 |
| **FZ** | FZ (H1) | SON203 (O3) | 85.6 |
| | TYR337 (HH) | FZ (O3) | 49.1 |
| | TYR337 (HH) | FZ (N4) | 40.2 |
| | TYR133 (HH) | FZ (N2) | 56.7 |
| | TYR133 (HH) | FZ (N1) | 69.7 |
| | SER125 (HG) | FZ (O2) | 11.4 |
| | ASN87 (D21) | FZ (O1) | 10.9 |

## **S18.** COM distances between POX and oximes

COM distances between POX and oximes during simulation for oximes (a) A, (b) B, (c) C, (d) D, (e) E, and (f) F. *E* isomers of oximes (black), *Z* isomers of oximes (red).

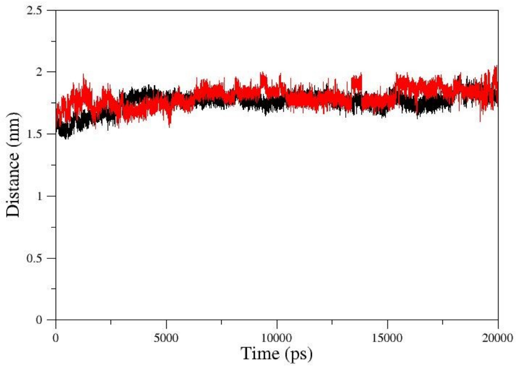


**(a)**

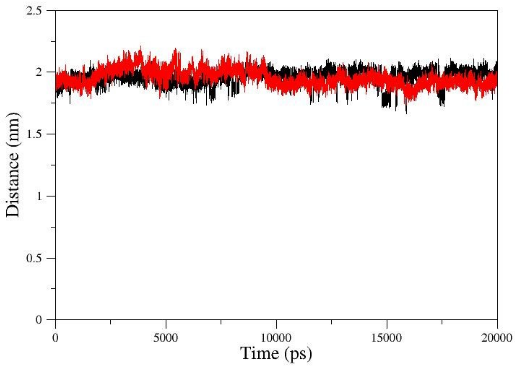


**(b)**

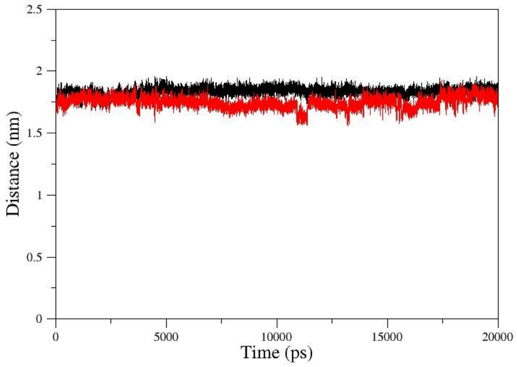


**(c)**

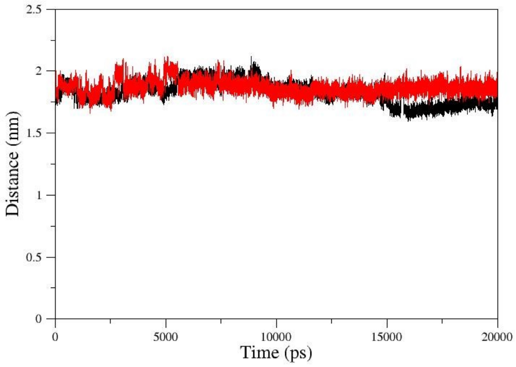


**(d)**

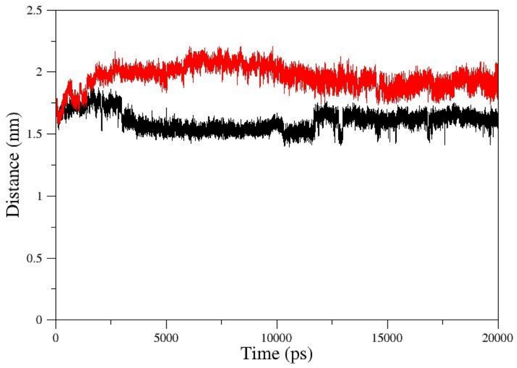


**(e)**

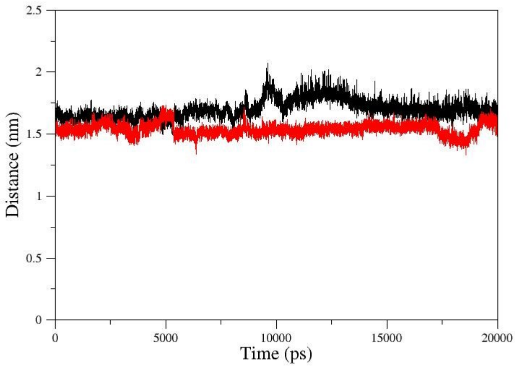


**(f)**

**S19.** M062X/6-31G* calculated cartesian coordinates of all optimized structures.

**Paraoxon-inhibited AChE (SON)**

| | | | |
|---|---|---|---|
| N | 3.22638900 | -3.00883000 | -0.73382000 |
| C | 2.15254200 | -2.20947700 | -0.15417900 |
| C | 0.87699100 | -3.01601600 | 0.00134500 |
| O | -0.21200800 | -2.67587800 | -0.41134200 |
| C | 1.95004300 | -0.96847600 | -1.01322700 |
| O | 0.91322100 | -0.12417200 | -0.47537800 |
| C | 0.73157100 | 2.87438200 | -0.98000400 |
| C | 1.41485300 | 4.10693100 | -1.52513500 |
| C | -0.75204400 | 0.13671900 | 2.16827400 |
| C | -2.17545600 | 0.52259200 | 2.49580300 |
| O | 1.60917200 | 2.28895900 | 0.01302300 |
| O | -0.20318800 | 1.15721300 | 1.29426100 |
| O | 2.30274100 | 0.44969900 | 1.63757100 |
| P | 1.25686900 | 0.91483100 | 0.71136100 |
| H | 2.89130800 | -0.41870600 | -1.08688200 |
| H | 1.61655200 | -1.23828800 | -2.01778700 |
| H | 3.57419500 | -3.68753700 | -0.06168700 |
| H | 2.89518900 | -3.53123200 | -1.54268800 |
| H | 2.46437700 | -1.89055300 | 0.84850200 |
| H | 1.00537900 | -3.99631000 | 0.49750600 |
| H | 0.55804500 | 2.13256200 | -1.76548400 |
| H | -0.21643600 | 3.12137200 | -0.49311000 |
| H | 1.59698700 | 4.82928900 | -0.72600900 |
| H | 2.36789100 | 3.84192900 | -1.98905500 |
| H | 0.77621100 | 4.57366800 | -2.27924800 |
| H | -2.19999900 | 1.49032800 | 3.00268800 |
| H | -2.78318700 | 0.59121200 | 1.58870400 |
| H | -2.61460200 | -0.22916200 | 3.15632500 |
| H | -0.12957400 | 0.08526500 | 3.06484900 |
| H | -0.70383600 | -0.83058900 | 1.65371800 |
| C | -2.59903500 | 0.63058500 | -1.16225400 |

| | | | |
|---|---|---|---|
| N | -1.93561300 | -0.53085500 | -0.84050000 |
| C | -3.92688000 | 0.38161400 | -0.99707400 |
| C | -2.81183400 | -1.46477300 | -0.48777800 |
| N | -4.02543400 | -0.92531000 | -0.57505000 |
| H | -4.79326400 | 1.00630600 | -1.13754700 |
| H | -2.57106700 | -2.47317100 | -0.19046500 |
| H | -4.88767800 | -1.41554100 | -0.36463000 |
| H | -2.08043300 | 1.52076400 | -1.47674800 |
| H | -0.92049300 | -0.68015000 | -0.84550200 |

**2-PAM**

| | | | |
|---|---|---|---|
| C | 2.57737300 | -0.76549500 | 0.00044600 |
| C | 1.57740900 | -1.76758200 | -0.00022900 |
| C | 0.25772200 | -1.41845600 | -0.00047700 |
| C | -0.14405800 | -0.04977100 | -0.00023900 |
| C | 2.17909500 | 0.53634800 | 0.00049600 |
| H | 3.63223200 | -1.00609000 | 0.00086600 |
| H | 1.85981900 | -2.81521700 | -0.00043800 |
| C | -1.49797700 | 0.34800400 | 0.00023100 |
| H | 2.87827600 | 1.36282200 | 0.00096000 |
| H | -1.81675300 | 1.38553100 | 0.00122400 |
| N | -2.43455400 | -0.61724700 | -0.00012000 |
| O | -3.62439000 | -0.22645200 | 0.00037500 |
| H | -0.53142500 | -2.15840300 | -0.00077700 |
| N | 0.85888700 | 0.88748100 | -0.00010500 |
| C | 0.50682200 | 2.31053200 | -0.00046000 |
| H | -0.07908200 | 2.54386800 | -0.89172800 |
| H | -0.07708100 | 2.54502100 | 0.89182700 |
| H | 1.42048400 | 2.90096100 | -0.00197500 |

**C1(2-PAM)**

| | | | |
|---|---|---|---|
| N | 5.02721600 | -2.53231200 | -2.15103400 |
| C | 3.96997600 | -1.61862200 | -1.75636300 |
| C | 3.86980100 | -1.65397600 | -0.22221400 |

| | | | |
|---|---|---|---|
| O | 2.88247400 | -0.74216500 | 0.27262200 |
| C | 4.15231200 | -0.18944400 | -2.24201000 |
| O | 3.30751300 | 0.67316100 | -2.12357400 |
| O | 0.91309400 | -1.93028900 | -0.95124800 |
| P | 1.32331400 | -1.19468000 | 0.25917300 |
| C | 1.11286500 | -1.38607800 | 2.88263900 |
| O | 0.69482000 | 0.23956200 | 0.51338100 |
| C | -0.25468500 | 0.78315600 | -0.43983900 |
| C | -0.98258900 | 1.92282700 | 0.23176100 |
| H | 5.00600800 | -2.68520800 | -3.15591100 |
| H | 3.01334500 | -1.97390500 | -2.15393000 |
| H | 3.64566900 | -2.67813500 | 0.09003800 |
| H | 4.81520900 | -1.35030800 | 0.23381300 |
| H | 5.12502700 | 0.03736700 | -2.72276900 |
| H | 2.12395900 | -1.03562400 | 3.11608900 |
| H | 0.42924900 | -0.53786200 | 2.81980000 |
| H | 0.30940000 | 1.12067400 | -1.31582300 |
| H | -0.94171600 | -0.00988000 | -0.74122600 |
| H | -1.70222400 | 2.35596300 | -0.46877600 |
| H | -0.28314000 | 2.70541100 | 0.54069700 |
| H | -1.52422000 | 1.55732200 | 1.10806400 |
| C | 2.55114700 | 4.02745300 | 1.32527100 |
| N | 2.94175900 | 4.31574900 | 0.03582100 |
| C | 2.45781100 | 2.67212500 | 1.40308800 |
| C | 3.08640700 | 3.18634100 | -0.65432600 |
| N | 2.79573700 | 2.18307000 | 0.16330800 |
| H | 2.17477700 | 2.02171400 | 2.21447100 |
| H | 3.38312600 | 3.09025800 | -1.68656400 |
| H | 2.82086000 | 1.19379500 | -0.09996200 |
| H | 2.37734900 | 4.79818100 | 2.05733000 |
| H | 5.94135800 | -2.13838400 | -1.93356300 |
| H | 3.09727200 | 5.24381300 | -0.34089200 |
| O | 1.14448100 | -2.03279100 | 1.58761900 |
| C | 0.61984200 | -2.40764300 | 3.88032700 |

| | | | |
|---|---|---|---|
| H | 1.27485400 | -3.28239100 | 3.90016100 |
| H | 0.59618600 | -1.96594700 | 4.88013400 |
| H | -0.39107100 | -2.72069300 | 3.60843700 |
| C | -6.93588400 | 1.03992500 | -0.76730600 |
| C | -6.31364400 | 1.23466100 | 0.48728600 |
| C | -5.12949300 | 0.61271900 | 0.76860300 |
| C | -4.50201400 | -0.23862900 | -0.18436700 |
| C | -6.32057400 | 0.22430700 | -1.66905200 |
| H | -7.87216300 | 1.51747200 | -1.02415100 |
| H | -6.77461200 | 1.88018300 | 1.22752200 |
| C | -3.27433500 | -0.89634200 | 0.06843700 |
| H | -6.72884100 | 0.02329700 | -2.65144600 |
| H | -2.78089900 | -1.53832000 | -0.65585600 |
| N | -2.69736800 | -0.70327000 | 1.26205100 |
| O | -1.58629400 | -1.26377900 | 1.43663100 |
| H | -4.62628000 | 0.74211800 | 1.71747400 |
| N | -5.13837700 | -0.39716400 | -1.38805900 |
| C | -4.53277700 | -1.25635000 | -2.41106300 |
| H | -3.54625200 | -0.87035800 | -2.67589900 |
| H | -4.43742100 | -2.27344900 | -2.02648900 |
| H | -5.17100700 | -1.25757800 | -3.29165000 |

**TS1(2-PAM)**

| | | | |
|---|---|---|---|
| N | 4.48004500 | -3.58308400 | -0.67149400 |
| C | 3.73874700 | -2.37811100 | -1.00602600 |
| C | 2.66001300 | -2.15014000 | 0.05799900 |
| O | 1.86431200 | -1.04709300 | -0.34072300 |
| C | 4.58906800 | -1.12565100 | -1.10427200 |
| O | 4.27381100 | -0.15157800 | -1.75334200 |
| O | -0.18314300 | -1.94126800 | 0.84113800 |
| P | 0.54607000 | -0.66855700 | 0.61053900 |
| C | 1.21999100 | 0.16308900 | 3.14326400 |
| O | 0.03524300 | 0.45033400 | -0.43104000 |
| C | -0.05777100 | 0.13466300 | -1.83529400 |

| | | | |
|---|---|---|---|
| C | -0.95866300 | 1.17424100 | -2.46438800 |
| H | 5.15272400 | -3.79534300 | -1.40447100 |
| H | 3.23326300 | -2.51260700 | -1.96799300 |
| H | 2.05095400 | -3.05537000 | 0.13335400 |
| H | 3.11897800 | -1.95088200 | 1.03736400 |
| H | 5.52854800 | -1.13585000 | -0.51399300 |
| H | 2.19924900 | 0.44828800 | 3.53470200 |
| H | 0.51522600 | 0.97687200 | 3.30516400 |
| H | 0.94502900 | 0.14894200 | -2.27258200 |
| H | -0.46401400 | -0.87727300 | -1.95282100 |
| H | -1.05861800 | 0.98346700 | -3.53615700 |
| H | -0.53645800 | 2.17340400 | -2.32456100 |
| H | -1.95197600 | 1.15055300 | -2.00557700 |
| C | 2.37639500 | 3.95905400 | 0.14994400 |
| N | 3.54520400 | 3.80482200 | -0.56353300 |
| C | 1.82564900 | 2.72063100 | 0.26084900 |
| C | 3.71189500 | 2.52400100 | -0.88234300 |
| N | 2.67646700 | 1.85289400 | -0.39103100 |
| H | 0.91764600 | 2.37743800 | 0.72990100 |
| H | 4.53285800 | 2.09337900 | -1.43310500 |
| H | 2.55365500 | 0.83693700 | -0.47985300 |
| H | 2.04640700 | 4.91978900 | 0.50794200 |
| H | 5.01833000 | -3.44221700 | 0.18247200 |
| H | 4.18860400 | 4.54650100 | -0.81429300 |
| O | 1.45157100 | 0.03627400 | 1.72456800 |
| C | 0.73812400 | -1.12300900 | 3.78197700 |
| H | 1.41887900 | -1.94808000 | 3.55067300 |
| H | 0.70874000 | -0.99249500 | 4.86770800 |
| H | -0.26121500 | -1.37823500 | 3.42621000 |
| C | -6.76039800 | 0.22673300 | -0.72306100 |
| C | -6.24814400 | 1.04480100 | 0.30048200 |
| C | -4.93804300 | 0.91439300 | 0.68832400 |
| C | -4.09093600 | -0.03535100 | 0.07457100 |
| C | -5.92265100 | -0.68225800 | -1.30564300 |

| | | | |
|---|---|---|---|
| H | -7.78701700 | 0.29899700 | -1.05724900 |
| H | -6.88637400 | 1.77727400 | 0.78309100 |
| C | -2.71528100 | -0.21307100 | 0.44882300 |
| H | -6.24029600 | -1.34511800 | -2.10003300 |
| H | -2.09488700 | -0.97787900 | -0.00089700 |
| N | -2.26194600 | 0.56684600 | 1.40684100 |
| O | -1.04264800 | 0.45185800 | 1.77438100 |
| H | -4.50732700 | 1.52483000 | 1.47063400 |
| N | -4.62432500 | -0.80787100 | -0.91667000 |
| C | -3.78329900 | -1.80383900 | -1.59891000 |
| H | -2.94799900 | -1.29974300 | -2.08879500 |
| H | -3.40798100 | -2.52760600 | -0.87464700 |
| H | -4.38544400 | -2.31412700 | -2.34669800 |

**IN(2-PAM)**

| | | | |
|---|---|---|---|
| N | 4.20015900 | -3.66906000 | -0.60617100 |
| C | 3.49378100 | -2.45686700 | -0.99327500 |
| C | 2.52273700 | -2.06574300 | 0.12859300 |
| O | 1.78201500 | -0.94079000 | -0.28107900 |
| C | 4.39702000 | -1.27150900 | -1.25954200 |
| O | 4.12640700 | -0.36945400 | -2.02363800 |
| O | -0.33223800 | -1.87937400 | 0.63394400 |
| P | 0.36677200 | -0.55354100 | 0.64627600 |
| C | 1.11584000 | 0.05199400 | 3.22809100 |
| O | 0.04167400 | 0.62486100 | -0.44041200 |
| C | 0.16472000 | 0.40517400 | -1.85407500 |
| C | -0.62454500 | 1.50062900 | -2.53974900 |
| H | 4.83360700 | -3.95893000 | -1.34772300 |
| H | 2.90694600 | -2.64338400 | -1.89847200 |
| H | 1.85985200 | -2.91534800 | 0.31937000 |
| H | 3.07996900 | -1.84582500 | 1.05256800 |
| H | 5.33748600 | -1.24983800 | -0.66909200 |
| H | 2.08903100 | 0.30296100 | 3.65897400 |
| H | 0.42969100 | 0.88114300 | 3.40347300 |

| | | | |
|---|---|---|---|
| H | 1.21901400 | 0.42746400 | -2.14488500 |
| H | -0.22686200 | -0.58845900 | -2.10424600 |
| H | -0.56608400 | 1.38377100 | -3.62531400 |
| H | -0.21997100 | 2.48125100 | -2.27244000 |
| H | -1.67612900 | 1.46727500 | -2.23875300 |
| C | 2.79322200 | 3.80741200 | 0.34561000 |
| N | 3.84761400 | 3.55733900 | -0.50517500 |
| C | 2.15051900 | 2.62113800 | 0.51777400 |
| C | 3.85489900 | 2.26959800 | -0.84464700 |
| N | 2.83195400 | 1.68665300 | -0.23102800 |
| H | 1.27780100 | 2.35507400 | 1.09085500 |
| H | 4.55509700 | 1.77843900 | -1.50223900 |
| H | 2.57628500 | 0.68186600 | -0.30643000 |
| H | 2.60076600 | 4.78893800 | 0.74538500 |
| H | 4.77969200 | -3.49073800 | 0.21333200 |
| H | 4.52087800 | 4.24092400 | -0.83045400 |
| O | 1.38437500 | -0.06624900 | 1.82201900 |
| C | 0.58895800 | -1.23892600 | 3.82440500 |
| H | 1.26795500 | -2.06688700 | 3.59808400 |
| H | 0.51387100 | -1.13885500 | 4.91126200 |
| H | -0.39876000 | -1.47476100 | 3.42298200 |
| C | -6.78228500 | 0.34503400 | -0.60865000 |
| C | -6.21040400 | 1.09003600 | 0.42814300 |
| C | -4.87214500 | 0.93169300 | 0.72292600 |
| C | -4.08438000 | 0.02672200 | -0.00039800 |
| C | -5.98583400 | -0.53321400 | -1.29994900 |
| H | -7.82645100 | 0.43997200 | -0.87688100 |
| H | -6.81216700 | 1.79103500 | 0.99598700 |
| C | -2.66490000 | -0.17339800 | 0.27651600 |
| H | -6.35837000 | -1.14546100 | -2.11072200 |
| H | -2.06578500 | -0.82518200 | -0.34495800 |
| N | -2.20705600 | 0.43942200 | 1.31818600 |
| O | -0.94198700 | 0.34058800 | 1.65780900 |
| H | -4.38872600 | 1.49209900 | 1.51214500 |

| | | | |
|---|---|---|---|
| N | -4.67286800 | -0.68666400 | -0.99799000 |
| C | -3.88721300 | -1.66454800 | -1.77714800 |
| H | -3.10043500 | -1.14640000 | -2.32624200 |
| H | -3.45634400 | -2.40336800 | -1.10145600 |
| H | -4.55397300 | -2.15749500 | -2.47989500 |

**TS2(2-PAM)**

| | | | |
|---|---|---|---|
| N | 3.91543500 | -3.82346500 | -0.28711200 |
| C | 3.19632300 | -2.68830400 | -0.86786900 |
| C | 2.73867500 | -1.74095200 | 0.22913500 |
| O | 1.92860400 | -0.71969600 | -0.32953200 |
| C | 4.06598300 | -1.98090500 | -1.89147200 |
| O | 4.44804000 | -0.83688400 | -1.78837200 |
| O | -0.29413500 | -1.67572000 | 0.55359900 |
| P | 0.14706300 | -0.28049000 | 0.80622800 |
| C | 1.09040300 | 0.07744800 | 3.29987200 |
| O | -0.01269600 | 0.86125300 | -0.30159800 |
| C | 0.04700700 | 0.60573600 | -1.72217100 |
| C | -0.93222600 | 1.54577400 | -2.39085500 |
| H | 4.08219700 | -4.53904200 | -0.99127500 |
| H | 2.30916000 | -3.07547600 | -1.38388800 |
| H | 2.14565800 | -2.30356400 | 0.95431900 |
| H | 3.60044900 | -1.29790700 | 0.74370400 |
| H | 4.40309100 | -2.61360500 | -2.73741700 |
| H | 2.05985000 | 0.39167500 | 3.68931500 |
| H | 0.33611900 | 0.80901600 | 3.59056900 |
| H | 1.07044000 | 0.78998400 | -2.05508700 |
| H | -0.19566700 | -0.44467800 | -1.91014800 |
| H | -0.89615800 | 1.40477500 | -3.47428000 |
| H | -0.67609400 | 2.58437100 | -2.16586400 |
| H | -1.95479500 | 1.35883300 | -2.04986800 |
| C | 3.36742000 | 3.81039500 | 0.07592400 |
| N | 4.42614400 | 3.35691100 | -0.67362200 |
| C | 2.56503700 | 2.72065600 | 0.26720600 |

| | | | |
|---|---|---|---|
| C | 4.24142300 | 2.04094700 | -0.90931100 |
| N | 3.12212600 | 1.62442900 | -0.35152500 |
| H | 1.63067800 | 2.64666500 | 0.80273300 |
| H | 4.92423900 | 1.43011300 | -1.47936600 |
| H | 2.46543800 | 0.15231100 | -0.37959600 |
| H | 3.28876100 | 4.83755300 | 0.39337300 |
| H | 4.83087900 | -3.52386000 | 0.04554600 |
| H | 5.20989100 | 3.90983900 | -0.99360200 |
| O | 1.26797500 | 0.13304200 | 1.86359000 |
| C | 0.73754600 | -1.31655200 | 3.77720000 |
| H | 1.48949800 | -2.03746400 | 3.44428100 |
| H | 0.70652100 | -1.32780400 | 4.87017200 |
| H | -0.24047600 | -1.62662600 | 3.40023000 |
| C | -6.83728600 | 0.37199100 | -0.79357700 |
| C | -6.28268300 | 1.27032400 | 0.11613200 |
| C | -4.95642500 | 1.12690600 | 0.49098900 |
| C | -4.19038200 | 0.08543100 | -0.02607500 |
| C | -6.04817800 | -0.64403300 | -1.28631100 |
| H | -7.86532200 | 0.44927300 | -1.12270100 |
| H | -6.87785700 | 2.08082400 | 0.52109600 |
| C | -2.77723800 | -0.10335500 | 0.33630600 |
| H | -6.41530400 | -1.37759100 | -1.99173500 |
| H | -2.14063800 | -0.73367100 | -0.27478700 |
| N | -2.38974600 | 0.49045900 | 1.40308900 |
| O | -1.10119500 | 0.39871200 | 1.78972600 |
| H | -4.48051900 | 1.81113200 | 1.18137700 |
| N | -4.75954700 | -0.78113800 | -0.90481300 |
| C | -3.97811000 | -1.90806800 | -1.46026300 |
| H | -3.19967900 | -1.52088300 | -2.11865700 |
| H | -3.54089400 | -2.48106500 | -0.64347700 |
| H | -4.65276000 | -2.54244500 | -2.02898500 |

**C2(2-PAM)**

| | | | |
|---|---|---|---|
| N | -3.17403400 | 3.45704200 | 2.34521500 |

| | | | |
|---|---|---|---|
| C | -2.63783800 | 2.92076800 | 1.09462600 |
| C | -3.19177000 | 1.51979900 | 0.87023200 |
| O | -2.77699500 | 1.07725800 | -0.40216500 |
| C | -3.00348900 | 3.78840300 | -0.09210300 |
| O | -2.20976900 | 4.30995600 | -0.84191200 |
| O | -0.14355000 | 0.91010700 | 1.26873500 |
| P | -0.09328700 | -0.13026600 | 0.23105200 |
| C | -1.36490900 | -2.20439400 | -0.75579200 |
| O | -0.01372200 | 0.32321800 | -1.28047400 |
| C | -0.07704100 | 1.72746900 | -1.64585100 |
| C | 1.30383900 | 2.34377500 | -1.60530700 |
| H | -2.76386100 | 4.36842900 | 2.53739700 |
| H | -1.54753700 | 2.86322500 | 1.16099900 |
| H | -2.82576300 | 0.86900900 | 1.67614700 |
| H | -4.29315800 | 1.55401800 | 0.93101900 |
| H | -4.09615400 | 3.95662000 | -0.21856700 |
| H | -2.04608200 | -1.72959400 | -1.46542000 |
| H | -0.40414800 | -2.38639900 | -1.24642100 |
| H | -0.49283000 | 1.72837200 | -2.65475700 |
| H | -0.77598500 | 2.23578200 | -0.98322400 |
| H | 1.24926400 | 3.38365600 | -1.94032200 |
| H | 1.98870800 | 1.80043200 | -2.26305600 |
| H | 1.70042200 | 2.32598300 | -0.58574100 |
| C | -5.54994500 | -3.09062300 | -0.74889900 |
| N | -5.53877700 | -2.75594900 | 0.58172700 |
| C | -4.86227100 | -2.08926800 | -1.37850800 |
| C | -4.86116000 | -1.59278700 | 0.71211500 |
| N | -4.43646700 | -1.15978800 | -0.45768800 |
| H | -4.64759800 | -1.98230200 | -2.43193300 |
| H | -4.70435000 | -1.11107600 | 1.66672600 |
| H | -3.28998500 | 0.25031100 | -0.57598000 |
| H | -6.03240300 | -3.98381900 | -1.11200300 |
| H | -4.17918800 | 3.60724200 | 2.25709600 |
| H | -5.96489300 | -3.28164600 | 1.33324500 |

| | | | |
|---|---|---|---|
| O | -1.17106700 | -1.27586000 | 0.34423800 |
| C | -1.93766900 | -3.48067200 | -0.18621800 |
| H | -2.14711000 | -4.18183600 | -0.99848700 |
| H | -1.22666200 | -3.94298100 | 0.50342400 |
| H | -2.86987500 | -3.27737100 | 0.34731000 |
| C | 7.25887600 | 0.58366500 | -0.29278200 |
| C | 6.13974400 | 1.24969700 | -0.78220900 |
| C | 4.88242700 | 0.69211700 | -0.59578800 |
| C | 4.74800500 | -0.51355400 | 0.08193000 |
| C | 7.08320700 | -0.61117700 | 0.37486700 |
| H | 8.25999700 | 0.97547700 | -0.41807100 |
| H | 6.24412700 | 2.19138500 | -1.30903000 |
| C | 3.43075400 | -1.13769400 | 0.29384700 |
| H | 7.91022500 | -1.17544700 | 0.78558000 |
| H | 3.33109700 | -2.19066200 | 0.54541000 |
| N | 2.40731000 | -0.39342200 | 0.14936200 |
| O | 1.24681800 | -1.10770900 | 0.34431900 |
| H | 3.98593500 | 1.16972500 | -0.97134200 |
| N | 5.85594500 | -1.13983900 | 0.55706200 |
| C | 5.74159900 | -2.41714900 | 1.29673300 |
| H | 5.04994300 | -2.29076600 | 2.12867600 |
| H | 5.39639800 | -3.19795000 | 0.61859300 |
| H | 6.72506400 | -2.67614500 | 1.67991900 |

**AE**

| | | | |
|---|---|---|---|
| C | -4.64709100 | 0.35899800 | -1.47694400 |
| C | -3.48956100 | 1.06903000 | -1.16832800 |
| C | -2.58853100 | 0.59517000 | -0.21971500 |
| C | -2.80591100 | -0.62533300 | 0.45895300 |
| C | -3.98603700 | -1.31815900 | 0.12367300 |
| C | -4.88937200 | -0.84664700 | -0.82105200 |
| H | -5.34397500 | 0.74158600 | -2.21564400 |
| H | -3.26314500 | 2.01524900 | -1.65194000 |
| C | -1.94002000 | -1.20409900 | 1.47963600 |

| | | | |
|---|---|---|---|
| H | -4.18725200 | -2.25779200 | 0.63301000 |
| H | -5.78438700 | -1.42028100 | -1.04309200 |
| H | -2.29163700 | -2.11942200 | 1.96905700 |
| O | -1.49211600 | 1.38981400 | 0.05304600 |
| N | -0.78557300 | -0.71180500 | 1.83170700 |
| O | -0.13487000 | -1.34982300 | 2.76364100 |
| C | 0.80783800 | 1.85817600 | -0.21739400 |
| C | 1.05743400 | 3.17592700 | -0.53639800 |
| N | 1.84142600 | 1.52803100 | 0.59420500 |
| H | 0.47945000 | 3.84190100 | -1.16058700 |
| N | 2.20143400 | 3.56677600 | 0.07322100 |
| N | 2.67522700 | 2.55875400 | 0.74944800 |
| C | -0.31095300 | 0.95251500 | -0.61407200 |
| H | -0.46426200 | 1.02081000 | -1.69906200 |
| H | -0.08407300 | -0.08147000 | -0.34343100 |
| C | 2.16784200 | 0.24276700 | 1.21421900 |
| H | 3.06093000 | 0.44858100 | 1.81105700 |
| H | 1.35638500 | -0.07448700 | 1.87689400 |
| C | 2.44799200 | -0.82473000 | 0.17888500 |
| C | 3.27305500 | -0.55948900 | -0.91670800 |
| C | 1.88587000 | -2.09350600 | 0.33163700 |
| C | 3.53953500 | -1.55634000 | -1.85069200 |
| H | 3.70455800 | 0.43226700 | -1.03646000 |
| C | 2.15910900 | -3.09199200 | -0.60290600 |
| H | 1.22747600 | -2.26961200 | 1.18082100 |
| C | 2.98374800 | -2.82649200 | -1.69385100 |
| H | 4.18054700 | -1.34320600 | -2.70089200 |
| H | 1.71870100 | -4.07755600 | -0.48305200 |
| H | 3.18927400 | -3.60357300 | -2.42391000 |

**C1(AE)**

| | | | |
|---|---|---|---|
| N | 7.00652400 | 1.01650800 | -2.42642100 |
| C | 5.81088100 | 1.01579100 | -1.60208700 |
| C | 5.13690100 | -0.35847600 | -1.76316300 |

| | | | |
|---|---|---|---|
| O | 3.99912900 | -0.51603000 | -0.90985000 |
| C | 6.05323100 | 1.33288800 | -0.13463400 |
| O | 5.16670500 | 1.47081000 | 0.68185900 |
| O | 2.77234800 | 1.55550100 | -1.90568700 |
| P | 2.59980100 | 0.20792200 | -1.33148300 |
| C | 1.48602900 | -2.11787100 | -1.91403500 |
| O | 1.87783900 | 0.06864600 | 0.07284500 |
| C | 1.41965100 | 1.28290000 | 0.72373500 |
| C | 0.55865000 | 0.88865000 | 1.89827300 |
| H | 7.37545700 | 1.95970500 | -2.51486400 |
| H | 5.10964700 | 1.76962400 | -1.97463500 |
| H | 4.85471900 | -0.48987100 | -2.81221700 |
| H | 5.83023500 | -1.15729500 | -1.48801500 |
| H | 7.11520900 | 1.44041000 | 0.16555800 |
| H | 2.29311700 | -2.82686800 | -2.12035600 |
| H | 1.29029900 | -2.09952200 | -0.83854500 |
| H | 2.30560700 | 1.84512700 | 1.03996600 |
| H | 0.84834000 | 1.86361600 | -0.00026700 |
| H | 0.26450400 | 1.78811600 | 2.44721300 |
| H | 1.09939300 | 0.22613600 | 2.58394800 |
| H | -0.34471000 | 0.38286200 | 1.54670400 |
| C | 3.06859800 | -1.69929800 | 3.73665000 |
| N | 3.58391600 | -0.47292400 | 4.09369700 |
| C | 3.25068300 | -1.81278400 | 2.39206600 |
| C | 4.06683800 | 0.14439600 | 3.01614100 |
| N | 3.87282200 | -0.65960100 | 1.97946500 |
| H | 2.99823000 | -2.60299600 | 1.70360500 |
| H | 4.53450400 | 1.11573000 | 2.97941300 |
| H | 4.09692500 | -0.42235300 | 1.00733300 |
| H | 2.63033600 | -2.36966100 | 4.45715000 |
| H | 7.73939600 | 0.45388100 | -1.99704000 |
| H | 3.60287300 | -0.08980200 | 5.03201600 |
| O | 1.93664300 | -0.81002300 | -2.34080300 |
| C | 0.22213900 | -2.43026300 | -2.68158700 |

| | | | |
|---|---|---|---|
| H | 0.42690800 | -2.50395900 | -3.75342500 |
| H | -0.20074000 | -3.37890900 | -2.33670500 |
| H | -0.49004000 | -1.62049100 | -2.49808900 |
| C | -4.50305400 | 4.77729700 | 1.24536900 |
| C | -4.84250600 | 3.52167700 | 0.74944400 |
| C | -3.90171000 | 2.73539400 | 0.08908800 |
| C | -2.57170900 | 3.17154800 | -0.08075400 |
| C | -2.26531900 | 4.45425400 | 0.41020200 |
| C | -3.20245000 | 5.24510300 | 1.06404500 |
| H | -5.24613900 | 5.38303500 | 1.75411000 |
| H | -5.85362100 | 3.13442500 | 0.84384700 |
| C | -1.52306600 | 2.41528200 | -0.76232400 |
| H | -1.25171900 | 4.82476000 | 0.27502800 |
| H | -2.91653200 | 6.22607500 | 1.43171000 |
| H | -0.67269400 | 2.97759900 | -1.16574700 |
| O | -4.33894900 | 1.54548700 | -0.45964400 |
| N | -1.53283100 | 1.11868200 | -0.86349500 |
| O | -0.52291300 | 0.55863700 | -1.47275700 |
| C | -5.02001600 | -0.69577000 | -0.19686200 |
| C | -6.34866800 | -1.06095600 | -0.22199600 |
| N | -4.42680500 | -1.60837300 | -1.00198700 |
| H | -7.18913000 | -0.59813600 | 0.27438800 |
| N | -6.48735800 | -2.15371500 | -1.01060700 |
| N | -5.31419400 | -2.48246500 | -1.47511000 |
| C | -4.30676200 | 0.44574100 | 0.44680600 |
| H | -4.82607800 | 0.71215000 | 1.37521700 |
| H | -3.26446700 | 0.18940900 | 0.66905800 |
| C | -3.00083700 | -1.78959700 | -1.26212500 |
| H | -2.94209700 | -2.39666700 | -2.16988500 |
| H | -2.54882800 | -0.81044700 | -1.44693400 |
| C | -2.31334700 | -2.46477100 | -0.09396600 |
| C | -2.87488900 | -3.58856500 | 0.51655600 |
| C | -1.10352600 | -1.94961900 | 0.37448200 |
| C | -2.22931200 | -4.19790500 | 1.58903900 |

| | | | |
|---|---|---|---|
| H | -3.82185500 | -3.98224800 | 0.15390100 |
| C | -0.46185600 | -2.56332100 | 1.45129700 |
| H | -0.67664100 | -1.07483700 | -0.12285900 |
| C | -1.01985500 | -3.68522100 | 2.05982500 |
| H | -2.67067300 | -5.07132400 | 2.05962600 |
| H | 0.47333500 | -2.14807900 | 1.81896300 |
| H | -0.51932400 | -4.15805200 | 2.89966000 |

**TS1(AE)**

| | | | |
|---|---|---|---|
| N | -6.86158600 | 1.92576000 | 1.55056000 |
| C | -5.83326300 | 1.49261100 | 0.61971900 |
| C | -4.60046600 | 1.06104500 | 1.42271800 |
| O | -3.56941300 | 0.69859200 | 0.51410100 |
| C | -6.25239500 | 0.35699500 | -0.29859800 |
| O | -5.67494900 | 0.08204100 | -1.32839000 |
| O | -1.81976400 | 2.11617200 | 1.67225900 |
| P | -1.99449100 | 0.79582500 | 1.02932100 |
| C | -1.24180100 | -0.95445600 | 3.00660700 |
| O | -1.38397100 | 0.44012000 | -0.40597900 |
| C | -1.71833300 | 1.27542800 | -1.53412300 |
| C | -0.77244600 | 0.91137000 | -2.65536900 |
| H | -7.65396700 | 2.31276600 | 1.04368600 |
| H | -5.53395900 | 2.33433600 | -0.01377000 |
| H | -4.28350700 | 1.90444500 | 2.04159900 |
| H | -4.83669500 | 0.20878700 | 2.07339500 |
| H | -7.12402800 | -0.23972500 | 0.03985900 |
| H | -1.77324100 | -1.83381200 | 3.37769800 |
| H | -0.24673300 | -1.23556800 | 2.66647300 |
| H | -2.76396300 | 1.09753000 | -1.80798600 |
| H | -1.61066700 | 2.32732900 | -1.24603900 |
| H | -0.98169200 | 1.52656700 | -3.53477200 |
| H | -0.89885100 | -0.14048600 | -2.92926200 |
| H | 0.26298200 | 1.07578800 | -2.34227000 |
| C | -2.39895900 | -3.56122700 | -1.57911600 |

| | | | |
|---|---|---|---|
| N | -3.59232000 | -3.45468400 | -2.25920200 |
| C | -2.30144400 | -2.45093800 | -0.79880000 |
| C | -4.20774800 | -2.32570000 | -1.91494300 |
| N | -3.43603800 | -1.70548900 | -1.03001700 |
| H | -1.53839200 | -2.12080400 | -0.11181600 |
| H | -5.15744200 | -1.96751100 | -2.27891000 |
| H | -3.66141300 | -0.80569500 | -0.58101900 |
| H | -1.73362700 | -4.39821900 | -1.71347300 |
| H | -7.21691100 | 1.13066600 | 2.08006500 |
| H | -3.95476400 | -4.12590600 | -2.92624400 |
| O | -2.01299900 | -0.56604500 | 1.84699100 |
| C | -1.17567700 | 0.12861300 | 4.06191800 |
| H | -2.17984100 | 0.47070000 | 4.33164500 |
| H | -0.69677800 | -0.27783500 | 4.95792800 |
| H | -0.59225100 | 0.97255200 | 3.69306400 |
| C | 3.76152400 | 4.36429800 | -2.40743500 |
| C | 4.28867800 | 3.41041100 | -1.54086800 |
| C | 3.46028100 | 2.67120300 | -0.70078100 |
| C | 2.06114400 | 2.85304300 | -0.70917100 |
| C | 1.56022200 | 3.83090000 | -1.58755100 |
| C | 2.38390100 | 4.57289800 | -2.42598600 |
| H | 4.41827300 | 4.93793100 | -3.05363100 |
| H | 5.35867400 | 3.23008600 | -1.48548400 |
| C | 1.10247800 | 2.14228900 | 0.14153900 |
| H | 0.48548400 | 4.00093400 | -1.60405600 |
| H | 1.95076600 | 5.31403100 | -3.09085400 |
| H | 0.10616700 | 2.57956600 | 0.24027300 |
| O | 4.06718900 | 1.79457400 | 0.17591300 |
| N | 1.39445700 | 1.03465000 | 0.74670000 |
| O | 0.46295800 | 0.45140600 | 1.46655600 |
| C | 4.78251000 | -0.39200100 | 0.71536600 |
| C | 6.08526700 | -0.45440400 | 1.16231500 |
| N | 4.14189900 | -1.30861700 | 1.48078000 |
| H | 6.94263500 | 0.11447700 | 0.83290200 |

| | | | |
|---|---|---|---|
| N | 6.16969200 | -1.38482100 | 2.14224700 |
| N | 4.98832700 | -1.90424500 | 2.32283600 |
| C | 4.14647200 | 0.46332700 | -0.33062500 |
| H | 4.77046500 | 0.45617400 | -1.23345600 |
| H | 3.14518400 | 0.09907000 | -0.57415200 |
| C | 2.74513400 | -1.74571600 | 1.43698700 |
| H | 2.65092100 | -2.44695600 | 2.27082800 |
| H | 2.08162400 | -0.89311700 | 1.60564900 |
| C | 2.42103300 | -2.42168000 | 0.12343400 |
| C | 3.24057700 | -3.44519000 | -0.35822800 |
| C | 1.30477000 | -2.02102200 | -0.61422200 |
| C | 2.94842800 | -4.07154900 | -1.56545000 |
| H | 4.11320100 | -3.74659300 | 0.21781900 |
| C | 1.01768000 | -2.64906700 | -1.82749900 |
| H | 0.68365000 | -1.20975400 | -0.23211700 |
| C | 1.83389900 | -3.67280800 | -2.30375600 |
| H | 3.58968200 | -4.86724500 | -1.93225200 |
| H | 0.15672500 | -2.33154200 | -2.40872000 |
| H | 1.60743500 | -4.15515200 | -3.24984400 |

**IN(AE)**

| | | | |
|---|---|---|---|
| N | -6.45699000 | 1.81855400 | 2.16274300 |
| C | -5.55664100 | 1.51714900 | 1.05800400 |
| C | -4.22358100 | 1.00064300 | 1.61802300 |
| O | -3.33026700 | 0.79346900 | 0.55412500 |
| C | -6.08463300 | 0.47011500 | 0.10348500 |
| O | -5.84183200 | 0.44362200 | -1.08453300 |
| O | -1.54910500 | 2.07646000 | 1.68292500 |
| P | -1.59592800 | 0.77218400 | 0.93856700 |
| C | -1.05673700 | -1.53536300 | 2.38509600 |
| O | -1.34332000 | 0.64530000 | -0.67977200 |
| C | -2.00058000 | 1.50901000 | -1.61343400 |
| C | -1.13095400 | 1.56444400 | -2.85313400 |
| H | -7.34142400 | 2.17512000 | 1.80749900 |

| | | | |
|---|---|---|---|
| H | -5.35218100 | 2.42723000 | 0.48517100 |
| H | -3.83313500 | 1.75273100 | 2.31016400 |
| H | -4.38821400 | 0.06575900 | 2.17764400 |
| H | -6.69559600 | -0.32992300 | 0.57434500 |
| H | -1.66761700 | -2.42390300 | 2.57219700 |
| H | -0.19943200 | -1.82939300 | 1.77415000 |
| H | -2.99985200 | 1.12241700 | -1.83897700 |
| H | -2.12425200 | 2.50487200 | -1.17067700 |
| H | -1.59097300 | 2.20389100 | -3.61185100 |
| H | -1.00539900 | 0.56146200 | -3.27229300 |
| H | -0.14121800 | 1.96441800 | -2.60974800 |
| C | -2.58993300 | -3.31981700 | -1.81872700 |
| N | -3.87219400 | -3.11230700 | -2.27671100 |
| C | -2.31881300 | -2.29182600 | -0.96928700 |
| C | -4.36746900 | -2.00062600 | -1.73243100 |
| N | -3.43790200 | -1.49162900 | -0.93395400 |
| H | -1.43668000 | -2.05153000 | -0.39868700 |
| H | -5.34386800 | -1.57667700 | -1.91007500 |
| H | -3.51572200 | -0.60601500 | -0.36960100 |
| H | -1.99606300 | -4.16303100 | -2.13130300 |
| H | -6.67785000 | 0.96642200 | 2.67697700 |
| H | -4.37194100 | -3.70484100 | -2.92891900 |
| O | -1.92151700 | -0.67733100 | 1.63074700 |
| C | -0.61543800 | -0.91396100 | 3.69591600 |
| H | -1.48702500 | -0.60404600 | 4.28043100 |
| H | -0.05025400 | -1.64577600 | 4.28153000 |
| H | 0.01913600 | -0.04518500 | 3.51062900 |
| C | 3.95928300 | 4.66838200 | -1.76388200 |
| C | 4.35666600 | 3.57563400 | -0.99874800 |
| C | 3.41270600 | 2.75445400 | -0.38742100 |
| C | 2.03704800 | 3.01178500 | -0.53433400 |
| C | 1.66269000 | 4.12706400 | -1.29848100 |
| C | 2.60258100 | 4.94504600 | -1.91459300 |
| H | 4.70607100 | 5.30121800 | -2.23269200 |

| | | | |
|---|---|---|---|
| H | 5.40734400 | 3.34407500 | -0.85036900 |
| C | 0.94827000 | 2.22577900 | 0.07213900 |
| H | 0.60274500 | 4.34585900 | -1.40701800 |
| H | 2.27796300 | 5.79690400 | -2.50306000 |
| H | -0.03493100 | 2.69418700 | 0.06856400 |
| O | 3.87572700 | 1.72043700 | 0.39549500 |
| N | 1.16369800 | 1.06822400 | 0.57270800 |
| O | 0.13987400 | 0.34724000 | 1.10247200 |
| C | 4.70090600 | -0.49296000 | 0.57224200 |
| C | 6.01739200 | -0.68640300 | 0.93131800 |
| N | 4.03803000 | -1.43507900 | 1.28562800 |
| H | 6.89519100 | -0.14666500 | 0.60699900 |
| N | 6.08487000 | -1.71238900 | 1.81258800 |
| N | 4.87973500 | -2.16475000 | 2.01685300 |
| C | 4.05709200 | 0.50402300 | -0.33179000 |
| H | 4.71476300 | 0.68681700 | -1.18969800 |
| H | 3.08943400 | 0.14036300 | -0.69344100 |
| C | 2.61532400 | -1.77291400 | 1.25830200 |
| H | 2.44778200 | -2.38220500 | 2.15054300 |
| H | 2.03864900 | -0.85035500 | 1.34015700 |
| C | 2.25117300 | -2.53152000 | -0.00073700 |
| C | 2.91209600 | -3.72348300 | -0.30915800 |
| C | 1.26444100 | -2.04705900 | -0.86144200 |
| C | 2.58740700 | -4.42905300 | -1.46299100 |
| H | 3.68575000 | -4.09319500 | 0.36022000 |
| C | 0.94244400 | -2.75570200 | -2.02104400 |
| H | 0.75280900 | -1.11569300 | -0.62230900 |
| C | 1.59937900 | -3.94554500 | -2.32215700 |
| H | 3.10393300 | -5.35562600 | -1.69371700 |
| H | 0.17939100 | -2.37017100 | -2.69167900 |
| H | 1.34768000 | -4.49398400 | -3.22467400 |

**TS2(AE)**

| | | | |
|---|---|---|---|
| N | -6.38530900 | 1.77836000 | 2.11535500 |

| | | | |
|---|---|---|---|
| C | -5.46978200 | 1.57467800 | 1.00181300 |
| C | -4.25667900 | 0.77169300 | 1.49199400 |
| O | -3.30843000 | 0.65824100 | 0.45297900 |
| C | -6.07206000 | 0.82496200 | -0.16806500 |
| O | -5.72381900 | 0.96614400 | -1.31985600 |
| O | -1.62982100 | 2.06508500 | 1.62321000 |
| P | -1.46914800 | 0.72822900 | 0.97052700 |
| C | -1.03933200 | -1.47887500 | 2.55455200 |
| O | -1.18037400 | 0.55901700 | -0.62050000 |
| C | -1.78214100 | 1.41979300 | -1.60103000 |
| C | -0.81631300 | 1.51720000 | -2.76355600 |
| H | -7.18612400 | 2.32932700 | 1.81447900 |
| H | -5.10392900 | 2.53988700 | 0.63769900 |
| H | -3.81341100 | 1.30610300 | 2.33569100 |
| H | -4.56953500 | -0.22661400 | 1.83375500 |
| H | -6.84691000 | 0.07555200 | 0.10348700 |
| H | -1.63047000 | -2.38647700 | 2.70452800 |
| H | -0.10858300 | -1.74848500 | 2.05119800 |
| H | -2.74257800 | 0.99779600 | -1.91121200 |
| H | -1.97426000 | 2.40240900 | -1.15523400 |
| H | -1.24009900 | 2.14657300 | -3.55109800 |
| H | -0.62089900 | 0.52418500 | -3.17883200 |
| H | 0.13506700 | 1.95219200 | -2.44189100 |
| C | -2.95395300 | -3.37044700 | -1.75297700 |
| N | -4.09968500 | -2.91307300 | -2.35999600 |
| C | -2.62180600 | -2.42477100 | -0.82800500 |
| C | -4.43635500 | -1.73372800 | -1.81775400 |
| N | -3.55685400 | -1.41640700 | -0.88094800 |
| H | -1.78659900 | -2.38306700 | -0.14618200 |
| H | -5.28631600 | -1.13737500 | -2.11358600 |
| H | -3.47889400 | -0.35986300 | -0.20555100 |
| H | -2.48913400 | -4.30253200 | -2.03104800 |
| H | -6.75708500 | 0.88291300 | 2.43104000 |
| H | -4.60810500 | -3.38083200 | -3.09950200 |

| | | | |
|---|---|---|---|
| O | -1.85196700 | -0.68596500 | 1.67170900 |
| C | -0.78651200 | -0.78267200 | 3.87676700 |
| H | -1.73520000 | -0.50077700 | 4.34330300 |
| H | -0.25486700 | -1.45711600 | 4.55468300 |
| H | -0.18084000 | 0.11396500 | 3.72907100 |
| C | 3.99434400 | 4.71831600 | -1.65673000 |
| C | 4.40750900 | 3.57463400 | -0.97945300 |
| C | 3.48010100 | 2.74428500 | -0.35612900 |
| C | 2.10663300 | 3.04675700 | -0.40136200 |
| C | 1.71554200 | 4.20753900 | -1.08453000 |
| C | 2.63922600 | 5.03528000 | -1.71147700 |
| H | 4.72879400 | 5.35789300 | -2.13569100 |
| H | 5.45783100 | 3.30694200 | -0.91311200 |
| C | 1.03317100 | 2.25757500 | 0.22619200 |
| H | 0.65719600 | 4.45543900 | -1.11959900 |
| H | 2.30343700 | 5.92468600 | -2.23429000 |
| H | 0.04553800 | 2.71891900 | 0.23110300 |
| O | 3.95943700 | 1.65306300 | 0.33138000 |
| N | 1.25594700 | 1.10873000 | 0.74077400 |
| O | 0.21534200 | 0.40369500 | 1.28824200 |
| C | 4.74161700 | -0.58173100 | 0.31267800 |
| C | 6.08410900 | -0.84599100 | 0.47726400 |
| N | 4.13879800 | -1.50708600 | 1.09763500 |
| H | 6.93314500 | -0.34258400 | 0.03834700 |
| N | 6.22297200 | -1.89433900 | 1.32304200 |
| N | 5.03746500 | -2.29256300 | 1.68965200 |
| C | 4.02853700 | 0.46973300 | -0.46830100 |
| H | 4.59344200 | 0.68057900 | -1.38349300 |
| H | 3.01743700 | 0.14530200 | -0.73956800 |
| C | 2.71098700 | -1.78680900 | 1.25064200 |
| H | 2.62483200 | -2.35040600 | 2.18319500 |
| H | 2.18778300 | -0.83510400 | 1.35600300 |
| C | 2.17954100 | -2.57973500 | 0.07501300 |
| C | 2.74871100 | -3.81792200 | -0.23582100 |

| | | | |
|---|---|---|---|
| C | 1.13892100 | -2.08216000 | -0.71124600 |
| C | 2.28362400 | -4.55306500 | -1.32051600 |
| H | 3.56358900 | -4.19831600 | 0.37602500 |
| C | 0.67794200 | -2.81984500 | -1.80375500 |
| H | 0.68705700 | -1.12126100 | -0.47086100 |
| C | 1.24644400 | -4.05295900 | -2.10952300 |
| H | 2.73003600 | -5.51517600 | -1.55276900 |
| H | -0.12926900 | -2.42437400 | -2.41363400 |
| H | 0.88672300 | -4.62373300 | -2.96040600 |

**C2(AE)**

| | | | |
|---|---|---|---|
| N | 4.83975500 | 4.08642900 | 0.87605000 |
| C | 3.96648500 | 2.96906600 | 1.23572000 |
| C | 4.43958200 | 1.70944900 | 0.52316700 |
| O | 3.70648700 | 0.61798700 | 1.02899100 |
| C | 3.96945600 | 2.71564000 | 2.72943800 |
| O | 2.99952500 | 2.78906400 | 3.44923300 |
| O | 1.55885800 | 2.10347800 | -0.72614400 |
| P | 1.24481800 | 0.66290600 | -0.74426800 |
| C | 2.23127200 | -1.69890500 | -1.28907200 |
| O | 0.88009000 | -0.01747800 | 0.63800500 |
| C | 0.88387900 | 0.70929400 | 1.88921500 |
| C | -0.54604600 | 0.92567400 | 2.33435100 |
| H | 4.50127400 | 4.94504800 | 1.30502100 |
| H | 2.94290800 | 3.19240400 | 0.92165100 |
| H | 4.29415500 | 1.84250200 | -0.55785500 |
| H | 5.51908400 | 1.57505300 | 0.70999400 |
| H | 4.97292700 | 2.48958700 | 3.15425400 |
| H | 2.71017800 | -1.94660700 | -0.33813900 |
| H | 1.17852200 | -1.99684500 | -1.24580000 |
| H | 1.44530400 | 0.08864800 | 2.59005100 |
| H | 1.42622500 | 1.64633800 | 1.76106400 |
| H | -0.56017800 | 1.42839800 | 3.30589600 |
| H | -1.06618800 | -0.03291800 | 2.42801200 |

| | | | |
|---|---|---|---|
| H | -1.08507000 | 1.54663600 | 1.61122100 |
| C | 6.24847700 | -3.15978400 | -1.09253100 |
| N | 6.61036600 | -2.03370100 | -1.78783500 |
| C | 5.42777200 | -2.71710700 | -0.09094400 |
| C | 6.01135200 | -0.97250600 | -1.20074500 |
| N | 5.28665500 | -1.35063600 | -0.16756500 |
| H | 4.93090700 | -3.29512000 | 0.67479100 |
| H | 6.13075400 | 0.03946900 | -1.56063400 |
| H | 4.15060300 | -0.18980200 | 0.67446300 |
| H | 6.59839800 | -4.14151500 | -1.36839800 |
| H | 5.77657600 | 3.92929400 | 1.24785900 |
| H | 7.21812700 | -1.99969500 | -2.59531100 |
| O | 2.31530300 | -0.26017100 | -1.45537200 |
| C | 2.93509600 | -2.34271100 | -2.46042800 |
| H | 2.93829600 | -3.42867900 | -2.33429900 |
| H | 2.42295700 | -2.09877600 | -3.39489700 |
| H | 3.97015900 | -1.99594800 | -2.52192400 |
| C | -4.79507500 | 4.47515400 | -0.63809100 |
| C | -4.93436800 | 3.09589600 | -0.51845100 |
| C | -3.87354500 | 2.25294000 | -0.83970200 |
| C | -2.65482100 | 2.78839700 | -1.28507800 |
| C | -2.54458700 | 4.17816200 | -1.43090300 |
| C | -3.59806800 | 5.02045300 | -1.10026700 |
| H | -5.62844100 | 5.12215900 | -0.38399900 |
| H | -5.86901000 | 2.65090300 | -0.19003100 |
| C | -1.47357600 | 1.98455000 | -1.61959700 |
| H | -1.60971600 | 4.59371600 | -1.79671400 |
| H | -3.48942500 | 6.09407300 | -1.20898500 |
| H | -0.71896700 | 2.44715900 | -2.25811200 |
| O | -4.06393200 | 0.89658300 | -0.78828300 |
| N | -1.27977300 | 0.80899500 | -1.15710200 |
| O | -0.04988600 | 0.28460500 | -1.66836700 |
| C | -4.91817100 | -0.87549000 | 0.51940800 |
| C | -6.15844600 | -1.12150600 | 1.06444200 |

| | | | |
|---|---|---|---|
| N | -4.64004900 | -2.01065500 | -0.16293700 |
| H | -6.77093900 | -0.47942700 | 1.68018400 |
| N | -6.55200400 | -2.36749300 | 0.70194500 |
| N | -5.62447200 | -2.89915200 | -0.04198800 |
| C | -4.02507700 | 0.31684600 | 0.52093600 |
| H | -4.38427800 | 1.03180600 | 1.26743700 |
| H | -2.98747700 | 0.04692700 | 0.75323700 |
| C | -3.40143400 | -2.36162300 | -0.85634800 |
| H | -3.68518800 | -3.04402600 | -1.66064900 |
| H | -3.00158600 | -1.44275600 | -1.29492700 |
| C | -2.40363700 | -2.99140600 | 0.09011900 |
| C | -2.65936000 | -4.24066800 | 0.66182200 |
| C | -1.23012600 | -2.31276100 | 0.41394200 |
| C | -1.74543400 | -4.80287900 | 1.54813100 |
| H | -3.57789000 | -4.76695900 | 0.41410200 |
| C | -0.31226400 | -2.87759500 | 1.30026900 |
| H | -1.03181000 | -1.33800900 | -0.02331500 |
| C | -0.56953300 | -4.12200400 | 1.86841800 |
| H | -1.94769400 | -5.77427900 | 1.98892100 |
| H | 0.59870000 | -2.33557400 | 1.53822700 |
| H | 0.14183600 | -4.56328300 | 2.55980600 |

**AZ**

| | | | |
|---|---|---|---|
| C | -2.44200700 | -2.83187500 | -0.58644700 |
| C | -1.50614100 | -1.79277500 | -0.54019400 |
| C | -1.92288000 | -0.49873600 | -0.25167800 |
| C | -3.28590800 | -0.18659200 | 0.00608900 |
| C | -4.19492200 | -1.25826000 | -0.05154500 |
| C | -3.78034800 | -2.55928300 | -0.34392700 |
| H | -2.10915800 | -3.84017600 | -0.81286400 |
| H | -0.46106100 | -2.01278600 | -0.73032400 |
| C | -3.65184200 | 1.19384800 | 0.30701300 |
| H | -5.23255800 | -1.02665200 | 0.14302900 |
| H | -4.51445900 | -3.35954800 | -0.38053300 |

| | | | |
|---|---|---|---|
| O | -1.05374900 | 0.56491400 | -0.19796900 |
| C | 1.02827700 | 1.59683000 | -0.23254700 |
| C | 0.63936900 | 2.80747500 | 0.29261600 |
| N | 2.31701500 | 1.79827800 | -0.58973400 |
| H | -0.31861800 | 3.09674300 | 0.69672400 |
| N | 1.69428500 | 3.66095800 | 0.23172600 |
| N | 2.70685200 | 3.04552700 | -0.29854300 |
| C | 0.31219500 | 0.29683300 | -0.39873800 |
| H | 0.49074600 | -0.11867700 | -1.40194300 |
| H | 0.67934100 | -0.43835400 | 0.33247300 |
| N | -4.82720400 | 1.69927100 | 0.61540500 |
| O | -5.86708800 | 0.92637300 | 0.68232800 |
| H | -2.85223200 | 1.92735600 | 0.28106700 |
| C | 3.24803500 | 0.84958800 | -1.19293800 |
| H | 2.90274900 | 0.60468400 | -2.20189500 |
| H | 4.19506700 | 1.38831900 | -1.27695900 |
| C | 3.38108200 | -0.39787100 | -0.34949500 |
| C | 3.17007500 | -1.65801800 | -0.90690800 |
| C | 3.68766200 | -0.28693300 | 1.00937900 |
| C | 3.26888100 | -2.80163000 | -0.11445900 |
| H | 2.92124500 | -1.74635800 | -1.96168100 |
| C | 3.78174400 | -1.42641900 | 1.80110700 |
| H | 3.84159000 | 0.69759200 | 1.44560000 |
| C | 3.57154900 | -2.68664300 | 1.23945600 |
| H | 3.10170500 | -3.77919700 | -0.55561000 |
| H | 4.01685700 | -1.33312800 | 2.85673000 |
| H | 3.63992200 | -3.57558300 | 1.85863100 |

**C1(AZ)**

| | | | |
|---|---|---|---|
| N | 8.28771900 | -1.64705200 | -2.10068800 |
| C | 7.04161700 | -0.97444200 | -1.73545200 |
| C | 7.21719600 | 0.53062500 | -1.80083600 |
| O | 7.26662800 | 1.25897600 | -0.83159400 |
| C | 6.64033500 | -1.45476600 | -0.34401700 |

| | | | |
|---|---|---|---|
| O | 5.55878500 | -0.70558300 | 0.22391800 |
| C | 2.97296600 | -2.75330800 | 0.53283400 |
| C | 2.23584100 | -3.95925800 | -0.00066300 |
| C | 2.47437200 | 1.25164900 | 0.32911500 |
| C | 1.35945400 | 1.20639400 | 1.34597800 |
| O | 3.66250600 | -2.14216800 | -0.58350500 |
| O | 3.36676200 | 0.14348100 | 0.61399100 |
| O | 4.20537000 | 0.01038000 | -1.87039100 |
| P | 4.12315400 | -0.62913800 | -0.54038700 |
| H | 6.37586200 | -2.51524300 | -0.38272400 |
| H | 7.47560700 | -1.32451400 | 0.34837300 |
| H | 8.47996700 | -1.53847400 | -3.09337200 |
| H | 9.07445600 | -1.24227300 | -1.59577300 |
| H | 6.28050600 | -1.25078800 | -2.47110600 |
| H | 7.39075000 | 0.93724100 | -2.81473100 |
| H | 3.72142100 | -3.02283900 | 1.28614700 |
| H | 2.27343400 | -2.02123700 | 0.93945900 |
| H | 1.50562500 | -3.62201100 | -0.74016600 |
| H | 2.92607900 | -4.67145200 | -0.46123800 |
| H | 1.70937800 | -4.46276800 | 0.81590200 |
| H | 0.82017900 | 0.26198100 | 1.23006600 |
| H | 1.75563500 | 1.28855000 | 2.36296400 |
| H | 0.66769900 | 2.03610000 | 1.17409300 |
| H | 2.08364300 | 1.15308000 | -0.68279500 |
| H | 3.07144300 | 2.16683400 | 0.41316500 |
| C | 4.96441900 | 1.86763100 | 2.55081300 |
| N | 5.76711200 | 1.84874600 | 1.43403500 |
| C | 4.87240900 | 3.16753800 | 2.94276500 |
| C | 6.15854700 | 3.08117100 | 1.13978700 |
| N | 5.62401200 | 3.89662000 | 2.04666300 |
| H | 4.35011200 | 3.63232800 | 3.76215100 |
| H | 6.78852200 | 3.35803500 | 0.30967600 |
| H | 5.75457300 | 4.90130800 | 2.06530000 |
| H | 4.52700800 | 0.96749100 | 2.95023000 |

| | | | |
|---|---|---|---|
| H | 6.01416200 | 1.01976500 | 0.88027300 |
| C | -2.94165300 | -2.51981400 | 2.08976000 |
| C | -3.62371800 | -1.50886100 | 1.40385100 |
| C | -2.96522200 | -0.76864400 | 0.42871000 |
| C | -1.60311300 | -1.00513100 | 0.09977600 |
| C | -0.95414400 | -2.03165900 | 0.80905400 |
| C | -1.61242200 | -2.77724200 | 1.78959200 |
| H | -3.46319400 | -3.09196900 | 2.85086700 |
| H | -4.66369100 | -1.31534900 | 1.64393600 |
| C | -0.97693400 | -0.18545500 | -0.93648500 |
| H | 0.07934900 | -2.22358200 | 0.55663400 |
| H | -1.07614800 | -3.56323800 | 2.31415200 |
| O | -3.57923900 | 0.23762700 | -0.27578200 |
| C | -5.39596300 | 1.50234300 | -0.99396200 |
| C | -4.82272400 | 2.01890300 | -2.13315200 |
| N | -6.57285800 | 2.16179700 | -0.89641500 |
| H | -3.87349000 | 1.77704900 | -2.58623600 |
| N | -5.66787700 | 2.94392400 | -2.65783900 |
| N | -6.72515000 | 3.02412600 | -1.90905800 |
| C | -4.94964800 | 0.44783200 | -0.03511900 |
| H | -5.12435300 | 0.76677400 | 1.00327200 |
| H | -5.52002800 | -0.47886200 | -0.19520400 |
| N | 0.25467100 | -0.20705800 | -1.38391300 |
| O | 1.10493200 | -1.05951600 | -0.87993200 |
| H | -1.60542600 | 0.56017600 | -1.41289300 |
| C | -7.60314800 | 2.03229000 | 0.13105100 |
| H | -7.21785800 | 2.43698100 | 1.07169300 |
| H | -8.42631200 | 2.66791800 | -0.20396200 |
| C | -8.03028100 | 0.59237400 | 0.29937700 |
| C | -7.94446100 | -0.03097100 | 1.54337300 |
| C | -8.47558200 | -0.13552600 | -0.80723700 |
| C | -8.29846700 | -1.37270500 | 1.68303300 |
| H | -7.59097200 | 0.53094300 | 2.40455400 |
| C | -8.82639600 | -1.47437200 | -0.66947700 |

| | | | |
|---|---|---|---|
| H | -8.53492300 | 0.34906000 | -1.77934700 |
| C | -8.73648700 | -2.09569500 | 0.57692300 |
| H | -8.22394500 | -1.85132000 | 2.65444200 |
| H | -9.16694500 | -2.03536500 | -1.53410700 |
| H | -9.00555300 | -3.14195900 | 0.68265300 |

**TS1(AZ)**

| | | | |
|---|---|---|---|
| N | 7.54501400 | -3.18436000 | -0.56456200 |
| C | 6.72624600 | -1.99224000 | -0.71697400 |
| C | 5.76832500 | -1.90619400 | 0.48566000 |
| O | 4.94405700 | -0.75548400 | 0.42608000 |
| C | 7.51136400 | -0.69932500 | -0.81487100 |
| O | 7.04590400 | 0.34295100 | -1.22624200 |
| O | 3.79043800 | -1.54112900 | -1.68848600 |
| P | 3.48521200 | -0.87403500 | -0.40206000 |
| C | 1.91527100 | -1.08324300 | 1.70685900 |
| O | 3.23244200 | 0.69802700 | -0.24692500 |
| C | 3.16332400 | 1.48487400 | -1.45924900 |
| C | 2.63174700 | 2.84992200 | -1.09199000 |
| H | 8.11218300 | -3.33144100 | -1.39599400 |
| H | 6.10582500 | -2.07529500 | -1.61388600 |
| H | 5.17138700 | -2.82383000 | 0.51149400 |
| H | 6.34065700 | -1.84692100 | 1.41818300 |
| H | 8.56226600 | -0.74546800 | -0.46016000 |
| H | 2.53144400 | -0.33627100 | 2.22127400 |
| H | 1.08163500 | -0.58127200 | 1.21064900 |
| H | 4.17537800 | 1.54369900 | -1.87905500 |
| H | 2.50229100 | 0.97179500 | -2.15758100 |
| H | 2.55075200 | 3.46671800 | -1.99174400 |
| H | 3.29319900 | 3.35863700 | -0.38404000 |
| H | 1.64053200 | 2.73389200 | -0.64907600 |
| C | 5.39736600 | 3.81383400 | 1.82894600 |
| N | 6.43268000 | 3.92600600 | 0.92717100 |
| C | 4.91209200 | 2.54945900 | 1.69540800 |

| | | | |
|---|---|---|---|
| C | 6.58169900 | 2.77791600 | 0.26752500 |
| N | 5.66535800 | 1.93465900 | 0.72344400 |
| H | 4.09829800 | 2.04321900 | 2.18746800 |
| H | 7.31114100 | 2.56108200 | -0.49645900 |
| H | 5.52637400 | 0.96079600 | 0.40812300 |
| H | 5.10334200 | 4.63023500 | 2.46706000 |
| H | 8.19490000 | -3.07171400 | 0.21261600 |
| H | 7.00243300 | 4.75119200 | 0.78001700 |
| O | 2.71115100 | -1.73282600 | 0.70035700 |
| C | 1.43974700 | -2.14771700 | 2.67072200 |
| H | 2.28846000 | -2.65128900 | 3.14144500 |
| H | 0.82819800 | -1.68991600 | 3.45331400 |
| H | 0.83239300 | -2.89058900 | 2.14630200 |
| C | -2.89692700 | -3.40988900 | 0.50849500 |
| C | -3.54649000 | -2.17929200 | 0.38207400 |
| C | -2.82680800 | -1.05656700 | -0.01611200 |
| C | -1.43808900 | -1.12375800 | -0.29679900 |
| C | -0.82116500 | -2.37833300 | -0.16272900 |
| C | -1.53931600 | -3.50656300 | 0.23832800 |
| H | -3.46595300 | -4.28156400 | 0.81679600 |
| H | -4.60788100 | -2.11687600 | 0.59546500 |
| C | -0.73402000 | 0.11510700 | -0.65144900 |
| H | 0.23760300 | -2.43609900 | -0.37789000 |
| H | -1.02943000 | -4.46072500 | 0.33541400 |
| O | -3.40250500 | 0.17947300 | -0.16040000 |
| C | -5.16062600 | 1.70567900 | -0.15586900 |
| C | -4.54879300 | 2.70464700 | -0.87724000 |
| N | -6.31218500 | 2.27261600 | 0.27055000 |
| H | -3.60237600 | 2.68607700 | -1.39568400 |
| N | -5.34821200 | 3.80280400 | -0.85307200 |
| N | -6.41375500 | 3.53523300 | -0.16152300 |
| C | -4.77596000 | 0.29113000 | 0.12936100 |
| H | -4.97800500 | 0.03520000 | 1.17986200 |
| H | -5.36481900 | -0.39447100 | -0.49701400 |

| | | | |
|---|---|---|---|
| N | 0.50393900 | 0.29136300 | -1.01426100 |
| O | 1.26388500 | -0.77375800 | -1.18587200 |
| H | -1.30284700 | 1.03624200 | -0.56814000 |
| C | -7.36315600 | 1.68157300 | 1.09511400 |
| H | -6.97422200 | 1.53020400 | 2.10650700 |
| H | -8.15269800 | 2.43557900 | 1.14103500 |
| C | -7.85478900 | 0.37877300 | 0.50711900 |
| C | -7.82304400 | -0.79342500 | 1.26167300 |
| C | -8.30798600 | 0.33602900 | -0.81399200 |
| C | -8.23876400 | -2.00123200 | 0.70221500 |
| H | -7.46417600 | -0.76441200 | 2.28760900 |
| C | -8.72128800 | -0.86860000 | -1.37335700 |
| H | -8.32401700 | 1.24890600 | -1.40504300 |
| C | -8.68548700 | -2.04016000 | -0.61563700 |
| H | -8.20822000 | -2.90941100 | 1.29578800 |
| H | -9.06921600 | -0.89572800 | -2.40115000 |
| H | -9.00470300 | -2.98036700 | -1.05431100 |

**IN(AZ)**

| | | | |
|---|---|---|---|
| N | -8.15222500 | 0.91136300 | -1.74240500 |
| C | -7.11597300 | -0.00744400 | -1.28913400 |
| C | -5.80174400 | 0.76474400 | -1.10439700 |
| O | -4.79115500 | -0.12909700 | -0.72673400 |
| C | -7.43130700 | -0.68296300 | 0.02504900 |
| O | -7.09717400 | -1.81225200 | 0.31497100 |
| O | -3.22112600 | 0.81435200 | -2.39696400 |
| P | -3.09254500 | 0.42381700 | -0.95376400 |
| C | -2.73021400 | 2.59664300 | 0.69618100 |
| O | -2.66658700 | -1.07852800 | -0.45503200 |
| C | -3.28923300 | -2.25567900 | -0.97712400 |
| C | -2.29389900 | -3.38929700 | -0.82968000 |
| H | -9.03274800 | 0.41615000 | -1.86474600 |
| H | -6.95273000 | -0.78538600 | -2.04140500 |
| H | -5.55783500 | 1.24784000 | -2.05640800 |

| | | | |
|---|---|---|---|
| H | -5.93808000 | 1.55274200 | -0.34494800 |
| H | -7.97199600 | -0.04713800 | 0.75970100 |
| H | -3.24860300 | 2.94305200 | 1.59563600 |
| H | -1.70278200 | 2.34393800 | 0.96563500 |
| H | -4.21303100 | -2.46529600 | -0.42750900 |
| H | -3.55738700 | -2.09775000 | -2.02714700 |
| H | -2.72143000 | -4.32211400 | -1.20817500 |
| H | -2.03295200 | -3.52984300 | 0.22354700 |
| H | -1.37927400 | -3.16913900 | -1.38699600 |
| C | -3.45018000 | -1.25808400 | 3.75580700 |
| N | -4.70380900 | -1.82642100 | 3.81264500 |
| C | -3.34186300 | -0.69714100 | 2.52108100 |
| C | -5.33841600 | -1.62395100 | 2.65791600 |
| N | -4.52753100 | -0.93763000 | 1.86331400 |
| H | -2.53617100 | -0.16348000 | 2.04483700 |
| H | -6.32970500 | -1.96548900 | 2.40148200 |
| H | -4.73584000 | -0.62951600 | 0.87471600 |
| H | -2.76303700 | -1.30441000 | 4.58399900 |
| H | -8.32069400 | 1.62340700 | -1.03238900 |
| H | -5.09282000 | -2.32712200 | 4.60267400 |
| O | -3.44588000 | 1.42187700 | 0.30579400 |
| C | -2.77530800 | 3.66468500 | -0.38104800 |
| H | -3.81255400 | 3.92115000 | -0.61793000 |
| H | -2.26217800 | 4.56967000 | -0.03986000 |
| H | -2.29218600 | 3.30741400 | -1.29490300 |
| C | 2.68046200 | 3.34092200 | 1.06246800 |
| C | 3.29238700 | 2.12213100 | 0.76846500 |
| C | 2.61924300 | 1.19011000 | -0.02076700 |
| C | 1.32815700 | 1.46041200 | -0.51602000 |
| C | 0.74841600 | 2.69440000 | -0.21336100 |
| C | 1.41356400 | 3.63348000 | 0.57080100 |
| H | 3.20976300 | 4.06127200 | 1.67838200 |
| H | 4.28290600 | 1.91577200 | 1.15829900 |
| C | 0.68558400 | 0.46781200 | -1.40019500 |

| | | | |
|---|---|---|---|
| H | -0.23149700 | 2.91322000 | -0.61903000 |
| H | 0.94409300 | 4.58717700 | 0.78938200 |
| O | 3.13627800 | -0.02780600 | -0.35732200 |
| C | 4.83116200 | -1.61385600 | -0.56263400 |
| C | 4.25600000 | -2.36857000 | -1.55850500 |
| N | 5.92266700 | -2.32836800 | -0.20341900 |
| H | 3.35980100 | -2.17356300 | -2.12720800 |
| N | 5.01583400 | -3.47802200 | -1.74951100 |
| N | 6.02280200 | -3.44742200 | -0.93097400 |
| C | 4.46359300 | -0.29816200 | 0.04086800 |
| H | 4.54059900 | -0.33624800 | 1.13665900 |
| H | 5.14165700 | 0.49010000 | -0.31557400 |
| N | -0.55599300 | 0.18583100 | -1.54165900 |
| O | -1.39268600 | 0.87504300 | -0.71109400 |
| H | 1.34169000 | -0.12418900 | -2.03401000 |
| C | 6.91235300 | -2.02553900 | 0.82787000 |
| H | 6.44707400 | -2.14391100 | 1.81098600 |
| H | 7.68334800 | -2.79200400 | 0.71912200 |
| C | 7.47092000 | -0.63126000 | 0.66061400 |
| C | 7.40029000 | 0.28812400 | 1.70612000 |
| C | 8.02645000 | -0.24437400 | -0.56193600 |
| C | 7.87976400 | 1.58644200 | 1.53406800 |
| H | 6.96114500 | -0.00870000 | 2.65549600 |
| C | 8.50192100 | 1.05125600 | -0.73537700 |
| H | 8.07398000 | -0.95962700 | -1.38003800 |
| C | 8.42775500 | 1.96937200 | 0.31292000 |
| H | 7.81753700 | 2.29713600 | 2.35209100 |
| H | 8.92918700 | 1.34726700 | -1.68834000 |
| H | 8.79604600 | 2.98105500 | 0.17524500 |

**TS2(AZ)**

| | | | |
|---|---|---|---|
| N | -7.96094500 | 1.21174500 | -1.83743100 |
| C | -6.98850600 | 0.19297600 | -1.46468900 |
| C | -5.73812000 | 0.87406000 | -0.88982600 |

| | | | |
|---|---|---|---|
| O | -4.77173200 | -0.09775900 | -0.57681000 |
| C | -7.49005800 | -0.78354700 | -0.42343700 |
| O | -7.18166300 | -1.95457400 | -0.37740900 |
| O | -3.14840400 | 0.74531800 | -2.26983000 |
| P | -2.94527400 | 0.41208100 | -0.82636500 |
| C | -2.59586400 | 2.63255900 | 0.71447300 |
| O | -2.63862700 | -1.09177000 | -0.29448200 |
| C | -3.24353400 | -2.23320800 | -0.91889800 |
| C | -2.21434500 | -3.34465800 | -0.92700300 |
| H | -8.79506900 | 0.77630800 | -2.22500400 |
| H | -6.68725300 | -0.37779600 | -2.34854500 |
| H | -5.34677500 | 1.56332800 | -1.64361300 |
| H | -6.00679800 | 1.45768600 | 0.00601500 |
| H | -8.14817800 | -0.33890700 | 0.35527900 |
| H | -3.09033400 | 3.01020400 | 1.61395200 |
| H | -1.55697300 | 2.40384600 | 0.95859400 |
| H | -4.13879600 | -2.51735200 | -0.35620500 |
| H | -3.55628300 | -1.97399200 | -1.93482200 |
| H | -2.63150800 | -4.24310300 | -1.39068900 |
| H | -1.90912800 | -3.59147100 | 0.09377900 |
| H | -1.32943300 | -3.03358200 | -1.48852200 |
| C | -4.45688100 | -1.05606700 | 3.94267100 |
| N | -5.46711900 | -1.92590000 | 3.60397900 |
| C | -4.14179600 | -0.38630700 | 2.79806900 |
| C | -5.74779900 | -1.78285300 | 2.30239400 |
| N | -4.95776400 | -0.85073300 | 1.79189600 |
| H | -3.40554100 | 0.37853600 | 2.61106800 |
| H | -6.50086800 | -2.33741600 | 1.76207900 |
| H | -4.89500700 | -0.49923800 | 0.64708500 |
| H | -4.06482200 | -0.99601200 | 4.94444400 |
| H | -8.26454400 | 1.72342000 | -1.00935600 |
| H | -5.92964300 | -2.57234300 | 4.23070600 |
| O | -3.30642900 | 1.42463100 | 0.40371300 |
| C | -2.69671200 | 3.64583000 | -0.41040400 |

| | | | |
|---|---|---|---|
| H | -3.74551300 | 3.87448000 | -0.62278800 |
| H | -2.18763600 | 4.57255300 | -0.12728300 |
| H | -2.23870900 | 3.25758400 | -1.32494300 |
| C | 2.81511000 | 3.37001500 | 0.92002800 |
| C | 3.41462500 | 2.12950200 | 0.70259100 |
| C | 2.73091500 | 1.15670000 | -0.02669200 |
| C | 1.44080500 | 1.40850500 | -0.53248900 |
| C | 0.87314900 | 2.66510600 | -0.30836300 |
| C | 1.55020300 | 3.64489000 | 0.41256500 |
| H | 3.35226100 | 4.12251900 | 1.48879000 |
| H | 4.40335600 | 1.93744800 | 1.10411100 |
| C | 0.78461300 | 0.36676400 | -1.34768000 |
| H | -0.10591800 | 2.86971800 | -0.72386600 |
| H | 1.09165400 | 4.61538200 | 0.57150600 |
| O | 3.23498800 | -0.08346200 | -0.28994000 |
| C | 4.92687100 | -1.67922700 | -0.43635400 |
| C | 4.33281600 | -2.48022100 | -1.38384300 |
| N | 6.02796400 | -2.37403600 | -0.06784000 |
| H | 3.42387100 | -2.31382300 | -1.94129100 |
| N | 5.09152400 | -3.59574600 | -1.53960800 |
| N | 6.11546600 | -3.52556100 | -0.74497500 |
| C | 4.56629300 | -0.33634400 | 0.10863300 |
| H | 4.65270600 | -0.32106800 | 1.20401500 |
| H | 5.24093600 | 0.43305900 | -0.29204700 |
| N | -0.45787400 | 0.07650600 | -1.44665400 |
| O | -1.26898800 | 0.82114500 | -0.62548400 |
| H | 1.42695800 | -0.25983200 | -1.96194000 |
| C | 7.03903800 | -2.02383500 | 0.92798000 |
| H | 6.59967700 | -2.11605800 | 1.92569000 |
| H | 7.81778700 | -2.78314000 | 0.82456600 |
| C | 7.57231800 | -0.62826500 | 0.70105100 |
| C | 7.50144900 | 0.32830100 | 1.71274000 |
| C | 8.09830900 | -0.27654700 | -0.54492000 |
| C | 7.94937200 | 1.62889800 | 1.48316400 |

| | | | |
|---|---|---|---|
| H | 7.08498900 | 0.05901000 | 2.68035800 |
| C | 8.54210900 | 1.02129800 | -0.77588000 |
| H | 8.14676100 | -1.02094200 | -1.33659000 |
| C | 8.46591600 | 1.97708800 | 0.23814200 |
| H | 7.88558000 | 2.36864000 | 2.27494500 |
| H | 8.94559500 | 1.28959700 | -1.74723300 |
| H | 8.80800100 | 2.99091500 | 0.05544800 |

**C2(AZ)**

| | | | |
|---|---|---|---|
| N | -8.99146400 | -0.48090000 | -1.11724600 |
| C | -7.53212700 | -0.54902400 | -1.07934900 |
| C | -6.99698400 | 0.63602400 | -0.26517500 |
| O | -5.59353200 | 0.71080700 | -0.28714000 |
| C | -7.04682600 | -1.84161600 | -0.45788200 |
| O | -6.23689700 | -2.59388100 | -0.95327300 |
| O | -2.99938100 | 1.59245400 | -2.62972400 |
| P | -2.57635400 | 1.34716300 | -1.24017500 |
| C | -3.11447800 | 2.12110900 | 1.22467600 |
| O | -2.82113600 | -0.10923400 | -0.66485600 |
| C | -3.49122400 | -1.12025800 | -1.45817100 |
| C | -2.52012800 | -1.79160600 | -2.40423700 |
| H | -9.36560700 | -1.25563600 | -1.66124800 |
| H | -7.13421900 | -0.48718100 | -2.09756200 |
| H | -7.40577600 | 1.55126200 | -0.70398200 |
| H | -7.37704000 | 0.56241000 | 0.76717700 |
| H | -7.51306400 | -2.07730600 | 0.52341800 |
| H | -3.29090100 | 1.05573800 | 1.39980400 |
| H | -2.11105500 | 2.38167800 | 1.57527100 |
| H | -3.89303800 | -1.81895200 | -0.72406800 |
| H | -4.33178800 | -0.66012100 | -1.97974300 |
| H | -3.03229700 | -2.59724900 | -2.93865800 |
| H | -1.67663100 | -2.21650700 | -1.85379700 |
| H | -2.13404400 | -1.07481900 | -3.13345600 |
| C | -4.55358900 | -1.57142700 | 3.92941800 |

| | | | |
|---|---|---|---|
| N | -4.57041700 | -2.71465100 | 3.17039800 |
| C | -4.86112000 | -0.55976800 | 3.06169100 |
| C | -4.87917300 | -2.36993500 | 1.90105000 |
| N | -5.06016200 | -1.06785400 | 1.79764700 |
| H | -4.95950300 | 0.49645000 | 3.26874700 |
| H | -4.98180900 | -3.08806300 | 1.09881700 |
| H | -5.26372500 | 0.05910600 | 0.37845200 |
| H | -4.33249000 | -1.58585600 | 4.98450000 |
| H | -9.36861500 | -0.58508300 | -0.17532300 |
| H | -4.38875400 | -3.65288200 | 3.50107000 |
| O | -3.16800200 | 2.37694200 | -0.19712100 |
| C | -4.18349700 | 2.96536900 | 1.87960700 |
| H | -4.13510500 | 2.84909900 | 2.96611800 |
| H | -4.04073600 | 4.02252100 | 1.64068200 |
| H | -5.16710400 | 2.64604000 | 1.52510900 |
| C | 3.33695600 | 3.53455200 | 0.96882700 |
| C | 3.80430500 | 2.24400000 | 0.73025100 |
| C | 3.02459700 | 1.36087700 | -0.01534400 |
| C | 1.77131600 | 1.76236600 | -0.52674900 |
| C | 1.33032900 | 3.06585800 | -0.27223100 |
| C | 2.10657700 | 3.94974600 | 0.47048800 |
| H | 3.94881600 | 4.21575100 | 1.55166800 |
| H | 4.76688800 | 1.94378300 | 1.12729700 |
| C | 1.05259500 | 0.79543800 | -1.37215700 |
| H | 0.38026800 | 3.39044700 | -0.67589300 |
| H | 1.75183400 | 4.95808200 | 0.65348600 |
| O | 3.39170100 | 0.07807900 | -0.28473600 |
| C | 4.85462500 | -1.73875400 | -0.32460400 |
| C | 4.16428200 | -2.51159500 | -1.22920000 |
| N | 5.85210800 | -2.54832100 | 0.09984000 |
| H | 3.28826800 | -2.26413100 | -1.80844500 |
| N | 4.77038900 | -3.72466500 | -1.30705300 |
| N | 5.79084400 | -3.74002100 | -0.50547300 |
| C | 4.67448200 | -0.33361900 | 0.14648000 |

| | | | |
|---|---|---|---|
| H | 4.74732200 | -0.27847200 | 1.24140100 |
| H | 5.44958500 | 0.31981900 | -0.27725500 |
| N | -0.19150500 | 0.65365100 | -1.62286000 |
| O | -0.98942700 | 1.58456000 | -0.92761400 |
| H | 1.65753500 | 0.05136600 | -1.88446500 |
| C | 6.90225200 | -2.26550100 | 1.07583100 |
| H | 6.45184100 | -2.19327700 | 2.07022500 |
| H | 7.55405600 | -3.14213700 | 1.05815200 |
| C | 7.64690600 | -0.99724200 | 0.72812600 |
| C | 7.75116900 | 0.03868300 | 1.65526400 |
| C | 8.20542200 | -0.84374800 | -0.54392500 |
| C | 8.40775500 | 1.22157300 | 1.31628700 |
| H | 7.31110700 | -0.07589600 | 2.64288000 |
| C | 8.85774100 | 0.33671200 | -0.88346800 |
| H | 8.11586800 | -1.64870700 | -1.26988100 |
| C | 8.95864000 | 1.37227300 | 0.04656700 |
| H | 8.48289200 | 2.02415000 | 2.04315700 |
| H | 9.28711300 | 0.45147400 | -1.87371500 |
| H | 9.46522900 | 2.29423300 | -0.22080700 |

**BE**

| | | | |
|---|---|---|---|
| C | -4.49808200 | -2.50980600 | -1.74404200 |
| C | -4.02288900 | -1.20514000 | -1.63884000 |
| C | -3.31831300 | -0.78638000 | -0.51434400 |
| C | -3.05000900 | -1.66702800 | 0.55779900 |
| C | -3.54341800 | -2.97926800 | 0.41911300 |
| C | -4.24997700 | -3.40009700 | -0.70090900 |
| H | -5.05034800 | -2.82212400 | -2.62452700 |
| H | -4.19563700 | -0.47605500 | -2.42561100 |
| C | -2.34130100 | -1.32969000 | 1.78747700 |
| H | -3.36049800 | -3.68101100 | 1.22965500 |
| H | -4.60970700 | -4.42329100 | -0.75658500 |
| H | -2.27401700 | -2.11124800 | 2.55259100 |
| O | -2.92632900 | 0.53774100 | -0.47963800 |

| | | | |
|---|---|---|---|
| N | -1.78922400 | -0.17318000 | 2.02233000 |
| O | -1.19666900 | -0.02619900 | 3.17519800 |
| C | -1.24250500 | 2.17505300 | -0.73000000 |
| C | -1.61819500 | 3.23553700 | -1.52652100 |
| N | -0.49355100 | 2.75697200 | 0.23823900 |
| H | -2.23040300 | 3.22989100 | -2.41658300 |
| N | -1.08737000 | 4.37443600 | -1.02159300 |
| N | -0.39931900 | 4.07413200 | 0.04281900 |
| C | -1.55770600 | 0.71918400 | -0.83163200 |
| H | -1.40466100 | 0.38824500 | -1.86707300 |
| H | -0.91609700 | 0.13633300 | -0.16598700 |
| C | 0.23979100 | 2.14412600 | 1.34573600 |
| H | 0.66858400 | 2.98933900 | 1.89160400 |
| H | -0.44457500 | 1.59895300 | 2.00327800 |
| C | 1.33073100 | 1.22215200 | 0.84745900 |
| C | 2.17230400 | 1.61136200 | -0.19646300 |
| C | 1.50399500 | -0.02726300 | 1.44524500 |
| C | 3.18328500 | 0.76775700 | -0.64410300 |
| H | 2.03589900 | 2.58173900 | -0.66842600 |
| C | 2.51684400 | -0.87982000 | 1.00929700 |
| H | 0.82217400 | -0.32427800 | 2.24117500 |
| C | 3.34215400 | -0.47117400 | -0.03105500 |
| H | 3.83688900 | 1.06505200 | -1.45659800 |
| H | 2.65509200 | -1.85462700 | 1.46443700 |
| Br | 4.70735100 | -1.63808000 | -0.64753400 |

**C1(BE)**

| | | | |
|---|---|---|---|
| N | -6.97349500 | -2.29515100 | -1.97453400 |
| C | -5.74738400 | -2.09399100 | -1.22312000 |
| C | -5.04191000 | -0.86298700 | -1.81857200 |
| O | -3.87538100 | -0.48041400 | -1.08126200 |
| C | -5.94332000 | -1.95360300 | 0.27983700 |
| O | -5.03711900 | -1.81135900 | 1.07299400 |
| O | -2.72609200 | -2.82839200 | -1.22615000 |

| | | | |
|---|---|---|---|
| P | -2.52199700 | -1.36817500 | -1.25272100 |
| C | -1.56000000 | 0.51044900 | -2.83983900 |
| O | -1.70793300 | -0.71663500 | -0.05601400 |
| C | -1.17488600 | -1.62149300 | 0.95051300 |
| C | -0.29235400 | -0.82770600 | 1.88083500 |
| H | -7.36999900 | -3.20883000 | -1.77116500 |
| H | -5.08706000 | -2.95604900 | -1.36739900 |
| H | -4.78426100 | -1.07484500 | -2.86076700 |
| H | -5.70624600 | 0.00401900 | -1.79560600 |
| H | -6.99413300 | -2.00058200 | 0.63075900 |
| H | -2.40262800 | 0.99185500 | -3.34446000 |
| H | -1.40271800 | 0.99658000 | -1.87342000 |
| H | -2.02461500 | -2.06869400 | 1.47811500 |
| H | -0.60618900 | -2.39842900 | 0.44002500 |
| H | 0.04636600 | -1.47734100 | 2.69342800 |
| H | -0.83268300 | 0.02031400 | 2.31765700 |
| H | 0.58440100 | -0.45818000 | 1.34204800 |
| C | -2.82951100 | 2.09922700 | 2.96751400 |
| N | -3.23530700 | 0.98355500 | 3.66508400 |
| C | -3.13269900 | 1.86244100 | 1.66118900 |
| C | -3.76116300 | 0.09201800 | 2.82592600 |
| N | -3.70588800 | 0.61417700 | 1.60729500 |
| H | -2.99682000 | 2.47317400 | 0.78291300 |
| H | -4.16651900 | -0.87487700 | 3.07941800 |
| H | -3.97749000 | 0.11874400 | 0.75090600 |
| H | -2.36348400 | 2.94384200 | 3.44742400 |
| H | -7.67413200 | -1.60454400 | -1.70973100 |
| H | -3.15348800 | 0.84992000 | 4.66673700 |
| O | -1.91763700 | -0.87585000 | -2.62795300 |
| C | -0.29567900 | 0.53338000 | -3.66932100 |
| H | -0.47878800 | 0.11087500 | -4.66143100 |
| H | 0.05595600 | 1.56323500 | -3.78597600 |
| H | 0.46520300 | -0.06281800 | -3.15581200 |
| C | 5.07453100 | -4.17276700 | 2.56259200 |

| | | | |
|---|---|---|---|
| C | 5.27111900 | -3.11114600 | 1.68452300 |
| C | 4.26259700 | -2.70498000 | 0.81427100 |
| C | 3.00417700 | -3.34073700 | 0.80214700 |
| C | 2.84268400 | -4.42259900 | 1.68850100 |
| C | 3.84711800 | -4.83352100 | 2.55604400 |
| H | 5.86999400 | -4.48285900 | 3.23259700 |
| H | 6.22140200 | -2.58555900 | 1.64073500 |
| C | 1.89079300 | -2.99975900 | -0.08081800 |
| H | 1.88776100 | -4.94319800 | 1.68970500 |
| H | 3.67134100 | -5.66987800 | 3.22602600 |
| H | 1.11341600 | -3.75940200 | -0.22155700 |
| O | 4.56838500 | -1.69480200 | -0.07786800 |
| N | 1.75599600 | -1.84610400 | -0.66563600 |
| O | 0.69441100 | -1.68142800 | -1.41094900 |
| C | 4.97791900 | 0.59425900 | -0.47859500 |
| C | 6.23677800 | 1.15371700 | -0.50248900 |
| N | 4.39003400 | 1.09450900 | -1.59069900 |
| H | 7.05158000 | 1.02090100 | 0.19411400 |
| N | 6.33998300 | 1.95256400 | -1.59220700 |
| N | 5.21206900 | 1.91524000 | -2.24467200 |
| C | 4.33042500 | -0.38772700 | 0.43867600 |
| H | 4.78037400 | -0.29128000 | 1.43374700 |
| H | 3.24931700 | -0.21695300 | 0.50824200 |
| C | 2.99987200 | 0.94674000 | -2.00971100 |
| H | 2.97704400 | 1.19787300 | -3.07360000 |
| H | 2.70433000 | -0.09864800 | -1.87155200 |
| C | 2.09090600 | 1.84876100 | -1.20170000 |
| C | 2.41055300 | 3.19057700 | -0.97847500 |
| C | 0.91905900 | 1.31803300 | -0.66561200 |
| C | 1.56479400 | 4.00481000 | -0.23039700 |
| H | 3.32961900 | 3.60481000 | -1.38589700 |
| C | 0.06901000 | 2.12332200 | 0.08958800 |
| H | 0.66456300 | 0.26981800 | -0.85163900 |
| C | 0.39937800 | 3.45442000 | 0.29511700 |

| | | | |
|---|---|---|---|
| H | 1.80768500 | 5.04704900 | -0.05479700 |
| H | -0.83247100 | 1.69607500 | 0.51472000 |
| Br | -0.76418100 | 4.54946200 | 1.32970700 |

**TS1(BE)**

| | | | |
|---|---|---|---|
| N | 6.01958600 | -3.48455500 | 0.58585200 |
| C | 4.89020900 | -2.69909700 | 0.09455500 |
| C | 4.46372000 | -1.67924500 | 1.13940600 |
| O | 3.34376500 | -0.95224000 | 0.65984400 |
| C | 5.22827600 | -2.04344600 | -1.23263300 |
| O | 5.14300400 | -0.85686700 | -1.47786900 |
| O | 1.85578600 | -2.85312900 | 1.36223000 |
| P | 1.81055900 | -1.38644100 | 1.13396900 |
| C | 0.99518200 | -0.33291100 | 3.54477600 |
| O | 1.11552700 | -0.72321800 | -0.15319300 |
| C | 1.48286800 | -1.20611400 | -1.45751500 |
| C | 0.45042300 | -0.70017700 | -2.44109000 |
| H | 6.18272600 | -4.29297400 | -0.00972200 |
| H | 4.04382800 | -3.37744700 | -0.07447500 |
| H | 4.23987100 | -2.19805600 | 2.07519600 |
| H | 5.26995100 | -0.95884500 | 1.31999700 |
| H | 5.63589800 | -2.73321300 | -1.99665600 |
| H | 1.44234800 | 0.47603100 | 4.12761600 |
| H | -0.04004100 | -0.09337000 | 3.31017200 |
| H | 2.48588600 | -0.84326900 | -1.70420600 |
| H | 1.50806500 | -2.30248400 | -1.44345300 |
| H | 0.68368500 | -1.06688100 | -3.44457800 |
| H | 0.44438200 | 0.39322800 | -2.46360100 |
| H | -0.54509400 | -1.05265200 | -2.15681200 |
| C | 2.96854100 | 3.37364800 | 0.22312300 |
| N | 3.49860200 | 3.53799800 | -1.03759400 |
| C | 3.15596900 | 2.06574400 | 0.55175400 |
| C | 3.99896300 | 2.38128600 | -1.46871700 |
| N | 3.79565700 | 1.48077000 | -0.51630700 |

| | | | |
|---|---|---|---|
| H | 2.87378900 | 1.49067900 | 1.42140100 |
| H | 4.47906500 | 2.20929800 | -2.41861700 |
| H | 4.07244000 | 0.49140100 | -0.56320800 |
| H | 2.48897000 | 4.18331000 | 0.74821800 |
| H | 6.87372600 | -2.92959500 | 0.56986100 |
| H | 3.49394900 | 4.40064100 | -1.56986900 |
| O | 1.75145600 | -0.31575400 | 2.31679800 |
| C | 1.10418900 | -1.65241500 | 4.27963500 |
| H | 2.15309000 | -1.91973800 | 4.44185300 |
| H | 0.61562500 | -1.56237200 | 5.25455400 |
| H | 0.61578200 | -2.43946100 | 3.70488500 |
| C | -3.63492300 | -4.34820000 | -3.14303000 |
| C | -4.18718700 | -3.48514200 | -2.20058600 |
| C | -3.40798800 | -2.94284300 | -1.18183400 |
| C | -2.03436100 | -3.24616700 | -1.07464900 |
| C | -1.50569800 | -4.12026300 | -2.04179800 |
| C | -2.28022500 | -4.66338200 | -3.05985600 |
| H | -4.25489900 | -4.76574300 | -3.92975100 |
| H | -5.23976900 | -3.21826600 | -2.22918700 |
| C | -1.11756300 | -2.75388700 | -0.04269800 |
| H | -0.44959400 | -4.37311500 | -1.97867400 |
| H | -0.11509700 | -3.18910000 | -0.02773700 |
| O | -4.04894300 | -2.12723600 | -0.27071700 |
| N | -1.44108300 | -1.85145300 | 0.82489200 |
| O | -0.52230800 | -1.47297800 | 1.69313200 |
| C | -4.77581100 | 0.03619100 | 0.36093500 |
| C | -6.13253000 | 0.27921800 | 0.37141500 |
| N | -4.33555500 | 0.66291000 | 1.47913400 |
| H | -6.88404900 | -0.04246700 | -0.33472900 |
| N | -6.43631100 | 1.02769500 | 1.45852000 |
| N | -5.33881800 | 1.26177600 | 2.12120500 |
| C | -3.91850900 | -0.74102300 | -0.58202600 |
| H | -4.26705700 | -0.56495500 | -1.60692000 |
| H | -2.86946700 | -0.43876700 | -0.49832700 |

| | | | |
|---|---|---|---|
| C | -2.96467900 | 0.82847300 | 1.95950100 |
| H | -3.06218500 | 1.17146800 | 2.99253000 |
| H | -2.45948400 | -0.14152200 | 1.94209700 |
| C | -2.19989800 | 1.82453700 | 1.11473900 |
| C | -2.70016700 | 3.10588000 | 0.87029000 |
| C | -0.97696700 | 1.44302300 | 0.56642800 |
| C | -1.97891500 | 4.00814700 | 0.09374400 |
| H | -3.65896600 | 3.40350500 | 1.28828500 |
| C | -0.25526200 | 2.32929400 | -0.22974900 |
| H | -0.58578500 | 0.44933200 | 0.77137100 |
| C | -0.76109200 | 3.60324000 | -0.44693000 |
| H | -2.35562100 | 5.00784200 | -0.09210700 |
| H | 0.68614900 | 2.01945500 | -0.67030200 |
| Br | 0.23675400 | 4.82621200 | -1.50959000 |
| H | -1.82711900 | -5.33351900 | -3.78382000 |

**IN(BE)**

| | | | |
|---|---|---|---|
| N | 6.74763100 | -2.42827700 | 1.46467300 |
| C | 5.76150700 | -1.88263400 | 0.54194800 |
| C | 4.43502500 | -1.67452300 | 1.28753600 |
| O | 3.46822000 | -1.20849200 | 0.38201000 |
| C | 6.15757500 | -0.55629000 | -0.06692400 |
| O | 5.82942500 | -0.18697700 | -1.17452300 |
| O | 1.85473200 | -2.93671800 | 1.09211400 |
| P | 1.75899500 | -1.46587900 | 0.80175500 |
| C | 1.14202200 | 0.14791700 | 2.96474900 |
| O | 1.40976900 | -0.85923800 | -0.68469300 |
| C | 2.07589600 | -1.33352100 | -1.86022500 |
| C | 1.14573600 | -1.08139100 | -3.02935200 |
| H | 7.63023900 | -2.58576800 | 0.98326800 |
| H | 5.58307800 | -2.59062800 | -0.27359200 |
| H | 4.13409900 | -2.63596100 | 1.71452900 |
| H | 4.57764900 | -0.95699100 | 2.11182800 |
| H | 6.75307600 | 0.10700500 | 0.59695400 |

| | | | |
|---|---|---|---|
| H | 1.67562200 | 0.99065600 | 3.41461800 |
| H | 0.20275900 | 0.51623000 | 2.54582300 |
| H | 3.03108300 | -0.81257800 | -1.98393000 |
| H | 2.29516800 | -2.40310700 | -1.75319100 |
| H | 1.61008900 | -1.41236500 | -3.96260900 |
| H | 0.92191600 | -0.01335700 | -3.11178600 |
| H | 0.20395600 | -1.62314700 | -2.89422400 |
| C | 2.30218600 | 3.33401100 | -0.59561800 |
| N | 3.56539800 | 3.38897400 | -1.14292200 |
| C | 2.17400400 | 2.08712000 | -0.06496900 |
| C | 4.18520000 | 2.22318000 | -0.95831000 |
| N | 3.35472500 | 1.42272200 | -0.30242700 |
| H | 1.35490100 | 1.60589400 | 0.44408400 |
| H | 5.17823900 | 1.96334700 | -1.29133800 |
| H | 3.52726400 | 0.41832500 | -0.03498300 |
| H | 1.61121300 | 4.16072000 | -0.65895100 |
| H | 6.94181500 | -1.75523200 | 2.20538200 |
| H | 3.97032500 | 4.18575800 | -1.61986800 |
| O | 2.00932600 | -0.28508500 | 1.90898400 |
| C | 0.90456100 | -0.93809600 | 3.99598800 |
| H | 1.85937100 | -1.30742700 | 4.38264900 |
| H | 0.32465500 | -0.53729800 | 4.83306300 |
| H | 0.35448500 | -1.77038700 | 3.55219400 |
| C | -3.58850700 | -4.85210500 | -2.83922700 |
| C | -4.04186200 | -4.03068300 | -1.81127200 |
| C | -3.14268600 | -3.34371800 | -0.99988800 |
| C | -1.75539900 | -3.46394800 | -1.20543200 |
| C | -1.32414300 | -4.30555500 | -2.24176600 |
| C | -2.21956000 | -4.98871000 | -3.05611000 |
| H | -4.30096200 | -5.38319400 | -3.46230300 |
| H | -5.10274200 | -3.91229300 | -1.61102900 |
| C | -0.70432900 | -2.80503100 | -0.41018500 |
| H | -0.25449400 | -4.41706500 | -2.40434000 |
| H | -1.85001100 | -5.62872200 | -3.85054900 |

| | | | |
|---|---|---|---|
| H | 0.31085400 | -3.16108800 | -0.57956600 |
| O | -3.66315900 | -2.58035800 | 0.02250100 |
| N | -0.98189100 | -1.88443900 | 0.43408200 |
| O | 0.00650400 | -1.27470900 | 1.14033100 |
| C | -4.66057200 | -0.54534600 | 0.73079100 |
| C | -6.00425300 | -0.44142700 | 1.01820900 |
| N | -4.06874400 | 0.13028400 | 1.74580200 |
| H | -6.84724300 | -0.84194400 | 0.47416000 |
| N | -6.15436800 | 0.27779900 | 2.15619700 |
| N | -4.97470500 | 0.62497000 | 2.58735500 |
| C | -3.93228300 | -1.23487500 | -0.37354800 |
| H | -4.56538900 | -1.23480200 | -1.26818800 |
| H | -2.99142100 | -0.72401400 | -0.60584000 |
| C | -2.65056200 | 0.43302200 | 1.93883500 |
| H | -2.53802400 | 0.65054400 | 3.00363000 |
| H | -2.07686600 | -0.45991900 | 1.68751200 |
| C | -2.22402000 | 1.61246800 | 1.09124000 |
| C | -2.71646900 | 2.88837400 | 1.37934800 |
| C | -1.35937800 | 1.43904800 | 0.00981300 |
| C | -2.34358300 | 3.98566000 | 0.61173100 |
| H | -3.39741800 | 3.02445200 | 2.21584900 |
| C | -0.99771500 | 2.52705400 | -0.78498400 |
| H | -0.94829600 | 0.45569300 | -0.20901400 |
| C | -1.48286100 | 3.78720100 | -0.46403000 |
| H | -2.71142300 | 4.97905100 | 0.84350300 |
| H | -0.33443000 | 2.39354500 | -1.63342100 |
| Br | -0.94474600 | 5.27741100 | -1.50771000 |

**TS2(BE)**

| | | | |
|---|---|---|---|
| N | 6.05431800 | -3.57069600 | 0.80428600 |
| C | 4.98083200 | -2.94827400 | 0.04365200 |
| C | 3.94085100 | -2.37752900 | 1.01913700 |
| O | 2.90734400 | -1.76483600 | 0.28115100 |
| C | 5.43421700 | -1.82478600 | -0.87117400 |

| | | | |
|---|---|---|---|
| O | 4.89164800 | -1.53722300 | -1.91486300 |
| O | 1.14359600 | -3.35228500 | 1.05988700 |
| P | 1.12093100 | -1.86182300 | 0.92812300 |
| C | 1.08107100 | -0.45423400 | 3.27918000 |
| O | 0.77113600 | -1.12935500 | -0.48240300 |
| C | 1.18814300 | -1.66712400 | -1.74675400 |
| C | 0.17110400 | -1.21723700 | -2.77503200 |
| H | 6.72686200 | -4.00326500 | 0.17511100 |
| H | 4.47960900 | -3.69950600 | -0.57528300 |
| H | 3.53189000 | -3.19712100 | 1.61519600 |
| H | 4.41091700 | -1.64808700 | 1.69661200 |
| H | 6.30127900 | -1.23646500 | -0.49992600 |
| H | 1.78186500 | 0.26060800 | 3.71973400 |
| H | 0.14803900 | 0.06390300 | 3.05304600 |
| H | 2.19352100 | -1.30612500 | -1.98236600 |
| H | 1.23451900 | -2.76034700 | -1.67946100 |
| H | 0.45717000 | -1.57494700 | -3.76805500 |
| H | 0.11518700 | -0.12468000 | -2.80118500 |
| H | -0.82211400 | -1.60927100 | -2.53411900 |
| C | 3.46521200 | 2.83046600 | 0.29294900 |
| N | 3.78137900 | 2.54847900 | -1.01488000 |
| C | 3.22958500 | 1.62418200 | 0.88486800 |
| C | 3.74132400 | 1.22006900 | -1.19452700 |
| N | 3.40769400 | 0.63593200 | -0.05485000 |
| H | 2.93963000 | 1.39152000 | 1.89666300 |
| H | 3.97231300 | 0.71257100 | -2.11928200 |
| H | 3.16601000 | -0.58736500 | 0.10381400 |
| H | 3.41439500 | 3.84158100 | 0.66283300 |
| H | 6.56660700 | -2.86290300 | 1.32965600 |
| H | 3.98819300 | 3.23092700 | -1.73363100 |
| O | 1.72146900 | -0.85325100 | 2.05488200 |
| C | 0.86459700 | -1.62546500 | 4.21695400 |
| H | 1.81050600 | -2.14434700 | 4.39854000 |
| H | 0.47762900 | -1.26558200 | 5.17474200 |

| | | | |
|---|---|---|---|
| H | 0.14762900 | -2.33204500 | 3.79336800 |
| C | -4.92027400 | -3.95020000 | -2.49468500 |
| C | -5.10234500 | -2.88510600 | -1.61751500 |
| C | -4.05670300 | -2.44048300 | -0.81343000 |
| C | -2.79612700 | -3.06241700 | -0.87009000 |
| C | -2.63597100 | -4.13002500 | -1.76594000 |
| C | -3.67722300 | -4.57354400 | -2.57247600 |
| H | -5.74514500 | -4.28671400 | -3.11447000 |
| H | -6.05942700 | -2.37894200 | -1.53495200 |
| C | -1.62098800 | -2.70131400 | -0.06006200 |
| H | -1.66555400 | -4.61708200 | -1.82283700 |
| H | -3.51885300 | -5.40156200 | -3.25534300 |
| H | -0.73396600 | -3.31907400 | -0.20872900 |
| O | -4.31544300 | -1.39534200 | 0.04368500 |
| N | -1.63886900 | -1.74158600 | 0.78534000 |
| O | -0.48855300 | -1.47204100 | 1.48828000 |
| C | -4.57599100 | 0.93734300 | 0.35479800 |
| C | -5.81495700 | 1.53991100 | 0.35388600 |
| N | -3.95394700 | 1.49658900 | 1.42013400 |
| H | -6.64358900 | 1.38483800 | -0.32151100 |
| N | -5.87500200 | 2.41774700 | 1.38362900 |
| N | -4.73949800 | 2.38880200 | 2.02236400 |
| C | -3.98752300 | -0.12111500 | -0.51507200 |
| H | -4.42302700 | -0.03688200 | -1.51696600 |
| H | -2.89832000 | -0.01732700 | -0.59241600 |
| C | -2.56908100 | 1.32786800 | 1.85522600 |
| H | -2.55282600 | 1.61022700 | 2.91046600 |
| H | -2.31082400 | 0.26934900 | 1.76123300 |
| C | -1.63092500 | 2.18774700 | 1.03559900 |
| C | -1.77579000 | 3.57720800 | 1.01305600 |
| C | -0.62289200 | 1.59007400 | 0.28234500 |
| C | -0.91995500 | 4.36240000 | 0.24843700 |
| H | -2.56609900 | 4.04913100 | 1.59153500 |
| C | 0.24056000 | 2.36297600 | -0.49261900 |

| | | | |
|---|---|---|---|
| H | -0.50149600 | 0.51229700 | 0.29523000 |
| C | 0.07884700 | 3.74158600 | -0.49793800 |
| H | -1.02635700 | 5.44137200 | 0.22748700 |
| H | 1.02268600 | 1.88916000 | -1.07999100 |
| Br | 1.23449900 | 4.81569800 | -1.55458100 |

**C2(BE)**

| | | | |
|---|---|---|---|
| N | 2.53026200 | 6.02528900 | -0.00863400 |
| C | 2.11389000 | 4.77383300 | 0.62429100 |
| C | 2.95572500 | 3.62719600 | 0.07901100 |
| O | 2.66729400 | 2.47441600 | 0.83462700 |
| C | 2.27815300 | 4.82122400 | 2.12927100 |
| O | 1.38225300 | 4.68319700 | 2.93103700 |
| O | 0.02490400 | 2.87430600 | -0.86025400 |
| P | 0.25637400 | 1.42277100 | -0.74079200 |
| C | 1.98121700 | -0.47776800 | -1.19249000 |
| O | 0.18306000 | 0.80479900 | 0.71754200 |
| C | 0.01970300 | 1.65501200 | 1.87945700 |
| C | -1.42022500 | 1.60877300 | 2.34179500 |
| H | 1.94222200 | 6.79141700 | 0.31247200 |
| H | 1.06198600 | 4.58175400 | 0.39586600 |
| H | 2.72446500 | 3.49527700 | -0.98675400 |
| H | 4.02293100 | 3.89548500 | 0.16686900 |
| H | 3.31436500 | 5.04035300 | 2.47151200 |
| H | 2.40686000 | -0.52253400 | -0.18544200 |
| H | 1.09504400 | -1.11755300 | -1.23300600 |
| H | 0.70362100 | 1.25560100 | 2.63056500 |
| H | 0.33749000 | 2.66822300 | 1.63516200 |
| H | -1.54233500 | 2.23607800 | 3.22986500 |
| H | -1.71581800 | 0.58728000 | 2.59884100 |
| H | -2.08822800 | 1.98254400 | 1.55834700 |
| C | 6.01122600 | -0.98059800 | -0.36169200 |
| N | 6.41169100 | 0.05299200 | -1.17084700 |
| C | 5.02933300 | -0.45507000 | 0.43310300 |

| | | | |
|---|---|---|---|
| C | 5.67310500 | 1.14058400 | -0.85313100 |
| N | 4.82340400 | 0.86678300 | 0.11630500 |
| H | 4.45046000 | -0.95425900 | 1.19620800 |
| H | 5.79002000 | 2.09360400 | -1.34865600 |
| H | 3.37539500 | 1.81903700 | 0.61875800 |
| H | 6.44709800 | -1.96486900 | -0.42794400 |
| H | 3.48276300 | 6.25714000 | 0.27356600 |
| H | 7.13092100 | 0.01302500 | -1.88063900 |
| O | 1.57472900 | 0.89206100 | -1.43919700 |
| C | 2.99391400 | -0.87983500 | -2.23709900 |
| H | 3.37608800 | -1.87463900 | -1.98694200 |
| H | 2.54029900 | -0.90845500 | -3.23135800 |
| H | 3.83434800 | -0.17992100 | -2.24511900 |
| C | -6.83952600 | 2.54759500 | -0.58915600 |
| C | -6.39668100 | 1.25414200 | -0.32836000 |
| C | -5.08711900 | 0.88632400 | -0.62273700 |
| C | -4.20001000 | 1.81983400 | -1.18642500 |
| C | -4.67259700 | 3.10915700 | -1.46560900 |
| C | -5.97614000 | 3.47986300 | -1.16130600 |
| H | -7.86326000 | 2.82144500 | -0.35532600 |
| H | -7.06047200 | 0.50568400 | 0.09300900 |
| C | -2.80323700 | 1.53435500 | -1.53617500 |
| H | -3.99578400 | 3.82698700 | -1.92059500 |
| H | -6.31857100 | 4.48593200 | -1.37759900 |
| H | -2.32118500 | 2.19579400 | -2.25840800 |
| O | -4.71599700 | -0.42067500 | -0.41590400 |
| N | -2.14094500 | 0.58040200 | -1.00408900 |
| O | -0.83040700 | 0.51744400 | -1.55947000 |
| C | -4.16238800 | -2.17075200 | 1.08036300 |
| C | -4.77379500 | -3.01467200 | 1.97963000 |
| N | -3.42371000 | -3.00678100 | 0.31069200 |
| H | -5.45684600 | -2.77433300 | 2.78083300 |
| N | -4.38147200 | -4.28657500 | 1.71778800 |
| N | -3.56761300 | -4.27292900 | 0.70336500 |

| | | | |
|---|---|---|---|
| C | -4.22175200 | -0.69264900 | 0.89907200 |
| H | -4.89571600 | -0.27220500 | 1.65308800 |
| H | -3.23157900 | -0.23598000 | 1.00808300 |
| C | -2.54066600 | -2.68727400 | -0.80801300 |
| H | -2.85271200 | -3.29258000 | -1.66191100 |
| H | -2.70824400 | -1.63371800 | -1.04569000 |
| C | -1.09052000 | -2.94490500 | -0.47138400 |
| C | -0.29141200 | -3.68769300 | -1.33906900 |
| C | -0.52971300 | -2.42873700 | 0.69960600 |
| C | 1.05401600 | -3.91149400 | -1.05381600 |
| H | -0.71705900 | -4.09849800 | -2.25015400 |
| C | 0.80677400 | -2.65537200 | 1.00833400 |
| H | -1.13743200 | -1.83676800 | 1.37959200 |
| C | 1.58495600 | -3.38991200 | 0.11875300 |
| H | 1.67805100 | -4.48243900 | -1.73257600 |
| H | 1.24442700 | -2.24484200 | 1.91194800 |
| Br | 3.43072500 | -3.63160700 | 0.47454200 |

**BZ**

| | | | |
|---|---|---|---|
| C | 1.97615200 | -3.05047700 | 0.95239500 |
| C | 1.57621400 | -1.72261500 | 0.76761800 |
| C | 2.48464800 | -0.78594800 | 0.28890600 |
| C | 3.82947400 | -1.13076500 | -0.01882400 |
| C | 4.19177100 | -2.47520400 | 0.18129600 |
| C | 3.28029000 | -3.41997500 | 0.65770500 |
| H | 1.26026900 | -3.77595900 | 1.32631000 |
| H | 0.55539900 | -1.44179700 | 1.00316600 |
| C | 4.73214100 | -0.09439200 | -0.51075000 |
| H | 5.21219100 | -2.74369300 | -0.05253900 |
| H | 3.60035900 | -4.44883100 | 0.79750300 |
| O | 2.15076100 | 0.53212700 | 0.08372600 |
| C | 0.70815600 | 2.35468300 | -0.00678100 |
| C | 1.47618200 | 3.16490500 | -0.81072700 |
| N | -0.30127000 | 3.16116600 | 0.39406000 |

| | | | |
|---|---|---|---|
| H | 2.38543200 | 2.92791400 | -1.34138200 |
| N | 0.90161200 | 4.39505300 | -0.85598000 |
| N | -0.17323500 | 4.38718100 | -0.12903100 |
| C | 0.82908000 | 0.91513300 | 0.37309500 |
| H | 0.60058900 | 0.77055200 | 1.43970700 |
| H | 0.11164000 | 0.31092900 | -0.20182900 |
| N | 6.00011400 | -0.18430400 | -0.85216900 |
| O | 6.61206900 | -1.32531100 | -0.76957300 |
| H | 4.31923700 | 0.90391200 | -0.61444800 |
| C | -1.41625500 | 2.85080000 | 1.28294100 |
| H | -1.03153900 | 2.69636200 | 2.29528100 |
| H | -2.04077600 | 3.74722500 | 1.28769900 |
| C | -2.18130400 | 1.63681300 | 0.80953800 |
| C | -2.39009400 | 0.55622900 | 1.66448600 |
| C | -2.66478000 | 1.57893200 | -0.49997700 |
| C | -3.07825800 | -0.57336000 | 1.22699700 |
| H | -2.01050500 | 0.58767500 | 2.68224200 |
| C | -3.35099500 | 0.45797400 | -0.95269000 |
| H | -2.49786600 | 2.41453100 | -1.17537400 |
| C | -3.55053400 | -0.60709500 | -0.07866000 |
| H | -3.24120200 | -1.41440400 | 1.89153700 |
| H | -3.72747300 | 0.41049100 | -1.96832600 |
| Br | -4.49429300 | -2.13848300 | -0.67829500 |

**C1(BZ)**

| | | | |
|---|---|---|---|
| N | 8.94281800 | -2.57972000 | -1.92425400 |
| C | 7.82776700 | -1.68150000 | -1.62786100 |
| C | 8.25232100 | -0.23635500 | -1.80835700 |
| O | 8.43273500 | 0.54259400 | -0.89595100 |
| C | 7.35616800 | -1.97314300 | -0.20695900 |
| O | 6.40577500 | -1.01602700 | 0.27868600 |
| C | 3.59913300 | -2.69711500 | 0.60767500 |
| C | 2.70470300 | -3.80712900 | 0.10765100 |
| C | 3.59397000 | 1.33384600 | 0.24964100 |

| | | | |
|---|---|---|---|
| C | 2.47718100 | 1.46582500 | 1.25699700 |
| O | 4.34517800 | -2.20451100 | -0.52985800 |
| O | 4.35085500 | 0.14256200 | 0.59350000 |
| O | 5.19477800 | -0.19374000 | -1.86780300 |
| P | 5.00904300 | -0.76849000 | -0.51899800 |
| H | 6.92543400 | -2.97720500 | -0.16031700 |
| H | 8.20075600 | -1.91668200 | 0.48377600 |
| H | 9.14636700 | -2.58855000 | -2.92053100 |
| H | 9.78826400 | -2.27036300 | -1.44744000 |
| H | 7.02915600 | -1.88620000 | -2.34739300 |
| H | 8.48285600 | 0.05726900 | -2.84961200 |
| H | 4.31596500 | -3.04746200 | 1.35844700 |
| H | 3.01090900 | -1.86973100 | 1.00868300 |
| H | 2.02676700 | -3.39682000 | -0.64477600 |
| H | 3.29310600 | -4.61845800 | -0.32975800 |
| H | 2.11523100 | -4.21104900 | 0.93650000 |
| H | 1.83206600 | 0.58558700 | 1.18037700 |
| H | 2.87521800 | 1.55236200 | 2.27293100 |
| H | 1.88705600 | 2.36018500 | 1.03717200 |
| H | 3.20024300 | 1.23256600 | -0.76039700 |
| H | 4.29447100 | 2.17474700 | 0.29791900 |
| C | 6.20208700 | 1.81979500 | 2.37065300 |
| N | 7.00921000 | 1.55342800 | 1.28898800 |
| C | 6.36917100 | 3.13721000 | 2.66724800 |
| C | 7.65206400 | 2.65521700 | 0.92530700 |
| N | 7.27471000 | 3.62842800 | 1.75208700 |
| H | 5.93313600 | 3.75936500 | 3.43082100 |
| H | 8.34772500 | 2.73446400 | 0.10516100 |
| H | 7.60887700 | 4.58418800 | 1.70660000 |
| H | 5.57967500 | 1.06017000 | 2.81456000 |
| H | 7.09749600 | 0.64808200 | 0.81072300 |
| C | -2.17733500 | -1.90129300 | 2.14802400 |
| C | -2.78164400 | -0.83680900 | 1.47027700 |
| C | -2.07426500 | -0.14774500 | 0.49165600 |

| | | | |
|---|---|---|---|
| C | -0.74068400 | -0.49636000 | 0.14568100 |
| C | -0.16913900 | -1.56999900 | 0.85096400 |
| C | -0.87494100 | -2.26183400 | 1.83772300 |
| H | -2.73685000 | -2.43289100 | 2.91154600 |
| H | -3.80116000 | -0.56339300 | 1.71934100 |
| C | -0.05840700 | 0.25970700 | -0.90320100 |
| H | 0.84408600 | -1.84016300 | 0.59053100 |
| H | -0.39650400 | -3.08672000 | 2.35852500 |
| O | -2.60799700 | 0.91167800 | -0.20081600 |
| C | -4.29021600 | 2.38697000 | -0.83906100 |
| C | -3.67150400 | 2.90759100 | -1.95182400 |
| N | -5.40118400 | 3.14592800 | -0.70149500 |
| H | -2.74738500 | 2.60486100 | -2.41993600 |
| N | -4.42865100 | 3.93193300 | -2.42417900 |
| N | -5.47513500 | 4.06918600 | -1.66896100 |
| C | -3.94672800 | 1.25063900 | 0.06698700 |
| H | -4.07774600 | 1.54331900 | 1.11969500 |
| H | -4.61020500 | 0.39413200 | -0.12369600 |
| N | 1.15842900 | 0.12155000 | -1.37094500 |
| O | 1.93498600 | -0.80481000 | -0.87703900 |
| H | -0.62158000 | 1.05902600 | -1.37436400 |
| C | -6.45551200 | 3.03077100 | 0.29982300 |
| H | -6.03448800 | 3.23981600 | 1.28756100 |
| H | -7.17311400 | 3.81883900 | 0.05943700 |
| C | -7.09075200 | 1.65953700 | 0.26166900 |
| C | -7.17798200 | 0.88015700 | 1.41342600 |
| C | -7.56443800 | 1.14986000 | -0.95005300 |
| C | -7.72957900 | -0.39923600 | 1.36556800 |
| H | -6.80439500 | 1.26411400 | 2.35895400 |
| C | -8.11384300 | -0.12420000 | -1.01489900 |
| H | -7.49296700 | 1.75030100 | -1.85373400 |
| C | -8.18671800 | -0.88652700 | 0.14818300 |
| H | -7.79042000 | -1.00916900 | 2.25981100 |
| H | -8.47749400 | -0.52438300 | -1.95454700 |

| | | | |
|---|---|---|---|
| Br | -8.90866700 | -2.63660600 | 0.05429500 |

**TS1(BZ)**

| | | | |
|---|---|---|---|
| N | 8.20442600 | -3.63073500 | -0.64570400 |
| C | 7.40611100 | -2.42615700 | -0.80858800 |
| C | 6.59383100 | -2.20859500 | 0.48244200 |
| O | 5.83228400 | -1.01431600 | 0.44323300 |
| C | 8.20995100 | -1.17713200 | -1.11035700 |
| O | 7.72788000 | -0.15112500 | -1.54356200 |
| O | 4.45915900 | -1.86505200 | -1.50724900 |
| P | 4.31153300 | -1.05946300 | -0.27398900 |
| C | 2.84486800 | -0.91547900 | 1.91797400 |
| O | 4.20341300 | 0.53538200 | -0.24817800 |
| C | 4.19875200 | 1.21089400 | -1.52695000 |
| C | 3.88886200 | 2.66804500 | -1.27721400 |
| H | 8.64708400 | -3.88142700 | -1.52636900 |
| H | 6.68485000 | -2.56075900 | -1.61937600 |
| H | 5.95401000 | -3.08458900 | 0.63276100 |
| H | 7.27017400 | -2.12971900 | 1.34051900 |
| H | 9.29645600 | -1.23816000 | -0.89091500 |
| H | 3.54838700 | -0.18530800 | 2.33473600 |
| H | 2.03691500 | -0.38698700 | 1.40737700 |
| H | 5.18755600 | 1.07754400 | -1.98329700 |
| H | 3.43773400 | 0.74288700 | -2.15125500 |
| H | 3.86002400 | 3.20616200 | -2.22894200 |
| H | 4.64563000 | 3.13649400 | -0.63962600 |
| H | 2.91173200 | 2.74725300 | -0.79583200 |
| C | 6.68929100 | 3.62142600 | 1.42584800 |
| N | 7.54088200 | 3.61991900 | 0.34327900 |
| C | 6.18669600 | 2.35984700 | 1.51583200 |
| C | 7.56583200 | 2.40716400 | -0.20855700 |
| N | 6.75024900 | 1.63289700 | 0.49451800 |
| H | 5.47734600 | 1.92505800 | 2.20036900 |
| H | 8.13979000 | 2.09844300 | -1.06774300 |

| | | | |
|---|---|---|---|
| H | 6.53209500 | 0.64298900 | 0.29378200 |
| H | 6.51744400 | 4.50431100 | 2.01866300 |
| H | 8.95812600 | -3.46989600 | 0.02143400 |
| H | 8.07508900 | 4.41403000 | 0.00996900 |
| O | 3.53369000 | -1.72694800 | 0.94938500 |
| C | 2.32160000 | -1.83964100 | 2.99517000 |
| H | 3.14251400 | -2.37992800 | 3.47439500 |
| H | 1.79535300 | -1.25807800 | 3.75720600 |
| H | 1.62160900 | -2.56322900 | 2.56819200 |
| C | -2.30271100 | -2.75231700 | 1.12752900 |
| C | -2.81705500 | -1.47772100 | 0.87574600 |
| C | -1.98984600 | -0.49156000 | 0.34727800 |
| C | -0.62492400 | -0.74277800 | 0.05403200 |
| C | -0.14602600 | -2.03693600 | 0.31419000 |
| C | -0.97160200 | -3.02810200 | 0.84870200 |
| H | -2.95538200 | -3.51647200 | 1.53825600 |
| H | -3.85932500 | -1.27474100 | 1.09584800 |
| C | 0.19922900 | 0.36462900 | -0.44611200 |
| H | 0.89264200 | -2.23584600 | 0.08536100 |
| H | -0.56576500 | -4.01734300 | 1.04009500 |
| O | -2.43024600 | 0.77898200 | 0.07718400 |
| C | -4.00593200 | 2.48370900 | -0.06905400 |
| C | -3.29148900 | 3.32948100 | -0.88519100 |
| N | -5.08273500 | 3.21790100 | 0.29466500 |
| H | -2.35704200 | 3.15166800 | -1.39487900 |
| N | -3.96101700 | 4.50816200 | -0.97370500 |
| N | -5.04383200 | 4.43513800 | -0.26230900 |
| C | -3.77838400 | 1.07066800 | 0.35759800 |
| H | -3.99296400 | 0.94630400 | 1.42926300 |
| H | -4.44705800 | 0.39416200 | -0.19483300 |
| N | 1.43264000 | 0.35635500 | -0.86444500 |
| O | 2.05575800 | -0.80246600 | -0.94816600 |
| H | -0.25835400 | 1.34927500 | -0.44028800 |
| C | -6.18645100 | 2.83921400 | 1.17165700 |

| | | | |
|---|---|---|---|
| H | -5.80960800 | 2.73457500 | 2.19326600 |
| H | -6.87942000 | 3.68350600 | 1.14651700 |
| C | -6.83918100 | 1.55824600 | 0.70584300 |
| C | -6.93931700 | 0.46123000 | 1.55944400 |
| C | -7.31008400 | 1.45252100 | -0.60522000 |
| C | -7.49776200 | -0.73632300 | 1.11585900 |
| H | -6.57013200 | 0.53081500 | 2.57928400 |
| C | -7.86853100 | 0.26532000 | -1.06327500 |
| H | -7.22946200 | 2.30221600 | -1.27866300 |
| C | -7.95192600 | -0.81917500 | -0.19396400 |
| H | -7.56749300 | -1.59278100 | 1.77684200 |
| H | -8.23075700 | 0.17891500 | -2.08140700 |
| Br | -8.68644000 | -2.45015300 | -0.82143200 |

**IN(BZ)**

| | | | |
|---|---|---|---|
| N | 8.95262100 | -1.31910000 | -1.74120600 |
| C | 7.97789900 | -0.34837500 | -1.26123700 |
| C | 6.61747100 | -1.03960200 | -1.08413300 |
| O | 5.67263900 | -0.09164800 | -0.66977400 |
| C | 8.33888000 | 0.27712800 | 0.06662000 |
| O | 8.06313100 | 1.41348400 | 0.38855400 |
| O | 4.07068300 | -0.73805600 | -2.44411600 |
| P | 3.93619100 | -0.47510300 | -0.97311200 |
| C | 3.34375100 | -2.74619100 | 0.46085600 |
| O | 3.63378000 | 1.00603200 | -0.33866000 |
| C | 4.34295500 | 2.16710900 | -0.78017200 |
| C | 3.44791000 | 3.36254400 | -0.52231900 |
| H | 9.86018500 | -0.87516700 | -1.86397400 |
| H | 7.85850700 | 0.45471300 | -1.99508900 |
| H | 6.33238300 | -1.47835300 | -2.04622300 |
| H | 6.70938800 | -1.85500200 | -0.34757600 |
| H | 8.85208700 | -0.40389200 | 0.78023300 |
| H | 3.81988000 | -3.23024000 | 1.31908400 |
| H | 2.34541300 | -2.41887300 | 0.75914300 |

| | | | |
|---|---|---|---|
| H | 5.29134000 | 2.25422400 | -0.23924200 |
| H | 4.57827700 | 2.07223600 | -1.84592000 |
| H | 3.94595500 | 4.28477900 | -0.83489600 |
| H | 3.21433200 | 3.43902800 | 0.54381100 |
| H | 2.51030300 | 3.26263500 | -1.07598400 |
| C | 4.49680100 | 0.68349800 | 3.91090400 |
| N | 5.75109000 | 1.25080800 | 3.95708000 |
| C | 4.33564600 | 0.21992600 | 2.64169700 |
| C | 6.33504700 | 1.14027200 | 2.76330500 |
| N | 5.49135400 | 0.51385200 | 1.95386900 |
| H | 3.51306200 | -0.28359200 | 2.16145900 |
| H | 7.31514100 | 1.50262000 | 2.49208100 |
| H | 5.65496200 | 0.28506000 | 0.93087700 |
| H | 3.84669900 | 0.66278200 | 4.76943100 |
| H | 9.08493100 | -2.05251600 | -1.04538800 |
| H | 6.17396400 | 1.68949900 | 4.76622400 |
| O | 4.17499200 | -1.61419100 | 0.18844600 |
| C | 3.28673500 | -3.70339100 | -0.71552000 |
| H | 4.29500300 | -4.03212700 | -0.98595200 |
| H | 2.69174400 | -4.58494900 | -0.45617500 |
| H | 2.83702900 | -3.21703000 | -1.58530400 |
| C | -2.16534400 | -2.80969800 | 0.84528100 |
| C | -2.62126000 | -1.51627600 | 0.59170500 |
| C | -1.83211000 | -0.64576500 | -0.15924600 |
| C | -0.57631800 | -1.05203800 | -0.65355500 |
| C | -0.15402800 | -2.35754000 | -0.39296500 |
| C | -0.93801600 | -3.23637100 | 0.35105900 |
| H | -2.78418800 | -3.48073200 | 1.43266900 |
| H | -3.58223500 | -1.20486900 | 0.98507200 |
| C | 0.19299100 | -0.10355100 | -1.48311800 |
| H | 0.79652200 | -2.68144100 | -0.79824500 |
| H | -0.58987500 | -4.24697700 | 0.53852200 |
| O | -2.19601200 | 0.63699100 | -0.45416500 |
| C | -3.68574400 | 2.42524100 | -0.59342900 |

| | | | |
|---|---|---|---|
| C | -3.03454000 | 3.13115100 | -1.57798200 |
| N | -4.67338400 | 3.26038800 | -0.19518300 |
| H | -2.17849000 | 2.84321100 | -2.16839400 |
| N | -3.65053500 | 4.33323600 | -1.72330300 |
| N | -4.64052900 | 4.40522500 | -0.88823600 |
| C | -3.47799900 | 1.05527200 | -0.03625300 |
| H | -3.54368200 | 1.06409700 | 1.06088200 |
| H | -4.24963500 | 0.36916600 | -0.41308200 |
| N | 1.46072800 | 0.03816400 | -1.59735000 |
| O | 2.19940900 | -0.79248900 | -0.80436100 |
| H | -0.37982600 | 0.59382100 | -2.08986000 |
| C | -5.66869600 | 3.06479700 | 0.85574500 |
| H | -5.17149100 | 3.11201400 | 1.82915900 |
| H | -6.34589300 | 3.91851800 | 0.77552000 |
| C | -6.39606800 | 1.75102600 | 0.68983000 |
| C | -6.41315800 | 0.81861600 | 1.72540500 |
| C | -7.02627400 | 1.44506300 | -0.51903900 |
| C | -7.04617800 | -0.41230900 | 1.56392400 |
| H | -5.92046300 | 1.04384700 | 2.66749100 |
| C | -7.65993900 | 0.22060000 | -0.69710700 |
| H | -7.01169400 | 2.16464500 | -1.33403300 |
| C | -7.65858100 | -0.69686900 | 0.35012100 |
| H | -7.05421900 | -1.14017600 | 2.36765900 |
| H | -8.14572400 | -0.02113500 | -1.63583400 |
| Br | -8.50205500 | -2.37679700 | 0.10997700 |

**TS2(BZ)**

| | | | |
|---|---|---|---|
| N | 8.87818500 | -1.43169600 | -1.85683600 |
| C | 7.88641800 | -0.42362400 | -1.50807100 |
| C | 6.66688200 | -1.10971300 | -0.87470700 |
| O | 5.69342700 | -0.14267500 | -0.57050400 |
| C | 8.38343300 | 0.61473700 | -0.52495700 |
| O | 8.03491100 | 1.77524800 | -0.51843000 |
| O | 4.08847200 | -1.02833500 | -2.25227900 |

| | | | |
|---|---|---|---|
| P | 3.87140200 | -0.62648400 | -0.82815700 |
| C | 3.40916400 | -2.74044200 | 0.83230000 |
| O | 3.58647700 | 0.90647600 | -0.36805600 |
| C | 4.21840000 | 2.00590300 | -1.03825500 |
| C | 3.19596000 | 3.11827700 | -1.15164700 |
| H | 9.68877100 | -0.99330800 | -2.28852000 |
| H | 7.54831200 | 0.09721200 | -2.40957500 |
| H | 6.26863800 | -1.83201200 | -1.59253200 |
| H | 6.97156900 | -1.65370200 | 0.03455100 |
| H | 9.08133900 | 0.22770500 | 0.24987100 |
| H | 3.90191700 | -3.10716800 | 1.73742600 |
| H | 2.39489100 | -2.42998700 | 1.08986500 |
| H | 5.09527000 | 2.31897200 | -0.46207800 |
| H | 4.56624800 | 1.68517700 | -2.02473600 |
| H | 3.63379100 | 3.98508200 | -1.65495800 |
| H | 2.85484200 | 3.42897800 | -0.16011400 |
| H | 2.33034300 | 2.77451200 | -1.72430800 |
| C | 5.51305600 | 0.90076500 | 3.94078500 |
| N | 6.44208600 | 1.82734500 | 3.52783500 |
| C | 5.18630100 | 0.17979000 | 2.83115200 |
| C | 6.66507900 | 1.66729300 | 2.21712400 |
| N | 5.91487800 | 0.67109100 | 1.77225300 |
| H | 4.49410900 | -0.63622500 | 2.70146100 |
| H | 7.34958900 | 2.25499700 | 1.62316100 |
| H | 5.82513500 | 0.28987300 | 0.64174400 |
| H | 5.17787800 | 0.84319200 | 4.96317000 |
| H | 9.21777200 | -1.89216100 | -1.01290100 |
| H | 6.89008700 | 2.52083100 | 4.11360100 |
| O | 4.18932700 | -1.59873900 | 0.44668600 |
| C | 3.40985100 | -3.81302600 | -0.24048000 |
| H | 4.43553900 | -4.11026400 | -0.47905200 |
| H | 2.86440800 | -4.69462100 | 0.11074600 |
| H | 2.93483300 | -3.44657100 | -1.15498300 |
| C | -2.10937900 | -2.93175000 | 1.24952700 |

| | | | |
|---|---|---|---|
| C | -2.58306900 | -1.66602500 | 0.90592100 |
| C | -1.81707300 | -0.85208600 | 0.07266000 |
| C | -0.57477400 | -1.29255600 | -0.42500500 |
| C | -0.13110400 | -2.56807900 | -0.06944600 |
| C | -0.88836600 | -3.38754500 | 0.76457800 |
| H | -2.70932200 | -3.55986800 | 1.90043500 |
| H | -3.53891100 | -1.33167600 | 1.29254300 |
| C | 0.15356400 | -0.41203400 | -1.36002100 |
| H | 0.81272200 | -2.91840100 | -0.47013400 |
| H | -0.52672300 | -4.37616600 | 1.02748500 |
| O | -2.18704200 | 0.40615800 | -0.30310600 |
| C | -3.60119400 | 2.25133500 | -0.41479900 |
| C | -2.94175400 | 2.93229300 | -1.41140700 |
| N | -4.54235000 | 3.12563700 | 0.00792200 |
| H | -2.10854100 | 2.61292200 | -2.01821700 |
| N | -3.51168100 | 4.15873300 | -1.54147000 |
| N | -4.48019200 | 4.26916800 | -0.68524000 |
| C | -3.43885200 | 0.87842300 | 0.14802500 |
| H | -3.47020800 | 0.90387400 | 1.24680200 |
| H | -4.24815300 | 0.21919200 | -0.19696400 |
| N | 1.41315100 | -0.24851400 | -1.51127700 |
| O | 2.17892900 | -0.98143200 | -0.63846000 |
| H | -0.44697600 | 0.20465000 | -2.02464800 |
| C | -5.54356400 | 2.94338900 | 1.05295700 |
| H | -5.03752000 | 2.83530300 | 2.01675400 |
| H | -6.11729300 | 3.87313900 | 1.07047800 |
| C | -6.42238600 | 1.74726500 | 0.76536500 |
| C | -6.60650000 | 0.75074000 | 1.72211800 |
| C | -7.03980300 | 1.62423300 | -0.48204400 |
| C | -7.40077000 | -0.36128800 | 1.44610700 |
| H | -6.12505200 | 0.83361600 | 2.69289100 |
| C | -7.83092400 | 0.52024700 | -0.77372700 |
| H | -6.89340700 | 2.39390600 | -1.23577800 |
| C | -8.00098000 | -0.46270500 | 0.19800900 |

| | | | |
|---|---|---|---|
| H | -7.54231200 | -1.13790200 | 2.18906800 |
| H | -8.30758500 | 0.41912000 | -1.74212700 |
| Br | -9.06735100 | -1.97668100 | -0.20627300 |

**C2(BZ)**

| | | | |
|---|---|---|---|
| N | 10.02521400 | -0.05498600 | -0.96365000 |
| C | 8.57802600 | 0.13969200 | -0.99843900 |
| C | 7.90213300 | -0.98835300 | -0.20622800 |
| O | 6.50070300 | -0.93050000 | -0.28557800 |
| C | 8.16525000 | 1.47040700 | -0.40680700 |
| O | 7.40587100 | 2.25825800 | -0.92614500 |
| O | 3.90140400 | -1.64142100 | -2.67834700 |
| P | 3.47438400 | -1.37113900 | -1.29467000 |
| C | 3.91942000 | -2.17823700 | 1.17641600 |
| O | 3.79317900 | 0.06934900 | -0.71645300 |
| C | 4.54514600 | 1.03601800 | -1.49156600 |
| C | 3.63636500 | 1.77169500 | -2.45187500 |
| H | 10.49303100 | 0.68299700 | -1.48568100 |
| H | 8.22446900 | 0.10246300 | -2.03408000 |
| H | 8.24272700 | -1.93907900 | -0.62775700 |
| H | 8.24433100 | -0.94582100 | 0.84087500 |
| H | 8.62797200 | 1.69427000 | 0.57904200 |
| H | 4.14741800 | -1.12366000 | 1.35760200 |
| H | 2.89656200 | -2.38629200 | 1.50553400 |
| H | 4.97837800 | 1.70490800 | -0.74723500 |
| H | 5.36317600 | 0.52432900 | -2.00041900 |
| H | 4.20714600 | 2.54462000 | -2.97504200 |
| H | 2.81173700 | 2.24766200 | -1.91473200 |
| H | 3.21922400 | 1.08225900 | -3.19041600 |
| C | 5.57202600 | 1.43213300 | 3.92996300 |
| N | 5.70809400 | 2.55952100 | 3.15949900 |
| C | 5.78788000 | 0.38531200 | 3.07644200 |
| C | 5.99554800 | 2.17173300 | 1.89765400 |
| N | 6.04970500 | 0.85691700 | 1.80983700 |

| | | | |
|---|---|---|---|
| H | 5.77889900 | -0.67283700 | 3.29618200 |
| H | 6.17473600 | 2.86726800 | 1.08886100 |
| H | 6.20521000 | -0.26562300 | 0.38362000 |
| H | 5.34260700 | 1.47979400 | 4.98228500 |
| H | 10.36164300 | 0.01688600 | -0.00346700 |
| H | 5.61678700 | 3.51474500 | 3.47849700 |
| O | 3.98887200 | -2.43438100 | -0.24451000 |
| C | 4.92816000 | -3.07899000 | 1.85148400 |
| H | 4.86111000 | -2.96422100 | 2.93719200 |
| H | 4.73536800 | -4.12642200 | 1.60485500 |
| H | 5.93526100 | -2.81132800 | 1.52073900 |
| C | -2.51068400 | -3.04839900 | 1.10566800 |
| C | -2.89962500 | -1.74359800 | 0.80996500 |
| C | -2.08631900 | -0.95484600 | -0.00247100 |
| C | -0.87651200 | -1.46435600 | -0.52259800 |
| C | -0.51537200 | -2.78040300 | -0.21085900 |
| C | -1.32609900 | -3.57079900 | 0.59749800 |
| H | -3.14767800 | -3.65663100 | 1.73993000 |
| H | -3.82787200 | -1.35889900 | 1.21555500 |
| C | -0.11282000 | -0.58795400 | -1.42528500 |
| H | 0.40002500 | -3.18742600 | -0.61947900 |
| H | -1.03393200 | -4.58996300 | 0.82546900 |
| O | -2.37695200 | 0.33372600 | -0.33189500 |
| C | -3.70251100 | 2.25053700 | -0.41259500 |
| C | -3.03126500 | 2.91012100 | -1.41571100 |
| N | -4.58948700 | 3.16583900 | 0.04071200 |
| H | -2.23074100 | 2.55794600 | -2.04765400 |
| N | -3.54081200 | 4.16519300 | -1.51865400 |
| N | -4.48403800 | 4.31321100 | -0.64019900 |
| C | -3.60385200 | 0.86332300 | 0.12972300 |
| H | -3.62895100 | 0.87288100 | 1.22848700 |
| H | -4.44376200 | 0.24871800 | -0.22306100 |
| N | 1.13193100 | -0.54891000 | -1.70919600 |
| O | 1.87340900 | -1.51406000 | -0.99925500 |

| | | | |
|---|---|---|---|
| H | -0.67375300 | 0.17947800 | -1.95260000 |
| C | -5.57668600 | 3.02162400 | 1.10554400 |
| H | -5.05615500 | 2.89138000 | 2.05888900 |
| H | -6.11208000 | 3.97368700 | 1.13528000 |
| C | -6.50654500 | 1.86102600 | 0.83406000 |
| C | -6.69933300 | 0.86442000 | 1.78907500 |
| C | -7.15757600 | 1.76615500 | -0.39852000 |
| C | -7.53244700 | -0.22156400 | 1.52471400 |
| H | -6.19236900 | 0.92549100 | 2.74845600 |
| C | -7.98856400 | 0.68854400 | -0.67829700 |
| H | -7.00524500 | 2.53590600 | -1.15102500 |
| C | -8.16383300 | -0.29667600 | 0.29020100 |
| H | -7.67891100 | -0.99920400 | 2.26563300 |
| H | -8.49060800 | 0.60867300 | -1.63580200 |
| Br | -9.27706000 | -1.77995900 | -0.10109700 |

**CE**

| | | | |
|---|---|---|---|
| C | 3.81689500 | -1.15297400 | -1.06853200 |
| C | 2.53761300 | -1.67049100 | -0.91652700 |
| C | 1.63229900 | -1.11844200 | -0.01244500 |
| C | 1.96990500 | -0.00133400 | 0.79497900 |
| C | 3.26679200 | 0.51235200 | 0.62508300 |
| C | 4.14738400 | -0.05512000 | -0.28273900 |
| H | 4.52834100 | -1.57285900 | -1.76712000 |
| H | 2.21337700 | -2.52744700 | -1.49803700 |
| C | 1.12044800 | 0.62750000 | 1.79122800 |
| H | 3.58796400 | 1.36288400 | 1.21536300 |
| H | 1.55862100 | 1.43956700 | 2.37991000 |
| O | 0.41004500 | -1.73218900 | 0.08893900 |
| N | -0.11957100 | 0.28082200 | 2.01692600 |
| O | -0.74311500 | 0.93304200 | 2.94703900 |
| C | -1.91280300 | -1.75648100 | -0.33382300 |
| C | -2.33631900 | -3.00650100 | -0.73206800 |
| N | -2.95171400 | -1.28799400 | 0.39943100 |

| | | | |
|---|---|---|---|
| H | -1.81629800 | -3.73825800 | -1.33301600 |
| N | -3.57837700 | -3.22598400 | -0.24017700 |
| N | -3.94719600 | -2.17575200 | 0.43666700 |
| C | -0.63331800 | -1.03509300 | -0.59780500 |
| H | -0.41886100 | -1.04969300 | -1.67367500 |
| H | -0.68958800 | -0.00174300 | -0.25013900 |
| C | -3.12318300 | 0.00572300 | 1.06328200 |
| H | -4.11308400 | -0.05638100 | 1.52415300 |
| H | -2.36919100 | 0.12937000 | 1.84738800 |
| C | -3.05859600 | 1.15610900 | 0.08315000 |
| C | -3.77248000 | 1.11388900 | -1.11696500 |
| C | -2.28695700 | 2.27838200 | 0.39005100 |
| C | -3.72066000 | 2.18721500 | -2.00128000 |
| H | -4.36795700 | 0.23552700 | -1.35748300 |
| C | -2.24134100 | 3.35512100 | -0.49504400 |
| H | -1.71915700 | 2.28170700 | 1.31853000 |
| C | -2.95565100 | 3.31190200 | -1.69017000 |
| H | -4.27780500 | 2.14821300 | -2.93243700 |
| H | -1.63911600 | 4.22596600 | -0.25355900 |
| H | -2.91463400 | 4.14888200 | -2.38057400 |
| N | 5.48410500 | 0.52475100 | -0.41533400 |
| O | 5.76798000 | 1.49245200 | 0.27251200 |
| O | 6.25590200 | 0.01329900 | -1.21151000 |

**C1(CE)**

| | | | |
|---|---|---|---|
| N | 7.02835300 | 1.72699400 | -2.39878000 |
| C | 5.81623500 | 1.56478500 | -1.61579500 |
| C | 5.43094900 | 0.07532700 | -1.66083600 |
| O | 4.30474800 | -0.22443700 | -0.82966100 |
| C | 5.91762100 | 2.05373900 | -0.17981000 |
| O | 4.97346900 | 2.11020100 | 0.57980400 |
| O | 2.73670600 | 1.44841700 | -2.06745400 |
| P | 2.81226900 | 0.15365800 | -1.36581000 |
| C | 2.22515800 | -2.39630000 | -1.73818700 |

| | | | |
|---|---|---|---|
| O | 2.05775500 | 0.00027100 | 0.02129500 |
| C | 1.36261700 | 1.16616700 | 0.53741600 |
| C | 0.53540000 | 0.73431200 | 1.72273700 |
| H | 7.21408000 | 2.71273700 | -2.56437600 |
| H | 5.00130100 | 2.12760500 | -2.08215500 |
| H | 5.23046200 | -0.20293900 | -2.69972000 |
| H | 6.25154200 | -0.54259400 | -1.28794600 |
| H | 6.92695800 | 2.36952600 | 0.15330700 |
| H | 3.16530800 | -2.94020400 | -1.86717000 |
| H | 1.99990700 | -2.31876200 | -0.67106200 |
| H | 2.11828100 | 1.90908300 | 0.81704700 |
| H | 0.73060000 | 1.56458700 | -0.25614400 |
| H | 0.05730700 | 1.61235100 | 2.16686900 |
| H | 1.15538100 | 0.25352000 | 2.48828800 |
| H | -0.24249200 | 0.03635900 | 1.40181200 |
| C | 3.44045100 | -1.12026000 | 3.89877600 |
| N | 3.66247800 | 0.21875000 | 4.13119300 |
| C | 3.70290900 | -1.32661700 | 2.57845200 |
| C | 4.04831200 | 0.81482700 | 3.00392500 |
| N | 4.07811300 | -0.11232600 | 2.05624400 |
| H | 3.66264500 | -2.21887100 | 1.97492900 |
| H | 4.29877600 | 1.85573900 | 2.87372500 |
| H | 4.29096300 | 0.06769700 | 1.06953300 |
| H | 3.12338700 | -1.79304900 | 4.67822000 |
| H | 7.83313200 | 1.35754700 | -1.89495400 |
| H | 3.55859500 | 0.69002700 | 5.02273600 |
| O | 2.41538000 | -1.06865400 | -2.28441100 |
| C | 1.07537800 | -3.03379200 | -2.48451600 |
| H | 1.32483400 | -3.16605700 | -3.54113100 |
| H | 0.84672200 | -4.01252700 | -2.05143400 |
| H | 0.20383700 | -2.37861500 | -2.39395200 |
| C | -5.10790500 | 3.51672700 | 0.51928400 |
| C | -5.16712000 | 2.16600600 | 0.20594100 |
| C | -4.07282700 | 1.50347400 | -0.34892800 |

| | | | |
|---|---|---|---|
| C | -2.84735200 | 2.16809100 | -0.59223800 |
| C | -2.81002800 | 3.53927300 | -0.29461000 |
| C | -3.91508700 | 4.17671800 | 0.24800900 |
| H | -5.95078000 | 4.04548800 | 0.94462200 |
| H | -6.08096000 | 1.60166400 | 0.36419200 |
| C | -1.65797300 | 1.55258700 | -1.16267400 |
| H | -1.90430400 | 4.10571100 | -0.47949700 |
| H | -0.91097600 | 2.21231800 | -1.61592700 |
| O | -4.25062800 | 0.19565700 | -0.72323700 |
| N | -1.42793100 | 0.27022400 | -1.10088800 |
| O | -0.31394200 | -0.15391900 | -1.61499800 |
| C | -4.50980500 | -2.07946300 | -0.15978300 |
| C | -5.74784000 | -2.68322400 | -0.11823600 |
| N | -3.75024900 | -2.96240600 | -0.85059600 |
| H | -6.66424000 | -2.32339000 | 0.32649500 |
| N | -5.67433000 | -3.87474400 | -0.75814400 |
| N | -4.45661900 | -4.03804400 | -1.19439800 |
| C | -4.02621400 | -0.75437700 | 0.32245000 |
| H | -4.59658600 | -0.46658200 | 1.21278700 |
| H | -2.95696500 | -0.77972900 | 0.56020700 |
| C | -2.31309700 | -2.91221900 | -1.10722800 |
| H | -2.13657400 | -3.60947300 | -1.93106600 |
| H | -2.04742200 | -1.89955800 | -1.42498800 |
| C | -1.51964600 | -3.29411300 | 0.12454700 |
| C | -1.86800000 | -4.40961100 | 0.89029900 |
| C | -0.42183400 | -2.51456000 | 0.49104000 |
| C | -1.11773500 | -4.74818900 | 2.01336900 |
| H | -2.73049300 | -5.00948500 | 0.60838000 |
| C | 0.32583800 | -2.85801900 | 1.61785500 |
| H | -0.16306800 | -1.64597700 | -0.11874100 |
| C | -0.01692700 | -3.97251400 | 2.37958100 |
| H | -1.39268200 | -5.61647700 | 2.60448400 |
| H | 1.17115900 | -2.23702200 | 1.90303800 |
| H | 0.56509000 | -4.23498800 | 3.25806600 |

| | | | |
|---|---|---|---|
| N | -3.82090900 | 5.60574000 | 0.54942100 |
| O | -2.77588200 | 6.18357500 | 0.29720300 |
| O | -4.79485300 | 6.15357800 | 1.04086700 |

**TS1(CE)**

| | | | |
|---|---|---|---|
| N | 6.67234000 | 2.09093600 | -2.05701600 |
| C | 5.68355900 | 1.70398800 | -1.06486700 |
| C | 4.63489900 | 0.80946000 | -1.73709200 |
| O | 3.63114900 | 0.49829100 | -0.78174200 |
| C | 6.25333700 | 0.97599300 | 0.14036800 |
| O | 5.67675400 | 0.88091400 | 1.20255300 |
| O | 1.77828500 | 1.25591700 | -2.32081300 |
| P | 2.07284000 | 0.22720300 | -1.29825700 |
| C | 1.63450900 | -2.14532500 | -2.61380300 |
| O | 1.46752200 | 0.26813500 | 0.18553600 |
| C | 1.68818300 | 1.44323300 | 0.99338500 |
| C | 0.77070300 | 1.33991800 | 2.19010500 |
| H | 7.32817200 | 2.75835500 | -1.65797700 |
| H | 5.16401700 | 2.59541000 | -0.69727300 |
| H | 4.20295300 | 1.35789100 | -2.57773600 |
| H | 5.09090700 | -0.11754800 | -2.10966800 |
| H | 7.24364900 | 0.49956100 | -0.01101800 |
| H | 2.29751900 | -3.00958900 | -2.69726700 |
| H | 0.67899600 | -2.45281800 | -2.19209700 |
| H | 2.74235700 | 1.48045800 | 1.28765600 |
| H | 1.46681600 | 2.33578200 | 0.39694400 |
| H | 0.87910700 | 2.22528900 | 2.82246600 |
| H | 1.02260800 | 0.45605000 | 2.78365500 |
| H | -0.27040300 | 1.26162600 | 1.86184300 |
| C | 2.98146300 | -2.99321900 | 2.53957100 |
| N | 4.11068100 | -2.50200600 | 3.15713000 |
| C | 2.77771200 | -2.21774000 | 1.44041000 |
| C | 4.58512000 | -1.46500900 | 2.47015900 |
| N | 3.78596600 | -1.28069200 | 1.42578900 |

| | | | |
|---|---|---|---|
| H | 2.01645400 | -2.24034500 | 0.67656500 |
| H | 5.45492900 | -0.87306700 | 2.70643100 |
| H | 3.90663500 | -0.55035700 | 0.70831100 |
| H | 2.43138400 | -3.83199900 | 2.93338900 |
| H | 7.22174400 | 1.28179200 | -2.34363100 |
| H | 4.52356900 | -2.86509300 | 4.00838800 |
| O | 2.30081400 | -1.30844100 | -1.64277400 |
| C | 1.45384900 | -1.46579400 | -3.95453600 |
| H | 2.41044400 | -1.08946600 | -4.33012000 |
| H | 1.06173700 | -2.19281600 | -4.67201000 |
| H | 0.75096100 | -0.63771200 | -3.85938400 |
| C | -4.26982500 | 3.92442100 | 0.68598500 |
| C | -4.59586600 | 2.68295700 | 0.15853600 |
| C | -3.61506200 | 1.83403800 | -0.35287000 |
| C | -2.24657100 | 2.19621000 | -0.34789800 |
| C | -1.93280900 | 3.45664300 | 0.17897900 |
| C | -2.92718100 | 4.28214000 | 0.68182900 |
| H | -5.02056200 | 4.59495700 | 1.08323700 |
| H | -5.62804700 | 2.35010800 | 0.11918800 |
| C | -1.14885100 | 1.39483900 | -0.88636300 |
| H | -0.90167800 | 3.79298000 | 0.19757800 |
| H | -0.20422800 | 1.90837000 | -1.07220600 |
| O | -4.03681700 | 0.65507800 | -0.90973800 |
| N | -1.27790000 | 0.12917900 | -1.13745500 |
| O | -0.24390500 | -0.52212700 | -1.60621400 |
| C | -4.47798800 | -1.66136400 | -0.71866000 |
| C | -5.73722600 | -2.03085800 | -1.14069200 |
| N | -3.69247400 | -2.68626500 | -1.13040800 |
| H | -6.67278400 | -1.50287800 | -1.02669800 |
| N | -5.65636300 | -3.23065000 | -1.76263700 |
| N | -4.41424900 | -3.62460900 | -1.74399600 |
| C | -4.00487300 | -0.44559100 | 0.00501300 |
| H | -4.68133600 | -0.23399900 | 0.84213500 |
| H | -2.98757200 | -0.58381900 | 0.37860600 |

| | | | |
|---|---|---|---|
| C | -2.25896400 | -2.89886500 | -0.92479300 |
| H | -2.03798600 | -3.82448300 | -1.46366700 |
| H | -1.69479000 | -2.07732000 | -1.37489300 |
| C | -1.92209100 | -3.03096300 | 0.54416100 |
| C | -2.67757000 | -3.86679100 | 1.37009100 |
| C | -0.85269800 | -2.30928600 | 1.07830400 |
| C | -2.36605700 | -3.98790200 | 2.72095500 |
| H | -3.51543900 | -4.41946900 | 0.94955400 |
| C | -0.54615400 | -2.43140700 | 2.43417900 |
| H | -0.28548200 | -1.64550900 | 0.42503800 |
| C | -1.29753700 | -3.26863000 | 3.25598000 |
| H | -2.95831500 | -4.63902300 | 3.35663700 |
| H | 0.27745700 | -1.86111300 | 2.85376600 |
| H | -1.05717800 | -3.35530600 | 4.31130500 |
| N | -2.54524900 | 5.58494800 | 1.22826400 |
| O | -1.36469000 | 5.89387600 | 1.21386400 |
| O | -3.42718000 | 6.30093000 | 1.67439000 |

**IN(CE)**

| | | | |
|---|---|---|---|
| N | 6.27221700 | 2.32589200 | -2.47723500 |
| C | 5.39152600 | 2.06453700 | -1.34705100 |
| C | 4.22595900 | 1.18152700 | -1.81325900 |
| O | 3.34342200 | 0.98201200 | -0.73810100 |
| C | 6.06573300 | 1.35876800 | -0.19217800 |
| O | 5.77051600 | 1.52208100 | 0.97278100 |
| O | 1.39528400 | 1.56325700 | -2.13881200 |
| P | 1.68983500 | 0.49354800 | -1.12630900 |
| C | 1.73759600 | -2.11680300 | -2.06614800 |
| O | 1.36507800 | 0.64319900 | 0.47601800 |
| C | 1.76994300 | 1.79415500 | 1.22582100 |
| C | 0.85696200 | 1.87601900 | 2.43210000 |
| H | 7.05301800 | 2.91040800 | -2.18723100 |
| H | 4.97674700 | 3.00544400 | -0.97184700 |
| H | 3.72085400 | 1.69357700 | -2.63723500 |

| | | | |
|---|---|---|---|
| H | 4.61115200 | 0.21874300 | -2.18534800 |
| H | 6.84811900 | 0.62406100 | -0.48000300 |
| H | 2.52481400 | -2.87621600 | -2.03474500 |
| H | 0.90678100 | -2.45475100 | -1.44192100 |
| H | 2.81988200 | 1.69809600 | 1.52054200 |
| H | 1.68311300 | 2.69104900 | 0.59990400 |
| H | 1.12737700 | 2.73203600 | 3.05638000 |
| H | 0.94221600 | 0.96468500 | 3.03205900 |
| H | -0.18581400 | 1.98549900 | 2.11727100 |
| C | 3.35882900 | -2.62557700 | 2.46897000 |
| N | 4.53764400 | -2.04480700 | 2.88149200 |
| C | 2.92459200 | -1.89219000 | 1.40796800 |
| C | 4.81530600 | -0.99568900 | 2.10754500 |
| N | 3.84724500 | -0.89103100 | 1.20586100 |
| H | 2.04762000 | -1.98314200 | 0.78785800 |
| H | 5.66591300 | -0.33735000 | 2.19609400 |
| H | 3.77106700 | -0.15206200 | 0.46407400 |
| H | 2.94160200 | -3.49096200 | 2.95718500 |
| H | 6.67704000 | 1.45276700 | -2.81364600 |
| H | 5.11201100 | -2.35466700 | 3.65632900 |
| O | 2.35028000 | -0.96251900 | -1.47690400 |
| C | 1.29496100 | -1.87116900 | -3.49498500 |
| H | 2.13790000 | -1.51972600 | -4.09780300 |
| H | 0.92108400 | -2.80170100 | -3.93312200 |
| H | 0.49941100 | -1.12401900 | -3.52387100 |
| C | -4.75711200 | 3.55155800 | 0.52085200 |
| C | -4.84538000 | 2.26169600 | 0.01846200 |
| C | -3.70172300 | 1.55769900 | -0.36081700 |
| C | -2.41989000 | 2.13574300 | -0.24017300 |
| C | -2.34121400 | 3.43969600 | 0.25678100 |
| C | -3.49273000 | 4.11543600 | 0.63015100 |
| H | -5.63582300 | 4.11068900 | 0.81598900 |
| H | -5.80861700 | 1.77728600 | -0.10262900 |
| C | -1.14904700 | 1.50006100 | -0.62911400 |

| | | | |
|---|---|---|---|
| H | -1.37822900 | 3.92959000 | 0.35377500 |
| H | -0.28738700 | 2.16136500 | -0.69584400 |
| O | -3.87840300 | 0.31381000 | -0.89971200 |
| N | -1.09150600 | 0.24662200 | -0.88111200 |
| O | 0.08284700 | -0.33278900 | -1.21725000 |
| C | -4.22458100 | -2.01245700 | -0.61622300 |
| C | -5.45084900 | -2.55310500 | -0.93680000 |
| N | -3.34190000 | -2.91108900 | -1.11652000 |
| H | -6.43783000 | -2.16265000 | -0.73568200 |
| N | -5.25533700 | -3.72351300 | -1.58898300 |
| N | -3.97304300 | -3.93508400 | -1.68799500 |
| C | -3.85245900 | -0.74248500 | 0.06951200 |
| H | -4.58511800 | -0.52979700 | 0.85586300 |
| H | -2.85399400 | -0.80846700 | 0.51334900 |
| C | -1.88316800 | -2.92050700 | -1.00830900 |
| H | -1.54297100 | -3.63288000 | -1.76447500 |
| H | -1.51198100 | -1.92618300 | -1.26059400 |
| C | -1.43559900 | -3.33566900 | 0.37768600 |
| C | -1.82991500 | -4.57457400 | 0.88988800 |
| C | -0.63929800 | -2.49044800 | 1.15333500 |
| C | -1.42869800 | -4.96969900 | 2.16158900 |
| H | -2.45654000 | -5.22643600 | 0.28523200 |
| C | -0.24119300 | -2.88781800 | 2.43175300 |
| H | -0.33149700 | -1.52465700 | 0.75460300 |
| C | -0.63168600 | -4.12505900 | 2.93607500 |
| H | -1.73782900 | -5.93501300 | 2.55045600 |
| H | 0.37210200 | -2.22305500 | 3.03407500 |
| H | -0.32109800 | -4.43106300 | 3.93035700 |
| N | -3.36928200 | 5.47737200 | 1.15313700 |
| O | -2.25153800 | 5.95472500 | 1.25270300 |
| O | -4.39199600 | 6.06420800 | 1.46404400 |

**TS2(CE)**

| | | | |
|---|---|---|---|
| N | -6.10456500 | 3.07897700 | 1.57919000 |

| | | | |
|---|---|---|---|
| C | -5.05561800 | 2.66767600 | 0.65782000 |
| C | -4.18349800 | 1.60516500 | 1.33987000 |
| O | -3.16350500 | 1.19878000 | 0.45101400 |
| C | -5.56499700 | 2.08518600 | -0.64693500 |
| O | -5.00163800 | 2.20922400 | -1.71185600 |
| O | -1.30412900 | 2.02012100 | 1.91487800 |
| P | -1.46973600 | 0.72227000 | 1.19219200 |
| C | -1.94822300 | -1.39460900 | 2.85549300 |
| O | -1.03809800 | 0.53926900 | -0.36059300 |
| C | -1.25745000 | 1.55974700 | -1.34806200 |
| C | -0.15707900 | 1.42684600 | -2.38079200 |
| H | -6.67240700 | 3.81271000 | 1.16163200 |
| H | -4.41744800 | 3.52274900 | 0.41341700 |
| H | -3.73512400 | 2.04091100 | 2.23593000 |
| H | -4.79561600 | 0.73988300 | 1.63556200 |
| H | -6.49740300 | 1.48549800 | -0.56269500 |
| H | -2.79121000 | -2.08502200 | 2.94811400 |
| H | -1.06117400 | -1.96081200 | 2.56779300 |
| H | -2.24557400 | 1.42176600 | -1.79611100 |
| H | -1.24166100 | 2.54397600 | -0.86655000 |
| H | -0.29668200 | 2.16872700 | -3.17197400 |
| H | -0.17530700 | 0.42921300 | -2.82906000 |
| H | 0.82568200 | 1.58338000 | -1.92559000 |
| C | -4.30772000 | -2.88885900 | -1.41306800 |
| N | -4.64538500 | -2.05207800 | -2.45010300 |
| C | -3.85684100 | -2.07724700 | -0.41352900 |
| C | -4.40923800 | -0.78489400 | -2.07790600 |
| N | -3.92803300 | -0.77248400 | -0.84483300 |
| H | -3.47885600 | -2.32395100 | 0.56540500 |
| H | -4.61089300 | 0.08510500 | -2.68548000 |
| H | -3.53929800 | 0.25343400 | -0.19389700 |
| H | -4.40933300 | -3.95937500 | -1.48422200 |
| H | -6.73344300 | 2.29875200 | 1.76649500 |
| H | -5.02266800 | -2.33656600 | -3.34532500 |

| | | | |
|---|---|---|---|
| O | -2.33627400 | -0.52266200 | 1.77792700 |
| C | -1.73561500 | -0.64059200 | 4.15324500 |
| H | -2.62663800 | -0.05752700 | 4.40414500 |
| H | -1.54640000 | -1.35039000 | 4.96375100 |
| H | -0.88099200 | 0.03498600 | 4.07296100 |
| C | 5.25189900 | 2.85462400 | -0.57978000 |
| C | 5.16871400 | 1.55436700 | -0.10492500 |
| C | 3.95817500 | 1.03348900 | 0.35293900 |
| C | 2.78495700 | 1.81764200 | 0.35127700 |
| C | 2.87703300 | 3.12668100 | -0.12903900 |
| C | 4.09094700 | 3.61693000 | -0.58529600 |
| H | 6.18399000 | 3.27059700 | -0.94061200 |
| H | 6.04414100 | 0.91460600 | -0.07727200 |
| C | 1.45980900 | 1.38901600 | 0.83202500 |
| H | 2.00059100 | 3.76532000 | -0.14697800 |
| H | 0.68681000 | 2.15796500 | 0.84105100 |
| O | 3.96692500 | -0.24699900 | 0.82822500 |
| N | 1.24315000 | 0.19372400 | 1.23211600 |
| O | -0.01330500 | -0.13932800 | 1.65265900 |
| C | 3.82532000 | -2.57515200 | 0.42276700 |
| C | 4.97608000 | -3.31203400 | 0.59845100 |
| N | 2.85808100 | -3.36855800 | 0.94269400 |
| H | 5.99247300 | -3.05771200 | 0.33551400 |
| N | 4.65494400 | -4.48734300 | 1.18998800 |
| N | 3.36720600 | -4.51664300 | 1.38685600 |
| C | 3.59238200 | -1.22174000 | -0.15555000 |
| H | 4.21971100 | -1.09251200 | -1.04447500 |
| H | 2.54153200 | -1.08512000 | -0.43311200 |
| C | 1.41435200 | -3.16024600 | 0.94558500 |
| H | 1.01378700 | -3.92367700 | 1.61804900 |
| H | 1.20524200 | -2.17337800 | 1.36720900 |
| C | 0.82516100 | -3.27821200 | -0.44481800 |
| C | 1.33996400 | -4.18438800 | -1.37524600 |
| C | -0.25565900 | -2.46975700 | -0.79493800 |

| | | | |
|---|---|---|---|
| C | 0.77582400 | -4.27757500 | -2.64455800 |
| H | 2.18606200 | -4.81305800 | -1.10725100 |
| C | -0.81625800 | -2.56084600 | -2.06790800 |
| H | -0.64682200 | -1.75423000 | -0.07886400 |
| C | -0.30209900 | -3.46421400 | -2.99539900 |
| H | 1.18121500 | -4.98302700 | -3.36337500 |
| H | -1.64800800 | -1.91194100 | -2.33252600 |
| H | -0.73474000 | -3.53419900 | -3.98870800 |
| N | 4.14997000 | 4.99079600 | -1.08819500 |
| O | 3.12119400 | 5.64504500 | -1.09172800 |
| O | 5.22670200 | 5.40806400 | -1.47929700 |

**C2(CE)**

| | | | |
|---|---|---|---|
| N | 3.98816400 | 4.85906600 | 0.64080500 |
| C | 3.42734000 | 3.57938200 | 1.07567500 |
| C | 4.17595400 | 2.44102700 | 0.39636000 |
| O | 3.74223200 | 1.22936500 | 0.96959700 |
| C | 3.53282000 | 3.40726000 | 2.57716100 |
| O | 2.59379900 | 3.29299400 | 3.33168500 |
| O | 1.27445900 | 2.02998900 | -0.72808400 |
| P | 1.32857900 | 0.55793600 | -0.67357900 |
| C | 2.82852300 | -1.52663400 | -1.18821000 |
| O | 1.19271500 | -0.12662400 | 0.74828200 |
| C | 1.02298200 | 0.64359800 | 1.96274000 |
| C | -0.40346400 | 0.48850300 | 2.44529600 |
| H | 3.45235700 | 5.62675800 | 1.04007200 |
| H | 2.37245000 | 3.53040400 | 0.79246600 |
| H | 3.97699600 | 2.48437200 | -0.68332300 |
| H | 5.25979700 | 2.58097000 | 0.55114400 |
| H | 4.57355000 | 3.44034500 | 2.97031300 |
| H | 3.36508900 | -1.61290600 | -0.23976200 |
| H | 1.87999000 | -2.06791600 | -1.11097700 |
| H | 1.74584500 | 0.23839500 | 2.67265700 |
| H | 1.28622300 | 1.68396600 | 1.76976200 |

| | | | |
|---|---|---|---|
| H | -0.53523200 | 1.02062500 | 3.39185200 |
| H | -0.64251100 | -0.56804100 | 2.60371700 |
| H | -1.10564500 | 0.89868900 | 1.71204100 |
| C | 7.08829300 | -1.93161500 | -1.03187300 |
| N | 7.15262300 | -0.79355100 | -1.79586600 |
| C | 6.19833000 | -1.64535900 | -0.03270900 |
| C | 6.31954800 | 0.12118900 | -1.24949800 |
| N | 5.72447400 | -0.36200400 | -0.17787500 |
| H | 5.87069200 | -2.28204500 | 0.77634900 |
| H | 6.18119600 | 1.10871300 | -1.66606100 |
| H | 4.36239400 | 0.53617400 | 0.63649500 |
| H | 7.66520200 | -2.81205900 | -1.26494600 |
| H | 4.94016200 | 4.96005200 | 0.99305300 |
| H | 7.72218600 | -0.65877700 | -2.62060700 |
| O | 2.55523600 | -0.11709800 | -1.40620000 |
| C | 3.65290000 | -2.02410300 | -2.35187800 |
| H | 3.92375200 | -3.07021800 | -2.18560500 |
| H | 3.08445500 | -1.95209800 | -3.28280400 |
| H | 4.57077400 | -1.43843400 | -2.45010100 |
| C | -5.39800300 | 2.78754600 | -0.10187400 |
| C | -5.20856200 | 1.41448300 | -0.09061900 |
| C | -4.00210200 | 0.86325400 | -0.52636800 |
| C | -2.95784300 | 1.69184300 | -0.97598900 |
| C | -3.16249600 | 3.07142200 | -1.01842100 |
| C | -4.36695000 | 3.59198100 | -0.57358600 |
| H | -6.32830300 | 3.23214800 | 0.22915300 |
| H | -6.00105000 | 0.74882900 | 0.23493200 |
| C | -1.64216500 | 1.19139200 | -1.40366000 |
| H | -2.38624900 | 3.73741900 | -1.37824800 |
| H | -1.05108800 | 1.81849500 | -2.07206000 |
| O | -3.87396300 | -0.48930000 | -0.59295300 |
| N | -1.16910100 | 0.09238500 | -0.95969400 |
| O | 0.11790800 | -0.15579300 | -1.51992700 |
| C | -4.22718400 | -2.56317100 | 0.48122800 |

| | | | |
|---|---|---|---|
| C | -5.36348100 | -3.21335500 | 0.90717500 |
| N | -3.59617600 | -3.48734100 | -0.27984600 |
| H | -6.16408600 | -2.84618600 | 1.53223300 |
| N | -5.35660800 | -4.47171500 | 0.40240700 |
| N | -4.28080600 | -4.62805200 | -0.31579200 |
| C | -3.72712700 | -1.17313200 | 0.66205400 |
| H | -4.31320100 | -0.67583400 | 1.44039100 |
| H | -2.66694600 | -1.15236100 | 0.94281500 |
| C | -2.28184900 | -3.38930400 | -0.91497700 |
| H | -2.32717400 | -4.01848200 | -1.80637000 |
| H | -2.14395800 | -2.34862300 | -1.22215400 |
| C | -1.18350600 | -3.83112900 | 0.02593500 |
| C | -1.06193400 | -5.17500200 | 0.38797600 |
| C | -0.29636600 | -2.89309100 | 0.55147000 |
| C | -0.05897400 | -5.57305100 | 1.26640900 |
| H | -1.75772200 | -5.90512500 | -0.01779900 |
| C | 0.71300800 | -3.29224900 | 1.42772200 |
| H | -0.38741900 | -1.84762800 | 0.27176400 |
| C | 0.83157200 | -4.63203700 | 1.78714300 |
| H | 0.03260400 | -6.61892000 | 1.54301900 |
| H | 1.40323400 | -2.54906300 | 1.81788100 |
| H | 1.61589500 | -4.94743600 | 2.46847000 |
| N | -4.56216000 | 5.04277000 | -0.61092500 |
| O | -3.65339700 | 5.73062900 | -1.04261500 |
| O | -5.62416300 | 5.48308100 | -0.20687000 |

**CZ**

| | | | |
|---|---|---|---|
| C | -2.36708500 | -1.77850500 | -0.35297700 |
| C | -1.19304300 | -1.02972400 | -0.36626100 |
| C | -1.23894300 | 0.34937600 | -0.17051600 |
| C | -2.46461400 | 1.04720000 | 0.04281100 |
| C | -3.63205900 | 0.27122200 | 0.05346000 |
| C | -3.55983500 | -1.10643500 | -0.14247800 |
| H | -2.35080200 | -2.85019200 | -0.50198000 |

| | | | |
|---|---|---|---|
| H | -0.25121200 | -1.53991700 | -0.52972200 |
| C | -2.44041500 | 2.48770000 | 0.23063400 |
| H | -4.57442800 | 0.77074600 | 0.21577700 |
| O | -0.12219200 | 1.12614000 | -0.17036800 |
| C | 2.16966300 | 1.54195500 | -0.19993900 |
| C | 2.13239600 | 2.82216200 | 0.30252200 |
| N | 3.46293400 | 1.37082800 | -0.55718200 |
| H | 1.29369900 | 3.37131800 | 0.70239900 |
| N | 3.38205800 | 3.34852200 | 0.22697100 |
| N | 4.18265900 | 2.46680700 | -0.28932500 |
| C | 1.12421800 | 0.48488400 | -0.33920600 |
| H | 1.18364800 | 0.00274600 | -1.32525600 |
| H | 1.26429100 | -0.29367600 | 0.42352700 |
| N | -3.45039100 | 3.30469000 | 0.46042200 |
| O | -4.64872100 | 2.83044500 | 0.54931000 |
| H | -1.47535800 | 2.97827700 | 0.17224600 |
| C | 4.09167900 | 0.19916000 | -1.16179300 |
| H | 3.72675900 | 0.09143000 | -2.18755500 |
| H | 5.15843200 | 0.43251400 | -1.19994600 |
| C | 3.81151900 | -1.04956900 | -0.35774500 |
| C | 3.23630300 | -2.16585300 | -0.96329500 |
| C | 4.09380000 | -1.07593600 | 1.01067300 |
| C | 2.94368000 | -3.30180800 | -0.20881400 |
| H | 3.00763300 | -2.14491700 | -2.02607700 |
| C | 3.79903100 | -2.20719200 | 1.76444000 |
| H | 4.53479300 | -0.20151300 | 1.48397600 |
| C | 3.22124600 | -3.32199900 | 1.15534800 |
| H | 2.49197900 | -4.16510700 | -0.68714100 |
| H | 4.01743600 | -2.21956500 | 2.82759400 |
| H | 2.98574300 | -4.20230100 | 1.74497100 |
| N | -4.79815800 | -1.87727000 | -0.12620500 |
| O | -4.73166300 | -3.08701000 | -0.29710400 |
| O | -5.85137100 | -1.28516300 | 0.05606600 |

**C1(CZ)**

| | | | |
|---|---|---|---|
| N | -8.39236100 | 1.07191700 | -2.27538500 |
| C | -7.17243700 | 0.40974100 | -1.81646200 |
| C | -7.46213800 | -1.03061700 | -1.43883900 |
| O | -7.47934900 | -1.44976900 | -0.30034600 |
| C | -6.61194100 | 1.21605800 | -0.64888700 |
| O | -5.53241000 | 0.55654100 | 0.02800200 |
| C | -2.96578800 | 2.42477900 | -0.66917400 |
| C | -2.19684200 | 3.28309800 | -1.64553300 |
| C | -2.53337900 | -1.50332000 | 0.45116300 |
| C | -1.37449700 | -1.27280300 | 1.38988500 |
| O | -3.69441200 | 1.44201800 | -1.44570900 |
| O | -3.34187200 | -0.29691900 | 0.45870200 |
| O | -4.47125100 | -0.95988900 | -1.80426600 |
| P | -4.21470300 | 0.09524100 | -0.80130100 |
| H | -6.28087300 | 2.19527300 | -1.00560400 |
| H | -7.38413500 | 1.35763800 | 0.11050900 |
| H | -8.68077200 | 0.71029800 | -3.18118000 |
| H | -9.15854000 | 0.90178000 | -1.62588700 |
| H | -6.46285000 | 0.39847400 | -2.64975500 |
| H | -7.75403100 | -1.68533100 | -2.28090300 |
| H | -3.69241500 | 3.00417000 | -0.08870100 |
| H | -2.29083500 | 1.89694400 | 0.00627800 |
| H | -1.48339200 | 2.65028900 | -2.17878300 |
| H | -2.87323200 | 3.75931700 | -2.36112800 |
| H | -1.65154600 | 4.05832100 | -1.10143200 |
| H | -0.78770200 | -0.42546900 | 1.02397000 |
| H | -1.72741200 | -1.06796100 | 2.40538800 |
| H | -0.73836400 | -2.16217100 | 1.41522900 |
| H | -2.18313700 | -1.69490100 | -0.56198900 |
| H | -3.17741400 | -2.32436600 | 0.78582500 |
| C | -4.86780700 | -1.36779400 | 2.89431000 |
| N | -5.77819900 | -1.58123600 | 1.88570200 |
| C | -4.75240300 | -2.54350000 | 3.56987400 |

| | | | |
|---|---|---|---|
| C | -6.21140100 | -2.83420600 | 1.92714200 |
| N | -5.59855800 | -3.43438200 | 2.94540000 |
| H | -4.15573200 | -2.82132900 | 4.42254100 |
| H | -6.92688600 | -3.27283900 | 1.25009600 |
| H | -5.74027900 | -4.40199200 | 3.21182000 |
| H | -4.38356500 | -0.41491500 | 3.03086700 |
| H | -6.07195000 | -0.89968000 | 1.17686400 |
| C | 2.73236700 | 2.54227300 | 1.26806500 |
| C | 3.49301800 | 1.46740400 | 0.81656600 |
| C | 2.91480500 | 0.49187400 | 0.00583300 |
| C | 1.54637400 | 0.54934000 | -0.39149800 |
| C | 0.80449600 | 1.64532700 | 0.06857900 |
| C | 1.40269300 | 2.60270400 | 0.88566500 |
| H | 3.16189900 | 3.30497900 | 1.90471200 |
| H | 4.53374700 | 1.40534100 | 1.11147700 |
| C | 1.00117900 | -0.51831700 | -1.21769300 |
| H | -0.23003100 | 1.72129800 | -0.23019900 |
| O | 3.60917000 | -0.57995800 | -0.45797700 |
| C | 5.49404600 | -1.90893600 | -0.78299600 |
| C | 5.01672700 | -2.65837800 | -1.83300000 |
| N | 6.66077100 | -2.51014300 | -0.45777500 |
| H | 4.10604800 | -2.53409700 | -2.39877900 |
| N | 5.90478600 | -3.65422200 | -2.08672500 |
| N | 6.89800600 | -3.55780800 | -1.25619700 |
| C | 4.97085300 | -0.68970600 | -0.09827200 |
| H | 5.07718400 | -0.77593600 | 0.99206600 |
| H | 5.52902200 | 0.20002700 | -0.42131000 |
| N | -0.22295900 | -0.65647300 | -1.67636700 |
| O | -1.10528100 | 0.26143900 | -1.41729900 |
| H | 1.67116600 | -1.32853600 | -1.48262500 |
| C | 7.60803700 | -2.14545800 | 0.59281700 |
| H | 7.15029200 | -2.34319100 | 1.56666500 |
| H | 8.45337900 | -2.82613900 | 0.46677000 |
| C | 8.02249500 | -0.69665100 | 0.47587000 |

| | | | |
|---|---|---|---|
| C | 7.85920500 | 0.17537800 | 1.55122500 |
| C | 8.53732500 | -0.21501300 | -0.73097600 |
| C | 8.20662300 | 1.52003700 | 1.42421500 |
| H | 7.45224600 | -0.19538800 | 2.48886300 |
| C | 8.88149100 | 1.12642900 | -0.85922300 |
| H | 8.65584500 | -0.89478300 | -1.57186600 |
| C | 8.71485200 | 1.99673500 | 0.21884600 |
| H | 8.07387800 | 2.19368100 | 2.26489800 |
| H | 9.27740600 | 1.49527500 | -1.80013200 |
| H | 8.97942200 | 3.04447700 | 0.11667600 |
| N | 0.59107200 | 3.71118800 | 1.37219700 |
| O | -0.61390000 | 3.68779800 | 1.16305800 |
| O | 1.14786400 | 4.61649800 | 1.97675000 |

**TS1(CZ)**

| | | | |
|---|---|---|---|
| N | -7.61670100 | 2.73165700 | -1.17365600 |
| C | -6.72450700 | 1.58313500 | -1.14219600 |
| C | -5.90468400 | 1.64585500 | 0.16068300 |
| O | -5.04555100 | 0.52962800 | 0.31029600 |
| C | -7.42567700 | 0.24203000 | -1.22640800 |
| O | -6.86292100 | -0.79809300 | -1.49765000 |
| O | -3.73884100 | 1.17062900 | -1.75714500 |
| P | -3.52201400 | 0.59315700 | -0.41019900 |
| C | -2.08490800 | 0.95866500 | 1.77889300 |
| O | -3.28297200 | -0.96136100 | -0.11571600 |
| C | -3.22160100 | -1.84277400 | -1.25979100 |
| C | -2.75450100 | -3.19329700 | -0.77038900 |
| H | -8.07819800 | 2.79405700 | -2.07785900 |
| H | -6.01247000 | 1.63903200 | -1.97034600 |
| H | -5.34178200 | 2.58518800 | 0.16219400 |
| H | -6.57758000 | 1.65148000 | 1.02518500 |
| H | -8.51267300 | 0.25102300 | -1.00075700 |
| H | -2.72622900 | 0.25971300 | 2.32849800 |
| H | -1.23593400 | 0.41131200 | 1.36180800 |

| | | | |
|---|---|---|---|
| H | -4.22490900 | -1.89373300 | -1.70174900 |
| H | -2.52383600 | -1.41728000 | -1.98201900 |
| H | -2.68323100 | -3.88766300 | -1.61224100 |
| H | -3.44841000 | -3.61272400 | -0.03471900 |
| H | -1.76827200 | -3.08245400 | -0.31444500 |
| C | -5.51941700 | -3.92739100 | 2.05641700 |
| N | -6.30820500 | -4.18808200 | 0.95774800 |
| C | -5.15190100 | -2.62054000 | 1.95881300 |
| C | -6.42628000 | -3.08659600 | 0.21678300 |
| N | -5.73109700 | -2.12796300 | 0.81378500 |
| H | -4.53056700 | -2.00791000 | 2.59088300 |
| H | -6.98519400 | -2.98019700 | -0.69946100 |
| H | -5.59790700 | -1.16395200 | 0.46151100 |
| H | -5.29554200 | -4.68138700 | 2.79247400 |
| H | -8.35534000 | 2.62635000 | -0.47913800 |
| H | -6.74236800 | -5.07722700 | 0.73838500 |
| O | -2.83537900 | 1.52570300 | 0.69124200 |
| C | -1.63742300 | 2.10139800 | 2.66391100 |
| H | -2.49998700 | 2.62890600 | 3.08027900 |
| H | -1.03190800 | 1.71667000 | 3.48912900 |
| H | -1.03757900 | 2.81234600 | 2.08818700 |
| C | 2.90894700 | 3.08248100 | 0.51909200 |
| C | 3.53610300 | 1.84562700 | 0.42307000 |
| C | 2.79423700 | 0.71089800 | 0.09067000 |
| C | 1.39531000 | 0.77305700 | -0.15842700 |
| C | 0.78574300 | 2.02779100 | -0.06093500 |
| C | 1.54509900 | 3.14492600 | 0.28035000 |
| H | 3.46755100 | 3.97388800 | 0.77398200 |
| H | 4.60195600 | 1.78407700 | 0.60668400 |
| C | 0.67522500 | -0.46941300 | -0.44694200 |
| H | -0.27447300 | 2.10850300 | -0.25429300 |
| O | 3.34973700 | -0.52162200 | -0.01761600 |
| C | 5.07473500 | -2.08208900 | -0.04309700 |
| C | 4.38755700 | -3.10352700 | -0.65674500 |

| | | | |
|---|---|---|---|
| N | 6.24476600 | -2.65125100 | 0.32455000 |
| H | 3.40361900 | -3.09137400 | -1.10013200 |
| N | 5.16539800 | -4.21681500 | -0.63292900 |
| N | 6.28715900 | -3.93722300 | -0.04273300 |
| C | 4.74162100 | -0.64684400 | 0.19673000 |
| H | 5.00447200 | -0.34877900 | 1.22144300 |
| H | 5.29799400 | -0.00145600 | -0.49704300 |
| N | -0.57005900 | -0.62725200 | -0.79557000 |
| O | -1.28997200 | 0.45089400 | -1.01540200 |
| H | 1.22540600 | -1.39545200 | -0.31891200 |
| C | 7.37288100 | -2.03577000 | 1.01787200 |
| H | 7.07006800 | -1.79276600 | 2.04083100 |
| H | 8.14178500 | -2.81087300 | 1.06347900 |
| C | 7.85249400 | -0.80083600 | 0.28952700 |
| C | 7.95908700 | 0.41867400 | 0.95636100 |
| C | 8.15482800 | -0.87116600 | -1.07325000 |
| C | 8.36788400 | 1.56151900 | 0.26900600 |
| H | 7.71516800 | 0.47759600 | 2.01425900 |
| C | 8.55851100 | 0.26879500 | -1.75991700 |
| H | 8.06082900 | -1.82118600 | -1.59480100 |
| C | 8.66474900 | 1.48794400 | -1.08919100 |
| H | 8.44585100 | 2.50810100 | 0.79443900 |
| H | 8.78856800 | 0.20864100 | -2.81900100 |
| H | 8.97511400 | 2.37800700 | -1.62740200 |
| N | 0.87708300 | 4.43676000 | 0.38399100 |
| O | -0.33345000 | 4.48066800 | 0.21706300 |
| O | 1.55599100 | 5.42105800 | 0.63642900 |

**IN(CZ)**

| | | | |
|---|---|---|---|
| N | -8.18723700 | -0.00566200 | -2.02256400 |
| C | -7.12182800 | -0.75324300 | -1.36823000 |
| C | -5.86069000 | 0.12094300 | -1.29855900 |
| O | -4.82035600 | -0.61799600 | -0.71876900 |
| C | -7.45090600 | -1.18485400 | 0.04255700 |

| | | | |
|---|---|---|---|
| O | -7.03347000 | -2.19722900 | 0.56398400 |
| O | -3.24480200 | -0.04108200 | -2.54268200 |
| P | -3.14689300 | -0.05784500 | -1.04684600 |
| C | -2.93103700 | 2.49519200 | -0.06032800 |
| O | -2.67039500 | -1.36171200 | -0.17650600 |
| C | -3.19562000 | -2.66671900 | -0.43756300 |
| C | -2.17084900 | -3.66538800 | 0.06123900 |
| H | -9.02798700 | -0.57502100 | -2.08900700 |
| H | -6.88240400 | -1.64830100 | -1.95058500 |
| H | -5.60155400 | 0.42089700 | -2.31928000 |
| H | -6.07175200 | 1.03182600 | -0.71450000 |
| H | -8.08451100 | -0.47358500 | 0.61552200 |
| H | -3.54131800 | 3.07521100 | 0.63863300 |
| H | -1.92516800 | 2.41348600 | 0.35760400 |
| H | -4.15669600 | -2.79210400 | 0.07212200 |
| H | -3.37181400 | -2.78344300 | -1.51245600 |
| H | -2.52165600 | -4.68724200 | -0.10866300 |
| H | -2.00163800 | -3.53027700 | 1.13374700 |
| H | -1.21965200 | -3.52676600 | -0.45979300 |
| C | -3.65908400 | -0.55739900 | 3.94198400 |
| N | -4.85553800 | -1.22591500 | 4.07875400 |
| C | -3.52258500 | -0.28886300 | 2.61496600 |
| C | -5.42953200 | -1.36352300 | 2.88312400 |
| N | -4.63516000 | -0.79769900 | 1.98372100 |
| H | -2.74294900 | 0.20778500 | 2.06145300 |
| H | -6.36700400 | -1.85551100 | 2.67338100 |
| H | -4.80606500 | -0.74973700 | 0.94161700 |
| H | -3.02755200 | -0.33625100 | 4.78609900 |
| H | -8.43667800 | 0.80856800 | -1.46196700 |
| H | -5.24799400 | -1.56715300 | 4.94814400 |
| O | -3.55929900 | 1.20907700 | -0.08899500 |
| C | -2.91990700 | 3.16347700 | -1.42263700 |
| H | -3.93630000 | 3.20812600 | -1.82671500 |
| H | -2.53066000 | 4.18095000 | -1.33435000 |

| | | | |
|---|---|---|---|
| H | -2.29421200 | 2.60373400 | -2.12277800 |
| C | 2.66186600 | 3.09272500 | 0.69130600 |
| C | 3.27722500 | 1.86237600 | 0.50589800 |
| C | 2.59686900 | 0.84229800 | -0.16657900 |
| C | 1.28930100 | 1.03999400 | -0.67068700 |
| C | 0.69428100 | 2.28389200 | -0.49150000 |
| C | 1.38198000 | 3.28223800 | 0.19028700 |
| H | 3.16539300 | 3.89286000 | 1.21920900 |
| H | 4.27658600 | 1.70962600 | 0.89468600 |
| C | 0.64586000 | -0.06406800 | -1.40586300 |
| H | -0.29000500 | 2.47507500 | -0.89496300 |
| O | 3.11736400 | -0.38743400 | -0.37211100 |
| C | 4.76846700 | -2.02937500 | -0.34851600 |
| C | 4.16818500 | -2.90014900 | -1.22809600 |
| N | 5.83374500 | -2.71961900 | 0.11779300 |
| H | 3.27865900 | -2.76273900 | -1.82364700 |
| N | 4.89104200 | -4.04942500 | -1.25186600 |
| N | 5.89766900 | -3.93378300 | -0.44059000 |
| C | 4.44487700 | -0.63075800 | 0.06076100 |
| H | 4.51902100 | -0.51253900 | 1.15001700 |
| H | 5.13564500 | 0.08230000 | -0.40896900 |
| N | -0.59960000 | -0.33454700 | -1.53908000 |
| O | -1.45084100 | 0.50764600 | -0.89780600 |
| H | 1.30540300 | -0.77067700 | -1.90177700 |
| C | 6.84028000 | -2.29264600 | 1.08665200 |
| H | 6.36910100 | -2.19898300 | 2.06946000 |
| H | 7.56268500 | -3.11120800 | 1.12991000 |
| C | 7.48258500 | -0.98987300 | 0.66891800 |
| C | 7.47707400 | 0.10951500 | 1.52621100 |
| C | 8.05514900 | -0.87291900 | -0.60079700 |
| C | 8.04052300 | 1.31916000 | 1.12030300 |
| H | 7.02572900 | 0.02196400 | 2.51157800 |
| C | 8.61443800 | 0.33375600 | -1.00709500 |
| H | 8.05107700 | -1.72934300 | -1.27131400 |

| | | | |
|---|---|---|---|
| C | 8.60657000 | 1.43247600 | -0.14658000 |
| H | 8.03036000 | 2.17174600 | 1.79180200 |
| H | 9.05522300 | 0.42017700 | -1.99522900 |
| H | 9.03937600 | 2.37501200 | -0.46685900 |
| N | 0.73036000 | 4.57050700 | 0.39153300 |
| O | -0.42110600 | 4.69923700 | 0.00417600 |
| O | 1.36375500 | 5.45731800 | 0.94116000 |

**TS2(CZ)**

| | | | |
|---|---|---|---|
| N | -8.07774400 | 0.17838600 | -2.07317600 |
| C | -7.04416900 | -0.65192600 | -1.46995700 |
| C | -5.84665300 | 0.23013300 | -1.08908400 |
| O | -4.81893900 | -0.57154800 | -0.55836200 |
| C | -7.48987500 | -1.37455800 | -0.21701500 |
| O | -7.10423200 | -2.47400700 | 0.11504800 |
| O | -3.23940900 | -0.06655900 | -2.42867100 |
| P | -3.02295000 | -0.02587000 | -0.95163700 |
| C | -2.82068800 | 2.52560000 | -0.01226700 |
| O | -2.62367900 | -1.33379900 | -0.07799200 |
| C | -3.15182000 | -2.62739100 | -0.40694900 |
| C | -2.05730500 | -3.64324200 | -0.15191100 |
| H | -8.86974700 | -0.39542100 | -2.35433100 |
| H | -6.69683100 | -1.39949300 | -2.18994700 |
| H | -5.49572400 | 0.73783000 | -1.99193400 |
| H | -6.15803100 | 0.99423500 | -0.35868000 |
| H | -8.18447800 | -0.79638800 | 0.43156900 |
| H | -3.41100900 | 3.10550100 | 0.70274000 |
| H | -1.80155900 | 2.44703000 | 0.37192400 |
| H | -4.03432800 | -2.82101000 | 0.21154000 |
| H | -3.46736500 | -2.63831700 | -1.45434600 |
| H | -2.41598800 | -4.65019900 | -0.38336200 |
| H | -1.74760800 | -3.61808800 | 0.89664200 |
| H | -1.18721900 | -3.42453900 | -0.77667300 |
| C | -4.47030300 | -0.35156800 | 4.05019800 |

| | | | |
|---|---|---|---|
| N | -5.41624700 | -1.34476100 | 3.94852000 |
| C | -4.19397800 | 0.03194200 | 2.77144600 |
| C | -5.69566600 | -1.54866400 | 2.65413800 |
| N | -4.96809700 | -0.72232800 | 1.91907600 |
| H | -3.51034000 | 0.77460900 | 2.39299200 |
| H | -6.40411200 | -2.27051600 | 2.27561800 |
| H | -4.91979600 | -0.66205700 | 0.70231600 |
| H | -4.09148100 | -0.01652100 | 5.00164500 |
| H | -8.43570400 | 0.84309000 | -1.38786700 |
| H | -5.83896800 | -1.84439600 | 4.72072500 |
| O | -3.44582700 | 1.23193700 | -0.00641900 |
| C | -2.86198200 | 3.17796800 | -1.38079400 |
| H | -3.89065600 | 3.20200200 | -1.75370800 |
| H | -2.48694700 | 4.20209000 | -1.31254800 |
| H | -2.24667800 | 2.62536100 | -2.09635300 |
| C | 2.75915300 | 3.08826900 | 0.63177100 |
| C | 3.35966200 | 1.84651900 | 0.47922500 |
| C | 2.67278200 | 0.82132100 | -0.17939200 |
| C | 1.37185200 | 1.02620300 | -0.69636000 |
| C | 0.79261500 | 2.28194200 | -0.55320900 |
| C | 1.48749200 | 3.28544800 | 0.11250000 |
| H | 3.26776600 | 3.89253700 | 1.14847400 |
| H | 4.35270400 | 1.68898500 | 0.88193800 |
| C | 0.71741600 | -0.08854100 | -1.40668500 |
| H | -0.18431800 | 2.48090900 | -0.97082500 |
| O | 3.17950200 | -0.41730900 | -0.35797600 |
| C | 4.82515200 | -2.06631600 | -0.31510200 |
| C | 4.23425500 | -2.93560100 | -1.20244400 |
| N | 5.88178900 | -2.75963800 | 0.16618900 |
| H | 3.35416900 | -2.79549100 | -1.81122900 |
| N | 4.95331400 | -4.08743800 | -1.21510700 |
| N | 5.94903700 | -3.97442000 | -0.39019200 |
| C | 4.50313300 | -0.66516900 | 0.08616900 |
| H | 4.56879900 | -0.54143300 | 1.17521600 |

| | | | |
|---|---|---|---|
| H | 5.20001400 | 0.04282500 | -0.38193600 |
| N | -0.52836200 | -0.35959200 | -1.51706300 |
| O | -1.36059600 | 0.51190700 | -0.86941200 |
| H | 1.36694400 | -0.80652600 | -1.89953400 |
| C | 6.87488000 | -2.33677700 | 1.15090100 |
| H | 6.39155600 | -2.25447500 | 2.12881100 |
| H | 7.60064400 | -3.15231500 | 1.19524500 |
| C | 7.51577200 | -1.02704700 | 0.75334500 |
| C | 7.49753300 | 0.06276500 | 1.62262600 |
| C | 8.09923400 | -0.89333900 | -0.50979700 |
| C | 8.05875800 | 1.27942700 | 1.23514100 |
| H | 7.03777700 | -0.03760300 | 2.60286400 |
| C | 8.65604000 | 0.32051400 | -0.89783900 |
| H | 8.10499300 | -1.74210300 | -1.19000200 |
| C | 8.63535000 | 1.40956400 | -0.02544600 |
| H | 8.03873200 | 2.12431400 | 1.91613700 |
| H | 9.10487200 | 0.41993900 | -1.88110600 |
| H | 9.06638300 | 2.35766400 | -0.33138400 |
| N | 0.85166300 | 4.58688900 | 0.27743800 |
| O | -0.29126600 | 4.72369000 | -0.13174000 |
| O | 1.48925900 | 5.47462200 | 0.81988400 |

**C2(CZ)**

| | | | |
|---|---|---|---|
| N | -9.11680800 | -1.18723000 | -0.78077000 |
| C | -7.65935300 | -1.26928400 | -0.73108900 |
| C | -7.08158200 | 0.14479300 | -0.57866200 |
| O | -5.67757800 | 0.15658300 | -0.64036700 |
| C | -7.17285700 | -2.12502000 | 0.41870900 |
| O | -6.34770400 | -3.00719600 | 0.32874600 |
| O | -3.06394000 | -0.21236100 | -3.08600800 |
| P | -2.66623600 | 0.25739000 | -1.74891500 |
| C | -3.19712800 | 2.15793700 | 0.00700500 |
| O | -2.90067600 | -0.73617400 | -0.53802700 |
| C | -3.56477200 | -2.01075000 | -0.73477000 |

| | | | |
|---|---|---|---|
| C | -2.58748000 | -3.04468400 | -1.24981200 |
| H | -9.51850300 | -2.11610600 | -0.88953300 |
| H | -7.28251700 | -1.70281000 | -1.66322000 |
| H | -7.47736900 | 0.75421400 | -1.39702800 |
| H | -7.44247500 | 0.58011300 | 0.36744100 |
| H | -7.64440700 | -1.88693500 | 1.39715700 |
| H | -3.33652300 | 1.32081300 | 0.69769700 |
| H | -2.20228500 | 2.58931100 | 0.15831900 |
| H | -3.95121600 | -2.26897300 | 0.25155300 |
| H | -4.41498700 | -1.86754400 | -1.40319300 |
| H | -3.08622700 | -4.01586700 | -1.31953300 |
| H | -1.73304200 | -3.13676600 | -0.57398300 |
| H | -2.22116600 | -2.76646300 | -2.24151800 |
| C | -4.69939000 | 0.18544900 | 4.18036900 |
| N | -4.76820600 | -1.17974600 | 4.05808300 |
| C | -4.94795600 | 0.67157800 | 2.92629700 |
| C | -5.04823100 | -1.47051900 | 2.76864500 |
| N | -5.16243300 | -0.36960200 | 2.05158800 |
| H | -4.99256200 | 1.70179400 | 2.60248900 |
| H | -5.17675800 | -2.47935400 | 2.40019200 |
| H | -5.35024700 | -0.08217700 | 0.26140200 |
| H | -4.49067100 | 0.66795600 | 5.12163200 |
| H | -9.47507800 | -0.82268300 | 0.10180200 |
| H | -4.63780200 | -1.85289200 | 4.80128200 |
| O | -3.26532300 | 1.66218400 | -1.34999100 |
| C | -4.28930300 | 3.19031300 | 0.16864700 |
| H | -4.23755600 | 3.63220400 | 1.16785300 |
| H | -4.17349800 | 3.98992800 | -0.56764200 |
| H | -5.26421100 | 2.71529200 | 0.03202600 |
| C | 2.99230200 | 3.07179200 | 0.39066900 |
| C | 3.58192600 | 1.81874600 | 0.33128200 |
| C | 2.89762200 | 0.75458400 | -0.26586900 |
| C | 1.60046000 | 0.93408900 | -0.81449300 |
| C | 1.02992000 | 2.20514900 | -0.75686800 |

| | | | |
|---|---|---|---|
| C | 1.72751700 | 3.24236300 | -0.15441900 |
| H | 3.50300000 | 3.90534900 | 0.85667700 |
| H | 4.56857300 | 1.68229700 | 0.75578500 |
| C | 0.96064300 | -0.24836900 | -1.41484600 |
| H | 0.05778900 | 2.39882300 | -1.18186700 |
| O | 3.40626200 | -0.49013400 | -0.35007500 |
| C | 5.03939800 | -2.14320200 | -0.14240600 |
| C | 4.48887200 | -3.06255900 | -1.00486500 |
| N | 6.06305600 | -2.81154800 | 0.43644600 |
| H | 3.64701000 | -2.95626300 | -1.67154800 |
| N | 5.19663800 | -4.21743900 | -0.90713400 |
| N | 6.14790800 | -4.05864100 | -0.03880700 |
| C | 4.71309000 | -0.71756100 | 0.15619000 |
| H | 4.73906600 | -0.52420600 | 1.23656300 |
| H | 5.43055800 | -0.04488800 | -0.33180600 |
| N | -0.24712600 | -0.47225600 | -1.76693800 |
| O | -1.08221700 | 0.63516100 | -1.56457000 |
| H | 1.59874300 | -1.11170600 | -1.57600200 |
| C | 7.00824800 | -2.33414100 | 1.44352700 |
| H | 6.47906000 | -2.20025500 | 2.39170600 |
| H | 7.73133500 | -3.14392200 | 1.56625200 |
| C | 7.66695000 | -1.04578600 | 1.00697100 |
| C | 7.60772200 | 0.08819400 | 1.81604600 |
| C | 8.30724400 | -0.97626800 | -0.23337500 |
| C | 8.18425100 | 1.28501300 | 1.39113800 |
| H | 7.10316000 | 0.03800700 | 2.77791600 |
| C | 8.87939100 | 0.21781200 | -0.65903400 |
| H | 8.34429700 | -1.85897100 | -0.86785900 |
| C | 8.81730100 | 1.35118200 | 0.15299800 |
| H | 8.13286600 | 2.16411800 | 2.02560600 |
| H | 9.37240300 | 0.26705200 | -1.62482300 |
| H | 9.26001600 | 2.28376000 | -0.18245800 |
| N | 1.09684800 | 4.55726500 | -0.08620800 |
| O | -0.03693100 | 4.67496400 | -0.52267900 |

| | | | |
|---|---|---|---|
| O | 1.73520500 | 5.47149800 | 0.40830200 |

**DE**

| | | | |
|---|---|---|---|
| C | -4.53306600 | -0.28947800 | -1.43724000 |
| C | -3.56958800 | 0.70989700 | -1.43075100 |
| C | -2.69495900 | 0.86888800 | -0.35761700 |
| C | -2.74268900 | 0.01891900 | 0.77714900 |
| C | -3.71954600 | -0.99027400 | 0.75176600 |
| C | -4.57665700 | -1.12670900 | -0.32888000 |
| H | -5.22007500 | -0.42350800 | -2.26229800 |
| H | -3.47842400 | 1.40003700 | -2.26330700 |
| C | -1.90694600 | 0.11831900 | 1.96121300 |
| H | -3.81190900 | -1.67037300 | 1.59088400 |
| H | -2.11305200 | -0.58051100 | 2.77789700 |
| O | -1.80914000 | 1.91378700 | -0.43161300 |
| N | -0.93992300 | 0.98579000 | 2.09761400 |
| O | -0.28790200 | 0.95887600 | 3.21907400 |
| C | 0.37700200 | 2.72843200 | -0.80188800 |
| C | 0.40453200 | 3.80829000 | -1.65802300 |
| N | 1.34281100 | 3.01760700 | 0.10457100 |
| H | -0.20626800 | 3.99860600 | -2.52859300 |
| N | 1.36259600 | 4.67121500 | -1.24533100 |
| N | 1.93266200 | 4.18004100 | -0.18265500 |
| C | -0.48772200 | 1.51218100 | -0.80342300 |
| H | -0.51799800 | 1.08875200 | -1.81485600 |
| H | -0.11007600 | 0.76244900 | -0.10500000 |
| C | 1.83552700 | 2.22728000 | 1.23359500 |
| H | 2.60238800 | 2.85683700 | 1.69328900 |
| H | 1.03285200 | 2.05286100 | 1.95665000 |
| C | 2.42682800 | 0.91233400 | 0.77511300 |
| C | 3.32371000 | 0.87031000 | -0.29447100 |
| C | 2.07758900 | -0.26968900 | 1.42955000 |
| C | 3.87127400 | -0.33740600 | -0.71315600 |
| H | 3.59428700 | 1.78898500 | -0.80999700 |

| | | | |
|---|---|---|---|
| C | 2.62172700 | -1.48654600 | 1.02216200 |
| H | 1.36273000 | -0.22194200 | 2.24928600 |
| C | 3.50986800 | -1.50384200 | -0.04589500 |
| H | 4.56495200 | -0.37496800 | -1.54592100 |
| H | 2.34933400 | -2.41033600 | 1.52111600 |
| N | -5.57671800 | -2.19460300 | -0.29703000 |
| O | -5.58982100 | -2.94726300 | 0.66382900 |
| O | -6.35277300 | -2.28330600 | -1.23549200 |
| Br | 4.23191500 | -3.16141200 | -0.62859800 |

**C1(DE)**

| | | | |
|---|---|---|---|
| N | -6.87215100 | -2.80668900 | -2.15055900 |
| C | -5.47396500 | -2.62836500 | -1.76389600 |
| C | -5.25154800 | -1.13662400 | -1.52368800 |
| O | -4.01597200 | -0.83870700 | -0.85526800 |
| C | -5.14723900 | -3.48516200 | -0.55557200 |
| O | -4.94116700 | -3.06555900 | 0.56517700 |
| O | -2.52916900 | -2.80037300 | -1.74436400 |
| P | -2.60447500 | -1.35603600 | -1.44391200 |
| C | -2.37153400 | 0.97654300 | -2.65829000 |
| O | -1.68609100 | -0.83460800 | -0.25906000 |
| C | -0.73401700 | -1.77312200 | 0.31678800 |
| C | 0.05918800 | -1.04562300 | 1.37404000 |
| H | -7.02341000 | -3.72796800 | -2.55342500 |
| H | -4.84764200 | -2.98052900 | -2.58813500 |
| H | -5.30017600 | -0.60138400 | -2.47643400 |
| H | -6.03020300 | -0.74763800 | -0.86329500 |
| H | -5.19185500 | -4.57487400 | -0.73772900 |
| H | -3.36093200 | 1.30383100 | -2.99039100 |
| H | -2.22816400 | 1.28982800 | -1.62075700 |
| H | -1.30076700 | -2.60793000 | 0.74200400 |
| H | -0.08639900 | -2.13280700 | -0.48306000 |
| H | 0.71673100 | -1.75778800 | 1.88167000 |
| H | -0.60239200 | -0.58887200 | 2.11838600 |

| | | | |
|---|---|---|---|
| H | 0.67774000 | -0.26909700 | 0.91692400 |
| C | -2.89248100 | 0.45301100 | 3.40349100 |
| N | -2.79995800 | -0.83270900 | 3.88840000 |
| C | -3.51315200 | 0.36167700 | 2.19420200 |
| C | -3.34074500 | -1.68195500 | 3.01563100 |
| N | -3.77670500 | -0.97013600 | 1.98544400 |
| H | -3.78924300 | 1.12046400 | 1.47897300 |
| H | -3.41318000 | -2.75279000 | 3.12358600 |
| H | -4.22237800 | -1.38091600 | 1.15640200 |
| H | -2.50865000 | 1.30321700 | 3.94303500 |
| H | -7.48366700 | -2.72598200 | -1.33988900 |
| H | -2.38458000 | -1.10275900 | 4.77304400 |
| O | -2.35196500 | -0.47146300 | -2.73229000 |
| C | -1.26358900 | 1.50354500 | -3.54186800 |
| H | -1.45182300 | 1.24526100 | -4.58795200 |
| H | -1.21093500 | 2.59352000 | -3.45585100 |
| H | -0.31411200 | 1.06149600 | -3.22351400 |
| C | 6.03594300 | -2.52333500 | 1.05519900 |
| C | 5.80162500 | -1.26985200 | 0.50795600 |
| C | 4.64464600 | -1.00366100 | -0.22361000 |
| C | 3.64980000 | -1.98980800 | -0.42520400 |
| C | 3.90902300 | -3.25814000 | 0.11810400 |
| C | 5.06920500 | -3.49821000 | 0.83712800 |
| H | 6.93157700 | -2.74669800 | 1.61965800 |
| H | 6.52977200 | -0.47267600 | 0.62064500 |
| C | 2.41789700 | -1.81008400 | -1.17875700 |
| H | 3.18982800 | -4.05809800 | -0.01599000 |
| H | 1.89456700 | -2.71363600 | -1.50771400 |
| O | 4.53634300 | 0.23754500 | -0.79849000 |
| N | 1.88883100 | -0.64396600 | -1.42508800 |
| O | 0.76324000 | -0.64248300 | -2.07576500 |
| C | 4.17367200 | 2.56123400 | -0.56680600 |
| C | 5.20127500 | 3.47323600 | -0.46236400 |
| N | 3.34051900 | 3.12675900 | -1.47233700 |

| | | | |
|---|---|---|---|
| H | 6.09350200 | 3.42561700 | 0.14468800 |
| N | 4.93907000 | 4.52192800 | -1.27909400 |
| N | 3.80513900 | 4.30570700 | -1.88377900 |
| C | 3.95232800 | 1.22501700 | 0.05550800 |
| H | 4.44234500 | 1.20277100 | 1.03544300 |
| H | 2.88406800 | 1.01043600 | 0.17394100 |
| C | 2.02250400 | 2.66302900 | -1.89454500 |
| H | 1.81167100 | 3.17703500 | -2.83616500 |
| H | 2.07275600 | 1.58357300 | -2.07010900 |
| C | 0.97277200 | 2.96474900 | -0.84635400 |
| C | 0.88438900 | 4.22183300 | -0.24238900 |
| C | 0.08097900 | 1.95840800 | -0.48161200 |
| C | -0.09571600 | 4.48104900 | 0.71201500 |
| H | 1.58521500 | 5.00671600 | -0.51699800 |
| C | -0.89675400 | 2.20387100 | 0.48035500 |
| H | 0.14690600 | 0.97791400 | -0.96093300 |
| C | -0.97731600 | 3.46162400 | 1.05860700 |
| H | -0.17411300 | 5.45722700 | 1.17768900 |
| H | -1.57331400 | 1.40658100 | 0.76834600 |
| N | 5.28486900 | -4.83535600 | 1.39205900 |
| O | 4.42954600 | -5.68528100 | 1.20368400 |
| O | 6.31218300 | -5.03706200 | 2.01987000 |
| Br | -2.33479000 | 3.79238200 | 2.35014000 |

**TS1(DE)**

| | | | |
|---|---|---|---|
| N | 3.47979300 | -5.84473300 | 1.27909900 |
| C | 2.89107700 | -4.65507200 | 0.66919000 |
| C | 3.24453300 | -3.41719700 | 1.47934900 |
| O | 2.64100400 | -2.27919100 | 0.88622900 |
| C | 3.32106100 | -4.52501400 | -0.78091500 |
| O | 3.85246300 | -3.55232300 | -1.27761400 |
| O | 0.48027200 | -2.86531400 | 2.02923500 |
| P | 1.21099200 | -1.68578900 | 1.49675800 |
| C | 1.49008200 | -0.01536900 | 3.66481900 |

| | | | |
|---|---|---|---|
| O | 0.81637600 | -0.96911600 | 0.11063200 |
| C | 0.66774300 | -1.79066700 | -1.06142100 |
| C | -0.08913100 | -0.98529700 | -2.09479600 |
| H | 3.08379100 | -6.69111900 | 0.87727200 |
| H | 1.79855200 | -4.76243100 | 0.68400200 |
| H | 2.91485600 | -3.55929700 | 2.51217400 |
| H | 4.32891800 | -3.25527800 | 1.47497200 |
| H | 3.16876600 | -5.43716100 | -1.38965300 |
| H | 2.39678400 | 0.44333400 | 4.06706400 |
| H | 0.76003700 | 0.75670700 | 3.42856200 |
| H | 1.65839900 | -2.08162600 | -1.42492100 |
| H | 0.12119300 | -2.70399100 | -0.79633000 |
| H | -0.24421600 | -1.58762500 | -2.99431500 |
| H | 0.47249300 | -0.08848800 | -2.37088400 |
| H | -1.06255200 | -0.68132800 | -1.69947500 |
| C | 4.53679500 | 1.39488000 | -0.48718500 |
| N | 4.84647300 | 1.04148700 | -1.78178600 |
| C | 4.07633300 | 0.26657400 | 0.11998700 |
| C | 4.59473400 | -0.25350500 | -1.96624100 |
| N | 4.12589300 | -0.73419900 | -0.82216700 |
| H | 3.69025500 | 0.08562100 | 1.11240600 |
| H | 4.74336200 | -0.80668200 | -2.87995600 |
| H | 3.83727900 | -1.70792900 | -0.65777100 |
| H | 4.64315400 | 2.40725800 | -0.13303700 |
| H | 4.48276800 | -5.87645400 | 1.10387800 |
| H | 5.18724500 | 1.67222000 | -2.49842300 |
| O | 1.93661500 | -0.60809800 | 2.42812300 |
| C | 0.92464000 | -1.03366900 | 4.63300500 |
| H | 1.64874200 | -1.83279100 | 4.82056700 |
| H | 0.70153100 | -0.53962400 | 5.58357800 |
| H | 0.00912800 | -1.46386100 | 4.22558400 |
| C | -5.61403800 | -1.75103000 | -1.68949400 |
| C | -5.41393100 | -0.58411300 | -0.96602600 |
| C | -4.28820600 | -0.41459700 | -0.16049000 |

| | | | |
|---|---|---|---|
| C | -3.30268900 | -1.42410900 | -0.04761200 |
| C | -3.51719800 | -2.59705600 | -0.78470300 |
| C | -4.64416200 | -2.73952700 | -1.57907500 |
| H | -6.48348200 | -1.89582600 | -2.31758900 |
| H | -6.13614200 | 0.22492700 | -1.00515900 |
| C | -2.09673100 | -1.37056400 | 0.77792000 |
| H | -2.79613000 | -3.40566400 | -0.73408300 |
| H | -1.49890900 | -2.28237200 | 0.83708400 |
| O | -4.20558700 | 0.75751900 | 0.54535200 |
| N | -1.73857300 | -0.30783500 | 1.42445900 |
| O | -0.62552800 | -0.35360600 | 2.12123700 |
| C | -3.52543400 | 3.02006500 | 0.68432500 |
| C | -4.51656400 | 3.97757000 | 0.69715600 |
| N | -2.64115500 | 3.46901600 | 1.60840200 |
| H | -5.42716800 | 4.01729500 | 0.11736000 |
| N | -4.18871500 | 4.93605200 | 1.59557200 |
| N | -3.04595900 | 4.62285900 | 2.13846300 |
| C | -3.39165600 | 1.75090000 | -0.08792200 |
| H | -3.75400700 | 1.91296800 | -1.10969000 |
| H | -2.35123500 | 1.41257400 | -0.11806300 |
| C | -1.33615800 | 2.92168900 | 1.97794600 |
| H | -1.07016100 | 3.42273600 | 2.91197700 |
| H | -1.43657700 | 1.84751700 | 2.15643400 |
| C | -0.30503200 | 3.17712100 | 0.90012700 |
| C | -0.02973600 | 4.47197200 | 0.45385500 |
| C | 0.37230700 | 2.09541300 | 0.34143200 |
| C | 0.92540100 | 4.68922700 | -0.53470200 |
| H | -0.56182700 | 5.31856400 | 0.88159300 |
| C | 1.31609800 | 2.29700900 | -0.66338800 |
| H | 0.17184800 | 1.09064500 | 0.70391200 |
| C | 1.58765800 | 3.59247000 | -1.08055600 |
| H | 1.15229600 | 5.69265000 | -0.87785400 |
| H | 1.82858500 | 1.44883900 | -1.10519500 |
| Br | 2.90130400 | 3.87780900 | -2.42450500 |

| | | | |
|---|---|---|---|
| N | -4.82012800 | -3.98374100 | -2.32954000 |
| O | -5.80055600 | -4.08476800 | -3.04912300 |
| O | -3.97984200 | -4.85907100 | -2.20091100 |

**IN(DE)**

| | | | |
|---|---|---|---|
| N | 4.62082700 | -5.34813800 | 2.00494100 |
| C | 3.98480100 | -4.51561200 | 0.99361900 |
| C | 3.15289500 | -3.42766800 | 1.68804800 |
| O | 2.48894600 | -2.66634000 | 0.71148100 |
| C | 4.95828700 | -3.82701600 | 0.06267200 |
| O | 4.71399200 | -3.54097800 | -1.09048600 |
| O | 0.32078500 | -2.92424400 | 1.86357000 |
| P | 1.04132000 | -1.78017500 | 1.20968800 |
| C | 1.80474300 | 0.28001100 | 2.89826800 |
| O | 0.85492100 | -1.37930600 | -0.37089000 |
| C | 0.93646800 | -2.35963800 | -1.41155600 |
| C | 0.18683200 | -1.79679900 | -2.60122200 |
| H | 5.15774500 | -6.09028900 | 1.56191300 |
| H | 3.30647200 | -5.12242300 | 0.38551400 |
| H | 2.43385600 | -3.92169300 | 2.34840500 |
| H | 3.80764200 | -2.78936200 | 2.30260600 |
| H | 5.93172800 | -3.54875600 | 0.52069700 |
| H | 2.78827600 | 0.70981900 | 3.11071600 |
| H | 1.19272700 | 1.04150900 | 2.40903800 |
| H | 1.98463000 | -2.56339900 | -1.65222400 |
| H | 0.48733700 | -3.29993700 | -1.06787600 |
| H | 0.22046100 | -2.49731800 | -3.44016600 |
| H | 0.63796200 | -0.85116600 | -2.91767200 |
| H | -0.85974100 | -1.60989100 | -2.33984200 |
| C | 3.97135800 | 1.41523300 | -1.34077100 |
| N | 4.92354600 | 0.60646500 | -1.92140100 |
| C | 3.24470900 | 0.61254300 | -0.51587400 |
| C | 4.78558100 | -0.64150300 | -1.47365200 |
| N | 3.77270800 | -0.65378200 | -0.61634500 |

| | | | |
|---|---|---|---|
| H | 2.39796300 | 0.82002500 | 0.11826500 |
| H | 5.37744500 | -1.49732000 | -1.75916900 |
| H | 3.39045700 | -1.49416100 | -0.11341700 |
| H | 3.88402800 | 2.46405200 | -1.57986100 |
| H | 5.28648100 | -4.79569000 | 2.54455600 |
| H | 5.62317600 | 0.90210400 | -2.59191200 |
| O | 2.08636100 | -0.78334300 | 1.97880500 |
| C | 1.15729700 | -0.21798300 | 4.17545700 |
| H | 1.77559100 | -0.99496100 | 4.63540600 |
| H | 1.05419000 | 0.60696700 | 4.88701200 |
| H | 0.16724400 | -0.62810900 | 3.96631800 |
| C | -5.83617600 | -1.96880200 | -1.29891400 |
| C | -5.54720000 | -0.87358400 | -0.49867300 |
| C | -4.28561600 | -0.71496900 | 0.07677000 |
| C | -3.26466000 | -1.66521700 | -0.13903700 |
| C | -3.56776900 | -2.77049000 | -0.93929000 |
| C | -4.82698200 | -2.90059900 | -1.50443300 |
| H | -6.81088800 | -2.10351500 | -1.75029000 |
| H | -6.30096200 | -0.11978600 | -0.29673200 |
| C | -1.90670600 | -1.61929500 | 0.43091500 |
| H | -2.81744600 | -3.53170900 | -1.12331900 |
| H | -1.31996500 | -2.53160500 | 0.33874600 |
| O | -4.09650600 | 0.37260600 | 0.88551300 |
| N | -1.47336000 | -0.56458400 | 1.01233700 |
| O | -0.22050800 | -0.52060600 | 1.51755700 |
| C | -3.65544500 | 2.69703700 | 1.10328800 |
| C | -4.63999900 | 3.61932500 | 1.38386300 |
| N | -2.63518000 | 3.06771800 | 1.91501100 |
| H | -5.63319400 | 3.69977900 | 0.96680400 |
| N | -4.17626100 | 4.48264100 | 2.31865300 |
| N | -2.95777000 | 4.14249100 | 2.63095200 |
| C | -3.62910400 | 1.52871900 | 0.17866100 |
| H | -4.29467000 | 1.73188100 | -0.66745500 |
| H | -2.61872100 | 1.34029200 | -0.19876300 |

| | | | |
|---|---|---|---|
| C | -1.28512500 | 2.51089200 | 2.00407200 |
| H | -0.91578000 | 2.79087700 | 2.99346300 |
| H | -1.36176400 | 1.42482700 | 1.94101800 |
| C | -0.39157600 | 3.06168500 | 0.91380600 |
| C | 0.01151600 | 4.39922200 | 0.95827300 |
| C | 0.02659600 | 2.25460100 | -0.14540600 |
| C | 0.83768600 | 4.92731200 | -0.02692300 |
| H | -0.31814900 | 5.03212400 | 1.77831900 |
| C | 0.83452900 | 2.77809400 | -1.15472400 |
| H | -0.25754400 | 1.20502300 | -0.17766800 |
| C | 1.24238000 | 4.10248300 | -1.07252200 |
| H | 1.16847900 | 5.95900400 | 0.01702500 |
| H | 1.15377800 | 2.15535000 | -1.98396800 |
| N | -5.10273600 | -4.06929000 | -2.34180300 |
| O | -4.20360700 | -4.87371700 | -2.51920900 |
| O | -6.21924500 | -4.17766600 | -2.82005400 |
| Br | 2.40748300 | 4.79389400 | -2.40130800 |

**TS2(DE)**

| | | | |
|---|---|---|---|
| N | 3.60044000 | -5.99898100 | 1.16990200 |
| C | 2.95435400 | -4.96798200 | 0.37131200 |
| C | 2.53564200 | -3.81284300 | 1.29173200 |
| O | 1.90757000 | -2.81241900 | 0.51813200 |
| C | 3.82535200 | -4.39655900 | -0.73316800 |
| O | 3.39938400 | -3.97844800 | -1.78670400 |
| O | -0.30341700 | -3.05841000 | 1.65865800 |
| P | 0.46307700 | -1.83200600 | 1.27820700 |
| C | 1.50875400 | -0.42693400 | 3.38040600 |
| O | 0.36072600 | -1.18383400 | -0.20877200 |
| C | 0.26285900 | -1.99064100 | -1.39355500 |
| C | -0.54010100 | -1.19803000 | -2.40434600 |
| H | 3.84192500 | -6.79655500 | 0.58612600 |
| H | 2.04846600 | -5.37021100 | -0.09331600 |
| H | 1.83429000 | -4.19328400 | 2.03759000 |

| | | | |
|---|---|---|---|
| H | 3.41314600 | -3.39698300 | 1.80972100 |
| H | 4.91099200 | -4.34883500 | -0.49862700 |
| H | 2.53606000 | -0.18334600 | 3.66471700 |
| H | 0.97907200 | 0.49849800 | 3.15078700 |
| H | 1.26781200 | -2.21598800 | -1.76100300 |
| H | -0.22377400 | -2.94089000 | -1.14795300 |
| H | -0.61699700 | -1.75481600 | -3.34223600 |
| H | -0.05492000 | -0.23872200 | -2.60763900 |
| H | -1.55000400 | -1.00199400 | -2.03075500 |
| C | 4.83760500 | 0.68898500 | -0.24909900 |
| N | 4.80540900 | 0.12056900 | -1.50024900 |
| C | 4.06434600 | -0.11078100 | 0.54015500 |
| C | 4.04370600 | -0.98291400 | -1.45527000 |
| N | 3.58019100 | -1.14535600 | -0.22639500 |
| H | 3.80983400 | -0.01825100 | 1.58342500 |
| H | 3.86345200 | -1.64306100 | -2.29046500 |
| H | 2.72581200 | -2.01695600 | 0.14466400 |
| H | 5.37521800 | 1.60184400 | -0.05064000 |
| H | 4.47727200 | -5.64723200 | 1.55319900 |
| H | 5.26150100 | 0.48486300 | -2.32736900 |
| O | 1.66038300 | -1.20656900 | 2.18005200 |
| C | 0.82686200 | -1.21164700 | 4.48377900 |
| H | 1.36095900 | -2.14878100 | 4.66636800 |
| H | 0.82669500 | -0.62673900 | 5.40799700 |
| H | -0.20644800 | -1.43930000 | 4.21320300 |
| C | -6.25403500 | -0.62978700 | -1.23861800 |
| C | -5.68109100 | 0.44541800 | -0.57601200 |
| C | -4.43429500 | 0.32850800 | 0.03948600 |
| C | -3.72325100 | -0.88992200 | 0.00924400 |
| C | -4.30479400 | -1.96657300 | -0.66607000 |
| C | -5.54259300 | -1.82190000 | -1.27261600 |
| H | -7.22040100 | -0.55273600 | -1.72036100 |
| H | -6.19134400 | 1.40080300 | -0.51889500 |
| C | -2.41707100 | -1.14644900 | 0.63987900 |

| | | | |
|---|---|---|---|
| H | -3.79278200 | -2.92105800 | -0.71734600 |
| H | -2.02363800 | -2.15673900 | 0.52644400 |
| O | -3.95398100 | 1.43034400 | 0.68955300 |
| N | -1.80221900 | -0.24663500 | 1.30848500 |
| O | -0.58878800 | -0.55480200 | 1.85959800 |
| C | -2.86896000 | 3.53264500 | 0.58424300 |
| C | -3.58260900 | 4.71070700 | 0.55456500 |
| N | -1.88745600 | 3.77972900 | 1.48512900 |
| H | -4.46083600 | 4.95771000 | -0.02362200 |
| N | -3.00691300 | 5.59105300 | 1.40792400 |
| N | -1.97632400 | 5.01893100 | 1.96378600 |
| C | -3.07437500 | 2.23214400 | -0.11257500 |
| H | -3.54025000 | 2.41809200 | -1.08585600 |
| H | -2.12747700 | 1.70177200 | -0.26477600 |
| C | -0.75726100 | 2.93112100 | 1.85913200 |
| H | -0.44028300 | 3.27789000 | 2.84519800 |
| H | -1.11813600 | 1.90243900 | 1.93982800 |
| C | 0.36495300 | 3.03428200 | 0.84904800 |
| C | 1.01965800 | 4.24997900 | 0.63557500 |
| C | 0.74442900 | 1.91173900 | 0.11633200 |
| C | 2.04771000 | 4.34243400 | -0.29625500 |
| H | 0.72310500 | 5.13090500 | 1.19908500 |
| C | 1.77235500 | 1.98807300 | -0.82229500 |
| H | 0.24521900 | 0.96219000 | 0.27625200 |
| C | 2.41090800 | 3.20557700 | -1.01453800 |
| H | 2.56289600 | 5.28164700 | -0.46378400 |
| H | 2.06620400 | 1.10704300 | -1.38668700 |
| Br | 3.81714300 | 3.33302700 | -2.28513800 |
| N | -6.12057900 | -2.97130800 | -1.97217600 |
| O | -7.21274800 | -2.82936800 | -2.49484000 |
| O | -5.48050100 | -4.00861500 | -1.99620800 |

**C2(DE)**

| | | | |
|---|---|---|---|
| N | 1.18343000 | 6.28564200 | 0.47569900 |

| | | | |
|---|---|---|---|
| C | 1.16339700 | 4.91028800 | 0.97463800 |
| C | 2.27664000 | 4.11487300 | 0.30581700 |
| O | 2.35506000 | 2.85634900 | 0.93298300 |
| C | 1.36028000 | 4.85663500 | 2.47577100 |
| O | 0.56266700 | 4.40488800 | 3.26600400 |
| O | -0.31862700 | 2.59167500 | -0.68549900 |
| P | 0.30689900 | 1.25742200 | -0.63825000 |
| C | 2.46929600 | -0.08036000 | -1.20753100 |
| O | 0.45862200 | 0.58071100 | 0.78637400 |
| C | 0.08812000 | 1.29034400 | 1.99444200 |
| C | -1.24289500 | 0.77692900 | 2.49828900 |
| H | 0.40699600 | 6.81138500 | 0.87150000 |
| H | 0.20132700 | 4.44942000 | 0.73310200 |
| H | 2.05494700 | 4.03286400 | -0.76705600 |
| H | 3.22629100 | 4.66657600 | 0.41680200 |
| H | 2.30896900 | 5.31781300 | 2.83066300 |
| H | 2.96355700 | -0.00734300 | -0.23349300 |
| H | 1.78930400 | -0.93718800 | -1.20020700 |
| H | 0.89524500 | 1.10136500 | 2.70435400 |
| H | 0.05821700 | 2.35963800 | 1.78985700 |
| H | -1.51188400 | 1.30136200 | 3.41998300 |
| H | -1.19423700 | -0.29445900 | 2.71456600 |
| H | -2.03018300 | 0.95289800 | 1.75748900 |
| C | 6.57847100 | 0.90636500 | -0.86256500 |
| N | 6.38984300 | 2.01526700 | -1.64843700 |
| C | 5.58416700 | 0.95638000 | 0.07618700 |
| C | 5.31238700 | 2.68204000 | -1.17568300 |
| N | 4.79872800 | 2.06627100 | -0.12990300 |
| H | 5.37462200 | 0.25363300 | 0.86897400 |
| H | 4.94639600 | 3.59594400 | -1.62132300 |
| H | 3.19385400 | 2.44547000 | 0.60877300 |
| H | 7.37832600 | 0.20546500 | -1.04029300 |
| H | 2.03514000 | 6.75465400 | 0.78442800 |
| H | 6.95477200 | 2.28768000 | -2.44156600 |

| | | | |
|---|---|---|---|
| O | 1.68940800 | 1.12809000 | -1.39752400 |
| C | 3.47280400 | -0.19448100 | -2.32979500 |
| H | 4.13037000 | -1.04519800 | -2.12438300 |
| H | 2.97108200 | -0.35323600 | -3.28793900 |
| H | 4.08626300 | 0.70845800 | -2.39024800 |
| C | -6.80447600 | 0.30735300 | -0.12333600 |
| C | -5.99400400 | -0.80891600 | 0.01672600 |
| C | -4.65610200 | -0.76877300 | -0.37613200 |
| C | -4.10310800 | 0.40675100 | -0.91974700 |
| C | -4.92811000 | 1.52056800 | -1.08468900 |
| C | -6.25006300 | 1.45525400 | -0.67620000 |
| H | -7.84526400 | 0.29324000 | 0.17517100 |
| H | -6.38794200 | -1.73637100 | 0.41809600 |
| C | -2.70274400 | 0.54216800 | -1.35063600 |
| H | -4.54281800 | 2.43695900 | -1.51746400 |
| H | -2.46760400 | 1.32497200 | -2.07359300 |
| O | -3.92329500 | -1.91630500 | -0.28603700 |
| N | -1.77775000 | -0.19475900 | -0.87155600 |
| O | -0.53055900 | 0.12226900 | -1.47309900 |
| C | -2.87315600 | -3.56610500 | 1.05140200 |
| C | -3.21780200 | -4.60843100 | 1.88166700 |
| N | -1.96816900 | -4.11669400 | 0.20620600 |
| H | -3.91497800 | -4.61872900 | 2.70627900 |
| N | -2.51758000 | -5.70886500 | 1.50916100 |
| N | -1.76634800 | -5.40251500 | 0.49236900 |
| C | -3.31769700 | -2.14627200 | 0.99578000 |
| H | -4.04455100 | -1.97039300 | 1.79524300 |
| H | -2.47834500 | -1.45136500 | 1.11072600 |
| C | -1.21686700 | -3.48566300 | -0.87621600 |
| H | -1.32916000 | -4.11073000 | -1.76451200 |
| H | -1.69135700 | -2.51928400 | -1.06525300 |
| C | 0.24103500 | -3.31564900 | -0.51815300 |
| C | 1.23555000 | -3.78360000 | -1.37554100 |
| C | 0.60738400 | -2.67400100 | 0.66735800 |

| | | | |
|---|---|---|---|
| C | 2.58290200 | -3.60185800 | -1.07000800 |
| H | 0.96223500 | -4.29215400 | -2.29568800 |
| C | 1.94608900 | -2.49682700 | 0.99567400 |
| H | -0.15605800 | -2.29444700 | 1.34180100 |
| C | 2.91841500 | -2.95386700 | 0.11163400 |
| H | 3.35662800 | -3.95192800 | -1.74451300 |
| H | 2.23041800 | -1.97676700 | 1.90393600 |
| N | -7.09592600 | 2.63984800 | -0.84536200 |
| O | -6.59389800 | 3.63271400 | -1.34226900 |
| O | -8.25533900 | 2.56663600 | -0.47827400 |
| Br | 4.74315900 | -2.59639900 | 0.47703200 |

**DZ**

| | | | |
|---|---|---|---|
| C | 2.34259600 | -1.83742800 | 0.83681000 |
| C | 1.51732200 | -0.72014100 | 0.73908200 |
| C | 2.01486000 | 0.47582600 | 0.22498000 |
| C | 3.36507600 | 0.61245100 | -0.21339400 |
| C | 4.17488400 | -0.52691700 | -0.10739300 |
| C | 3.65383600 | -1.71152700 | 0.40880400 |
| H | 1.97413900 | -2.77482700 | 1.23255000 |
| H | 0.48877800 | -0.80199900 | 1.06961500 |
| C | 3.82076100 | 1.89005100 | -0.73415500 |
| H | 5.20010200 | -0.45477700 | -0.43537800 |
| O | 1.25120300 | 1.59600000 | 0.10651100 |
| C | -0.72309500 | 2.82785000 | 0.14023500 |
| C | -0.28872600 | 3.87937100 | -0.63259000 |
| N | -1.94440300 | 3.22184600 | 0.56831900 |
| H | 0.64148800 | 3.98908600 | -1.16859300 |
| N | -1.25262500 | 4.83634500 | -0.63536500 |
| N | -2.25206200 | 4.43357900 | 0.08760600 |
| C | -0.10959400 | 1.50714300 | 0.47078700 |
| H | -0.21260900 | 1.28281800 | 1.54210100 |
| H | -0.60617800 | 0.70351500 | -0.09063800 |
| N | 5.01489900 | 2.21072200 | -1.19452600 |

| | | | |
|---|---|---|---|
| O | 5.94996800 | 1.31909800 | -1.21748800 |
| H | 3.10308500 | 2.70255300 | -0.75291100 |
| C | -2.88341600 | 2.50660900 | 1.42798600 |
| H | -2.47259900 | 2.45484800 | 2.44056000 |
| H | -3.78222900 | 3.12706600 | 1.45347600 |
| C | -3.16685900 | 1.12327500 | 0.88999300 |
| C | -2.97035100 | -0.00328800 | 1.68595600 |
| C | -3.58627000 | 0.96388700 | -0.43327800 |
| C | -3.18242000 | -1.28177700 | 1.17274900 |
| H | -2.63650800 | 0.10859800 | 2.71404800 |
| C | -3.79816500 | -0.30395700 | -0.96108100 |
| H | -3.73579300 | 1.83841800 | -1.06199200 |
| C | -3.58876800 | -1.41506800 | -0.14860800 |
| H | -3.02228200 | -2.15928800 | 1.78909100 |
| H | -4.11773100 | -0.43032900 | -1.98944600 |
| N | 4.52936400 | -2.87440900 | 0.50442000 |
| O | 4.07130000 | -3.90883600 | 0.97020800 |
| O | 5.68311700 | -2.76943000 | 0.11601600 |
| Br | -3.84807400 | -3.14843100 | -0.87025500 |

**C1(DZ)**

| | | | |
|---|---|---|---|
| N | 8.98041600 | -1.46886000 | -2.64006200 |
| C | 7.85898400 | -0.73122000 | -2.06110900 |
| C | 8.30006800 | 0.66354200 | -1.66139600 |
| O | 8.42419700 | 1.04996500 | -0.51747600 |
| C | 7.32245800 | -1.54075900 | -0.88414300 |
| O | 6.35212100 | -0.83352000 | -0.10184900 |
| C | 3.57645300 | -2.44605400 | -0.72995900 |
| C | 2.63056800 | -3.12211100 | -1.69443100 |
| C | 3.61747000 | 1.43986800 | 0.67130300 |
| C | 2.47781400 | 1.26014100 | 1.64477800 |
| O | 4.36095900 | -1.49527300 | -1.48976100 |
| O | 4.26253500 | 0.15049500 | 0.51893600 |
| O | 5.30993400 | 0.85913100 | -1.77506100 |

| | | | |
|---|---|---|---|
| P | 5.02355700 | -0.22754600 | -0.81454700 |
| H | 6.89271800 | -2.47643900 | -1.25299200 |
| H | 8.13642400 | -1.77368300 | -0.19306200 |
| H | 9.22694400 | -1.09370700 | -3.55267800 |
| H | 9.80597200 | -1.38859100 | -2.04858600 |
| H | 7.09117500 | -0.62447400 | -2.83381000 |
| H | 8.60306800 | 1.31342600 | -2.50321400 |
| H | 4.26880300 | -3.15467000 | -0.26202600 |
| H | 3.02755600 | -1.90604100 | 0.04384000 |
| H | 1.96241300 | -2.36594600 | -2.11332100 |
| H | 3.18175000 | -3.61555200 | -2.49999800 |
| H | 2.03703500 | -3.86911000 | -1.16107000 |
| H | 1.76413100 | 0.54484200 | 1.22669500 |
| H | 2.84493700 | 0.89449200 | 2.60876700 |
| H | 1.97357400 | 2.21753000 | 1.80447900 |
| H | 3.25640400 | 1.77885400 | -0.29831500 |
| H | 4.37365700 | 2.13573300 | 1.05242100 |
| C | 6.12192600 | 0.67840400 | 2.82123700 |
| N | 6.97567600 | 1.12378900 | 1.83986700 |
| C | 6.03520100 | 1.67560600 | 3.74313300 |
| C | 7.40470600 | 2.34374600 | 2.13441600 |
| N | 6.84214100 | 2.69626600 | 3.28853000 |
| H | 5.48276400 | 1.75088000 | 4.66486800 |
| H | 8.08178900 | 2.93671100 | 1.54032600 |
| H | 6.98926500 | 3.58390100 | 3.75516800 |
| H | 5.64513800 | -0.28595200 | 2.76192900 |
| H | 7.24618100 | 0.61455900 | 0.99097800 |
| C | -2.11270900 | -2.03895900 | 1.21856900 |
| C | -2.75724500 | -0.87663800 | 0.80350600 |
| C | -2.06297400 | 0.08521200 | 0.07178500 |
| C | -0.69002600 | -0.07208600 | -0.27884300 |
| C | -0.06551400 | -1.25236500 | 0.14514900 |
| C | -0.77819600 | -2.19539700 | 0.88285900 |
| H | -2.63325900 | -2.79629200 | 1.79033400 |

| | | | |
|---|---|---|---|
| H | -3.79974300 | -0.73657300 | 1.06293200 |
| C | -0.02220200 | 0.98629500 | -1.02235800 |
| H | 0.97096700 | -1.40494900 | -0.11566400 |
| O | -2.64007700 | 1.23792700 | -0.35773900 |
| C | -4.37724700 | 2.74571400 | -0.70191600 |
| C | -3.79637200 | 3.47841000 | -1.71067400 |
| N | -5.48716400 | 3.45155200 | -0.38589500 |
| H | -2.88695500 | 3.28030800 | -2.25696400 |
| N | -4.57186700 | 4.56763000 | -1.95057400 |
| N | -5.59349400 | 4.54505900 | -1.15067700 |
| C | -4.00085800 | 1.45613600 | -0.04982800 |
| H | -4.15031600 | 1.50735100 | 1.03784600 |
| H | -4.62133600 | 0.63556600 | -0.43695800 |
| N | 1.21470800 | 1.02034500 | -1.46525500 |
| O | 1.98616400 | -0.00640900 | -1.26917300 |
| H | -0.59806100 | 1.87986800 | -1.23559500 |
| C | -6.49035400 | 3.16017500 | 0.63275000 |
| H | -6.03755400 | 3.27515600 | 1.62191000 |
| H | -7.25459600 | 3.93207400 | 0.51500500 |
| C | -7.06361000 | 1.77237700 | 0.46125400 |
| C | -7.07536800 | 0.87552100 | 1.52768700 |
| C | -7.56790700 | 1.37161600 | -0.77864700 |
| C | -7.59030200 | -0.40985600 | 1.36984600 |
| H | -6.67739700 | 1.17527700 | 2.49346000 |
| C | -8.08114200 | 0.09181800 | -0.95324900 |
| H | -7.55354900 | 2.06279800 | -1.61777400 |
| C | -8.08712900 | -0.78487900 | 0.12834700 |
| H | -7.59997600 | -1.10767300 | 2.19951600 |
| H | -8.47214400 | -0.22304600 | -1.91426900 |
| N | -0.08358400 | -3.39489400 | 1.33468100 |
| O | 1.12514000 | -3.46786200 | 1.16175100 |
| O | -0.73674300 | -4.27552200 | 1.87580400 |
| Br | -8.79376400 | -2.52876100 | -0.09983200 |

**TS1(DZ)**

| | | | |
|---|---|---|---|
| N | 8.33150600 | -3.10490900 | -1.24695900 |
| C | 7.48686900 | -1.92137700 | -1.20351800 |
| C | 6.68263200 | -1.95153900 | 0.11024100 |
| O | 5.87101300 | -0.80165200 | 0.26924000 |
| C | 8.24006700 | -0.60987700 | -1.29554600 |
| O | 7.71873300 | 0.44879400 | -1.57865200 |
| O | 4.48384600 | -1.48242400 | -1.72224100 |
| P | 4.32690300 | -0.81077000 | -0.41105000 |
| C | 2.87013600 | -0.94026200 | 1.79610700 |
| O | 4.17762800 | 0.76710000 | -0.19623600 |
| C | 4.22514800 | 1.59613900 | -1.37926300 |
| C | 3.83459900 | 2.99645600 | -0.96877700 |
| H | 8.78676800 | -3.17970000 | -2.15342900 |
| H | 6.76190700 | -1.94825500 | -2.02187500 |
| H | 6.08425000 | -2.86830000 | 0.11780200 |
| H | 7.36647800 | -1.98345800 | 0.96566500 |
| H | 9.32456700 | -0.65786200 | -1.06367900 |
| H | 3.54650000 | -0.25507900 | 2.32021600 |
| H | 2.06928900 | -0.36393900 | 1.32754600 |
| H | 5.24541600 | 1.55453300 | -1.78127200 |
| H | 3.52606100 | 1.18909000 | -2.11136900 |
| H | 3.84718300 | 3.65593900 | -1.84101200 |
| H | 4.52435000 | 3.39993900 | -0.22024900 |
| H | 2.82486000 | 2.97385900 | -0.55266500 |
| C | 6.52511600 | 3.65735900 | 1.96174700 |
| N | 7.33789200 | 3.86786700 | 0.86982100 |
| C | 6.10090800 | 2.36692800 | 1.87650000 |
| C | 7.41533200 | 2.75247500 | 0.14439200 |
| N | 6.67143000 | 1.83373000 | 0.74547100 |
| H | 5.44723200 | 1.79026500 | 2.50966500 |
| H | 7.97784600 | 2.60903300 | -0.76455800 |
| H | 6.49832300 | 0.87248500 | 0.40392900 |
| H | 6.32647900 | 4.42961500 | 2.68591800 |

| | | | |
|---|---|---|---|
| H | 9.07623100 | -3.03479000 | -0.55453300 |
| H | 7.81497400 | 4.73337700 | 0.64535800 |
| O | 3.60436000 | -1.61937800 | 0.76191100 |
| C | 2.32704100 | -1.99670800 | 2.73299900 |
| H | 3.14163800 | -2.55619900 | 3.20091100 |
| H | 1.73194400 | -1.52370700 | 3.51912500 |
| H | 1.69170500 | -2.69725300 | 2.18330600 |
| C | -2.34808700 | -2.55278400 | 0.83967700 |
| C | -2.82763700 | -1.26060800 | 0.65808300 |
| C | -1.97678600 | -0.26045300 | 0.18624600 |
| C | -0.60934500 | -0.51531900 | -0.11551200 |
| C | -0.14953800 | -1.82308600 | 0.06951400 |
| C | -1.01752700 | -2.80424300 | 0.54466900 |
| H | -2.99317000 | -3.34322200 | 1.20116700 |
| H | -3.86578300 | -1.05170100 | 0.88645300 |
| C | 0.23131900 | 0.60236300 | -0.54888700 |
| H | 0.88177100 | -2.04859600 | -0.16070000 |
| O | -2.38873500 | 1.01627800 | -0.01564900 |
| C | -3.91461500 | 2.77104400 | -0.09495400 |
| C | -3.17310300 | 3.61955200 | -0.88361900 |
| N | -4.96151600 | 3.52997800 | 0.30401400 |
| H | -2.24983200 | 3.42641400 | -1.40791500 |
| N | -3.79820300 | 4.82466900 | -0.92219500 |
| N | -4.87871200 | 4.76464700 | -0.20646000 |
| C | -3.73539600 | 1.33483000 | 0.27305900 |
| H | -3.95168600 | 1.16883000 | 1.33716200 |
| H | -4.41490300 | 0.70084800 | -0.31355000 |
| N | 1.48279400 | 0.59228300 | -0.91139100 |
| O | 2.09286800 | -0.56797300 | -0.99682200 |
| H | -0.22025800 | 1.58831800 | -0.52998600 |
| C | -6.07354100 | 3.16521600 | 1.17769700 |
| H | -5.69897000 | 3.04176800 | 2.19801700 |
| H | -6.74739600 | 4.02499800 | 1.16177100 |
| C | -6.75388000 | 1.90407900 | 0.69812100 |

| | | | |
|---|---|---|---|
| C | -6.88301700 | 0.80342900 | 1.54320200 |
| C | -7.22131700 | 1.81878300 | -0.61580900 |
| C | -7.46530600 | -0.37810400 | 1.08801300 |
| H | -6.51724300 | 0.85739700 | 2.56514700 |
| C | -7.80271300 | 0.64715500 | -1.08555000 |
| H | -7.11952600 | 2.67135600 | -1.28276700 |
| C | -7.91384400 | -0.44176400 | -0.22487100 |
| H | -7.55729100 | -1.23744800 | 1.74258500 |
| H | -8.16120900 | 0.57624100 | -2.10621800 |
| N | -0.50619300 | -4.15679300 | 0.73429700 |
| O | 0.67719400 | -4.36880900 | 0.51046700 |
| O | -1.28297300 | -5.02163300 | 1.11119800 |
| Br | -8.67793100 | -2.05297400 | -0.86731200 |

**IN(DZ)**

| | | | |
|---|---|---|---|
| N | -8.99809700 | 0.69523400 | -2.15635000 |
| C | -8.01949000 | -0.17127900 | -1.51342900 |
| C | -6.68552500 | 0.57900200 | -1.38759100 |
| O | -5.73508900 | -0.27328700 | -0.80949400 |
| C | -8.41689500 | -0.62245100 | -0.12640800 |
| O | -8.12253200 | -1.69584400 | 0.35562200 |
| O | -4.05747100 | 0.24838700 | -2.55863700 |
| P | -4.00927500 | 0.13147900 | -1.06461400 |
| C | -3.56472800 | 2.58827200 | 0.08034900 |
| O | -3.68986300 | -1.26279600 | -0.26420400 |
| C | -4.35045400 | -2.48992100 | -0.58945600 |
| C | -3.47498100 | -3.61464800 | -0.07467200 |
| H | -9.89131700 | 0.21536600 | -2.24240500 |
| H | -7.85210000 | -1.06354600 | -2.12519100 |
| H | -6.37296400 | 0.88559600 | -2.39109100 |
| H | -6.82538000 | 1.48670600 | -0.77733700 |
| H | -8.98014400 | 0.12894800 | 0.46828600 |
| H | -4.13200900 | 3.18558100 | 0.80077200 |
| H | -2.58624300 | 2.37298200 | 0.51530600 |

| | | | |
|---|---|---|---|
| H | -5.34306400 | -2.51680100 | -0.12834300 |
| H | -4.48705200 | -2.55769200 | -1.67444600 |
| H | -3.93838800 | -4.58290900 | -0.28398500 |
| H | -3.33934800 | -3.52176200 | 1.00722200 |
| H | -2.49129500 | -3.58437200 | -0.55099700 |
| C | -4.67069300 | -0.59431600 | 3.87012200 |
| N | -5.91599300 | -1.17845500 | 3.94459300 |
| C | -4.49161800 | -0.24399800 | 2.56762900 |
| C | -6.47848000 | -1.18714200 | 2.73595700 |
| N | -5.62873300 | -0.62072300 | 1.88896600 |
| H | -3.66803100 | 0.22969700 | 2.05936200 |
| H | -7.44753300 | -1.58823600 | 2.48019800 |
| H | -5.77595200 | -0.49133600 | 0.85186800 |
| H | -4.03729900 | -0.48560200 | 4.73463800 |
| H | -9.16867900 | 1.51711900 | -1.57770300 |
| H | -6.34786300 | -1.54852800 | 4.78287800 |
| O | -4.32407000 | 1.38156200 | -0.04738500 |
| C | -3.44207500 | 3.33465800 | -1.23526300 |
| H | -4.43492600 | 3.49702200 | -1.66697700 |
| H | -2.96596300 | 4.30458500 | -1.07054100 |
| H | -2.84488200 | 2.76385000 | -1.95112600 |
| C | 2.06534100 | 2.59057800 | 0.94670000 |
| C | 2.54917900 | 1.30988300 | 0.71953700 |
| C | 1.77802500 | 0.39712100 | -0.00613400 |
| C | 0.50830200 | 0.75309800 | -0.52137600 |
| C | 0.04302500 | 2.04337000 | -0.29389800 |
| C | 0.82128700 | 2.93418900 | 0.43791100 |
| H | 2.64070900 | 3.31162100 | 1.51379000 |
| H | 3.51873400 | 1.03578500 | 1.11685000 |
| C | -0.22543900 | -0.24294800 | -1.32399400 |
| H | -0.90802300 | 2.35718100 | -0.70097000 |
| O | 2.17115700 | -0.87160200 | -0.25588300 |
| C | 3.64749800 | -2.67282500 | -0.28642200 |
| C | 2.96570600 | -3.44623500 | -1.19655100 |

| | | | |
|---|---|---|---|
| N | 4.64538100 | -3.47894800 | 0.14332600 |
| H | 2.09220500 | -3.20042500 | -1.78029400 |
| N | 3.57715000 | -4.65682900 | -1.27215900 |
| N | 4.59246700 | -4.67005400 | -0.46489100 |
| C | 3.45918200 | -1.26821100 | 0.18227900 |
| H | 3.52564900 | -1.20636300 | 1.27683200 |
| H | 4.22662000 | -0.60923400 | -0.24579600 |
| N | -1.48718900 | -0.37992100 | -1.49908400 |
| O | -2.26904300 | 0.51006100 | -0.83456700 |
| H | 0.37235000 | -0.98672500 | -1.84287100 |
| C | 5.68882800 | -3.19401300 | 1.12334800 |
| H | 5.23154500 | -3.08997400 | 2.11145100 |
| H | 6.32491000 | -4.08222600 | 1.13360700 |
| C | 6.46667100 | -1.95185700 | 0.75483700 |
| C | 6.62527700 | -0.91635700 | 1.67390800 |
| C | 7.01951400 | -1.82740000 | -0.52251200 |
| C | 7.33455000 | 0.23410300 | 1.33300200 |
| H | 6.19193500 | -1.00092500 | 2.66687300 |
| C | 7.72566800 | -0.68486700 | -0.87880700 |
| H | 6.89223700 | -2.62724400 | -1.24773100 |
| C | 7.87638800 | 0.33412900 | 0.05820400 |
| H | 7.45998900 | 1.03882600 | 2.04837900 |
| H | 8.15619400 | -0.58467300 | -1.86868800 |
| N | 0.30836800 | 4.27674300 | 0.68254600 |
| O | -0.82923600 | 4.53329700 | 0.31881800 |
| O | 1.03839300 | 5.07866500 | 1.24257200 |
| Br | 8.84881900 | 1.88883000 | -0.41941600 |

**TS2(DZ)**

| | | | |
|---|---|---|---|
| N | -8.79608000 | 0.89542900 | -2.32053400 |
| C | -7.86937600 | -0.06172400 | -1.73241800 |
| C | -6.63578700 | 0.68774100 | -1.20742800 |
| O | -5.72404100 | -0.23391500 | -0.66117000 |
| C | -8.44858600 | -0.85124300 | -0.57751500 |

| | | | |
|---|---|---|---|
| O | -8.15116600 | -1.99573400 | -0.31375700 |
| O | -3.99227300 | 0.21209100 | -2.40070100 |
| P | -3.86584600 | 0.12947400 | -0.91460400 |
| C | -3.44486200 | 2.58926100 | 0.16953800 |
| O | -3.66696000 | -1.26486700 | -0.10917500 |
| C | -4.31462600 | -2.46870400 | -0.54794700 |
| C | -3.34511200 | -3.61116600 | -0.32516600 |
| H | -9.61022100 | 0.41231700 | -2.69397200 |
| H | -7.53200400 | -0.77258100 | -2.49323100 |
| H | -6.17562700 | 1.21817000 | -2.04531000 |
| H | -6.93811400 | 1.42795700 | -0.44932900 |
| H | -9.15643600 | -0.28511200 | 0.06718600 |
| H | -3.99614000 | 3.19304800 | 0.89551400 |
| H | -2.45231200 | 2.38773900 | 0.57721500 |
| H | -5.23907900 | -2.60400100 | 0.02278400 |
| H | -4.58455100 | -2.37740700 | -1.60418600 |
| H | -3.79624900 | -4.55654000 | -0.63934000 |
| H | -3.07969300 | -3.68809200 | 0.73287900 |
| H | -2.43120400 | -3.44483400 | -0.90168100 |
| C | -5.80718800 | -0.23381700 | 3.96708900 |
| N | -6.76879000 | -1.18638400 | 3.72299300 |
| C | -5.38863000 | 0.19522600 | 2.74257400 |
| C | -6.92068700 | -1.32234300 | 2.39886900 |
| N | -6.09449300 | -0.49218100 | 1.78164200 |
| H | -4.64629200 | 0.92811100 | 2.47066200 |
| H | -7.61166700 | -1.99853500 | 1.91744000 |
| H | -5.92844600 | -0.38006400 | 0.58223100 |
| H | -5.51685600 | 0.04389500 | 4.96682000 |
| H | -9.14404600 | 1.52967800 | -1.60186400 |
| H | -7.28451400 | -1.70416400 | 4.42349000 |
| O | -4.19849900 | 1.36827000 | 0.09038900 |
| C | -3.37281900 | 3.30485100 | -1.16618500 |
| H | -4.38068700 | 3.45981500 | -1.56347200 |
| H | -2.88970100 | 4.27658200 | -1.03922800 |

| | | | |
|---|---|---|---|
| H | -2.80221100 | 2.71935500 | -1.89246100 |
| C | 2.19319400 | 2.57864500 | 0.89988800 |
| C | 2.66959000 | 1.28817000 | 0.71807200 |
| C | 1.88752700 | 0.35011700 | 0.03650000 |
| C | 0.61333300 | 0.69242300 | -0.47589900 |
| C | 0.15734600 | 1.99399900 | -0.29865200 |
| C | 0.94618900 | 2.90912400 | 0.38912900 |
| H | 2.77752800 | 3.31778300 | 1.43333400 |
| H | 3.64207200 | 1.02617800 | 1.11577200 |
| C | -0.13847100 | -0.33267600 | -1.22340700 |
| H | -0.79514200 | 2.29761700 | -0.70919400 |
| O | 2.27355200 | -0.92788400 | -0.16968300 |
| C | 3.75856800 | -2.72574500 | -0.16062100 |
| C | 3.07466000 | -3.53039500 | -1.04141500 |
| N | 4.76366200 | -3.51349400 | 0.28730500 |
| H | 2.19649700 | -3.30784500 | -1.62744800 |
| N | 3.69139300 | -4.73999300 | -1.08321900 |
| N | 4.71224200 | -4.72310400 | -0.28282800 |
| C | 3.56905800 | -1.30705700 | 0.26326300 |
| H | 3.65031300 | -1.20765000 | 1.35380900 |
| H | 4.32697400 | -0.65985500 | -0.19787500 |
| N | -1.40235000 | -0.47097600 | -1.36810800 |
| O | -2.15842800 | 0.46172500 | -0.71288100 |
| H | 0.44430700 | -1.10110900 | -1.72284300 |
| C | 5.81393600 | -3.19343400 | 1.24958800 |
| H | 5.36585700 | -3.07012500 | 2.23960200 |
| H | 6.46039200 | -4.07376000 | 1.27450100 |
| C | 6.57094600 | -1.95094900 | 0.84057300 |
| C | 6.71487500 | -0.88396000 | 1.72553600 |
| C | 7.10772600 | -1.85351500 | -0.44595100 |
| C | 7.38851700 | 0.27344200 | 1.33900000 |
| H | 6.29450200 | -0.94658200 | 2.72554200 |
| C | 7.77899600 | -0.70492400 | -0.84754800 |
| H | 6.99202700 | -2.67915900 | -1.14366600 |

| | | | |
|---|---|---|---|
| C | 7.91043900 | 0.34810100 | 0.05416900 |
| H | 7.49845500 | 1.10522900 | 2.02537300 |
| H | 8.19393100 | -0.62537900 | -1.84590500 |
| N | 0.43941900 | 4.26192100 | 0.58638000 |
| O | -0.69748300 | 4.50919800 | 0.21416400 |
| O | 1.17309300 | 5.07941800 | 1.11745700 |
| Br | 8.81846000 | 1.92101700 | -0.48852800 |

**C2(DZ)**

| | | | |
|---|---|---|---|
| N | -10.13305100 | -0.34777000 | -0.93772800 |
| C | -8.69765400 | -0.60980200 | -0.88513300 |
| C | -7.96098200 | 0.69591100 | -0.55307100 |
| O | -6.56510800 | 0.54395700 | -0.59315600 |
| C | -8.33627100 | -1.65251500 | 0.15092200 |
| O | -7.59669900 | -2.59243900 | -0.04040000 |
| O | -3.94262900 | 0.19720700 | -3.03765200 |
| P | -3.53547700 | 0.47087000 | -1.64971400 |
| C | -3.94762900 | 2.19314400 | 0.30881200 |
| O | -3.89742400 | -0.62264200 | -0.56352900 |
| C | -4.66530800 | -1.80197100 | -0.91509800 |
| C | -3.76600700 | -2.86127200 | -1.51369900 |
| H | -10.63980600 | -1.20215300 | -1.15925600 |
| H | -8.35074100 | -0.96703300 | -1.86027800 |
| H | -8.25900900 | 1.44195100 | -1.29636700 |
| H | -8.29464300 | 1.05610500 | 0.43389500 |
| H | -8.81207100 | -1.49828400 | 1.14396300 |
| H | -4.21007600 | 1.30018600 | 0.88368800 |
| H | -2.91987800 | 2.48654700 | 0.54602300 |
| H | -5.10917500 | -2.12671900 | 0.02651100 |
| H | -5.47402400 | -1.51073100 | -1.58684600 |
| H | -4.34788700 | -3.76660200 | -1.70999400 |
| H | -2.95205800 | -3.10881000 | -0.82713800 |
| H | -3.33418200 | -2.51050600 | -2.45469500 |
| C | -5.71691900 | -0.13654200 | 4.20736400 |

| | | | |
|---|---|---|---|
| N | -5.91685600 | -1.45915300 | 3.90077000 |
| C | -5.88531200 | 0.53532100 | 3.02775900 |
| C | -6.19274100 | -1.54556300 | 2.58112900 |
| N | -6.18016400 | -0.35317400 | 2.01816800 |
| H | -5.81979000 | 1.59860100 | 2.84541700 |
| H | -6.41183000 | -2.47834800 | 2.07927200 |
| H | -6.29519500 | 0.16361400 | 0.27878800 |
| H | -5.48245600 | 0.19190400 | 5.20710200 |
| H | -10.46602600 | -0.04707600 | -0.02186900 |
| H | -5.87096100 | -2.23459300 | 4.54810100 |
| O | -4.00348100 | 1.87495500 | -1.10091900 |
| C | -4.92971300 | 3.31397300 | 0.56077800 |
| H | -4.88113600 | 3.62207900 | 1.60915000 |
| H | -4.69407000 | 4.18011100 | -0.06312700 |
| H | -5.94170200 | 2.96990800 | 0.33186200 |
| C | 2.41733500 | 2.64794100 | 0.47208700 |
| C | 2.87574800 | 1.34372400 | 0.36858000 |
| C | 2.07756900 | 0.37204000 | -0.24389700 |
| C | 0.79602800 | 0.69593200 | -0.76099700 |
| C | 0.35627700 | 2.01482200 | -0.65532800 |
| C | 1.16687600 | 2.95936100 | -0.04198000 |
| H | 3.01811100 | 3.41297100 | 0.94817300 |
| H | 3.84994600 | 1.09575500 | 0.77051800 |
| C | 0.03451300 | -0.39409200 | -1.39402500 |
| H | -0.60023000 | 2.31871200 | -1.05052600 |
| O | 2.45599900 | -0.91505500 | -0.37225700 |
| C | 3.91762400 | -2.72953700 | -0.23117500 |
| C | 3.30148800 | -3.55493300 | -1.14252000 |
| N | 4.84800600 | -3.52176000 | 0.34955600 |
| H | 2.49527500 | -3.33774500 | -1.82620000 |
| N | 3.88139800 | -4.78088400 | -1.06872200 |
| N | 4.81707300 | -4.75419300 | -0.17033500 |
| C | 3.73787700 | -1.28786500 | 0.11024500 |
| H | 3.79421100 | -1.12854400 | 1.19479900 |

| | | | |
|---|---|---|---|
| H | 4.51499800 | -0.67781800 | -0.36914900 |
| N | -1.19968100 | -0.49076700 | -1.71033200 |
| O | -1.92685800 | 0.66774000 | -1.40570600 |
| H | 0.58722800 | -1.29882600 | -1.62721300 |
| C | 5.79880000 | -3.18504900 | 1.40490100 |
| H | 5.25074600 | -3.01249600 | 2.33600800 |
| H | 6.41690800 | -4.07708100 | 1.53220800 |
| C | 6.62601900 | -1.97488900 | 1.03683300 |
| C | 6.71242200 | -0.88907200 | 1.90631000 |
| C | 7.29355800 | -1.92725400 | -0.18952400 |
| C | 7.45974700 | 0.23682900 | 1.56559000 |
| H | 6.19003300 | -0.91343200 | 2.85901300 |
| C | 8.04037200 | -0.81014400 | -0.54502800 |
| H | 7.22412600 | -2.76701100 | -0.87654900 |
| C | 8.11442800 | 0.26138300 | 0.34100200 |
| H | 7.52592800 | 1.08210000 | 2.24116100 |
| H | 8.55910300 | -0.76975500 | -1.49618200 |
| N | 0.67469100 | 4.32947100 | 0.07222300 |
| O | -0.45408100 | 4.56968900 | -0.32444400 |
| O | 1.41764300 | 5.16366300 | 0.56212400 |
| Br | 9.13042300 | 1.78690600 | -0.13820800 |

**EE**

| | | | |
|---|---|---|---|
| C | 4.07346300 | -0.86662900 | -0.97279100 |
| C | 2.81296900 | -1.44670700 | -0.81806200 |
| C | 1.87947100 | -0.94894200 | 0.07773500 |
| C | 2.16995600 | 0.18941900 | 0.87027600 |
| C | 3.44127000 | 0.75716800 | 0.70316900 |
| C | 4.38028100 | 0.25136700 | -0.19691500 |
| H | 4.77875000 | -1.28574500 | -1.67970600 |
| H | 2.53964600 | -2.32226800 | -1.40023100 |
| C | 1.27727300 | 0.79467800 | 1.85129000 |
| H | 3.71902400 | 1.62801800 | 1.29106900 |
| H | 1.68402000 | 1.60893900 | 2.46122300 |

| | | | |
|---|---|---|---|
| O | 0.67952800 | -1.62618800 | 0.18920600 |
| N | 0.03587500 | 0.43726400 | 2.02784300 |
| O | -0.63514200 | 1.07687700 | 2.94278000 |
| C | -1.61643000 | -1.80550400 | -0.34156700 |
| C | -1.98139600 | -3.06708700 | -0.76080000 |
| N | -2.68558000 | -1.39083300 | 0.38071400 |
| H | -1.42194100 | -3.76826900 | -1.36291600 |
| N | -3.22103100 | -3.34490800 | -0.29201600 |
| N | -3.64405100 | -2.31965700 | 0.39185200 |
| C | -0.36442600 | -1.02491600 | -0.57076300 |
| H | -0.10358100 | -1.06365700 | -1.63659100 |
| H | -0.49895100 | 0.01636300 | -0.26825100 |
| C | -2.92307500 | -0.10825100 | 1.04461500 |
| H | -3.90079900 | -0.22597700 | 1.52047900 |
| H | -2.16560600 | 0.06384600 | 1.81577500 |
| C | -2.94012000 | 1.04109700 | 0.06065800 |
| C | -3.63640600 | 0.94204200 | -1.14644800 |
| C | -2.26155500 | 2.22020800 | 0.37393100 |
| C | -3.65870800 | 2.01532600 | -2.03243200 |
| H | -4.15896000 | 0.01940000 | -1.39091200 |
| C | -2.29117900 | 3.29610700 | -0.51260000 |
| H | -1.70384100 | 2.26478400 | 1.30780100 |
| C | -2.98706800 | 3.19629700 | -1.71542300 |
| H | -4.20001900 | 1.93184400 | -2.97009500 |
| H | -1.76021400 | 4.21129100 | -0.26746600 |
| H | -3.00357300 | 4.03327200 | -2.40695400 |
| O | 5.57096900 | 0.91613800 | -0.24432000 |
| C | 6.53820400 | 0.44368900 | -1.16200900 |
| H | 6.16447500 | 0.48812900 | -2.19153900 |
| H | 7.40102200 | 1.10193200 | -1.06115500 |
| H | 6.83609000 | -0.58575900 | -0.93179000 |

**C1(EE)**

| | | | |
|---|---|---|---|
| N | 7.06698800 | 1.48403700 | -2.42466700 |

| | | | |
|---|---|---|---|
| C | 5.85067500 | 1.40193200 | -1.63538700 |
| C | 5.40396800 | -0.07057900 | -1.62309200 |
| O | 4.25724200 | -0.28962100 | -0.79525600 |
| C | 5.97424000 | 1.94828200 | -0.22125900 |
| O | 5.04068900 | 2.05124200 | 0.54657600 |
| O | 2.77879900 | 1.41246300 | -2.10130900 |
| P | 2.78887200 | 0.13671100 | -1.36155900 |
| C | 2.11289200 | -2.39792400 | -1.67246400 |
| O | 2.01488600 | 0.05644100 | 0.02037400 |
| C | 1.34868900 | 1.25989900 | 0.48793500 |
| C | 0.50278700 | 0.89546200 | 1.68262600 |
| H | 7.28785700 | 2.45359200 | -2.63599800 |
| H | 5.05864800 | 1.97810000 | -2.12479300 |
| H | 5.20334000 | -0.38301600 | -2.65237300 |
| H | 6.19348200 | -0.70684900 | -1.21547600 |
| H | 6.99137000 | 2.26528300 | 0.08604600 |
| H | 3.03362200 | -2.97822500 | -1.78268100 |
| H | 1.88638100 | -2.28479300 | -0.60874900 |
| H | 2.12371100 | 1.99055000 | 0.74654600 |
| H | 0.73200000 | 1.64470500 | -0.32414300 |
| H | 0.05029000 | 1.80354100 | 2.09205000 |
| H | 1.10417100 | 0.42324800 | 2.46824000 |
| H | -0.29550500 | 0.21199100 | 1.38126300 |
| C | 3.33479600 | -1.02016400 | 3.92747200 |
| N | 3.59204200 | 0.31562400 | 4.14217200 |
| C | 3.60177500 | -1.25327200 | 2.61260200 |
| C | 4.00324700 | 0.88389300 | 3.00926900 |
| N | 4.01433200 | -0.05784400 | 2.07600800 |
| H | 3.54115600 | -2.15287900 | 2.02165200 |
| H | 4.28362000 | 1.91556300 | 2.86631200 |
| H | 4.24096600 | 0.10129500 | 1.08875500 |
| H | 2.99422400 | -1.67251700 | 4.71430800 |
| H | 7.85918700 | 1.10912000 | -1.90532100 |
| H | 3.49437500 | 0.80193000 | 5.02627400 |

| | | | |
|---|---|---|---|
| O | 2.35210900 | -1.09282300 | -2.25203000 |
| C | 0.94399400 | -3.01253700 | -2.40794000 |
| H | 1.19239400 | -3.17856300 | -3.46007000 |
| H | 0.67897500 | -3.97202500 | -1.95302300 |
| H | 0.09663300 | -2.32415100 | -2.33599400 |
| C | -5.02710300 | 3.80798400 | 0.47162600 |
| C | -5.14140800 | 2.45414800 | 0.15334700 |
| C | -4.08464600 | 1.73592000 | -0.38690200 |
| C | -2.83616400 | 2.35780000 | -0.61892500 |
| C | -2.74367500 | 3.72187100 | -0.31464400 |
| C | -3.80936200 | 4.44331300 | 0.22504400 |
| H | -5.87569800 | 4.33692000 | 0.88786000 |
| H | -6.08531700 | 1.93788700 | 0.30617700 |
| C | -1.66496300 | 1.69757800 | -1.19099000 |
| H | -1.80816600 | 4.24675800 | -0.48981400 |
| H | -0.90181900 | 2.33225900 | -1.65574100 |
| O | -4.31825000 | 0.42430000 | -0.75662600 |
| N | -1.46747200 | 0.41334100 | -1.12406600 |
| O | -0.36291000 | -0.04586400 | -1.64504700 |
| C | -4.61502500 | -1.83929500 | -0.16599600 |
| C | -5.86563300 | -2.41708800 | -0.12389300 |
| N | -3.86933300 | -2.75112200 | -0.83352900 |
| H | -6.77805600 | -2.03038200 | 0.30622600 |
| N | -5.81245600 | -3.62197900 | -0.74077100 |
| N | -4.59492600 | -3.81900900 | -1.16340100 |
| C | -4.10831400 | -0.51391000 | 0.29578500 |
| H | -4.67185800 | -0.20797800 | 1.18535100 |
| H | -3.03963600 | -0.55837700 | 0.53596300 |
| C | -2.42989200 | -2.73470700 | -1.08025500 |
| H | -2.26148400 | -3.45343400 | -1.88723900 |
| H | -2.14011500 | -1.73432100 | -1.41615900 |
| C | -1.65218000 | -3.10541400 | 0.16477100 |
| C | -2.02584900 | -4.19682100 | 0.95283300 |
| C | -0.54115300 | -2.33915600 | 0.51958300 |

| | | | |
|---|---|---|---|
| C | -1.28763100 | -4.52536900 | 2.08696500 |
| H | -2.89825300 | -4.78610900 | 0.67898300 |
| C | 0.19387100 | -2.67233600 | 1.65766800 |
| H | -0.26180000 | -1.49031500 | -0.10967900 |
| C | -0.17382600 | -3.76301700 | 2.44194000 |
| H | -1.58150200 | -5.37570000 | 2.69492500 |
| H | 1.04967600 | -2.06103100 | 1.93237900 |
| H | 0.39915700 | -4.01759700 | 3.32873500 |
| O | -3.56425000 | 5.76049900 | 0.47879700 |
| C | -4.62378900 | 6.52170400 | 1.02600800 |
| H | -4.93793600 | 6.12467600 | 1.99822500 |
| H | -4.23685900 | 7.53228900 | 1.15492700 |
| H | -5.48697800 | 6.54426300 | 0.35100300 |

**TS1(EE)**

| | | | |
|---|---|---|---|
| N | 6.67667200 | 2.06981000 | -1.99717300 |
| C | 5.66738500 | 1.67654800 | -1.02839100 |
| C | 4.65102700 | 0.76027900 | -1.72165500 |
| O | 3.63163200 | 0.43052700 | -0.78947300 |
| C | 6.21574400 | 0.96798900 | 0.19800700 |
| O | 5.61671800 | 0.88202700 | 1.24855100 |
| O | 1.82309100 | 1.25568200 | -2.34530200 |
| P | 2.06750900 | 0.22155700 | -1.31572400 |
| C | 1.51443600 | -2.13512500 | -2.61349000 |
| O | 1.45051800 | 0.28566700 | 0.16097900 |
| C | 1.68909900 | 1.46242500 | 0.96148100 |
| C | 0.76697400 | 1.38431600 | 2.15646500 |
| H | 7.31227300 | 2.74959800 | -1.58649500 |
| H | 5.12459100 | 2.56286400 | -0.68271700 |
| H | 4.22869700 | 1.29878700 | -2.57324100 |
| H | 5.13334800 | -0.15838100 | -2.08032100 |
| H | 7.21271400 | 0.49750500 | 0.07443500 |
| H | 2.11255100 | -3.04796300 | -2.66122600 |
| H | 0.52927900 | -2.35516400 | -2.20630100 |

| | | | |
|---|---|---|---|
| H | 2.74329800 | 1.48298500 | 1.25824700 |
| H | 1.48436900 | 2.35428200 | 0.35845200 |
| H | 0.89337600 | 2.27058000 | 2.78441100 |
| H | 0.99899300 | 0.49819000 | 2.75498100 |
| H | -0.27435700 | 1.32841300 | 1.82487500 |
| C | 2.90929000 | -2.98787200 | 2.56336600 |
| N | 4.01889400 | -2.48526300 | 3.20659900 |
| C | 2.73186900 | -2.22366100 | 1.45152800 |
| C | 4.50733800 | -1.45274800 | 2.52249400 |
| N | 3.73591200 | -1.28244800 | 1.45543100 |
| H | 1.99203700 | -2.25592800 | 0.66688200 |
| H | 5.36839700 | -0.85513400 | 2.77576200 |
| H | 3.87339800 | -0.56163100 | 0.73140200 |
| H | 2.35239100 | -3.82532000 | 2.95042800 |
| H | 7.24465400 | 1.26618000 | -2.26236200 |
| H | 4.41038000 | -2.83828300 | 4.07209700 |
| O | 2.22239900 | -1.32441000 | -1.64910300 |
| C | 1.41498200 | -1.47496900 | -3.97192500 |
| H | 2.40480400 | -1.17586200 | -4.33088500 |
| H | 0.98891800 | -2.18710200 | -4.68499300 |
| H | 0.77113200 | -0.59706000 | -3.91144100 |
| C | -4.03454700 | 4.17914300 | 0.84635000 |
| C | -4.44680200 | 2.96143000 | 0.30287400 |
| C | -3.54920600 | 2.07314100 | -0.27108000 |
| C | -2.16816200 | 2.37499900 | -0.31166000 |
| C | -1.77142700 | 3.60335000 | 0.22869000 |
| C | -2.67683500 | 4.49652500 | 0.80460700 |
| H | -4.76563100 | 4.85016900 | 1.28035300 |
| H | -5.49905500 | 2.69099500 | 0.30604300 |
| C | -1.12803000 | 1.52840800 | -0.90241600 |
| H | -0.72064500 | 3.88296200 | 0.21162900 |
| H | -0.16468700 | 1.99808500 | -1.11184400 |
| O | -4.05604600 | 0.92103400 | -0.83979100 |
| N | -1.31434500 | 0.27330100 | -1.16047200 |

| | | | |
|---|---|---|---|
| O | -0.31361000 | -0.41481900 | -1.66286000 |
| C | -4.58499500 | -1.37782800 | -0.67143000 |
| C | -5.87132800 | -1.69056000 | -1.05652600 |
| N | -3.85551900 | -2.42444800 | -1.12939000 |
| H | -6.78097300 | -1.12895300 | -0.90043000 |
| N | -5.85988600 | -2.88052000 | -1.70244300 |
| N | -4.63464100 | -3.32266300 | -1.73442800 |
| C | -4.03924500 | -0.19109000 | 0.05277100 |
| H | -4.67687500 | 0.02945700 | 0.91856000 |
| H | -3.01661200 | -0.37938900 | 0.38994000 |
| C | -2.42637400 | -2.69953800 | -0.97374000 |
| H | -2.26000100 | -3.62146700 | -1.53817800 |
| H | -1.84107800 | -1.89118300 | -1.42107300 |
| C | -2.05401500 | -2.87780500 | 0.48166000 |
| C | -2.82284900 | -3.69724100 | 1.31173800 |
| C | -0.94276100 | -2.21014900 | 1.00085500 |
| C | -2.48511700 | -3.85484900 | 2.65247000 |
| H | -3.69311100 | -4.20684200 | 0.90265600 |
| C | -0.61236700 | -2.36623300 | 2.34765000 |
| H | -0.36297100 | -1.56048400 | 0.34372300 |
| C | -1.37751700 | -3.18662600 | 3.17392300 |
| H | -3.08779300 | -4.49357400 | 3.29100400 |
| H | 0.24167700 | -1.83499300 | 2.75734700 |
| H | -1.11713600 | -3.29996100 | 4.22199700 |
| O | -2.14002900 | 5.64908000 | 1.29528000 |
| C | -3.02606100 | 6.56438300 | 1.91139500 |
| H | -3.53371200 | 6.10857200 | 2.76910200 |
| H | -2.41319800 | 7.39830200 | 2.25264300 |
| H | -3.77609800 | 6.93085400 | 1.20120700 |

**IN(EE)**

| | | | |
|---|---|---|---|
| N | 6.32991700 | 2.25785200 | -2.38381000 |
| C | 5.43373600 | 2.00449300 | -1.26379000 |
| C | 4.24352000 | 1.16750600 | -1.75329300 |

| | | | |
|---|---|---|---|
| O | 3.34479100 | 0.97927200 | -0.69055300 |
| C | 6.07646800 | 1.25569700 | -0.11852700 |
| O | 5.77805700 | 1.40456300 | 1.04757000 |
| O | 1.43809700 | 1.68500100 | -2.08674900 |
| P | 1.66802400 | 0.56713700 | -1.10942200 |
| C | 1.63713400 | -2.03077400 | -2.09170900 |
| O | 1.34106300 | 0.68659100 | 0.49575500 |
| C | 1.77734700 | 1.80845100 | 1.27103000 |
| C | 0.83685000 | 1.92236000 | 2.45356100 |
| H | 7.12873600 | 2.80890100 | -2.07777100 |
| H | 5.04606300 | 2.95123000 | -0.87459400 |
| H | 3.76328300 | 1.70924400 | -2.57340000 |
| H | 4.60140100 | 0.19942300 | -2.13920200 |
| H | 6.83647800 | 0.50154700 | -0.41603500 |
| H | 2.39919200 | -2.81576900 | -2.06160300 |
| H | 0.79464900 | -2.34395800 | -1.47020000 |
| H | 2.81334000 | 1.65878400 | 1.59251600 |
| H | 1.75037100 | 2.71554500 | 0.65485500 |
| H | 1.13406900 | 2.75622000 | 3.09573100 |
| H | 0.85920000 | 1.00169400 | 3.04461800 |
| H | -0.18992700 | 2.09046900 | 2.11269800 |
| C | 3.22427500 | -2.71383000 | 2.38829900 |
| N | 4.42653800 | -2.19623900 | 2.81703000 |
| C | 2.81595300 | -1.92335600 | 1.35851200 |
| C | 4.74169400 | -1.12917900 | 2.08252700 |
| N | 3.77571100 | -0.95166700 | 1.19031200 |
| H | 1.93347600 | -1.95689800 | 0.74087100 |
| H | 5.61708800 | -0.50775300 | 2.19408600 |
| H | 3.72317300 | -0.18372100 | 0.47203400 |
| H | 2.77409500 | -3.57974800 | 2.84536800 |
| H | 6.70637000 | 1.37807200 | -2.73560600 |
| H | 4.99179400 | -2.55897300 | 3.57545800 |
| O | 2.28646200 | -0.89914600 | -1.49922600 |
| C | 1.20434800 | -1.76859200 | -3.52074300 |

| | | | |
|---|---|---|---|
| H | 2.05799500 | -1.43929900 | -4.12090300 |
| H | 0.80559000 | -2.68704000 | -3.96254500 |
| H | 0.42986100 | -0.99963900 | -3.54844700 |
| C | -4.62953600 | 3.81899500 | 0.65449200 |
| C | -4.77427400 | 2.54226900 | 0.11209300 |
| C | -3.68028500 | 1.79858500 | -0.30841300 |
| C | -2.38028200 | 2.33113300 | -0.18646600 |
| C | -2.24587000 | 3.61442700 | 0.34661700 |
| C | -3.34863100 | 4.35845300 | 0.77283100 |
| H | -5.50829300 | 4.36960300 | 0.96770800 |
| H | -5.76252000 | 2.10678700 | -0.00311700 |
| C | -1.13476800 | 1.66061000 | -0.60097200 |
| H | -1.25870900 | 4.05939700 | 0.44338500 |
| H | -0.24816600 | 2.29044700 | -0.64762400 |
| O | -3.91595200 | 0.56647200 | -0.88045000 |
| N | -1.11769600 | 0.41483500 | -0.89138600 |
| O | 0.04571200 | -0.19130300 | -1.24544900 |
| C | -4.32203800 | -1.75375900 | -0.62901500 |
| C | -5.56370300 | -2.25647200 | -0.95292500 |
| N | -3.46729500 | -2.67226400 | -1.14091400 |
| H | -6.53900300 | -1.84041000 | -0.74610900 |
| N | -5.40339600 | -3.42545500 | -1.61774400 |
| N | -4.12805400 | -3.67270600 | -1.72208700 |
| C | -3.91076400 | -0.50037800 | 0.06845700 |
| H | -4.63014100 | -0.28323000 | 0.86685000 |
| H | -2.91190800 | -0.60379000 | 0.50605800 |
| C | -2.00948100 | -2.72232400 | -1.03715200 |
| H | -1.68971600 | -3.43326400 | -1.80362400 |
| H | -1.61296600 | -1.73418500 | -1.27582400 |
| C | -1.56857400 | -3.16643200 | 0.34176500 |
| C | -1.99117200 | -4.40109000 | 0.84203800 |
| C | -0.74809700 | -2.35019800 | 1.12298500 |
| C | -1.59382600 | -4.82019000 | 2.10730300 |
| H | -2.63606700 | -5.03080600 | 0.23313700 |

| | | | |
|---|---|---|---|
| C | -0.35411700 | -2.77130700 | 2.39492400 |
| H | -0.41806300 | -1.38817700 | 0.73315500 |
| C | -0.77241900 | -4.00434200 | 2.88729100 |
| H | -1.92457600 | -5.78198200 | 2.48695000 |
| H | 0.27913800 | -2.12895200 | 3.00078800 |
| H | -0.46431200 | -4.32926600 | 3.87635500 |
| O | -3.07316700 | 5.58852400 | 1.28021400 |
| C | -4.16809000 | 6.35806500 | 1.74474300 |
| H | -4.69774700 | 5.84254900 | 2.55350800 |
| H | -3.74602900 | 7.28902800 | 2.12137400 |
| H | -4.87055300 | 6.57765000 | 0.93296400 |

**TS2(EE)**

| | | | |
|---|---|---|---|
| N | -6.33550200 | 2.09081800 | 2.30291400 |
| C | -5.38789600 | 1.95487400 | 1.20604600 |
| C | -4.33633600 | 0.90222100 | 1.58191900 |
| O | -3.36853100 | 0.81067700 | 0.55826200 |
| C | -6.01464100 | 1.52774900 | -0.10491100 |
| O | -5.59019800 | 1.83824900 | -1.19629500 |
| O | -1.52749300 | 1.69292600 | 1.97974600 |
| P | -1.57169200 | 0.47147100 | 1.11772400 |
| C | -1.64337300 | -1.89658000 | 2.48500300 |
| O | -1.24164500 | 0.50310900 | -0.47290500 |
| C | -1.63868700 | 1.60066300 | -1.31191000 |
| C | -0.62148800 | 1.70115200 | -2.42941000 |
| H | -7.02704100 | 2.80399300 | 2.08249600 |
| H | -4.86871100 | 2.90444900 | 1.04175600 |
| H | -3.85729000 | 1.21418300 | 2.51331700 |
| H | -4.81383700 | -0.07614300 | 1.74327100 |
| H | -6.89070400 | 0.85014700 | -0.00720800 |
| H | -2.37010000 | -2.71289600 | 2.50491400 |
| H | -0.70286300 | -2.27420300 | 2.08033200 |
| H | -2.64440200 | 1.41401800 | -1.69949900 |
| H | -1.67277400 | 2.52057100 | -0.71727200 |

| | | | |
|---|---|---|---|
| H | -0.89795600 | 2.50706600 | -3.11491300 |
| H | -0.58163300 | 0.76368400 | -2.99159900 |
| H | 0.37598900 | 1.90887800 | -2.02969000 |
| C | -3.67003800 | -2.84517000 | -2.23384300 |
| N | -4.64875700 | -2.07106200 | -2.81114700 |
| C | -3.21463400 | -2.13032300 | -1.16487900 |
| C | -4.77098700 | -0.93366500 | -2.11149500 |
| N | -3.91356600 | -0.94615300 | -1.10357200 |
| H | -2.44509200 | -2.36377800 | -0.44516600 |
| H | -5.45589900 | -0.13289700 | -2.34809800 |
| H | -3.67624600 | -0.03671600 | -0.26446000 |
| H | -3.39228900 | -3.80891300 | -2.62863300 |
| H | -6.85165800 | 1.22068600 | 2.42896600 |
| H | -5.19220400 | -2.31154600 | -3.63034300 |
| O | -2.21867700 | -0.94908200 | 1.56825700 |
| C | -1.46330900 | -1.30504800 | 3.86907500 |
| H | -2.41302300 | -0.90306200 | 4.23446300 |
| H | -1.12388200 | -2.07953800 | 4.56295600 |
| H | -0.72288900 | -0.50196400 | 3.85112500 |
| C | 4.62215100 | 3.80816000 | -0.67912900 |
| C | 4.79344600 | 2.52831800 | -0.15250100 |
| C | 3.71870400 | 1.77627600 | 0.30090300 |
| C | 2.41435800 | 2.30550000 | 0.23010400 |
| C | 2.25210100 | 3.59074800 | -0.28963500 |
| C | 3.33575300 | 4.34309500 | -0.74909600 |
| H | 5.48679200 | 4.36521100 | -1.01920200 |
| H | 5.78720000 | 2.09695600 | -0.07695200 |
| C | 1.18788100 | 1.62649700 | 0.68452600 |
| H | 1.25979100 | 4.03055800 | -0.34884400 |
| H | 0.29270400 | 2.24565800 | 0.73451900 |
| O | 3.97606900 | 0.53980200 | 0.85082300 |
| N | 1.18815700 | 0.38979400 | 1.00782100 |
| O | 0.01646400 | -0.19904800 | 1.40509300 |
| C | 4.37100500 | -1.77626400 | 0.56228300 |

| | | | |
|---|---|---|---|
| C | 5.62486200 | -2.27013800 | 0.85003100 |
| N | 3.53753200 | -2.71005300 | 1.08086400 |
| H | 6.59100400 | -1.84136100 | 0.62702300 |
| N | 5.49155400 | -3.45052900 | 1.50085700 |
| N | 4.22177800 | -3.71296500 | 1.63067800 |
| C | 3.92761500 | -0.51893200 | -0.10684100 |
| H | 4.60858700 | -0.29259100 | -0.93565900 |
| H | 2.90952400 | -0.62104200 | -0.49927800 |
| C | 2.08091200 | -2.77626900 | 1.00085900 |
| H | 1.78736400 | -3.53869900 | 1.72765500 |
| H | 1.67322200 | -1.81223900 | 1.31053500 |
| C | 1.61574700 | -3.13717800 | -0.39436500 |
| C | 2.24045000 | -4.17265900 | -1.09419200 |
| C | 0.55758900 | -2.44381200 | -0.98439300 |
| C | 1.80773700 | -4.51804900 | -2.37039200 |
| H | 3.07030200 | -4.70480900 | -0.63423300 |
| C | 0.12516600 | -2.79340500 | -2.26443900 |
| H | 0.07948700 | -1.62784300 | -0.44583600 |
| C | 0.74707400 | -3.82864000 | -2.95841400 |
| H | 2.29851400 | -5.32426600 | -2.90695300 |
| H | -0.69729300 | -2.24857000 | -2.71930300 |
| H | 0.41134800 | -4.09592100 | -3.95571300 |
| O | 3.03757100 | 5.57451200 | -1.23797900 |
| C | 4.11374600 | 6.35705900 | -1.72507200 |
| H | 4.62029400 | 5.85652500 | -2.55763600 |
| H | 3.67491700 | 7.29089900 | -2.07438800 |
| H | 4.83985200 | 6.56826300 | -0.93222400 |

**C2(EE)**

| | | | |
|---|---|---|---|
| N | 4.08044600 | 4.79508300 | 0.69758200 |
| C | 3.48022000 | 3.52856400 | 1.11852000 |
| C | 4.19490700 | 2.37508000 | 0.42821900 |
| O | 3.72587100 | 1.17166100 | 0.99063400 |
| C | 3.57896500 | 3.33870700 | 2.61840700 |

| | | | |
|---|---|---|---|
| O | 2.63603600 | 3.25392500 | 3.37206200 |
| O | 1.28809700 | 2.07514100 | -0.71661600 |
| P | 1.28498500 | 0.60131200 | -0.67789700 |
| C | 2.71841900 | -1.52661100 | -1.20474000 |
| O | 1.11683700 | -0.08657900 | 0.73992800 |
| C | 0.98133000 | 0.68530800 | 1.95714600 |
| C | -0.45704900 | 0.61873900 | 2.42352700 |
| H | 3.56904700 | 5.57450000 | 1.10610600 |
| H | 2.42469200 | 3.51503800 | 0.83334600 |
| H | 3.99637100 | 2.43498400 | -0.65077200 |
| H | 5.28244600 | 2.48235700 | 0.58380400 |
| H | 4.62005400 | 3.32880300 | 3.01175100 |
| H | 3.24875400 | -1.64633100 | -0.25632900 |
| H | 1.75035200 | -2.03342700 | -1.13704500 |
| H | 1.66877800 | 0.23079300 | 2.67271000 |
| H | 1.31368000 | 1.70770700 | 1.77495000 |
| H | -0.56514300 | 1.15271600 | 3.37208300 |
| H | -0.76439900 | -0.42143200 | 2.57275600 |
| H | -1.12406900 | 1.07691200 | 1.68561000 |
| C | 6.96921500 | -2.07723100 | -1.04367200 |
| N | 7.07516300 | -0.93398600 | -1.79523800 |
| C | 6.08255000 | -1.77256500 | -0.04699500 |
| C | 6.26890400 | 0.00162600 | -1.24414100 |
| N | 5.65163000 | -0.47308800 | -0.18131800 |
| H | 5.72910900 | -2.40698200 | 0.75295100 |
| H | 6.16463900 | 0.99726900 | -1.65119100 |
| H | 4.31596400 | 0.46146900 | 0.63935400 |
| H | 7.51963000 | -2.97309000 | -1.28194400 |
| H | 5.03542000 | 4.86217100 | 1.04995900 |
| H | 7.65371400 | -0.80926200 | -2.61527200 |
| O | 2.49925500 | -0.10574400 | -1.40372800 |
| C | 3.52749900 | -2.03965300 | -2.37249300 |
| H | 3.76081000 | -3.09661000 | -2.21833100 |
| H | 2.96453000 | -1.93636100 | -3.30384500 |

| | | | |
|---|---|---|---|
| H | 4.46576900 | -1.48579400 | -2.46206600 |
| C | -5.36970700 | 3.13477400 | -0.33445400 |
| C | -5.23926500 | 1.74859500 | -0.27825500 |
| C | -4.05109500 | 1.12040800 | -0.62645400 |
| C | -2.95735800 | 1.89527700 | -1.04990300 |
| C | -3.09995400 | 3.28030000 | -1.13547200 |
| C | -4.29164400 | 3.90900000 | -0.76907300 |
| H | -6.31237900 | 3.58916600 | -0.05417200 |
| H | -6.07999000 | 1.13371200 | 0.02840600 |
| C | -1.65145800 | 1.34190300 | -1.43418100 |
| H | -2.27360300 | 3.89594400 | -1.47849200 |
| H | -1.01853600 | 1.95003100 | -2.08184000 |
| O | -3.99629500 | -0.25229600 | -0.62957200 |
| N | -1.22766900 | 0.21945500 | -0.99593300 |
| O | 0.06360600 | -0.06602200 | -1.53915000 |
| C | -4.32680300 | -2.24053100 | 0.59293500 |
| C | -5.44260600 | -2.83421900 | 1.13944900 |
| N | -3.78052400 | -3.22346100 | -0.15974100 |
| H | -6.18333300 | -2.41074100 | 1.80160700 |
| N | -5.50624800 | -4.11824700 | 0.70817200 |
| N | -4.49356000 | -4.34518400 | -0.07900200 |
| C | -3.78633300 | -0.85339700 | 0.65240300 |
| H | -4.32076700 | -0.29588500 | 1.42825900 |
| H | -2.71320300 | -0.84266800 | 0.88051200 |
| C | -2.51182500 | -3.20384500 | -0.88907200 |
| H | -2.65261800 | -3.83679700 | -1.76790600 |
| H | -2.34149100 | -2.17381000 | -1.21488900 |
| C | -1.37920200 | -3.69964000 | -0.01905500 |
| C | -1.26352900 | -5.05923300 | 0.28053100 |
| C | -0.46146400 | -2.79596200 | 0.51479900 |
| C | -0.23786300 | -5.50776900 | 1.10712500 |
| H | -1.98285500 | -5.76210300 | -0.13197900 |
| C | 0.56876800 | -3.24521200 | 1.34086900 |
| H | -0.54459900 | -1.73861400 | 0.27973800 |

| | | | |
|---|---|---|---|
| C | 0.68061800 | -4.60081000 | 1.63904500 |
| H | -0.15104600 | -6.56580900 | 1.33467200 |
| H | 1.28097400 | -2.52815900 | 1.74009200 |
| H | 1.48151100 | -4.95388400 | 2.28151700 |
| O | -4.30750500 | 5.26033500 | -0.87749900 |
| C | -5.50506600 | 5.92925700 | -0.51774100 |
| H | -5.75678500 | 5.74788400 | 0.53285300 |
| H | -5.31363700 | 6.99068600 | -0.66904300 |
| H | -6.33950300 | 5.61338200 | -1.15325000 |

**EZ**

| | | | |
|---|---|---|---|
| C | 2.52595000 | 1.87012100 | -0.32473300 |
| C | 1.37106200 | 1.07101700 | -0.34335700 |
| C | 1.45281500 | -0.29819700 | -0.16051500 |
| C | 2.70570100 | -0.94506900 | 0.04674400 |
| C | 3.84143000 | -0.12625200 | 0.05983700 |
| C | 3.75561100 | 1.25988500 | -0.12241500 |
| H | 2.42919000 | 2.93936400 | -0.46816500 |
| H | 0.41493700 | 1.55765700 | -0.50340500 |
| C | 2.73336900 | -2.39161600 | 0.22615300 |
| H | 4.80364600 | -0.59210900 | 0.21557900 |
| O | 0.35049300 | -1.12441000 | -0.16740300 |
| C | -1.93324100 | -1.57978700 | -0.19469600 |
| C | -1.88212300 | -2.85900800 | 0.30962800 |
| N | -3.22629200 | -1.42871900 | -0.56150500 |
| H | -1.03817100 | -3.39540400 | 0.71575200 |
| N | -3.12347500 | -3.40386600 | 0.22593500 |
| N | -3.93317500 | -2.53453600 | -0.29762200 |
| C | -0.90171600 | -0.50773800 | -0.32702300 |
| H | -0.97845400 | -0.01402500 | -1.30784200 |
| H | -1.05859400 | 0.26514500 | 0.44016400 |
| N | 3.76001600 | -3.18467000 | 0.45376200 |
| O | 4.95121300 | -2.68447600 | 0.55178200 |
| H | 1.78211000 | -2.90989000 | 0.16320400 |

| | | | |
|---|---|---|---|
| C | -3.86545500 | -0.26787600 | -1.17483500 |
| H | -3.48581400 | -0.15266100 | -2.19445500 |
| H | -4.92742000 | -0.51913300 | -1.23053700 |
| C | -3.62063900 | 0.98584200 | -0.36702500 |
| C | -3.05659500 | 2.11210100 | -0.96444200 |
| C | -3.92538200 | 1.00829500 | 0.99654900 |
| C | -2.79810400 | 3.25420600 | -0.20708600 |
| H | -2.80982400 | 2.09403700 | -2.02324900 |
| C | -3.66459800 | 2.14573900 | 1.75346800 |
| H | -4.35707300 | 0.12589800 | 1.46363400 |
| C | -3.09839000 | 3.27070600 | 1.15234500 |
| H | -2.35504700 | 4.12547800 | -0.67908300 |
| H | -3.90029500 | 2.15508000 | 2.81298700 |
| H | -2.88905300 | 4.15604700 | 1.74440700 |
| O | 4.95062300 | 1.92794300 | -0.08458800 |
| C | 4.90622900 | 3.32898000 | -0.25692000 |
| H | 4.31645900 | 3.80997400 | 0.53283800 |
| H | 5.93740300 | 3.67855600 | -0.20214800 |
| H | 4.48231200 | 3.59797800 | -1.23203600 |

**C1(EZ)**

| | | | |
|---|---|---|---|
| N | -8.36893500 | 1.33279100 | -2.11141000 |
| C | -7.13477900 | 0.64820300 | -1.72851700 |
| C | -7.38323700 | -0.84151400 | -1.58804000 |
| O | -7.43371100 | -1.42764400 | -0.52700200 |
| C | -6.63250900 | 1.27400800 | -0.43183600 |
| O | -5.54374200 | 0.55016700 | 0.15626600 |
| C | -2.93179900 | 2.54813800 | -0.09598300 |
| C | -2.24581400 | 3.64431400 | -0.87686600 |
| C | -2.46592300 | -1.43402600 | 0.36246400 |
| C | -1.28504200 | -1.22381200 | 1.27911700 |
| O | -3.69714700 | 1.76076500 | -1.04026400 |
| O | -3.34664300 | -0.29060700 | 0.52354400 |
| O | -4.37237900 | -0.56616500 | -1.87470600 |

| | | | |
|---|---|---|---|
| P | -4.17618700 | 0.29445400 | -0.68952100 |
| H | -6.33241300 | 2.30965700 | -0.61391100 |
| H | -7.42655400 | 1.26076600 | 0.31876300 |
| H | -8.62242400 | 1.10998600 | -3.07077400 |
| H | -9.14199500 | 1.03328000 | -1.51947300 |
| H | -6.40605300 | 0.79221700 | -2.53166500 |
| H | -7.61482600 | -1.36942600 | -2.53218900 |
| H | -3.62288600 | 2.94327600 | 0.65663000 |
| H | -2.19990400 | 1.88913000 | 0.37395800 |
| H | -1.61447700 | 3.18295000 | -1.64070400 |
| H | -2.97376100 | 4.30692800 | -1.35306200 |
| H | -1.61057500 | 4.23114000 | -0.20693800 |
| H | -0.75860100 | -0.31589700 | 0.96985200 |
| H | -1.61018100 | -1.13139500 | 2.32026200 |
| H | -0.60161400 | -2.07445400 | 1.20283300 |
| H | -2.14627000 | -1.50356100 | -0.67625600 |
| H | -3.04969500 | -2.31940300 | 0.63594700 |
| C | -4.87985500 | -1.76878500 | 2.74641600 |
| N | -5.77970100 | -1.80337100 | 1.70640200 |
| C | -4.89215000 | -3.00527900 | 3.31449300 |
| C | -6.32981700 | -3.00777000 | 1.62763800 |
| N | -5.80173200 | -3.75165900 | 2.59749700 |
| H | -4.34635200 | -3.41514500 | 4.14786400 |
| H | -7.06448100 | -3.31620500 | 0.90088100 |
| H | -6.03967500 | -4.72110600 | 2.77256000 |
| H | -4.31116600 | -0.88261200 | 2.97590400 |
| H | -5.99285700 | -1.02703200 | 1.06738900 |
| C | 2.81895000 | 2.70627900 | 1.23538800 |
| C | 3.51349500 | 1.57126800 | 0.78628900 |
| C | 2.89281500 | 0.63118300 | -0.01833600 |
| C | 1.53369400 | 0.78291300 | -0.41534900 |
| C | 0.86506000 | 1.92203700 | 0.04468400 |
| C | 1.49423800 | 2.87196900 | 0.85891800 |
| H | 3.33340500 | 3.42273500 | 1.86422300 |

| | | | |
|---|---|---|---|
| H | 4.54849300 | 1.45293500 | 1.08807100 |
| C | 0.92226200 | -0.23679800 | -1.26384400 |
| H | -0.16409300 | 2.06203800 | -0.25043200 |
| O | 3.52564200 | -0.49945900 | -0.48186000 |
| C | 5.34482100 | -1.92021100 | -0.78282800 |
| C | 4.84148700 | -2.66161000 | -1.82655800 |
| N | 6.48327400 | -2.56751600 | -0.44543900 |
| H | 3.93923000 | -2.50462700 | -2.39767900 |
| N | 5.68729100 | -3.69734900 | -2.06511000 |
| N | 6.68011800 | -3.63415000 | -1.23063700 |
| C | 4.87164200 | -0.67113800 | -0.11478000 |
| H | 4.97516100 | -0.75185600 | 0.97793300 |
| H | 5.48009800 | 0.18611500 | -0.43928800 |
| N | -0.31595200 | -0.33120000 | -1.68605300 |
| O | -1.18069100 | 0.58548700 | -1.34687200 |
| H | 1.56342900 | -1.04994100 | -1.58883600 |
| C | 7.43766700 | -2.23343500 | 0.60789000 |
| H | 6.96052600 | -2.38748100 | 1.58032100 |
| H | 8.24658300 | -2.96056700 | 0.50427400 |
| C | 7.93216000 | -0.81172000 | 0.47011500 |
| C | 7.80856900 | 0.08642800 | 1.52905300 |
| C | 8.48671400 | -0.38141300 | -0.73829600 |
| C | 8.23689700 | 1.40582700 | 1.38481000 |
| H | 7.37023000 | -0.24418800 | 2.46763600 |
| C | 8.91146300 | 0.93495800 | -0.88396400 |
| H | 8.57415000 | -1.08102000 | -1.56655900 |
| C | 8.78597200 | 1.83124500 | 0.17825300 |
| H | 8.13510200 | 2.10006000 | 2.21294100 |
| H | 9.33861200 | 1.26428700 | -1.82598300 |
| H | 9.11451600 | 2.85930800 | 0.06264400 |
| O | 0.71074900 | 3.93135500 | 1.23100900 |
| C | 1.31124600 | 4.92010200 | 2.04244600 |
| H | 2.16430800 | 5.38751600 | 1.53615100 |
| H | 0.54418400 | 5.67201000 | 2.22759500 |

| | | | |
|---|---|---|---|
| H | 1.64908400 | 4.50059900 | 2.99761100 |

**TS1(EZ)**

| | | | |
|---|---|---|---|
| N | -7.65937800 | 2.81047600 | -1.16069400 |
| C | -6.76859500 | 1.66110300 | -1.12651500 |
| C | -5.92380700 | 1.74544400 | 0.15906000 |
| O | -5.06362100 | 0.62992100 | 0.31254800 |
| C | -7.47310000 | 0.31982600 | -1.17597600 |
| O | -6.91463600 | -0.72692200 | -1.42990800 |
| O | -3.79992700 | 1.21996700 | -1.80173700 |
| P | -3.56179700 | 0.67194200 | -0.44679600 |
| C | -2.06392700 | 1.07455200 | 1.69380400 |
| O | -3.31497000 | -0.87592100 | -0.13199600 |
| C | -3.26055800 | -1.77395000 | -1.26418000 |
| C | -2.78792300 | -3.11671100 | -0.75934700 |
| H | -8.13444700 | 2.86102100 | -2.05853900 |
| H | -6.07286200 | 1.70152100 | -1.96926600 |
| H | -5.35903500 | 2.68326300 | 0.13308300 |
| H | -6.57953300 | 1.76754200 | 1.03629300 |
| H | -8.55824200 | 0.33584700 | -0.94235200 |
| H | -2.69923200 | 0.39426900 | 2.27306200 |
| H | -1.22908800 | 0.51252100 | 1.26845200 |
| H | -4.26731500 | -1.83215700 | -1.69713200 |
| H | -2.56723300 | -1.35615000 | -1.99465800 |
| H | -2.72302900 | -3.82262600 | -1.59215900 |
| H | -3.47470100 | -3.52725200 | -0.01195000 |
| H | -1.79734400 | -2.99768500 | -0.31521600 |
| C | -5.49807800 | -3.80782800 | 2.13077900 |
| N | -6.31134200 | -4.08470000 | 1.05420500 |
| C | -5.13464100 | -2.50205800 | 2.00659700 |
| C | -6.44801200 | -2.99384500 | 0.30081300 |
| N | -5.74068900 | -2.02630000 | 0.86839000 |
| H | -4.50018500 | -1.88015600 | 2.61622600 |
| H | -7.02786300 | -2.90076700 | -0.60379900 |

| | | | |
|---|---|---|---|
| H | -5.61739900 | -1.06838000 | 0.49857700 |
| H | -5.25614300 | -4.55146600 | 2.87170900 |
| H | -8.38729300 | 2.71680100 | -0.45332600 |
| H | -6.74961100 | -4.97716400 | 0.85773000 |
| O | -2.83481400 | 1.62257000 | 0.60969800 |
| C | -1.58990900 | 2.23407300 | 2.54276700 |
| H | -2.44058700 | 2.78296400 | 2.95636400 |
| H | -0.98114200 | 1.86063200 | 3.37108400 |
| H | -0.98211400 | 2.92045500 | 1.94566600 |
| C | 2.85106300 | 3.12482500 | 0.54622600 |
| C | 3.48804500 | 1.88114900 | 0.42421000 |
| C | 2.77144900 | 0.75261500 | 0.06035500 |
| C | 1.37801700 | 0.82429400 | -0.20935100 |
| C | 0.76827000 | 2.07638400 | -0.09755400 |
| C | 1.48946300 | 3.21186300 | 0.29126300 |
| H | 3.43719000 | 3.98829100 | 0.83686900 |
| H | 4.55273200 | 1.82813300 | 0.62322500 |
| C | 0.65755200 | -0.41526000 | -0.52153100 |
| H | -0.28961600 | 2.16021700 | -0.30849700 |
| O | 3.33823800 | -0.49318900 | -0.06146900 |
| C | 5.08827800 | -2.02431600 | -0.04946600 |
| C | 4.43541000 | -3.06178200 | -0.67405700 |
| N | 6.26290800 | -2.56870900 | 0.34065600 |
| H | 3.45922900 | -3.07154200 | -1.13432400 |
| N | 5.23645500 | -4.15835900 | -0.63460800 |
| N | 6.34081300 | -3.85427900 | -0.02381800 |
| C | 4.71922200 | -0.59649400 | 0.18460200 |
| H | 4.96034100 | -0.29728000 | 1.21615900 |
| H | 5.28767100 | 0.06054600 | -0.49036600 |
| N | -0.58213500 | -0.57981500 | -0.88633100 |
| O | -1.31072300 | 0.49384600 | -1.11301400 |
| H | 1.20878100 | -1.34179100 | -0.39366400 |
| C | 7.36335600 | -1.92682300 | 1.05267700 |
| H | 7.02881200 | -1.66780000 | 2.06181800 |

| | | | |
|---|---|---|---|
| H | 8.14012100 | -2.69091500 | 1.13515200 |
| C | 7.84952600 | -0.69959200 | 0.31566100 |
| C | 7.92751400 | 0.53180400 | 0.96414200 |
| C | 8.18958600 | -0.79000400 | -1.03685200 |
| C | 8.34607000 | 1.66636600 | 0.26908600 |
| H | 7.65348000 | 0.60641700 | 2.01367800 |
| C | 8.60303700 | 0.34156900 | -1.73148100 |
| H | 8.11740200 | -1.74942700 | -1.54456000 |
| C | 8.68121800 | 1.57260800 | -1.07893400 |
| H | 8.40189600 | 2.62224800 | 0.78039500 |
| H | 8.86250500 | 0.26561400 | -2.78276900 |
| H | 8.99925700 | 2.45616800 | -1.62339500 |
| O | 0.75338000 | 4.36050300 | 0.39588500 |
| C | 1.43601100 | 5.52456600 | 0.81682500 |
| H | 2.22375700 | 5.80284200 | 0.10666400 |
| H | 0.69015700 | 6.31827200 | 0.85982500 |
| H | 1.88057500 | 5.38718600 | 1.80948500 |

**IN(EZ)**

| | | | |
|---|---|---|---|
| N | -8.20563300 | 0.67138900 | -1.80994300 |
| C | -7.17857900 | -0.24056000 | -1.32355500 |
| C | -5.85693700 | 0.52515000 | -1.16434900 |
| O | -4.86125000 | -0.36182200 | -0.73392100 |
| C | -7.50034800 | -0.86680100 | 0.01356900 |
| O | -7.17263400 | -1.98650300 | 0.34469700 |
| O | -3.27322700 | 0.41289000 | -2.47200600 |
| P | -3.15171900 | 0.13466500 | -1.00247000 |
| C | -2.73878600 | 2.43763100 | 0.43888400 |
| O | -2.76222100 | -1.33226300 | -0.38325400 |
| C | -3.39563000 | -2.53157100 | -0.83782900 |
| C | -2.42748100 | -3.67067300 | -0.58845100 |
| H | -9.08916600 | 0.17898000 | -1.92133100 |
| H | -7.02181300 | -1.04602000 | -2.04776500 |
| H | -5.59810400 | 0.95857800 | -2.13617400 |

| | | | |
|---|---|---|---|
| H | -5.99067300 | 1.34961400 | -0.44434800 |
| H | -8.03824100 | -0.20184300 | 0.72409200 |
| H | -3.24531800 | 2.88131300 | 1.30149700 |
| H | -1.71457400 | 2.19314700 | 0.72825000 |
| H | -4.33744900 | -2.68463800 | -0.30005200 |
| H | -3.63432400 | -2.44239100 | -1.90333200 |
| H | -2.86408900 | -4.61858800 | -0.91570200 |
| H | -2.19576700 | -3.74549000 | 0.47819200 |
| H | -1.49488500 | -3.50481600 | -1.13458200 |
| C | -3.61051400 | -1.15967100 | 3.83442500 |
| N | -4.84886300 | -1.76017700 | 3.89417400 |
| C | -3.48204700 | -0.67731700 | 2.56865000 |
| C | -5.45504300 | -1.65187600 | 2.71155500 |
| N | -4.64090100 | -0.99436400 | 1.89633100 |
| H | -2.67989800 | -0.14969300 | 2.07968000 |
| H | -6.42908100 | -2.03792900 | 2.45135300 |
| H | -4.82670500 | -0.75805600 | 0.88204300 |
| H | -2.94723700 | -1.13234600 | 4.68262600 |
| H | -8.37224900 | 1.40561900 | -1.12245200 |
| H | -5.24607100 | -2.22029900 | 4.70446800 |
| O | -3.47761600 | 1.24169100 | 0.16982000 |
| C | -2.76791900 | 3.39529100 | -0.73851100 |
| H | -3.79839600 | 3.67680300 | -0.97653200 |
| H | -2.20078100 | 4.30045400 | -0.49999800 |
| H | -2.32748000 | 2.92605000 | -1.62298700 |
| C | 2.65535400 | 3.16137400 | 0.68628400 |
| C | 3.25118100 | 1.90797300 | 0.50460600 |
| C | 2.57789100 | 0.90190700 | -0.17658300 |
| C | 1.28413700 | 1.13329000 | -0.68982200 |
| C | 0.71188400 | 2.38746600 | -0.51105400 |
| C | 1.38421000 | 3.40273800 | 0.17567700 |
| H | 3.20280800 | 3.92573800 | 1.22461300 |
| H | 4.24374600 | 1.74461900 | 0.90927200 |
| C | 0.62348400 | 0.06266300 | -1.46381400 |

| | | | |
|---|---|---|---|
| H | -0.26856200 | 2.59437500 | -0.92031000 |
| O | 3.08388800 | -0.35225200 | -0.38765900 |
| C | 4.74901500 | -1.97962100 | -0.37419000 |
| C | 4.16713300 | -2.84900800 | -1.26765200 |
| N | 5.81844900 | -2.66238900 | 0.09350200 |
| H | 3.28056000 | -2.71528600 | -1.86845100 |
| N | 4.90373500 | -3.98966700 | -1.29725700 |
| N | 5.90232300 | -3.87053200 | -0.47637000 |
| C | 4.40507900 | -0.58858300 | 0.04499600 |
| H | 4.48241100 | -0.48003300 | 1.13655400 |
| H | 5.09707100 | 0.13381200 | -0.41149700 |
| N | -0.62282400 | -0.21700600 | -1.56251400 |
| O | -1.44058500 | 0.56259600 | -0.79847700 |
| H | 1.26794800 | -0.59631900 | -2.04071400 |
| C | 6.80991600 | -2.23288400 | 1.07589600 |
| H | 6.32577900 | -2.14502600 | 2.05299500 |
| H | 7.53688900 | -3.04706000 | 1.12622300 |
| C | 7.45082100 | -0.92488900 | 0.67214300 |
| C | 7.43369900 | 0.16909100 | 1.53607700 |
| C | 8.03546200 | -0.79811400 | -0.59100700 |
| C | 7.99786500 | 1.38274400 | 1.14351200 |
| H | 6.97258000 | 0.07422400 | 2.51625800 |
| C | 8.59547700 | 0.41258300 | -0.98419200 |
| H | 8.03998800 | -1.65028400 | -1.26696900 |
| C | 8.57637100 | 1.50572600 | -0.11688900 |
| H | 7.97861100 | 2.23104700 | 1.82030900 |
| H | 9.04554000 | 0.50649200 | -1.96747300 |
| H | 9.00992400 | 2.45142800 | -0.42674700 |
| O | 0.71154400 | 4.58295400 | 0.28744800 |
| C | 1.36329900 | 5.63471000 | 0.97490200 |
| H | 0.67820400 | 6.48163400 | 0.95471800 |
| H | 1.57299800 | 5.35912000 | 2.01480400 |
| H | 2.30042900 | 5.91138400 | 0.47819800 |

**TS2(EZ)**

| | | | |
|---|---|---|---|
| N | -8.05811700 | 0.92231300 | -1.86214900 |
| C | -7.07673100 | -0.07615600 | -1.45935500 |
| C | -5.81740800 | 0.63344200 | -0.94101700 |
| O | -4.85117500 | -0.32367700 | -0.58320900 |
| C | -7.55595800 | -1.00458100 | -0.36444200 |
| O | -7.24265700 | -2.17119000 | -0.26948000 |
| O | -3.24344900 | 0.38303600 | -2.35185600 |
| P | -3.02478400 | 0.14622900 | -0.89164700 |
| C | -2.61512100 | 2.45369300 | 0.50347200 |
| O | -2.72706300 | -1.32323200 | -0.26616600 |
| C | -3.34713100 | -2.49670300 | -0.81050800 |
| C | -2.34277900 | -3.62618700 | -0.70944500 |
| H | -8.89475300 | 0.46762000 | -2.22094700 |
| H | -6.79023100 | -0.68686900 | -2.32111400 |
| H | -5.43255800 | 1.27552000 | -1.73853000 |
| H | -6.07193800 | 1.26870200 | -0.07707100 |
| H | -8.20074700 | -0.52572500 | 0.40505900 |
| H | -3.09936900 | 2.90107700 | 1.37608300 |
| H | -1.58266900 | 2.21356100 | 0.76378200 |
| H | -4.25969600 | -2.71730900 | -0.24721300 |
| H | -3.63242500 | -2.31236900 | -1.85081300 |
| H | -2.77176800 | -4.55068000 | -1.10617900 |
| H | -2.06014600 | -3.79494700 | 0.33353200 |
| H | -1.44238900 | -3.38057400 | -1.27860600 |
| C | -4.52823300 | -0.97192300 | 3.99030900 |
| N | -5.52587600 | -1.87501600 | 3.70592200 |
| C | -4.21707400 | -0.37438100 | 2.80509700 |
| C | -5.80291300 | -1.82039200 | 2.39666400 |
| N | -5.02269500 | -0.91389700 | 1.82865500 |
| H | -3.48956200 | 0.38602900 | 2.57041200 |
| H | -6.54626500 | -2.41931900 | 1.89102300 |
| H | -4.96069300 | -0.63899800 | 0.65746800 |
| H | -4.13940800 | -0.84284400 | 4.98681700 |

| | | | |
|---|---|---|---|
| H | -8.35407900 | 1.46753700 | -1.05294600 |
| H | -5.98312700 | -2.48545800 | 4.37141900 |
| O | -3.35781300 | 1.24680900 | 0.26981500 |
| C | -2.68618300 | 3.39607900 | -0.68336700 |
| H | -3.72495400 | 3.66611300 | -0.89593600 |
| H | -2.11961000 | 4.30741800 | -0.46906300 |
| H | -2.26508600 | 2.92396500 | -1.57592400 |
| C | 2.76835900 | 3.15992600 | 0.60613000 |
| C | 3.35470400 | 1.89836600 | 0.45651700 |
| C | 2.67046700 | 0.87763000 | -0.19182100 |
| C | 1.37480400 | 1.10435300 | -0.70101900 |
| C | 0.81148800 | 2.36699400 | -0.55541400 |
| C | 1.49518700 | 3.39648500 | 0.09753700 |
| H | 3.32399600 | 3.93523900 | 1.11987800 |
| H | 4.34871500 | 1.74014900 | 0.85949700 |
| C | 0.70148700 | 0.01471900 | -1.43670800 |
| H | -0.17003700 | 2.57110600 | -0.96362800 |
| O | 3.16640000 | -0.38451300 | -0.37207900 |
| C | 4.82316400 | -2.02148000 | -0.31724500 |
| C | 4.24241200 | -2.90712500 | -1.19529600 |
| N | 5.88512900 | -2.69994900 | 0.17330500 |
| H | 3.36148100 | -2.78171600 | -1.80608100 |
| N | 4.97213700 | -4.05260800 | -1.19388400 |
| N | 5.96527400 | -3.92108200 | -0.36841700 |
| C | 4.48612200 | -0.61903500 | 0.06770300 |
| H | 4.56205200 | -0.48396500 | 1.15630000 |
| H | 5.18262800 | 0.08800900 | -0.40549400 |
| N | -0.54504200 | -0.26674800 | -1.50648400 |
| O | -1.34081300 | 0.54669000 | -0.73960100 |
| H | 1.33391600 | -0.66130100 | -2.00712600 |
| C | 6.86992000 | -2.25685100 | 1.15675000 |
| H | 6.37935000 | -2.15754000 | 2.12953600 |
| H | 7.59753100 | -3.06936500 | 1.22196800 |
| C | 7.51184400 | -0.95308600 | 0.74130200 |

| | | | |
|---|---|---|---|
| C | 7.48109600 | 0.15326700 | 1.58912300 |
| C | 8.11004500 | -0.84226200 | -0.51692500 |
| C | 8.04456500 | 1.36349400 | 1.18519100 |
| H | 7.00962000 | 0.07063600 | 2.56549500 |
| C | 8.66934100 | 0.36506200 | -0.92157500 |
| H | 8.12528300 | -1.70400000 | -1.18045900 |
| C | 8.63617700 | 1.47065100 | -0.07056100 |
| H | 8.01485800 | 2.22131600 | 1.84950200 |
| H | 9.12965500 | 0.44670900 | -1.90120900 |
| H | 9.06901900 | 2.41364900 | -0.38948800 |
| O | 0.83120600 | 4.58321900 | 0.18168100 |
| C | 1.50086900 | 5.65351700 | 0.82232000 |
| H | 0.82245000 | 6.50492200 | 0.78014800 |
| H | 1.72114700 | 5.41364300 | 1.86876900 |
| H | 2.43369200 | 5.90328700 | 0.30388400 |

**C2(EZ)**

| | | | |
|---|---|---|---|
| N | -9.09936200 | -0.69254500 | -1.13377100 |
| C | -7.64754700 | -0.82198100 | -1.03752500 |
| C | -7.06269000 | 0.47788100 | -0.46837700 |
| O | -5.65785200 | 0.46728800 | -0.45742300 |
| C | -7.23365400 | -1.97547400 | -0.14851500 |
| O | -6.42999500 | -2.83117800 | -0.44664600 |
| O | -3.06563900 | 0.73766200 | -2.91170600 |
| P | -2.65058100 | 0.81957600 | -1.50091500 |
| C | -3.20936800 | 2.14323800 | 0.71443900 |
| O | -2.90427600 | -0.46154000 | -0.60415500 |
| C | -3.57969400 | -1.62735500 | -1.13847400 |
| C | -2.61079000 | -2.50835400 | -1.89625800 |
| H | -9.50484800 | -1.54580700 | -1.51256400 |
| H | -7.22524500 | -0.98998800 | -2.03367200 |
| H | -7.40553100 | 1.30233400 | -1.10113900 |
| H | -7.46878000 | 0.64228900 | 0.54326200 |
| H | -7.74009000 | -1.99368600 | 0.84126900 |

| | | | |
|---|---|---|---|
| H | -3.41851600 | 1.15007600 | 1.12250500 |
| H | -2.20546500 | 2.45309600 | 1.02145500 |
| H | -3.98723600 | -2.13014500 | -0.26060100 |
| H | -4.41620700 | -1.30085700 | -1.75776200 |
| H | -3.12511600 | -3.41804000 | -2.22031700 |
| H | -1.76621100 | -2.78990700 | -1.26192200 |
| H | -2.22642200 | -1.98894100 | -2.77794100 |
| C | -4.84568400 | -0.90427000 | 4.18837600 |
| N | -4.94036600 | -2.17717200 | 3.68450300 |
| C | -5.04943000 | -0.07785900 | 3.11754200 |
| C | -5.19172600 | -2.08567800 | 2.36037400 |
| N | -5.26337800 | -0.82462100 | 1.98088900 |
| H | -5.06259500 | 1.00265200 | 3.09599900 |
| H | -5.33236200 | -2.94611200 | 1.71984100 |
| H | -5.38091400 | -0.03584000 | 0.34718200 |
| H | -4.64953900 | -0.71234900 | 5.23095400 |
| H | -9.50110400 | -0.57345000 | -0.20391400 |
| H | -4.84408500 | -3.03589800 | 4.20978000 |
| O | -3.22963900 | 2.07077900 | -0.72920400 |
| C | -4.26567900 | 3.13832400 | 1.13633300 |
| H | -4.24475700 | 3.26373500 | 2.22270500 |
| H | -4.08615400 | 4.11267800 | 0.67437100 |
| H | -5.25050400 | 2.77309300 | 0.83384100 |
| C | 3.27932000 | 3.20256000 | 0.50031800 |
| C | 3.73852500 | 1.88681300 | 0.40413300 |
| C | 2.96559500 | 0.91683500 | -0.22087600 |
| C | 1.70180400 | 1.25506500 | -0.75860500 |
| C | 1.25911200 | 2.57282200 | -0.65673300 |
| C | 2.04057800 | 3.54821100 | -0.03193900 |
| H | 3.90540000 | 3.93663200 | 0.99317900 |
| H | 4.70698600 | 1.64484600 | 0.82654700 |
| C | 0.96681100 | 0.18598100 | -1.45402500 |
| H | 0.30640300 | 2.87033600 | -1.07314100 |
| O | 3.34184300 | -0.38970000 | -0.34737400 |

| | | | |
|---|---|---|---|
| C | 4.84594600 | -2.16910300 | -0.22261800 |
| C | 4.19825200 | -3.02436300 | -1.08366000 |
| N | 5.84310500 | -2.92314600 | 0.29486300 |
| H | 3.33855500 | -2.83970800 | -1.70925900 |
| N | 4.82590600 | -4.22796900 | -1.04541400 |
| N | 5.82067500 | -4.16054900 | -0.21448900 |
| C | 4.62958100 | -0.73216600 | 0.12139200 |
| H | 4.69809400 | -0.57493100 | 1.20701800 |
| H | 5.39384000 | -0.10365000 | -0.35766700 |
| N | -0.27371700 | 0.04063200 | -1.72109800 |
| O | -1.06013600 | 1.10577000 | -1.24392000 |
| H | 1.55767700 | -0.65591700 | -1.80481000 |
| C | 6.85409300 | -2.54577900 | 1.28061400 |
| H | 6.37213400 | -2.42851600 | 2.25562700 |
| H | 7.53447100 | -3.39888700 | 1.33480800 |
| C | 7.56596400 | -1.27567900 | 0.87628600 |
| C | 7.57845400 | -0.17123800 | 1.72728800 |
| C | 8.17906200 | -1.18863300 | -0.37647100 |
| C | 8.19595300 | 1.01456700 | 1.33045100 |
| H | 7.09560600 | -0.23500500 | 2.69952200 |
| C | 8.79308300 | -0.00556400 | -0.77391400 |
| H | 8.16182300 | -2.04829500 | -1.04252900 |
| C | 8.80031500 | 1.09898300 | 0.07904800 |
| H | 8.19817900 | 1.87133700 | 1.99672400 |
| H | 9.26429900 | 0.05801100 | -1.74969600 |
| H | 9.27547900 | 2.02326300 | -0.23432800 |
| O | 1.50421300 | 4.79788000 | 0.00333900 |
| C | 2.27561900 | 5.81547300 | 0.61576200 |
| H | 1.68674300 | 6.72888600 | 0.54052700 |
| H | 2.46357700 | 5.58860000 | 1.67130200 |
| H | 3.23209200 | 5.95276800 | 0.09867900 |

**FE**

| | | | |
|---|---|---|---|
| C | -4.79088500 | -0.77541100 | -1.25709600 |

| | | | |
|---|---|---|---|
| C | -3.90579800 | 0.30442900 | -1.26057600 |
| C | -3.00082300 | 0.51639800 | -0.23205200 |
| C | -2.93638200 | -0.37217600 | 0.86976000 |
| C | -3.82833300 | -1.45349000 | 0.85327900 |
| C | -4.73962500 | -1.66278100 | -0.18226200 |
| H | -5.48893100 | -0.90373300 | -2.07505000 |
| H | -3.91789800 | 1.01751000 | -2.08011300 |
| C | -2.05276800 | -0.24126500 | 2.02214500 |
| H | -3.82415300 | -2.16209800 | 1.67733000 |
| H | -2.17507700 | -0.97025900 | 2.83081700 |
| O | -2.20013900 | 1.64091900 | -0.31065400 |
| N | -1.14010500 | 0.68087200 | 2.13920800 |
| O | -0.43527700 | 0.67597200 | 3.23586600 |
| C | -0.10943800 | 2.62782400 | -0.80675800 |
| C | -0.17761700 | 3.70553900 | -1.66358000 |
| N | 0.85606900 | 2.98607100 | 0.07474800 |
| H | -0.82221900 | 3.85110900 | -2.51830200 |
| N | 0.72898300 | 4.63369800 | -1.27646400 |
| N | 1.35760500 | 4.18633000 | -0.22699900 |
| C | -0.88589800 | 1.35230000 | -0.77835200 |
| H | -0.94623100 | 0.94479400 | -1.79571700 |
| H | -0.40412300 | 0.62131400 | -0.12335300 |
| C | 1.43019900 | 2.23340100 | 1.18892100 |
| H | 2.11318200 | 2.93766100 | 1.67230200 |
| H | 0.64931700 | 1.94578100 | 1.89894000 |
| C | 2.18115900 | 1.00916500 | 0.71333100 |
| C | 3.06086600 | 1.08464500 | -0.36862500 |
| C | 2.01150900 | -0.20393400 | 1.38250900 |
| C | 3.77883500 | -0.03421400 | -0.77810700 |
| H | 3.19024600 | 2.02739300 | -0.89555200 |
| C | 2.72919400 | -1.33062600 | 0.98526700 |
| H | 1.30394200 | -0.24966600 | 2.20952900 |
| C | 3.60576700 | -1.22966000 | -0.08781900 |
| H | 4.46484800 | 0.02049100 | -1.61611100 |

| | | | |
|---|---|---|---|
| H | 2.60521600 | -2.27772100 | 1.49894300 |
| O | -5.54182400 | -2.75883900 | -0.05199200 |
| C | -6.47716400 | -3.00041500 | -1.08544000 |
| H | -5.97593700 | -3.15583800 | -2.04777300 |
| H | -7.01396400 | -3.90654000 | -0.80536700 |
| H | -7.18742500 | -2.17094400 | -1.18043700 |
| Br | 4.59635600 | -2.76059600 | -0.61990200 |

**C1(FE)**

| | | | |
|---|---|---|---|
| N | -6.84804300 | -2.78335300 | -2.14134100 |
| C | -5.45583800 | -2.59621400 | -1.73624700 |
| C | -5.20327300 | -1.09264000 | -1.64136700 |
| O | -3.98995800 | -0.75705200 | -0.95031900 |
| C | -5.18008300 | -3.33345700 | -0.43927300 |
| O | -4.99804700 | -2.80675600 | 0.63939500 |
| O | -2.51897800 | -2.77937900 | -1.71559600 |
| P | -2.55858200 | -1.32803900 | -1.44499000 |
| C | -2.17080500 | 0.97659700 | -2.66784600 |
| O | -1.70180900 | -0.81362100 | -0.21109700 |
| C | -0.84891700 | -1.78914600 | 0.45329300 |
| C | -0.05898200 | -1.07449500 | 1.52125600 |
| H | -7.00876200 | -3.73610100 | -2.45822800 |
| H | -4.81697600 | -3.04254200 | -2.50281300 |
| H | -5.19552900 | -0.65621900 | -2.64458800 |
| H | -5.99885900 | -0.61903200 | -1.06127700 |
| H | -5.23904000 | -4.43509500 | -0.51605200 |
| H | -3.13479800 | 1.34618200 | -3.02872000 |
| H | -2.03980000 | 1.29294600 | -1.62935600 |
| H | -1.49686200 | -2.56150000 | 0.88068400 |
| H | -0.18865500 | -2.22655700 | -0.29581600 |
| H | 0.52279800 | -1.80911200 | 2.08612000 |
| H | -0.71976800 | -0.54523000 | 2.21716800 |
| H | 0.63309900 | -0.36064500 | 1.06691600 |
| C | -3.04139800 | 0.77378200 | 3.38937000 |

| | | | |
|---|---|---|---|
| N | -3.12978100 | -0.50683700 | 3.88831900 |
| C | -3.51903500 | 0.72027500 | 2.11442600 |
| C | -3.63830800 | -1.31709500 | 2.96052900 |
| N | -3.87885800 | -0.58478200 | 1.88226500 |
| H | -3.64024400 | 1.49060800 | 1.36952400 |
| H | -3.82246500 | -2.37539800 | 3.05969300 |
| H | -4.23610300 | -0.97053300 | 1.00128700 |
| H | -2.64750500 | 1.59400000 | 3.96673500 |
| H | -7.47710500 | -2.61457100 | -1.35804100 |
| H | -2.85347100 | -0.79915200 | 4.81884500 |
| O | -2.21466600 | -0.47066700 | -2.72901800 |
| C | -1.01525900 | 1.43870200 | -3.52651500 |
| H | -1.19319100 | 1.18846000 | -4.57635200 |
| H | -0.89921900 | 2.52370200 | -3.44038300 |
| H | -0.10390900 | 0.93867600 | -3.18356300 |
| C | 5.88559300 | -3.02056300 | 1.22515300 |
| C | 5.74778700 | -1.78038300 | 0.60073900 |
| C | 4.61373500 | -1.44587100 | -0.12554200 |
| C | 3.54130700 | -2.35887000 | -0.24720700 |
| C | 3.70230100 | -3.60605700 | 0.37084500 |
| C | 4.84425400 | -3.94067300 | 1.09969200 |
| H | 6.78689700 | -3.24818600 | 1.78078600 |
| H | 6.54888300 | -1.04862100 | 0.66109800 |
| C | 2.30904900 | -2.12342400 | -0.99576400 |
| H | 2.91088500 | -4.34695500 | 0.29436800 |
| H | 1.71484000 | -3.00188200 | -1.27079300 |
| O | 4.60241300 | -0.22090200 | -0.76777000 |
| N | 1.86363000 | -0.94057500 | -1.30338100 |
| O | 0.72637700 | -0.88830100 | -1.94525200 |
| C | 4.39198300 | 2.12719000 | -0.64767400 |
| C | 5.47726700 | 2.97473100 | -0.59389600 |
| N | 3.59231300 | 2.69945800 | -1.57842300 |
| H | 6.36888100 | 2.89958900 | 0.01139500 |
| N | 5.27910800 | 3.99574800 | -1.46249700 |

| | | | |
|---|---|---|---|
| N | 4.12939700 | 3.82391600 | -2.05144000 |
| C | 4.09068800 | 0.83696900 | 0.03855500 |
| H | 4.58603000 | 0.83271000 | 1.01684800 |
| H | 3.01092400 | 0.70063700 | 0.17531600 |
| C | 2.24012400 | 2.30727900 | -1.96359800 |
| H | 2.05540000 | 2.77913600 | -2.93245100 |
| H | 2.21004900 | 1.21835600 | -2.07333700 |
| C | 1.23044100 | 2.74676500 | -0.92502400 |
| C | 1.23458900 | 4.04606600 | -0.41118400 |
| C | 0.28531200 | 1.82717300 | -0.47371400 |
| C | 0.29637600 | 4.43436900 | 0.54114600 |
| H | 1.97618400 | 4.76282400 | -0.75577200 |
| C | -0.65209700 | 2.20284700 | 0.48591200 |
| H | 0.27497900 | 0.81532200 | -0.88954500 |
| C | -0.63920700 | 3.49994500 | 0.97567100 |
| H | 0.29139500 | 5.44371700 | 0.93770900 |
| H | -1.37496500 | 1.47701300 | 0.84163300 |
| O | 4.84950100 | -5.18718000 | 1.65249600 |
| C | 5.99431400 | -5.56045200 | 2.39563700 |
| H | 6.14591900 | -4.89442700 | 3.25292000 |
| H | 5.80874200 | -6.57347400 | 2.75200300 |
| H | 6.89427800 | -5.55270800 | 1.77030400 |
| Br | -1.94682200 | 4.00743900 | 2.26426000 |

**TS1(FE)**

| | | | |
|---|---|---|---|
| N | -6.20091100 | 2.69475200 | 2.09099800 |
| C | -5.08226900 | 2.25009300 | 1.26110800 |
| C | -4.60749100 | 0.88018300 | 1.72040900 |
| O | -3.51165300 | 0.44877500 | 0.92664700 |
| C | -5.48230800 | 2.24742600 | -0.20084100 |
| O | -5.46503600 | 1.27776100 | -0.93301600 |
| O | -2.02182200 | 2.12419000 | 2.05699900 |
| P | -1.97348000 | 0.75256500 | 1.48815300 |
| C | -1.17366700 | -0.82280500 | 3.59042500 |

| | | | |
|---|---|---|---|
| O | -1.28638200 | 0.39870600 | 0.08059400 |
| C | -1.60925600 | 1.19738900 | -1.07130100 |
| C | -0.65976500 | 0.78976100 | -2.17522100 |
| H | -6.39458600 | 3.68150400 | 1.93896500 |
| H | -4.25166100 | 2.95507200 | 1.38625500 |
| H | -4.34287400 | 0.92950300 | 2.78140200 |
| H | -5.40812800 | 0.14102800 | 1.59963500 |
| H | -5.87853300 | 3.20983000 | -0.57681400 |
| H | -1.62659500 | -1.73869600 | 3.97814800 |
| H | -0.13558500 | -1.01071500 | 3.32111300 |
| H | -2.65378900 | 1.02686500 | -1.35183200 |
| H | -1.49243100 | 2.25726700 | -0.81919200 |
| H | -0.84260300 | 1.39226800 | -3.06921900 |
| H | -0.80242800 | -0.26524500 | -2.42979400 |
| H | 0.37415600 | 0.93984700 | -1.85002300 |
| C | -3.09827700 | -3.05391100 | -1.67839600 |
| N | -4.00569300 | -2.75000100 | -2.66917500 |
| C | -3.19631500 | -2.06071500 | -0.75068800 |
| C | -4.63864000 | -1.61816700 | -2.36589500 |
| N | -4.15876000 | -1.19154500 | -1.20611700 |
| H | -2.67790900 | -1.88724200 | 0.17990700 |
| H | -5.40132800 | -1.13585400 | -2.95636700 |
| H | -4.44772200 | -0.32176400 | -0.73905400 |
| H | -2.45821200 | -3.92082800 | -1.73265100 |
| H | -7.04984900 | 2.18613000 | 1.84932900 |
| H | -4.17320700 | -3.29680000 | -3.50601600 |
| O | -1.91445200 | -0.56896000 | 2.37996500 |
| C | -1.28580600 | 0.30357800 | 4.59662600 |
| H | -2.33514200 | 0.52675600 | 4.81465400 |
| H | -0.79720900 | 0.00179300 | 5.52818400 |
| H | -0.79969000 | 1.19907600 | 4.20838300 |
| C | 3.40471800 | 4.45347700 | -2.18843400 |
| C | 3.97072000 | 3.49139600 | -1.35116700 |
| C | 3.21601200 | 2.79603300 | -0.41739200 |

| | | | |
|---|---|---|---|
| C | 1.82914500 | 3.03917000 | -0.29546300 |
| C | 1.27781600 | 4.01525100 | -1.13220800 |
| C | 2.03969900 | 4.71474900 | -2.07008100 |
| H | 4.02709700 | 4.97812200 | -2.90295700 |
| H | 5.03440900 | 3.27745100 | -1.40736600 |
| C | 0.92531600 | 2.37177000 | 0.64678500 |
| H | 0.21742200 | 4.24530300 | -1.06413700 |
| H | -0.06049700 | 2.81911400 | 0.79517400 |
| O | 3.87556100 | 1.90900100 | 0.41148600 |
| N | 1.24429600 | 1.28326000 | 1.26526500 |
| O | 0.34104900 | 0.73145300 | 2.05441800 |
| C | 4.71930500 | -0.26328100 | 0.82155600 |
| C | 6.08487100 | -0.41232600 | 0.93526600 |
| N | 4.23318700 | -1.06302600 | 1.80188000 |
| H | 6.86798100 | 0.05295800 | 0.35440400 |
| N | 6.35042000 | -1.27701400 | 1.94308700 |
| N | 5.22087900 | -1.67280800 | 2.45842300 |
| C | 3.89107500 | 0.57598200 | -0.09456200 |
| H | 4.34668400 | 0.56951600 | -1.09258000 |
| H | 2.86747100 | 0.19292500 | -0.15896300 |
| C | 2.84380900 | -1.38442000 | 2.12386600 |
| H | 2.88564700 | -1.88797200 | 3.09326800 |
| H | 2.26956300 | -0.45948400 | 2.21828100 |
| C | 2.22542200 | -2.27625300 | 1.06924500 |
| C | 2.87158000 | -3.44079500 | 0.64895400 |
| C | 0.99717500 | -1.92485700 | 0.50945200 |
| C | 2.29521900 | -4.26149900 | -0.31561000 |
| H | 3.83430500 | -3.71002400 | 1.07763800 |
| C | 0.41851400 | -2.73069800 | -0.46826700 |
| H | 0.49553700 | -1.01887700 | 0.84607500 |
| C | 1.06989800 | -3.89157500 | -0.86160400 |
| H | 2.78800600 | -5.17155200 | -0.63963000 |
| H | -0.52314200 | -2.44369800 | -0.92317600 |
| Br | 0.26700400 | -5.00285300 | -2.17983800 |

| | | | |
|---|---|---|---|
| O | 1.36310800 | 5.63017800 | -2.81850000 |
| C | 2.10723500 | 6.35808200 | -3.77778400 |
| H | 2.54348100 | 5.69083000 | -4.52987000 |
| H | 1.40263100 | 7.03633700 | -4.25819800 |
| H | 2.90645000 | 6.93826400 | -3.30289200 |

**IN(FE)**

| | | | |
|---|---|---|---|
| N | 5.72011500 | -4.25596900 | 1.89390400 |
| C | 4.92818100 | -3.56494200 | 0.88562600 |
| C | 3.82504400 | -2.75513100 | 1.58142600 |
| O | 3.01464600 | -2.15045400 | 0.60706300 |
| C | 5.72500000 | -2.61030600 | 0.02478700 |
| O | 5.46375500 | -2.35361400 | -1.13164900 |
| O | 0.95322200 | -2.94281600 | 1.71890100 |
| P | 1.38965100 | -1.64103100 | 1.10869800 |
| C | 1.65252900 | 0.44416600 | 2.90820200 |
| O | 1.13199200 | -1.25561300 | -0.46668600 |
| C | 1.46516700 | -2.15456700 | -1.52927300 |
| C | 0.57674400 | -1.79484200 | -2.70303500 |
| H | 6.45357300 | -4.80495700 | 1.45110200 |
| H | 4.44712900 | -4.29422300 | 0.22601900 |
| H | 3.23921600 | -3.44319200 | 2.19866000 |
| H | 4.27615200 | -1.99603400 | 2.24067000 |
| H | 6.57086700 | -2.10523300 | 0.53867500 |
| H | 2.50984500 | 1.06967500 | 3.17427700 |
| H | 0.88788300 | 1.07928600 | 2.45443500 |
| H | 2.52611500 | -2.05633800 | -1.78162500 |
| H | 1.29583800 | -3.18871500 | -1.20558600 |
| H | 0.79862100 | -2.43694100 | -3.56005900 |
| H | 0.74239600 | -0.75327300 | -2.99555800 |
| H | -0.47799100 | -1.91592300 | -2.43544200 |
| C | 3.44574200 | 2.23803100 | -1.30104600 |
| N | 4.59086500 | 1.72533400 | -1.87030600 |
| C | 2.93294200 | 1.24602000 | -0.52261200 |

| | | | |
|---|---|---|---|
| C | 4.77219200 | 0.46992200 | -1.46053100 |
| N | 3.77590200 | 0.16463200 | -0.63896200 |
| H | 2.04781600 | 1.20410900 | 0.09097800 |
| H | 5.57598800 | -0.19059800 | -1.74695700 |
| H | 3.61289200 | -0.76248500 | -0.17039600 |
| H | 3.09385700 | 3.23567100 | -1.51544900 |
| H | 6.19052300 | -3.57905300 | 2.49381200 |
| H | 5.20663500 | 2.21642500 | -2.50742700 |
| O | 2.17707000 | -0.46016100 | 1.92791100 |
| C | 1.12242700 | -0.27400600 | 4.13408900 |
| H | 1.89816300 | -0.91639800 | 4.56238900 |
| H | 0.82144600 | 0.45621400 | 4.89168700 |
| H | 0.25800300 | -0.88738200 | 3.87227500 |
| C | -5.23110300 | -3.26737200 | -1.53956200 |
| C | -5.20979300 | -2.18098800 | -0.66556900 |
| C | -4.03927000 | -1.76153100 | -0.04849700 |
| C | -2.82996900 | -2.44138400 | -0.30219400 |
| C | -2.86342100 | -3.53596000 | -1.16798900 |
| C | -4.04161400 | -3.95033500 | -1.79327100 |
| H | -6.16567900 | -3.56423600 | -1.99990100 |
| H | -6.12627900 | -1.64228900 | -0.44316900 |
| C | -1.52037600 | -2.11843600 | 0.29304800 |
| H | -1.95108400 | -4.08977900 | -1.37430400 |
| H | -0.74709800 | -2.87772400 | 0.19038000 |
| O | -4.11557700 | -0.70202500 | 0.83272900 |
| N | -1.32875300 | -1.00840100 | 0.89878900 |
| O | -0.11475400 | -0.70836100 | 1.42700000 |
| C | -4.21825100 | 1.64705900 | 1.17682300 |
| C | -5.38094400 | 2.30639100 | 1.51208300 |
| N | -3.29566800 | 2.19782800 | 2.00272100 |
| H | -6.37321800 | 2.17980000 | 1.10435900 |
| N | -5.11015900 | 3.20287300 | 2.49092700 |
| N | -3.84114700 | 3.13321700 | 2.77871600 |
| C | -3.94357000 | 0.56149900 | 0.19066200 |

| | | | |
|---|---|---|---|
| H | -4.65470600 | 0.65288800 | -0.63852300 |
| H | -2.92529900 | 0.63686000 | -0.20645000 |
| C | -1.85406500 | 1.95564300 | 2.05488900 |
| H | -1.53906200 | 2.26063500 | 3.05556600 |
| H | -1.68767300 | 0.88465200 | 1.93207400 |
| C | -1.12546200 | 2.74555200 | 0.98886200 |
| C | -1.03688200 | 4.13666600 | 1.09095000 |
| C | -0.55473200 | 2.10187500 | -0.11019400 |
| C | -0.37624300 | 4.88249600 | 0.12096500 |
| H | -1.48753900 | 4.64156800 | 1.94172500 |
| C | 0.09043500 | 2.83901000 | -1.10235600 |
| H | -0.59556100 | 1.01803000 | -0.18893700 |
| C | 0.18224400 | 4.21760200 | -0.96658900 |
| H | -0.29292500 | 5.96021400 | 0.20679300 |
| H | 0.52767800 | 2.34223000 | -1.96249500 |
| O | -3.92826600 | -5.02272000 | -2.61993200 |
| C | -5.10533100 | -5.46744300 | -3.27098600 |
| H | -5.52045700 | -4.68415300 | -3.91508500 |
| H | -4.81031000 | -6.32111300 | -3.87991500 |
| H | -5.86410100 | -5.78092100 | -2.54522800 |
| Br | 1.10951700 | 5.20655900 | -2.29455200 |

**TS2(FE)**

| | | | |
|---|---|---|---|
| N | 4.30677000 | -5.57460400 | 0.99589100 |
| C | 3.54814200 | -4.59405500 | 0.23277000 |
| C | 2.95534500 | -3.55695000 | 1.19688500 |
| O | 2.21439800 | -2.61244100 | 0.45599100 |
| C | 4.36414700 | -3.85000400 | -0.80728500 |
| O | 3.92507700 | -3.45881300 | -1.86601800 |
| O | 0.04454900 | -3.20778000 | 1.54398900 |
| P | 0.63422200 | -1.87049100 | 1.22302500 |
| C | 1.45085600 | -0.39433500 | 3.38027700 |
| O | 0.46702300 | -1.18780900 | -0.24387400 |
| C | 0.51055000 | -1.94781700 | -1.46108700 |

| | | | |
|---|---|---|---|
| C | -0.39130200 | -1.24729600 | -2.45665700 |
| H | 4.67550600 | -6.29307000 | 0.37692700 |
| H | 2.71729700 | -5.08547000 | -0.28337800 |
| H | 2.29690000 | -4.06869900 | 1.90288900 |
| H | 3.75794900 | -3.05662300 | 1.76018600 |
| H | 5.41777200 | -3.64446800 | -0.51838300 |
| H | 2.43088100 | -0.01743300 | 3.68680100 |
| H | 0.80339600 | 0.45417100 | 3.15460600 |
| H | 1.54336700 | -1.99866800 | -1.81804200 |
| H | 0.17348400 | -2.97174500 | -1.26517400 |
| H | -0.35513000 | -1.76161000 | -3.42114000 |
| H | -0.06592700 | -0.21265200 | -2.60235600 |
| H | -1.42766200 | -1.23793100 | -2.10483400 |
| C | 4.58529700 | 1.31996900 | -0.14354800 |
| N | 4.67918500 | 0.77908200 | -1.40370100 |
| C | 3.92516300 | 0.39324700 | 0.60865600 |
| C | 4.09869400 | -0.42963400 | -1.40153700 |
| N | 3.63163600 | -0.68740800 | -0.19012400 |
| H | 3.62984000 | 0.42207600 | 1.64487300 |
| H | 4.04822300 | -1.09316400 | -2.25200700 |
| H | 2.92617400 | -1.67893200 | 0.13151800 |
| H | 4.96782800 | 2.30123400 | 0.08623000 |
| H | 5.11366600 | -5.13059300 | 1.43326000 |
| H | 5.09630800 | 1.22679100 | -2.21006600 |
| O | 1.72569200 | -1.12656600 | 2.17296700 |
| C | 0.86780000 | -1.28328400 | 4.46117300 |
| H | 1.52419800 | -2.14043400 | 4.63880600 |
| H | 0.77359200 | -0.72021400 | 5.39428100 |
| H | -0.12002100 | -1.64739100 | 4.17020600 |
| C | -6.10640800 | -1.56405000 | -1.43060700 |
| C | -5.70937000 | -0.42785900 | -0.72645200 |
| C | -4.49014000 | -0.36796000 | -0.06631800 |
| C | -3.61852500 | -1.47478300 | -0.10286500 |
| C | -4.02131400 | -2.60619200 | -0.81483400 |

| | | | |
|---|---|---|---|
| C | -5.24963300 | -2.66386400 | -1.47698300 |
| H | -7.06784900 | -1.57368400 | -1.92973800 |
| H | -6.35957300 | 0.44035600 | -0.67825100 |
| C | -2.30461800 | -1.56224200 | 0.55715300 |
| H | -3.37310700 | -3.47696100 | -0.86031800 |
| H | -1.77458200 | -2.50747800 | 0.43480500 |
| O | -4.18903500 | 0.78280600 | 0.62901600 |
| N | -1.82360400 | -0.60420200 | 1.25443200 |
| O | -0.58574600 | -0.77569200 | 1.82712300 |
| C | -3.42431300 | 3.02165600 | 0.59140900 |
| C | -4.29762000 | 4.08764100 | 0.58303700 |
| N | -2.50186200 | 3.37748400 | 1.51715200 |
| H | -5.19446800 | 4.22609600 | -0.00307000 |
| N | -3.86507500 | 5.01415700 | 1.47174800 |
| N | -2.77188900 | 4.57710800 | 2.03009900 |
| C | -3.43661100 | 1.72229400 | -0.14071400 |
| H | -3.91707700 | 1.86844100 | -1.11459900 |
| H | -2.41960900 | 1.34372800 | -0.30187200 |
| C | -1.27221900 | 2.68083400 | 1.89035700 |
| H | -1.01706900 | 3.04278900 | 2.88897800 |
| H | -1.49234800 | 1.61070700 | 1.93937700 |
| C | -0.16262700 | 2.95735900 | 0.89931000 |
| C | 0.32693200 | 4.25329700 | 0.71892500 |
| C | 0.36681900 | 1.91189800 | 0.14607300 |
| C | 1.33800200 | 4.50281700 | -0.20315200 |
| H | -0.08683300 | 5.07434700 | 1.29913000 |
| C | 1.37857600 | 2.14575300 | -0.78359300 |
| H | -0.00465000 | 0.90155300 | 0.28150700 |
| C | 1.84995500 | 3.44156100 | -0.94541200 |
| H | 1.72550900 | 5.50554400 | -0.34562100 |
| H | 1.78741600 | 1.32515900 | -1.36744900 |
| Br | 3.22324600 | 3.78108200 | -2.21265200 |
| O | -5.51787600 | -3.82435500 | -2.12881000 |
| C | -6.76242600 | -3.91930900 | -2.79990000 |

| | | | |
|---|---|---|---|
| H | -6.84805600 | -3.15965600 | -3.58473700 |
| H | -6.78841500 | -4.91129000 | -3.24935200 |
| H | -7.59755200 | -3.81364800 | -2.09857900 |

**C2(FE)**

| | | | |
|---|---|---|---|
| N | 1.48074700 | 6.25187900 | 0.41037300 |
| C | 1.35600000 | 4.88547500 | 0.91886100 |
| C | 2.44293300 | 4.01659100 | 0.30072400 |
| O | 2.41812400 | 2.76178500 | 0.93884900 |
| C | 1.48972600 | 4.83748700 | 2.42712800 |
| O | 0.63436700 | 4.44802700 | 3.18940100 |
| O | -0.21504200 | 2.64893500 | -0.77849500 |
| P | 0.32013000 | 1.27750600 | -0.68611500 |
| C | 2.39565800 | -0.21149400 | -1.19536600 |
| O | 0.38915600 | 0.62613400 | 0.75696800 |
| C | 0.02797100 | 1.37914600 | 1.94032500 |
| C | -1.32317800 | 0.91580400 | 2.43942100 |
| H | 0.72690200 | 6.82944200 | 0.77627800 |
| H | 0.37788700 | 4.48197600 | 0.64188000 |
| H | 2.25740300 | 3.93893900 | -0.77953100 |
| H | 3.42008300 | 4.51001700 | 0.44433300 |
| H | 2.45102200 | 5.24228300 | 2.81523800 |
| H | 2.87747600 | -0.17607200 | -0.21296100 |
| H | 1.64842700 | -1.01003500 | -1.19983000 |
| H | 0.82118100 | 1.18960500 | 2.66547400 |
| H | 0.02990400 | 2.44317000 | 1.70719000 |
| H | -1.58834400 | 1.46773400 | 3.34607100 |
| H | -1.30813200 | -0.15187300 | 2.67845100 |
| H | -2.09558300 | 1.09686700 | 1.68446700 |
| C | 6.56386000 | 0.61513800 | -0.85503700 |
| N | 6.39120300 | 1.72967300 | -1.63666100 |
| C | 5.57314300 | 0.67798500 | 0.08691800 |
| C | 5.32669600 | 2.41261300 | -1.15763900 |
| N | 4.80611400 | 1.80194000 | -0.11246400 |

| | | | |
|---|---|---|---|
| H | 5.35310000 | -0.02421700 | 0.87740700 |
| H | 4.97392100 | 3.33385900 | -1.59899100 |
| H | 3.22441400 | 2.28348400 | 0.62488800 |
| H | 7.35188600 | -0.09779600 | -1.03744900 |
| H | 2.35019600 | 6.66846900 | 0.74388700 |
| H | 6.95834900 | 1.99568700 | -2.43037800 |
| O | 1.71626200 | 1.05344700 | -1.39862100 |
| C | 3.40527500 | -0.40946100 | -2.30091200 |
| H | 3.99581800 | -1.30372600 | -2.07686300 |
| H | 2.90759600 | -0.54166700 | -3.26520300 |
| H | 4.08379500 | 0.44556500 | -2.36110800 |
| C | -6.82112800 | 0.89147900 | -0.30075900 |
| C | -6.11262600 | -0.29770200 | -0.13908100 |
| C | -4.77109900 | -0.39280300 | -0.48164600 |
| C | -4.10926300 | 0.73203600 | -1.00775500 |
| C | -4.82744500 | 1.91323600 | -1.19216700 |
| C | -6.17328900 | 2.00863100 | -0.83138700 |
| H | -7.86723200 | 0.92644400 | -0.02132700 |
| H | -6.60903900 | -1.18078100 | 0.25133500 |
| C | -2.69482900 | 0.75808900 | -1.40532800 |
| H | -4.34227100 | 2.78841700 | -1.61414300 |
| H | -2.37965800 | 1.54775200 | -2.08957000 |
| O | -4.14393500 | -1.61176500 | -0.36098600 |
| N | -1.83721800 | -0.07019900 | -0.94744900 |
| O | -0.55608400 | 0.17661500 | -1.52079500 |
| C | -3.23337300 | -3.29611300 | 1.03426800 |
| C | -3.66548400 | -4.28926500 | 1.88399400 |
| N | -2.35467900 | -3.93246900 | 0.22252400 |
| H | -4.37745800 | -4.22791500 | 2.69371300 |
| N | -3.03887400 | -5.44622700 | 1.55443200 |
| N | -2.24871000 | -5.22152600 | 0.54580800 |
| C | -3.57471800 | -1.84923800 | 0.92943800 |
| H | -4.29560400 | -1.60000300 | 1.71567200 |
| H | -2.68615900 | -1.21759700 | 1.04650800 |

| | | | |
|---|---|---|---|
| C | -1.54575800 | -3.38460300 | -0.86291600 |
| H | -1.68618100 | -4.02575900 | -1.73571000 |
| H | -1.95029300 | -2.39319900 | -1.08492300 |
| C | -0.08501300 | -3.30114100 | -0.48584800 |
| C | 0.89044800 | -3.83880100 | -1.32400300 |
| C | 0.30460500 | -2.66407300 | 0.69471400 |
| C | 2.24272800 | -3.73018600 | -1.00533300 |
| H | 0.59856000 | -4.34434200 | -2.24016100 |
| C | 1.64762100 | -2.55729100 | 1.03520900 |
| H | -0.44404600 | -2.23475500 | 1.35574300 |
| C | 2.60168400 | -3.08388300 | 0.17011400 |
| H | 3.00234300 | -4.13364500 | -1.66588100 |
| H | 1.95192500 | -2.03895600 | 1.93798100 |
| O | -6.75790000 | 3.21328600 | -1.03980600 |
| C | -8.12586100 | 3.34508200 | -0.68950500 |
| H | -8.27894000 | 3.15895800 | 0.37902800 |
| H | -8.39736500 | 4.37375200 | -0.92263200 |
| H | -8.75009200 | 2.66010700 | -1.27358000 |
| Br | 4.43974000 | -2.81892600 | 0.54885000 |

**FZ**

| | | | |
|---|---|---|---|
| C | 2.39482200 | -2.22190100 | 0.73387500 |
| C | 1.68774000 | -1.01376900 | 0.62290600 |
| C | 2.30813400 | 0.12989200 | 0.15280800 |
| C | 3.68117700 | 0.12512000 | -0.23053900 |
| C | 4.36403200 | -1.09046200 | -0.10653100 |
| C | 3.73275800 | -2.24787500 | 0.36653800 |
| H | 1.88130800 | -3.10111600 | 1.10323100 |
| H | 0.64301500 | -1.00371800 | 0.91429700 |
| C | 4.28211600 | 1.35931300 | -0.72097100 |
| H | 5.40688000 | -1.12005900 | -0.38778800 |
| O | 1.66007900 | 1.33863200 | 0.02455500 |
| C | -0.17142900 | 2.76778100 | 0.12011500 |
| C | 0.34129700 | 3.78698300 | -0.64841000 |

| | | | |
|---|---|---|---|
| N | -1.32389400 | 3.28038300 | 0.60902100 |
| H | 1.25511200 | 3.81025900 | -1.22191200 |
| N | -0.51270500 | 4.84194000 | -0.58885800 |
| N | -1.51923300 | 4.53013100 | 0.16858500 |
| C | 0.30685200 | 1.38073800 | 0.39916000 |
| H | 0.18387200 | 1.13289400 | 1.46479600 |
| H | -0.28386200 | 0.65282400 | -0.17760300 |
| N | 5.50946500 | 1.57966800 | -1.14451300 |
| O | 6.37120900 | 0.61182700 | -1.15709400 |
| H | 3.64326700 | 2.23593800 | -0.74857100 |
| C | -2.29098400 | 2.64889000 | 1.49958000 |
| H | -1.82507300 | 2.49077700 | 2.47664800 |
| H | -3.09483000 | 3.37924500 | 1.61918600 |
| C | -2.79856600 | 1.34410700 | 0.93014600 |
| C | -2.73719400 | 0.17302800 | 1.68332300 |
| C | -3.31564600 | 1.29955600 | -0.36689300 |
| C | -3.19347500 | -1.03428600 | 1.15823200 |
| H | -2.32762700 | 0.19516000 | 2.68965900 |
| C | -3.77213600 | 0.10263600 | -0.90633600 |
| H | -3.35545500 | 2.20756100 | -0.96351600 |
| C | -3.70690800 | -1.05268600 | -0.13183200 |
| H | -3.14732900 | -1.94578700 | 1.74337000 |
| H | -4.17350100 | 0.06451800 | -1.91283000 |
| O | 4.52739300 | -3.36159400 | 0.43077900 |
| C | 3.91953700 | -4.55738600 | 0.87212800 |
| H | 3.09035400 | -4.84927200 | 0.21627100 |
| H | 4.69313300 | -5.32500600 | 0.84035500 |
| H | 3.54485800 | -4.46085000 | 1.89829900 |
| Br | -4.33919400 | -2.68849200 | -0.85180700 |

**C1(FZ)**

| | | | |
|---|---|---|---|
| N | 9.18649100 | -1.83259800 | -2.01854200 |
| C | 7.99372800 | -1.06301300 | -1.66635800 |
| C | 8.34452400 | 0.40327500 | -1.50042800 |

| | | | |
|---|---|---|---|
| O | 8.41037000 | 0.97291400 | -0.43124300 |
| C | 7.40591400 | -1.66729300 | -0.39521400 |
| O | 6.36552700 | -0.86693700 | 0.18222100 |
| C | 3.56678400 | -2.58769200 | -0.13516100 |
| C | 2.79085100 | -3.61106100 | -0.93034600 |
| C | 3.49584500 | 1.40932100 | 0.32557400 |
| C | 2.28778700 | 1.32973800 | 1.22738800 |
| O | 4.43080200 | -1.88531400 | -1.06192100 |
| O | 4.24743200 | 0.17935400 | 0.50153800 |
| O | 5.35606300 | 0.36505200 | -1.86815300 |
| P | 5.05033100 | -0.47706100 | -0.69262700 |
| H | 7.02519600 | -2.67056100 | -0.60610100 |
| H | 8.17756400 | -1.73645500 | 0.37500600 |
| H | 9.48616000 | -1.61945200 | -2.96666400 |
| H | 9.95996700 | -1.59687200 | -1.39877200 |
| H | 7.28336200 | -1.14483900 | -2.49419600 |
| H | 8.63619900 | 0.92354500 | -2.43199700 |
| H | 4.19596400 | -3.04833400 | 0.63437300 |
| H | 2.89664200 | -1.85466400 | 0.31654700 |
| H | 2.23247700 | -3.09168100 | -1.71361000 |
| H | 3.45775800 | -4.35001100 | -1.38297100 |
| H | 2.07963700 | -4.12233900 | -0.27475200 |
| H | 1.67340400 | 0.47906400 | 0.91787300 |
| H | 2.58898500 | 1.21301500 | 2.27331700 |
| H | 1.69719800 | 2.24576300 | 1.13579500 |
| H | 3.19952900 | 1.50611300 | -0.71751200 |
| H | 4.16497800 | 2.23082100 | 0.60444300 |
| C | 5.81566000 | 1.47248800 | 2.79290600 |
| N | 6.73255100 | 1.45240400 | 1.76750100 |
| C | 5.87266100 | 2.71565100 | 3.34308400 |
| C | 7.33531200 | 2.63081200 | 1.67995600 |
| N | 6.82485400 | 3.41135900 | 2.63022000 |
| H | 5.33232900 | 3.16120600 | 4.16150800 |
| H | 8.09433200 | 2.89579600 | 0.96113300 |

| | | | |
|---|---|---|---|
| H | 7.10159200 | 4.37240200 | 2.79494400 |
| H | 5.20555400 | 0.61558100 | 3.02693700 |
| H | 6.91586300 | 0.66108500 | 1.13825800 |
| C | -2.15288200 | -2.17408100 | 1.19251800 |
| C | -2.73244200 | -0.97520000 | 0.74629400 |
| C | -2.02580600 | -0.10267200 | -0.06313400 |
| C | -0.69120800 | -0.38988100 | -0.46918900 |
| C | -0.13786800 | -1.59110900 | -0.01298300 |
| C | -0.85437100 | -2.47239300 | 0.80655900 |
| H | -2.73278300 | -2.83481200 | 1.82541200 |
| H | -3.74817700 | -0.75306700 | 1.05470900 |
| C | 0.01198800 | 0.56610000 | -1.32035200 |
| H | 0.86978400 | -1.83577600 | -0.31425100 |
| O | -2.54598500 | 1.08586300 | -0.52270300 |
| C | -4.21988000 | 2.67941000 | -0.81150800 |
| C | -3.66197800 | 3.36800600 | -1.86344700 |
| N | -5.28266900 | 3.43802800 | -0.45643100 |
| H | -2.79008100 | 3.12145400 | -2.44964900 |
| N | -4.40309300 | 4.48432500 | -2.08805000 |
| N | -5.38304800 | 4.52056600 | -1.23817900 |
| C | -3.86670100 | 1.38812400 | -0.14944700 |
| H | -3.95766300 | 1.47597500 | 0.94387400 |
| H | -4.55573300 | 0.59365300 | -0.47375600 |
| N | 1.25136200 | 0.53923200 | -1.74929400 |
| O | 2.02172200 | -0.45912700 | -1.41657900 |
| H | -0.54855300 | 1.43909100 | -1.63901000 |
| C | -6.24519700 | 3.20790300 | 0.61580500 |
| H | -5.73718500 | 3.31024900 | 1.57931400 |
| H | -6.97635100 | 4.01507500 | 0.52706700 |
| C | -6.89224100 | 1.84776600 | 0.49086800 |
| C | -6.86912900 | 0.95056600 | 1.55679400 |
| C | -7.49257400 | 1.46865100 | -0.71223700 |
| C | -7.43781900 | -0.31606700 | 1.43295600 |
| H | -6.39711300 | 1.23275300 | 2.49422400 |

| | | | |
|---|---|---|---|
| C | -8.06155000 | 0.20856200 | -0.85281700 |
| H | -7.50678300 | 2.16030000 | -1.55098900 |
| C | -8.02538300 | -0.67117500 | 0.22575500 |
| H | -7.41552600 | -1.01576700 | 2.26084200 |
| H | -8.52465200 | -0.09020300 | -1.78658400 |
| O | -0.17886000 | -3.60534000 | 1.17380700 |
| C | -0.86267500 | -4.51814000 | 2.00797100 |
| H | -1.76683600 | -4.90317600 | 1.52141200 |
| H | -0.17203100 | -5.34092800 | 2.19282500 |
| H | -1.14049100 | -4.05410500 | 2.96197600 |
| Br | -8.78712300 | -2.39595700 | 0.03232200 |

**TS1(FZ)**

| | | | |
|---|---|---|---|
| N | 8.10777300 | -3.46985400 | -1.06228200 |
| C | 7.38590200 | -2.20748900 | -1.08916100 |
| C | 6.53487700 | -2.11108300 | 0.19101600 |
| O | 5.82280700 | -0.88852000 | 0.26949100 |
| C | 8.27128400 | -0.98126600 | -1.19088600 |
| O | 7.86819800 | 0.11877800 | -1.50634400 |
| O | 4.47978500 | -1.42128100 | -1.81232400 |
| P | 4.31776900 | -0.80233600 | -0.47682800 |
| C | 2.86024700 | -0.95617700 | 1.71420400 |
| O | 4.23972600 | 0.77493300 | -0.22501100 |
| C | 4.23959300 | 1.62923200 | -1.39299300 |
| C | 3.88847800 | 3.02605800 | -0.93911900 |
| H | 8.58321700 | -3.62204400 | -1.94843400 |
| H | 6.69274800 | -2.19061500 | -1.93504600 |
| H | 5.85566200 | -2.96965300 | 0.21301200 |
| H | 7.17982900 | -2.16494200 | 1.07500700 |
| H | 9.34029600 | -1.13665900 | -0.93497000 |
| H | 3.59447600 | -0.32617000 | 2.23028100 |
| H | 2.07158200 | -0.32272000 | 1.29950000 |
| H | 5.24098300 | 1.58659500 | -1.83977300 |
| H | 3.50375500 | 1.24070500 | -2.09757600 |

| | | | |
|---|---|---|---|
| H | 3.87316900 | 3.69995600 | -1.80049300 |
| H | 4.61851600 | 3.40516600 | -0.21715100 |
| H | 2.89688100 | 3.01139000 | -0.48147800 |
| C | 6.83750100 | 3.52380300 | 1.87797100 |
| N | 7.76021800 | 3.61445300 | 0.85918600 |
| C | 6.24994000 | 2.30284900 | 1.75130300 |
| C | 7.74538400 | 2.49482600 | 0.13678600 |
| N | 6.83390400 | 1.69078700 | 0.66762100 |
| H | 5.46887600 | 1.82590800 | 2.32002500 |
| H | 8.35877100 | 2.26835200 | -0.72070000 |
| H | 6.58042100 | 0.75014200 | 0.32254200 |
| H | 6.68437000 | 4.32124300 | 2.58560800 |
| H | 8.83236200 | -3.44952600 | -0.34557900 |
| H | 8.36473500 | 4.40748600 | 0.67861600 |
| O | 3.51018600 | -1.63232200 | 0.62292200 |
| C | 2.30679500 | -2.01651400 | 2.64097400 |
| H | 3.11341700 | -2.63572500 | 3.04326600 |
| H | 1.78439700 | -1.54032700 | 3.47556900 |
| H | 1.60005000 | -2.65916900 | 2.10775400 |
| C | -2.24346800 | -2.49767400 | 0.90416500 |
| C | -2.75869900 | -1.20719100 | 0.71294800 |
| C | -1.95480700 | -0.19025700 | 0.22419200 |
| C | -0.58931900 | -0.42351300 | -0.09432800 |
| C | -0.10285000 | -1.72052300 | 0.08747200 |
| C | -0.91402900 | -2.74447700 | 0.59149800 |
| H | -2.89568200 | -3.27171900 | 1.28993500 |
| H | -3.79986300 | -1.02852600 | 0.95814300 |
| C | 0.23467900 | 0.71142300 | -0.52481700 |
| H | 0.92976600 | -1.92760700 | -0.16069700 |
| O | -2.40484800 | 1.09209500 | 0.01664200 |
| C | -4.01358000 | 2.77009300 | -0.07138100 |
| C | -3.32101400 | 3.65310600 | -0.86674900 |
| N | -5.10191400 | 3.47214300 | 0.32014800 |
| H | -2.38643000 | 3.50823200 | -1.38660600 |

| | | | |
|---|---|---|---|
| N | -4.01348400 | 4.82053900 | -0.91664300 |
| N | -5.09010100 | 4.70603000 | -0.20105700 |
| C | -3.75588500 | 1.34921500 | 0.30928400 |
| H | -3.96626300 | 1.18521200 | 1.37662500 |
| H | -4.41327300 | 0.67810300 | -0.26339600 |
| N | 1.46921100 | 0.72620400 | -0.94083900 |
| O | 2.08138600 | -0.42678400 | -1.11258100 |
| H | -0.21914500 | 1.69464900 | -0.44772300 |
| C | -6.18895400 | 3.04959400 | 1.19815900 |
| H | -5.80055500 | 2.93185800 | 2.21404500 |
| H | -6.90085700 | 3.87840000 | 1.19729500 |
| C | -6.81823100 | 1.76436200 | 0.71254000 |
| C | -6.87617700 | 0.64634200 | 1.54271900 |
| C | -7.31172300 | 1.67691000 | -0.59151900 |
| C | -7.41601900 | -0.55351600 | 1.08312800 |
| H | -6.48917500 | 0.70237000 | 2.55676700 |
| C | -7.85199000 | 0.48735400 | -1.06561200 |
| H | -7.26445500 | 2.54345000 | -1.24632300 |
| C | -7.89434500 | -0.61748000 | -0.21914900 |
| H | -7.45405700 | -1.42582100 | 1.72564800 |
| H | -8.23211200 | 0.41492300 | -2.07832100 |
| O | -0.29517400 | -3.95514700 | 0.74391400 |
| C | -1.07289900 | -5.01291900 | 1.26793700 |
| H | -1.92104200 | -5.24563000 | 0.61328900 |
| H | -0.41300100 | -5.87835400 | 1.32616100 |
| H | -1.44864200 | -4.77216500 | 2.26947500 |
| Br | -8.60943400 | -2.24966800 | -0.86519900 |

**IN(FZ)**

| | | | |
|---|---|---|---|
| N | -8.96954500 | 1.16929400 | -2.04550400 |
| C | -8.04072700 | 0.19583900 | -1.48624600 |
| C | -6.67211900 | 0.86250400 | -1.28022000 |
| O | -5.76359300 | -0.09101500 | -0.80251900 |
| C | -8.47307200 | -0.36762400 | -0.15184300 |

| | | | |
|---|---|---|---|
| O | -8.22980600 | -1.49327300 | 0.22862800 |
| O | -4.07068700 | 0.51244600 | -2.50512900 |
| P | -4.01275800 | 0.26100900 | -1.02723200 |
| C | -3.43924000 | 2.52322300 | 0.43415300 |
| O | -3.76103500 | -1.21899800 | -0.36851000 |
| C | -4.46691700 | -2.37300700 | -0.83238600 |
| C | -3.60436700 | -3.57921700 | -0.51927900 |
| H | -9.88343100 | 0.74421400 | -2.18660000 |
| H | -7.91115400 | -0.63839500 | -2.18287500 |
| H | -6.34355800 | 1.26367100 | -2.24471700 |
| H | -6.77163300 | 1.70269000 | -0.57306700 |
| H | -9.00944200 | 0.34834700 | 0.50807100 |
| H | -3.94218100 | 3.01237300 | 1.27388800 |
| H | -2.46286800 | 2.17183600 | 0.77464600 |
| H | -5.44163100 | -2.44012600 | -0.33717500 |
| H | -4.64891900 | -2.28605200 | -1.90914800 |
| H | -4.09927200 | -4.49703300 | -0.84933400 |
| H | -3.42654400 | -3.64928500 | 0.55803100 |
| H | -2.63853600 | -3.49705300 | -1.02522000 |
| C | -4.71806500 | -0.91888900 | 3.80745000 |
| N | -6.00270900 | -1.41474300 | 3.83978900 |
| C | -4.51530600 | -0.46996100 | 2.53897900 |
| C | -6.56523600 | -1.27613400 | 2.63875600 |
| N | -5.67743200 | -0.70151100 | 1.83806100 |
| H | -3.65911700 | -0.01701200 | 2.06719500 |
| H | -7.56066000 | -1.58306500 | 2.35653200 |
| H | -5.81498800 | -0.46682400 | 0.81527500 |
| H | -4.07831800 | -0.93057500 | 4.67385800 |
| H | -9.10869300 | 1.93558400 | -1.38755600 |
| H | -6.45928000 | -1.82561100 | 4.64518500 |
| O | -4.28296100 | 1.41266200 | 0.11505500 |
| C | -3.30720000 | 3.48584400 | -0.73159500 |
| H | -4.29075200 | 3.86473400 | -1.02626900 |
| H | -2.67291700 | 4.33225600 | -0.45000800 |

| | | | |
|---|---|---|---|
| H | -2.85941700 | 2.98249600 | -1.59306400 |
| C | 2.03103900 | 2.65490800 | 0.92581100 |
| C | 2.49939700 | 1.35282100 | 0.71738600 |
| C | 1.74402700 | 0.44229800 | -0.00941100 |
| C | 0.49308800 | 0.82009200 | -0.54330000 |
| C | 0.04794700 | 2.12060100 | -0.33713800 |
| C | 0.80396500 | 3.04045500 | 0.39594800 |
| H | 2.64035300 | 3.34150300 | 1.50116200 |
| H | 3.45908500 | 1.07535100 | 1.13875900 |
| C | -0.24939900 | -0.16018700 | -1.36081500 |
| H | -0.89450200 | 2.44128200 | -0.76166600 |
| O | 2.12448100 | -0.85100200 | -0.24785300 |
| C | 3.62445200 | -2.62942200 | -0.29370700 |
| C | 2.96104200 | -3.40442700 | -1.21632400 |
| N | 4.62836800 | -3.42697400 | 0.13761400 |
| H | 2.08885000 | -3.16403800 | -1.80439800 |
| N | 3.58860900 | -4.60648800 | -1.29745900 |
| N | 4.59727800 | -4.61396400 | -0.48141800 |
| C | 3.41261400 | -1.23018700 | 0.18246500 |
| H | 3.48965700 | -1.17494600 | 1.27818300 |
| H | 4.17479800 | -0.55979100 | -0.24059400 |
| N | -1.51269700 | -0.30501500 | -1.51508900 |
| O | -2.27820100 | 0.55382800 | -0.78290800 |
| H | 0.34410100 | -0.88032000 | -1.91867900 |
| C | 5.65978800 | -3.13488700 | 1.12719500 |
| H | 5.19133500 | -3.02648000 | 2.10964200 |
| H | 6.29809700 | -4.02135600 | 1.14943400 |
| C | 6.43952800 | -1.89286900 | 0.76176300 |
| C | 6.59259300 | -0.85617000 | 1.68026800 |
| C | 7.00100400 | -1.77057400 | -0.51191900 |
| C | 7.30523800 | 0.29318800 | 1.34268100 |
| H | 6.15222400 | -0.93895200 | 2.67030100 |
| C | 7.71059600 | -0.62920200 | -0.86507600 |
| H | 6.87752000 | -2.57136300 | -1.23678400 |

| | | | |
|---|---|---|---|
| C | 7.85580400 | 0.39092300 | 0.07150100 |
| H | 7.42634500 | 1.09890900 | 2.05770700 |
| H | 8.14760300 | -0.53056400 | -1.85228600 |
| O | 0.25241400 | 4.27948100 | 0.53128600 |
| C | 0.99480300 | 5.23876100 | 1.26099700 |
| H | 0.39997300 | 6.15146800 | 1.25615400 |
| H | 1.15520600 | 4.91254700 | 2.29507200 |
| H | 1.96487400 | 5.43147300 | 0.78856100 |
| Br | 8.83198600 | 1.94467300 | -0.40236200 |

**TS2(FZ)**

| | | | |
|---|---|---|---|
| N | -8.81833100 | 1.36259400 | -2.08559200 |
| C | -7.93462500 | 0.30720300 | -1.60985500 |
| C | -6.64513400 | 0.93825800 | -1.06406900 |
| O | -5.76364800 | -0.07372800 | -0.64449400 |
| C | -8.52628200 | -0.54038300 | -0.50432500 |
| O | -8.28641500 | -1.71651000 | -0.33868900 |
| O | -4.06364300 | 0.46436400 | -2.38488400 |
| P | -3.89870900 | 0.25389300 | -0.91359800 |
| C | -3.33273700 | 2.52829100 | 0.49217600 |
| O | -3.72444900 | -1.21539900 | -0.24216600 |
| C | -4.41625200 | -2.35569900 | -0.77000800 |
| C | -3.49586500 | -3.55061100 | -0.62786000 |
| H | -9.67685300 | 0.96222100 | -2.45730900 |
| H | -7.66151800 | -0.35442100 | -2.43789400 |
| H | -6.18580800 | 1.52403400 | -1.86528000 |
| H | -6.88279600 | 1.61684400 | -0.22894800 |
| H | -9.18348500 | 0.01002600 | 0.20440400 |
| H | -3.81627900 | 3.02081300 | 1.34046000 |
| H | -2.34708700 | 2.18120400 | 0.80799400 |
| H | -5.34976700 | -2.49852500 | -0.21581500 |
| H | -4.67369800 | -2.17575300 | -1.81825300 |
| H | -3.98450000 | -4.45145300 | -1.00992600 |
| H | -3.24081100 | -3.71430900 | 0.42300300 |

| | | | |
|---|---|---|---|
| H | -2.57191100 | -3.38428700 | -1.18806300 |
| C | -5.54844600 | -0.66685800 | 3.93856000 |
| N | -6.64532600 | -1.45181800 | 3.66916900 |
| C | -5.16678600 | -0.13661200 | 2.74210600 |
| C | -6.91093200 | -1.39414200 | 2.35754500 |
| N | -6.02816100 | -0.59894300 | 1.77360000 |
| H | -4.35378700 | 0.52648900 | 2.49413400 |
| H | -7.71562200 | -1.91503300 | 1.86022200 |
| H | -5.92982000 | -0.35190800 | 0.59637600 |
| H | -5.14968000 | -0.56170700 | 4.93393900 |
| H | -9.09528500 | 1.96116800 | -1.30801500 |
| H | -7.17219000 | -1.98926700 | 4.34601900 |
| O | -4.18209100 | 1.40579400 | 0.20916500 |
| C | -3.24045100 | 3.47732500 | -0.68761800 |
| H | -4.23276700 | 3.85253600 | -0.95525100 |
| H | -2.59855200 | 4.32631800 | -0.43281800 |
| H | -2.81669400 | 2.96884000 | -1.55847900 |
| C | 2.13460800 | 2.66228000 | 0.83150000 |
| C | 2.59416900 | 1.35241000 | 0.65750600 |
| C | 1.82611800 | 0.42421400 | -0.03327800 |
| C | 0.57091900 | 0.79348600 | -0.56184300 |
| C | 0.13397900 | 2.10195900 | -0.39059300 |
| C | 0.90317700 | 3.03921100 | 0.30492000 |
| H | 2.75402700 | 3.36284900 | 1.37855000 |
| H | 3.55719900 | 1.08273300 | 1.07616600 |
| C | -0.18640900 | -0.20781600 | -1.33920700 |
| H | -0.81192500 | 2.41628000 | -0.81247200 |
| O | 2.19680900 | -0.87666400 | -0.23905400 |
| C | 3.68762800 | -2.66444900 | -0.24329300 |
| C | 3.01858900 | -3.45786600 | -1.14594500 |
| N | 4.68808000 | -3.45682200 | 0.20575300 |
| H | 2.14722000 | -3.22675800 | -1.73895500 |
| N | 3.63897700 | -4.66513100 | -1.19836300 |
| N | 4.64870500 | -4.65850400 | -0.38367300 |

| | | | |
|---|---|---|---|
| C | 3.48456500 | -1.25226900 | 0.19711400 |
| H | 3.56422600 | -1.16804900 | 1.29063900 |
| H | 4.24897300 | -0.59753100 | -0.24591000 |
| N | -1.45116500 | -0.35686300 | -1.46439500 |
| O | -2.19336200 | 0.53232300 | -0.72879900 |
| H | 0.39378000 | -0.94379600 | -1.89028000 |
| C | 5.71961100 | -3.15016100 | 1.19122500 |
| H | 5.25221200 | -3.03546900 | 2.17344100 |
| H | 6.36242000 | -4.03317100 | 1.22021500 |
| C | 6.49246300 | -1.90738300 | 0.81468100 |
| C | 6.63430300 | -0.85984100 | 1.72266400 |
| C | 7.05822100 | -1.79439800 | -0.45793100 |
| C | 7.33942300 | 0.29123900 | 1.37569500 |
| H | 6.19085400 | -0.93552600 | 2.71191000 |
| C | 7.76063700 | -0.65143200 | -0.82044900 |
| H | 6.94381100 | -2.60366200 | -1.17482500 |
| C | 7.89440900 | 0.37963800 | 0.10572700 |
| H | 7.45186500 | 1.10533700 | 2.08262200 |
| H | 8.20104500 | -0.56009300 | -1.80686400 |
| Br | 8.86249300 | 1.93508200 | -0.37914800 |
| O | 0.35937800 | 4.28404400 | 0.40985600 |
| C | 1.11835400 | 5.26264900 | 1.09590700 |
| H | 1.29324200 | 4.96978600 | 2.13751200 |
| H | 2.08171600 | 5.43424500 | 0.60217200 |
| H | 0.52800800 | 6.17790200 | 1.07044300 |

**C2(FZ)**

| | | | |
|---|---|---|---|
| N | -10.08551200 | 0.00005700 | -1.38825200 |
| C | -8.66055900 | -0.28041800 | -1.23141400 |
| C | -7.97729000 | 0.93942400 | -0.59808200 |
| O | -6.58214700 | 0.79031700 | -0.52370200 |
| C | -8.41091600 | -1.49196900 | -0.35900000 |
| O | -7.68059000 | -2.41629200 | -0.64076200 |
| O | -3.84007900 | 0.80041400 | -2.81587600 |

| | | | |
|---|---|---|---|
| P | -3.49192400 | 0.81672300 | -1.38460700 |
| C | -4.03166700 | 2.15620100 | 0.82724200 |
| O | -3.93462700 | -0.43987200 | -0.52662000 |
| C | -4.69913000 | -1.51576400 | -1.12566900 |
| C | -3.78525400 | -2.49017000 | -1.83566600 |
| H | -10.55764400 | -0.79683000 | -1.81024600 |
| H | -8.21010900 | -0.46563500 | -2.21202500 |
| H | -8.20875300 | 1.80918600 | -1.22045400 |
| H | -8.41351300 | 1.11673200 | 0.39862500 |
| H | -8.97177100 | -1.48565500 | 0.60125900 |
| H | -4.38892300 | 1.19041800 | 1.19521000 |
| H | -3.01882900 | 2.33157300 | 1.20331900 |
| H | -5.21743700 | -1.98080100 | -0.28676900 |
| H | -5.44948800 | -1.08844900 | -1.79231200 |
| H | -4.37594500 | -3.32470300 | -2.22514200 |
| H | -3.03236100 | -2.88443000 | -1.14804800 |
| H | -3.27445500 | -2.00045800 | -2.66870700 |
| C | -6.15089300 | -0.75883600 | 4.12304500 |
| N | -6.35437200 | -2.00107400 | 3.57636400 |
| C | -6.20227000 | 0.11068800 | 3.06834600 |
| C | -6.51881600 | -1.84992200 | 2.24392200 |
| N | -6.43039900 | -0.58009500 | 1.89955200 |
| H | -6.09658700 | 1.18627200 | 3.07917400 |
| H | -6.71577700 | -2.67414000 | 1.57166900 |
| H | -6.39548500 | 0.24916500 | 0.28228900 |
| H | -5.99495400 | -0.61476100 | 5.18007400 |
| H | -10.51548700 | 0.13555600 | -0.47332400 |
| H | -6.38260800 | -2.87749500 | 4.07992900 |
| O | -3.96864600 | 2.11208800 | -0.61618700 |
| C | -4.97912200 | 3.27058600 | 1.20781800 |
| H | -5.01350200 | 3.37379700 | 2.29620600 |
| H | -4.64722100 | 4.22155800 | 0.78282600 |
| H | -5.98076700 | 3.04029200 | 0.83565700 |
| C | 2.63060400 | 2.75485400 | 0.52683900 |

| | | | |
|---|---|---|---|
| C | 3.00193500 | 1.41170700 | 0.43303800 |
| C | 2.14643700 | 0.48395300 | -0.14658100 |
| C | 0.88364400 | 0.89288700 | -0.63619300 |
| C | 0.52786400 | 2.23709500 | -0.53499900 |
| C | 1.39473400 | 3.17045900 | 0.03936600 |
| H | 3.32219200 | 3.45447700 | 0.98075600 |
| H | 3.96883000 | 1.11522100 | 0.82197400 |
| C | 0.05694400 | -0.13739000 | -1.28610000 |
| H | -0.42196000 | 2.58797800 | -0.91407700 |
| O | 2.43934900 | -0.84439900 | -0.27174700 |
| C | 3.84621200 | -2.70606000 | -0.20744900 |
| C | 3.16223200 | -3.51045600 | -1.08883300 |
| N | 4.78443100 | -3.52691100 | 0.31939300 |
| H | 2.32625900 | -3.26726300 | -1.72624800 |
| N | 3.71195200 | -4.75192800 | -1.05165800 |
| N | 4.69352600 | -4.75569400 | -0.20330100 |
| C | 3.72205000 | -1.26107500 | 0.14713800 |
| H | 3.84201800 | -1.11268400 | 1.22942700 |
| H | 4.50175300 | -0.67551600 | -0.36047000 |
| N | -1.19675400 | -0.20027300 | -1.52283100 |
| O | -1.89465000 | 0.92540600 | -1.04874000 |
| H | 0.57910800 | -1.02619400 | -1.63007000 |
| C | 5.79050600 | -3.22746600 | 1.33372200 |
| H | 5.29084500 | -3.06635700 | 2.29368700 |
| H | 6.39877500 | -4.13178800 | 1.41259900 |
| C | 6.62125300 | -2.02362000 | 0.95336800 |
| C | 6.76892700 | -0.96084200 | 1.84276400 |
| C | 7.23100100 | -1.95770800 | -0.30188700 |
| C | 7.51914100 | 0.16028900 | 1.49320900 |
| H | 6.29210200 | -0.99986200 | 2.81863000 |
| C | 7.97908300 | -0.84437300 | -0.66713800 |
| H | 7.11362800 | -2.77872000 | -1.00481800 |
| C | 8.11402000 | 0.20403600 | 0.23903900 |
| H | 7.63369200 | 0.98682600 | 2.18526800 |

| | | | |
|---|---|---|---|
| H | 8.45130800 | -0.78861300 | -1.64148300 |
| O | 0.94025000 | 4.45187200 | 0.07205600 |
| C | 1.79122700 | 5.42754500 | 0.64635100 |
| H | 1.25470700 | 6.37337200 | 0.58048700 |
| H | 2.00240400 | 5.19939400 | 1.69710500 |
| H | 2.73513600 | 5.50352400 | 0.09466800 |
| Br | 9.13220900 | 1.72478300 | -0.25162800 |

# Spectra

## FT-IR spectra

**S20.** O-propargylated salicylaldehyde

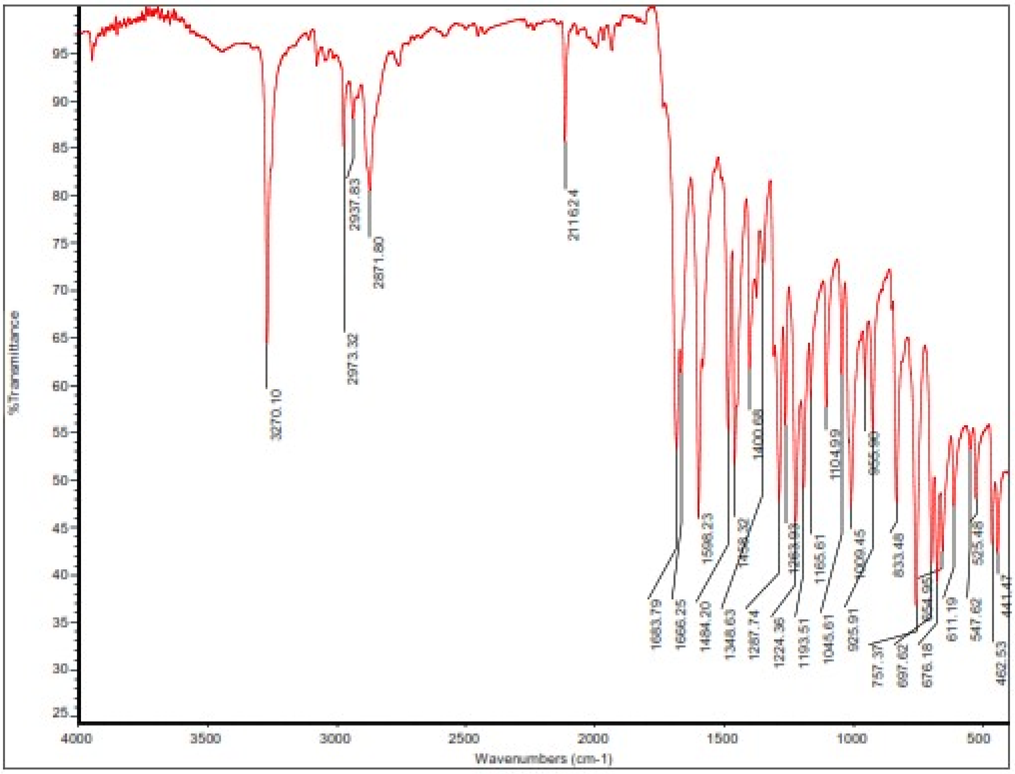


**S21.** O-propargylated salicylaldoxime

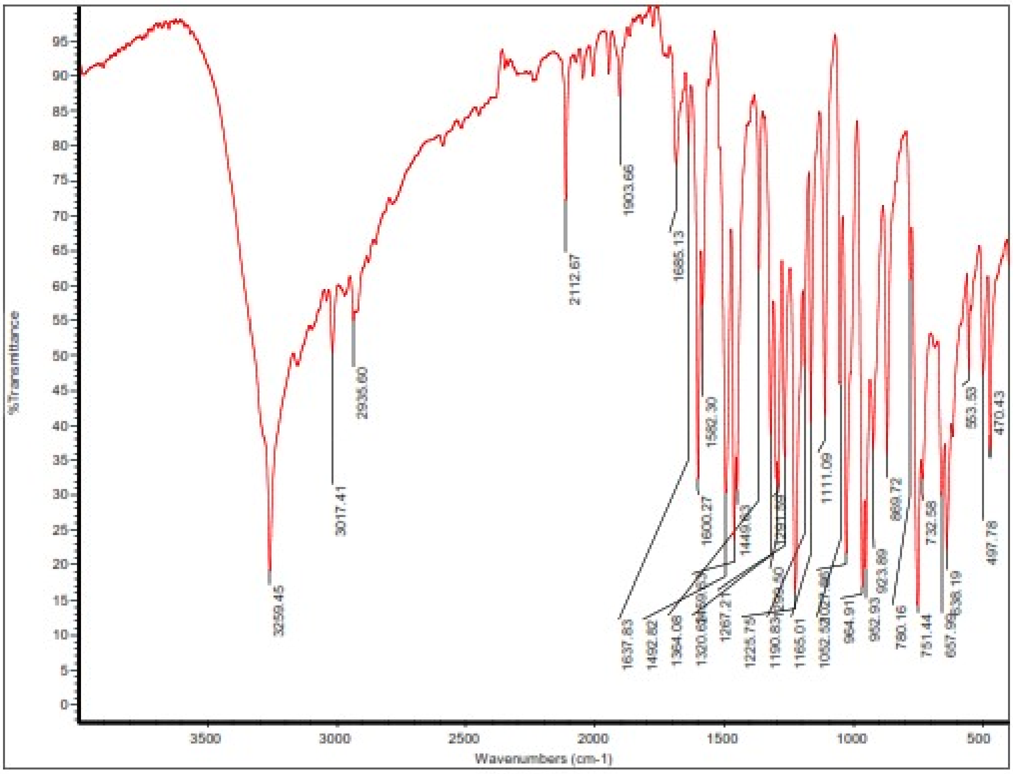

**S22.** 2-((1-benzyl-1H-1,2,3-triazol-5-yl)methoxy)benzaldehyde oxime (A)

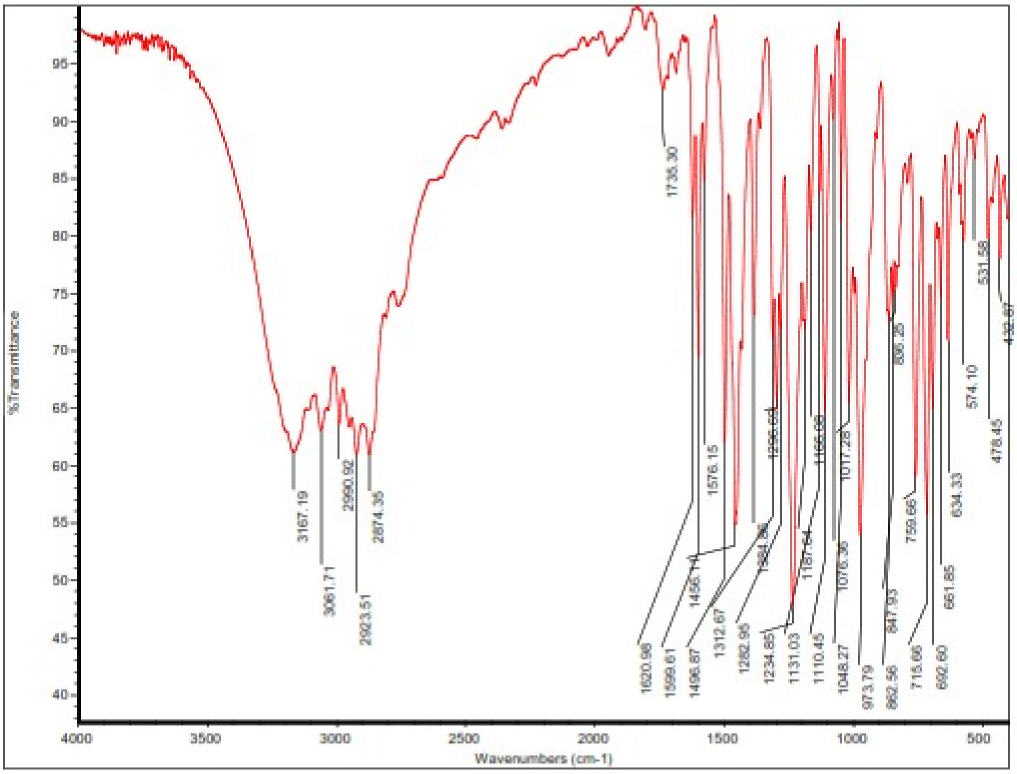


**S23.** 2-((1-(4-bromobenzyl)-1H-1,2,3-triazol-5-yl)methoxy)benzaldehyde oxime (B)

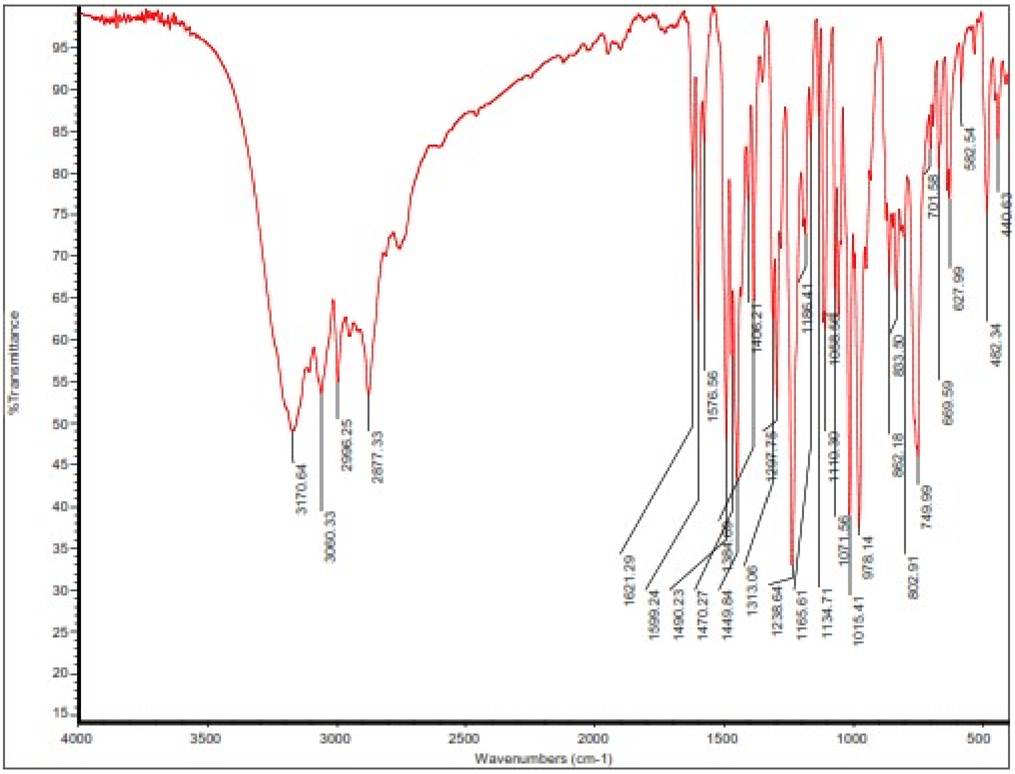

NMR spectra

**S24.** O-propargylated salicylaldehyde

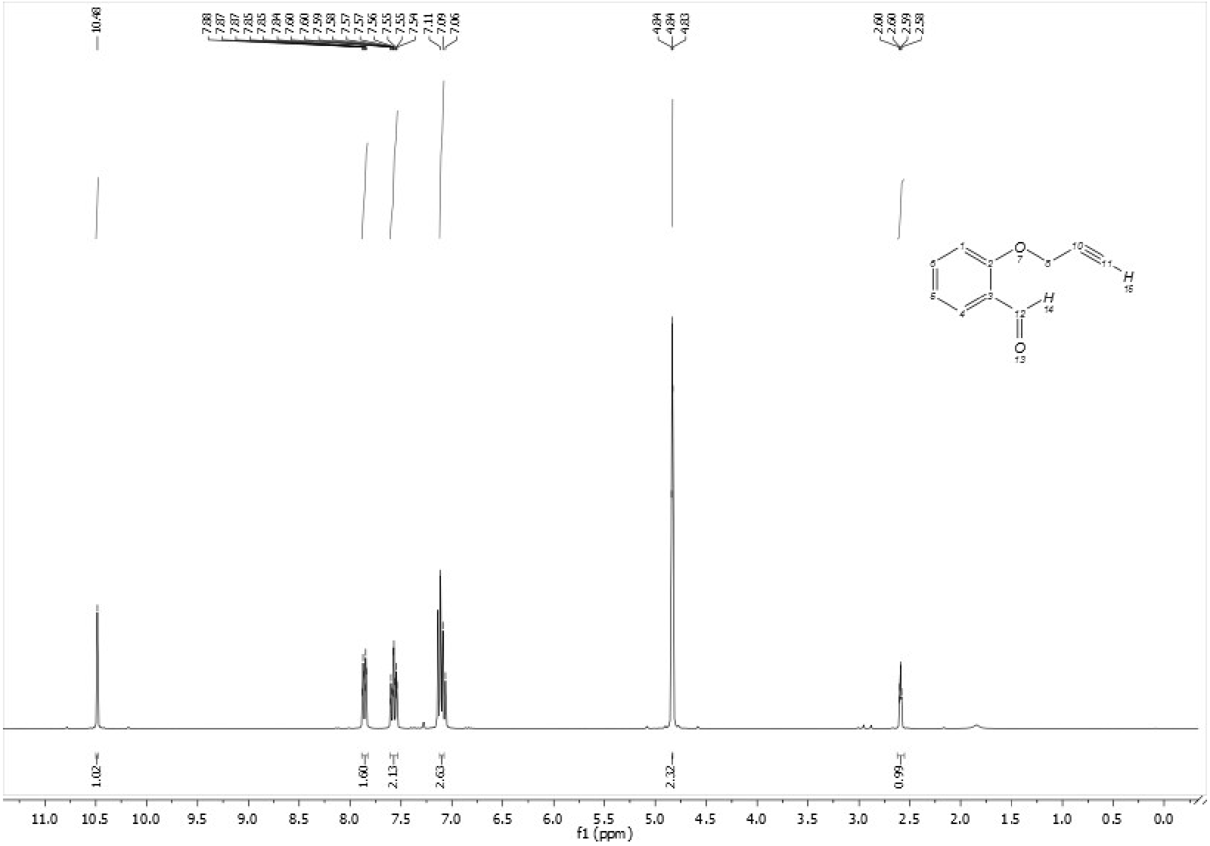

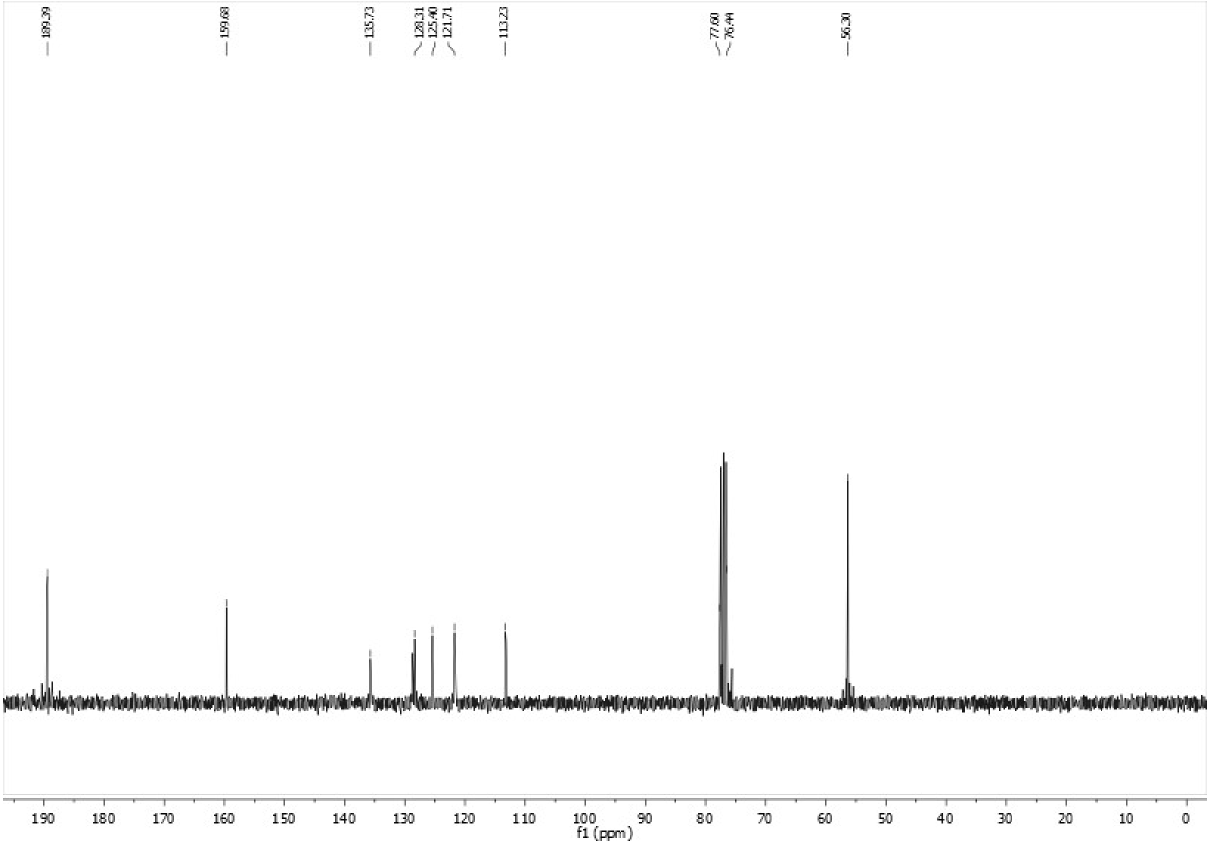

**S25.** O-propargylated salicylaldoxime

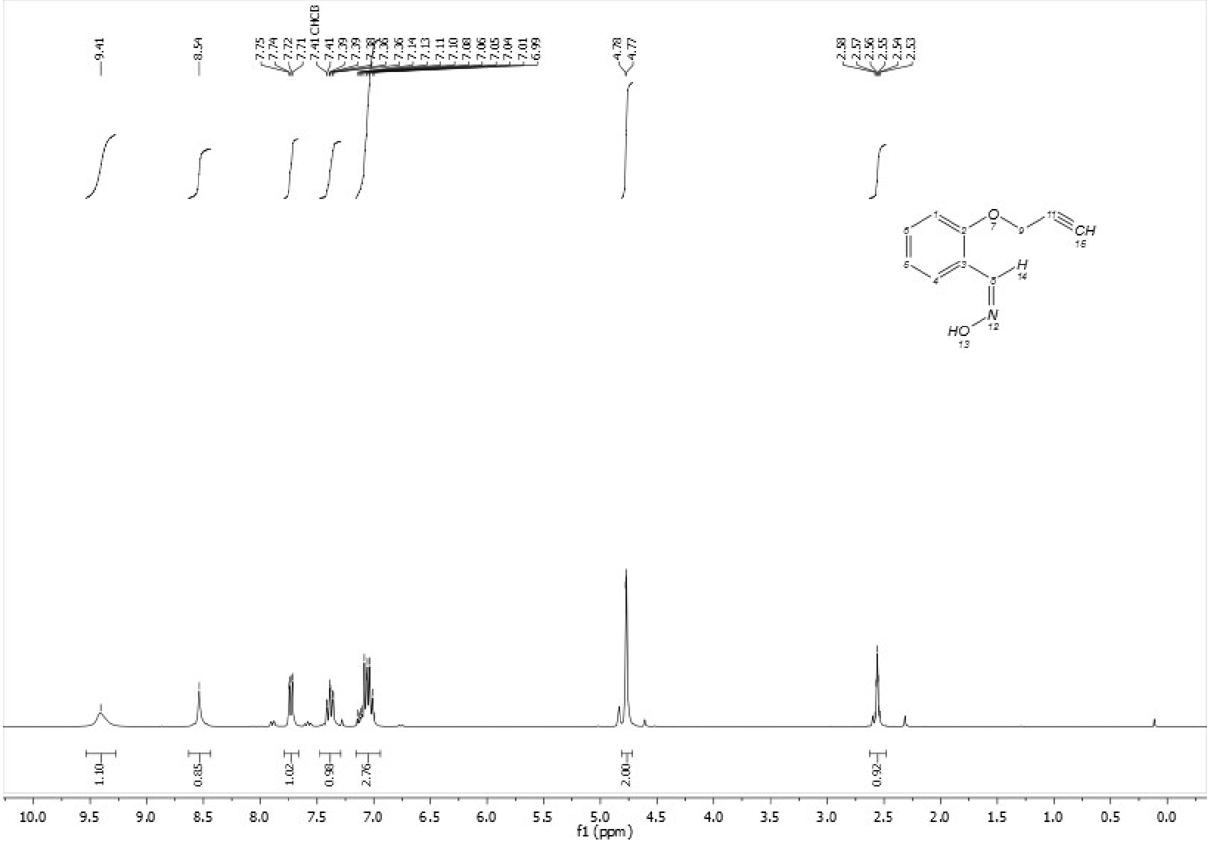


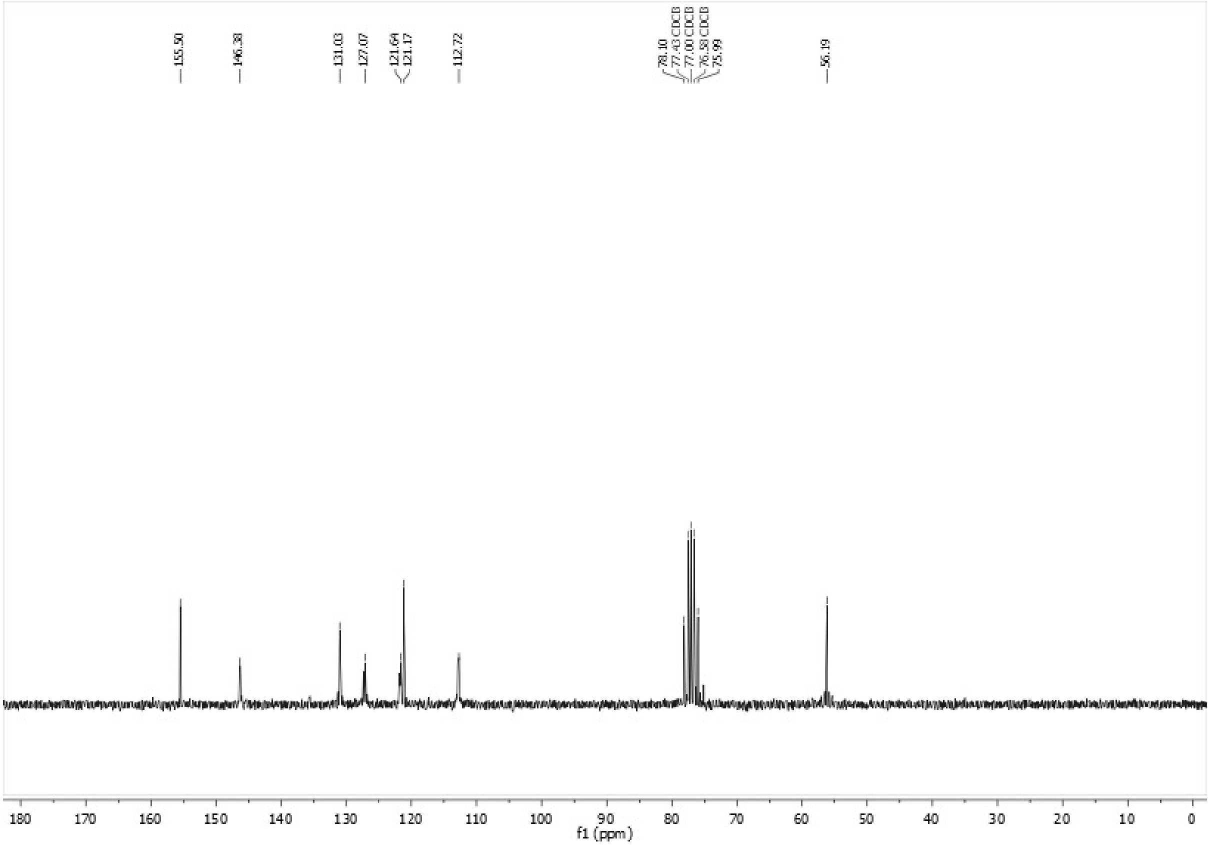

**S26.** 2-((1-benzyl-1H-1,2,3-triazol-5-yl)methoxy)benzaldehyde oxime (A)

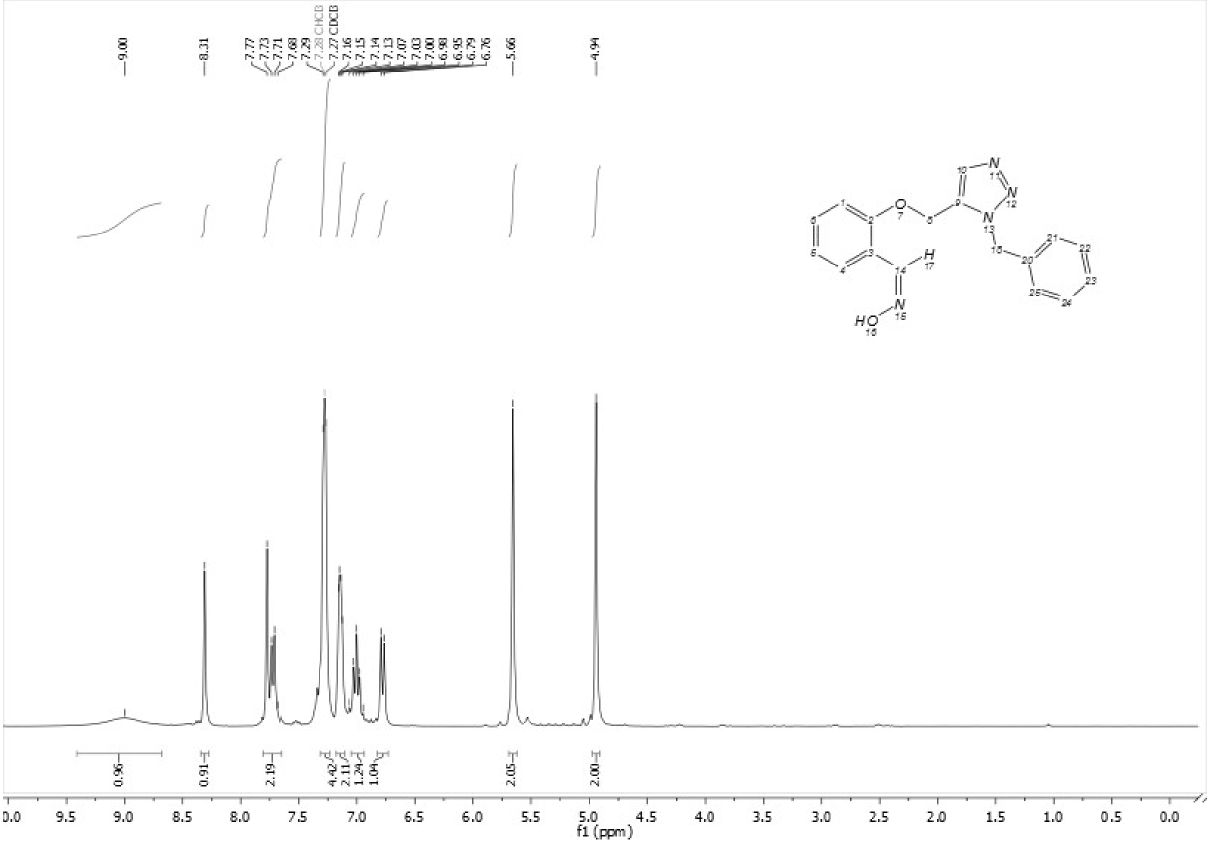


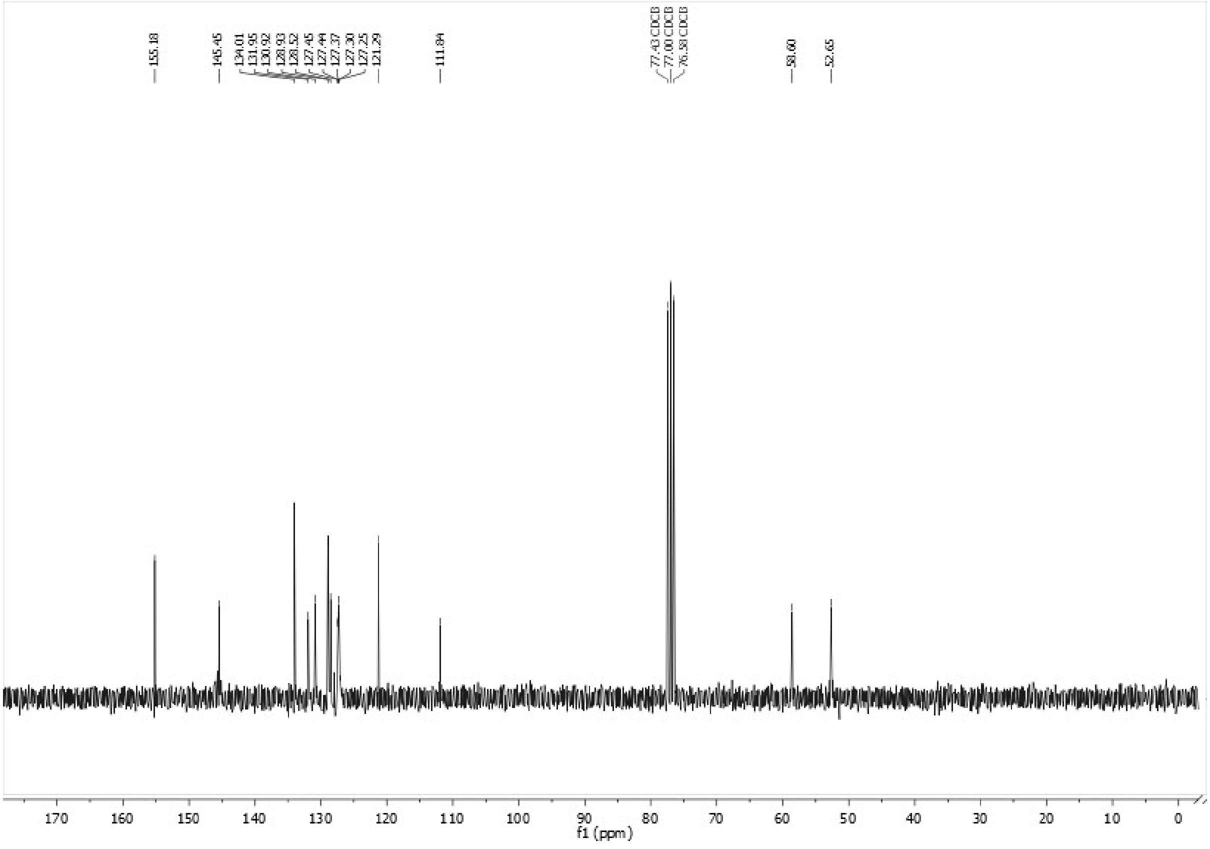

S27. 2-((1-(4-bromobenzyl)-1H-1,2,3-triazol-5-yl)methoxy)benzaldehyde oxime (B)

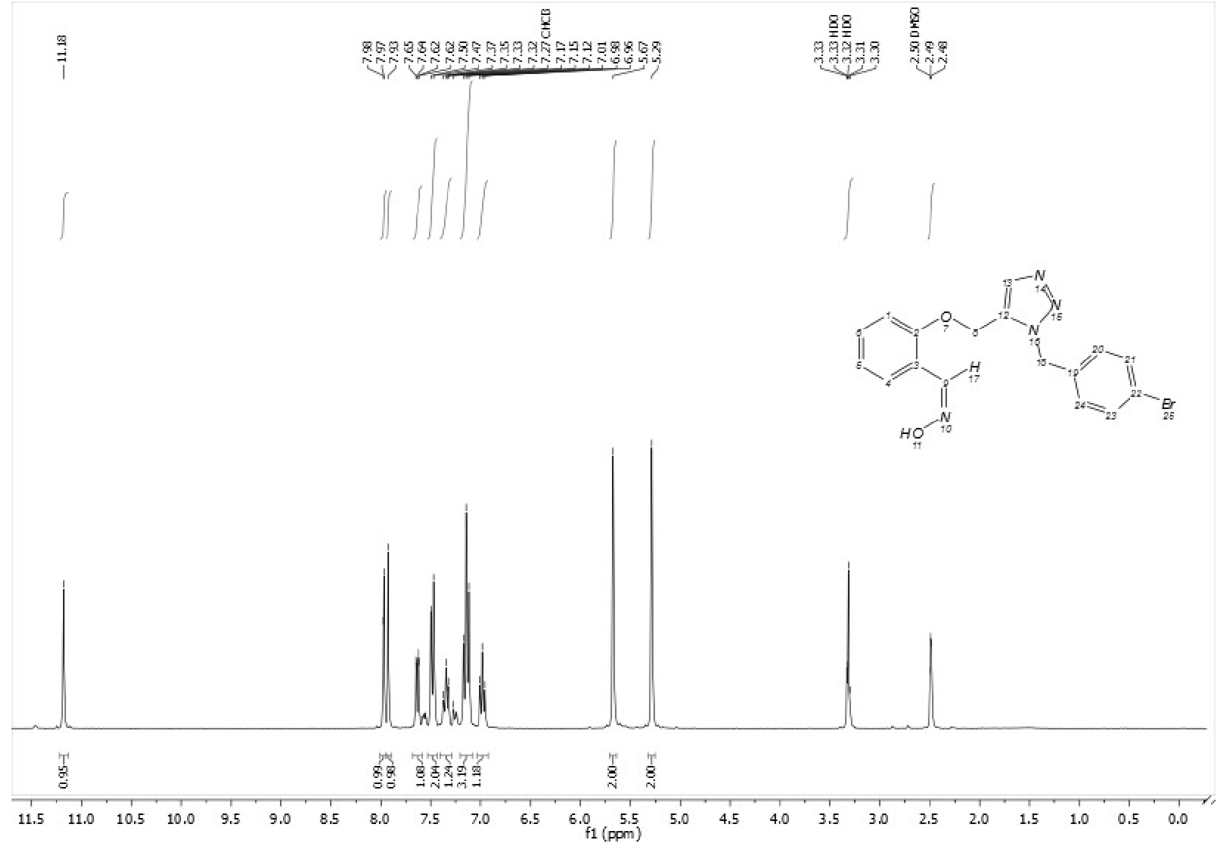

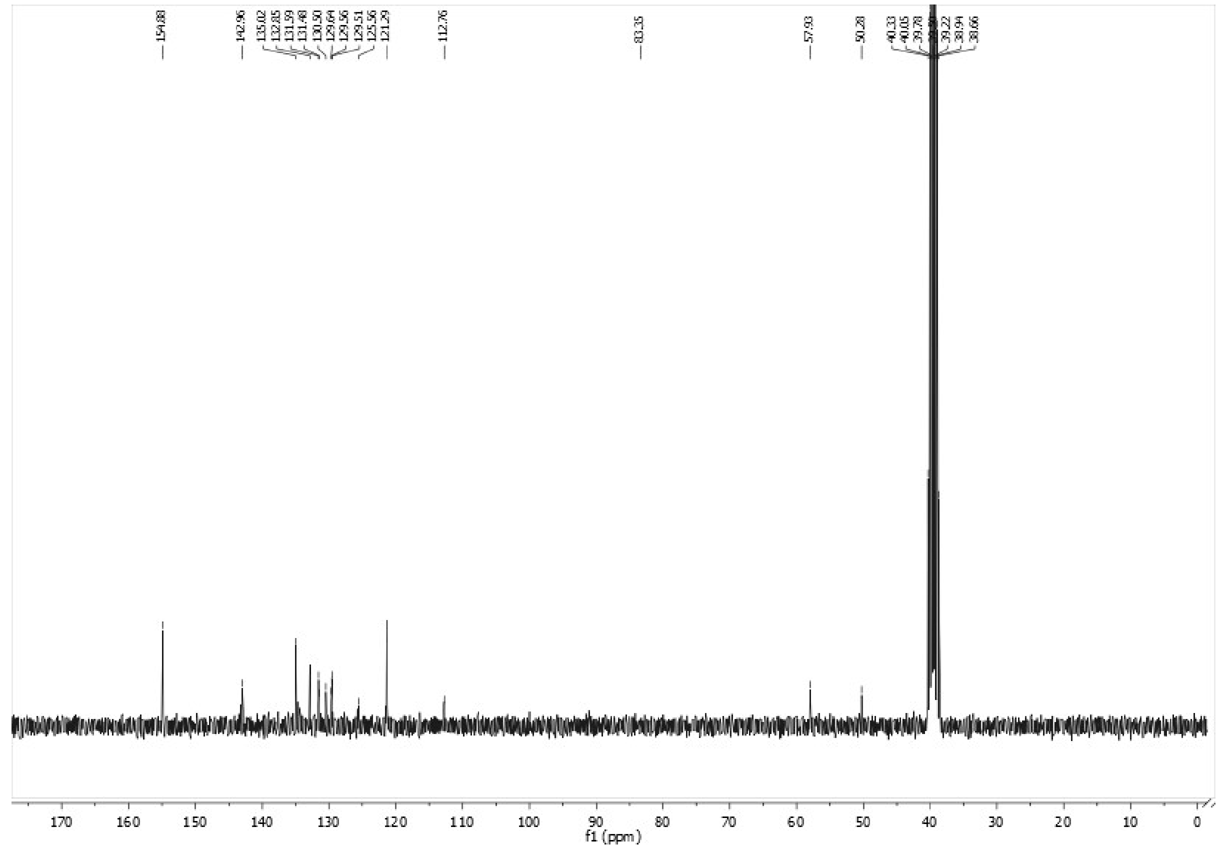

# Determination of reactivation parameters

In order to determine the reactivation rate constant ($k_{+2}$), dissociation constant ($K_{ox}$) and second order reactivation rate constant ($k_r$) of the selected reactivators, the reactivation rate at different time intervals and at different concentrations were measured. Oxime reactivation of phosphylated cholinesterases proceeds according to following scheme.

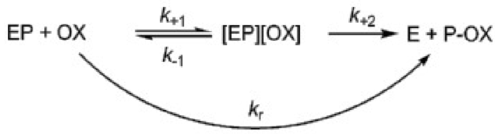


In this scheme, EP is the phosphylated enzyme, EP-OX is the reversible Michaelis-type complex between EP and the oxime (OX), E is the active enzyme and P-OX the phosphylated oxime. $K_{ox}$ is equal to the ratio ($k_{-1}$+ $k_{+2}$)/ $k_{+1}$, and it typically approximates the dissociation constant of the EP-OX complex, where from it follows that: $k_r = k_{+2}/ K_{ox}$.
Since $k_{obs}$ vs [OX] was linear, the slope corresponded to overall second-order rate constant of reactivation ($k_r$); in this case, maximum first-order rate constant ($k_{+2}$) and the dissociation constant of the phosphorylated enzyme-oxime complex ($K_{ox}$) could not be determined.

## S28. Plot of reactivation *vs* time for compounds A and B.

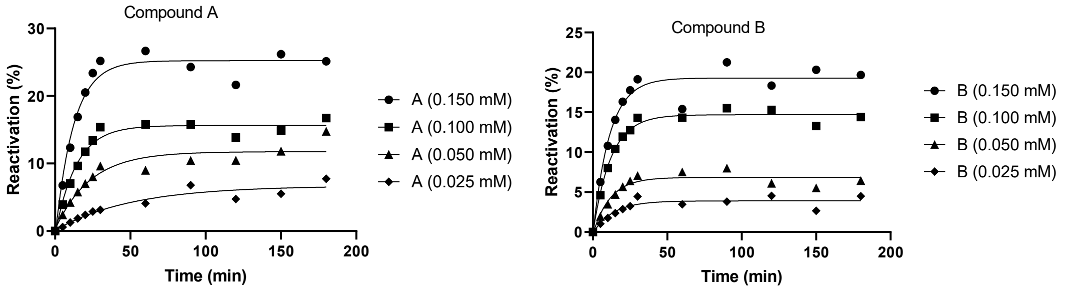


## S29. Plot of log(100-react.%) *vs* time for compounds A and B

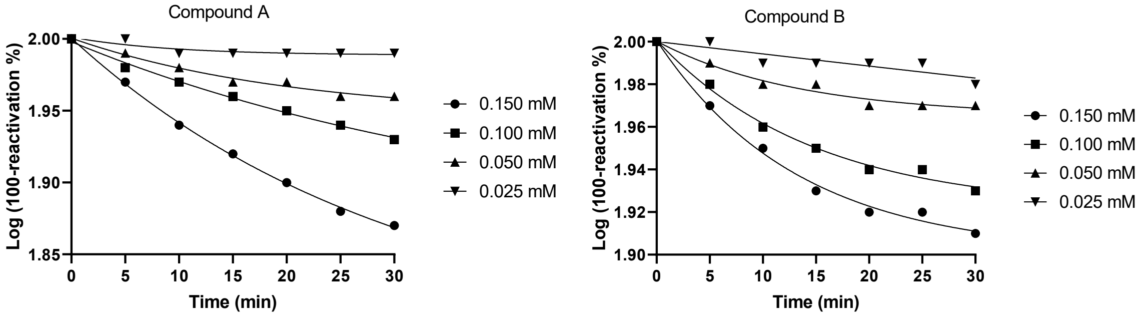


## S30. Plot of $K_{obs}$ *vs* concentration for compounds A and B

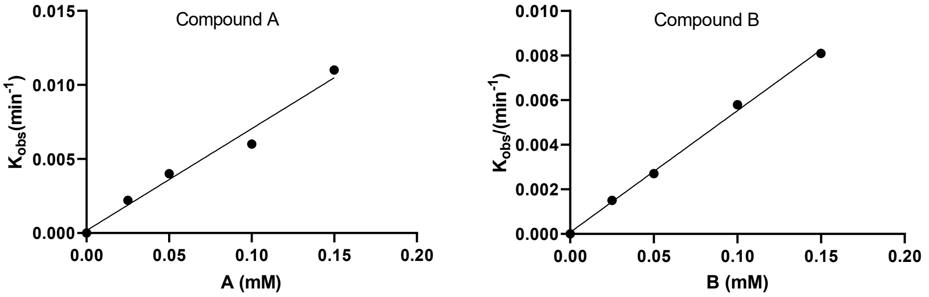

Supplement: RA-013-D3RA05658A-s001 [file RA-013-D3RA05658A-s001.pdf]
